# A unified strategy for the synthesis of aldohexoses by boronate assisted assembly of CH_2_X_2_ derived C_1_-building blocks†‡

**DOI:** 10.1039/d3sc03778a

**Published:** 2023-09-04

**Authors:** Sujenth Kirupakaran, Glib Arago, Christoph Hirschhäuser

**Affiliations:** a University of Duisburg-Essen Universitätsstr. 5-7 45117 Essen Germany Christoph.hirschhäuser@uni-due.de

## Abstract

A synthetic strategy for all aldohexoses with individually addressable protecting groups from dihalomethane C_1_-units is reported. The underlying synthesis of C_6_-sugar alcohols relies on three consecutive Matteson sequences, vinylation and bishydroxylation. *Erythro* and *threo* isomers have been realized for every glycol motif by strategic variation of the sequence.

## Abstract 2

The synthesis of aldohexoses from CH_2_X_2_ derived C_1_-building blocks relies on Matteson homologation and strategically applied, boron mediated olefinations. A 25 min video tutorial is available *via* QR-code or this link: https://youtu.be/vXy5oVavJUU.

## Graphical abstract

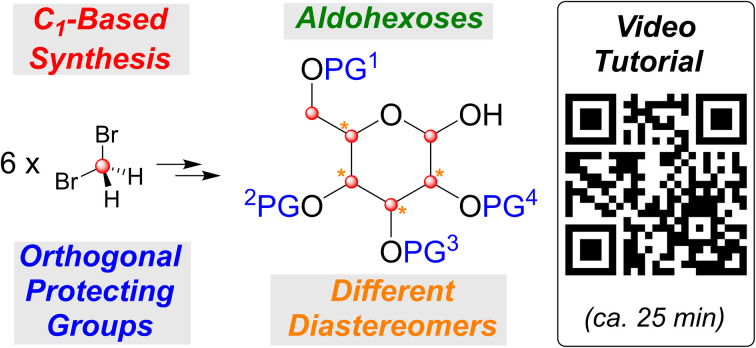

Carbohydrates are of immense biological importance as a source of energy and as complex chiral scaffolds that participate in numerous recognition processes.^1^ Deciphering this “glycocode” is a task, which requires modern analytics as well as organic synthesis.^2^ Nowadays oligosaccharides can even be prepared in an automated manner from orthogonally protected monosaccharides.^3^ Syntheses of the latter often still rely on ex-chiral pool strategies, each of which has to face the challenge of differentiating five similar hydroxyl groups.^4^*De novo* syntheses, such as the ones developed by Sharpless,^5^ Danishefsky^6^ or MacMillan^7^ approach this problem from the bottom up. Strategies based on C_1_-building blocks, like those by Fischer,^8^ Dondoni^9^ and Matteson^10^ could allow for maximal protecting group variability and enable isothopic labelling of individual atoms.^11^ However, each of these methods has its individual limitations^12^ and no unified C_1_-based strategy to aldohexoses had been reported until now. One key to our route is the Matteson homologation (MH) shown in Scheme 1A.^13^ This sequence employs chiral boronic esters (1), which react with a lithiated dihalomethane and ZnCl_2_ at low temperatures. In the resulting ate complex (2) electrostatic interactions^14^ between the zinc bound chloride atoms and the carbenoid C–H favour an antiperiplanar arrangement of one C–X-bond relative to the boronate R-group. Upon warming 1,2-rearrangement results in the diastereoselective formation of α-halo boronates (3). Reaction with various nucleophiles yields α-chiral boronates (4) under stereochemical inversion. Thus MHs are highly useful for preparing heteroatom rich motifs.^16^

**Scheme 1:**
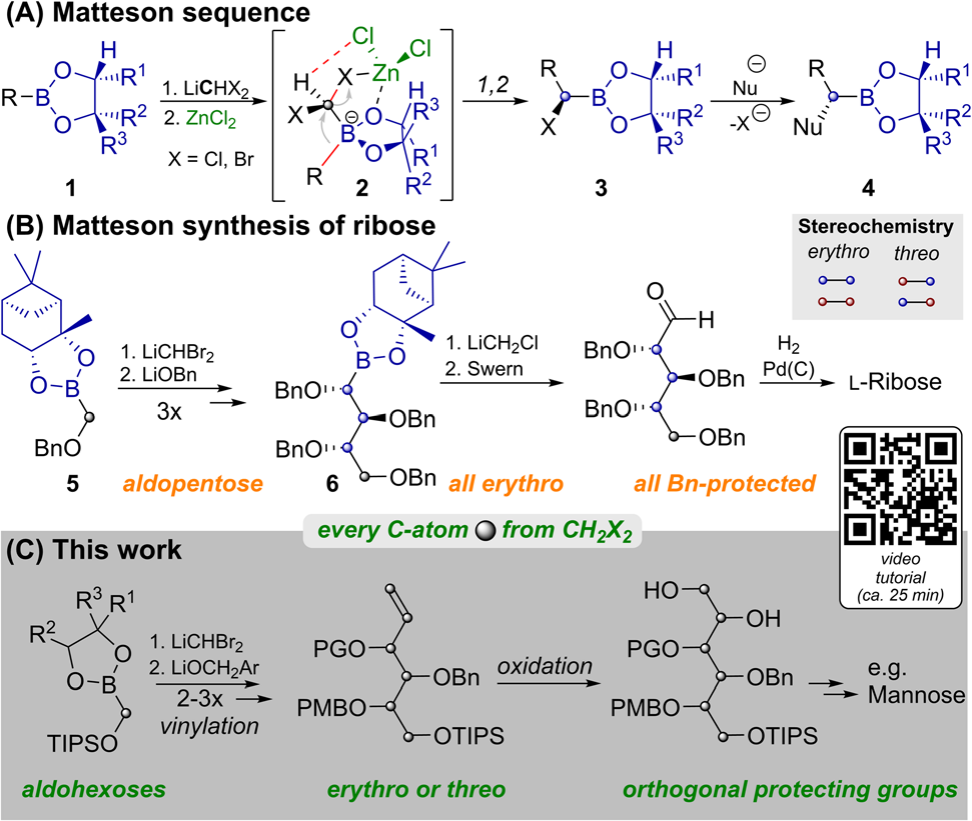
Matteson reactions in the synthesis of monosaccharides. YouTube tutorial: https://youtu.be/vXy5oVavJUU.

As shown in Scheme 1B, iterative MH and substitution with alkoxides can lead to carbohydrate like structures. This was applied by Matteson to the synthesis of l-ribose.^10^ While MH and substitution with LiOBn worked well for the C_1_-building block 5, and two more homologs, further homologation proved to be problematic. Attempts to react 6 with LiCHBr_2_ led to intractable mixtures and the use of LiCHCl_2_ only allowed for the indirect detection of product traces. The route was thus concluded by homologation with LiCH_2_Cl, which does not allow for installation of another stereocenter. For detailed discussion of this surprising limitation, an explanatory hypothesis and supporting evidence see the ESI.‡ Importantly this restricted Matteson's synthesis to ribose (an aldopentose),^15^ while most biologically relevant monosaccharides are hexoses. Thus, a C_1_-based synthesis of aldohexoses that allows for (i) installing individual protecting groups, (ii) choosing the configuration at each stereocenter and (iii) potentially introducing isotopic labels at every individual atom, remained an open challenge.^11^ We achieved this by preparing orthogonally protected versions of prototypical sugar alcohols from CH_2_X_2_ building blocks through three MHs, vinylation and bishydroxylation (Scheme 1C). By strategically combining different homologation and vinylation strategies, both *erythro* and *threo* isomers were realized. Conversion of the sugar alcohols into aldohexoses can be achieved by oxidation of either terminal hydroxyl functionality. By combining this with a short synthesis of the vinylation agent from CH_2_X_2_ building blocks we paved the way for the late-stage introduction of isotopic labels.

To start the discussion with the stereochemically most basic example, the synthesis of allitol 12a is depicted in Scheme 2. It begins with the CH_2_Br_2_ derived C_1_-building block 7a. MH with LiCHBr_2_ and substitution with LiOPMB delivered the C_2_-building block 8a. Two consecutive MHs, which are followed by substitution with an alkoxide, produce a masked *erythro* glycol motif. Thus, a second MH and substitution with LiOBn delivered 9a, with an *erythro* relationship between C^2^ and C^3^ (as IUPAC priorities change during the route, carbon atoms are numbered according to their introduction in this article). Another homologation and substitution with LiOBn yielded 10a in 47% yield after two steps.^18^ While other alkoxide based protecting groups could have been used here, a second benzyl group was chosen, to allow for confirmation of the relative configuration by direct comparison (see ESI‡). Vinylation of 10a was achieved by Zweifel-olefination.^17^ Although this reaction had not been described for the sterically hindered and thermodynamically stable pinanediol boronic esters, it proceeded reasonably well after some optimization (see ESI‡) yielding 11a. The product contained some unidentified contaminations, which were removed after the next step, in which Sharpless bishydroxylation delivered the desired allitol-derivative 12a (32% yield, calculated over both steps). Several silylethers at C^1^ were tested but neither the use of TBS- (*tert*-butyldimethylsilyl) nor TBDPS-groups (*tert*-butyldiphenyl-sily) on the first hydroxyl group allowed for introduction of a forth carbon atom (13→14). This was quite surprising as a third Matteson-sequence had worked well for the benzyl-derivative 6. Indeed, homologation of 13 to bromides 15 proceeded with reasonable efficiency, but subsequent substitution with LiOBn led to decomposition. This was attributed to competing nucleophilic attack of benzoxide on the silyl ether. We suspected that the latter was activated by an intramolecular O–B interaction, which simultaneously deactivates the boron-atom as an electrophile. By using the TIPS-group (triisopropylsilyl) this side reaction was avoided, through better shielding of the silicon atom (further discussion in ESI‡).^19^

**Scheme 2:**
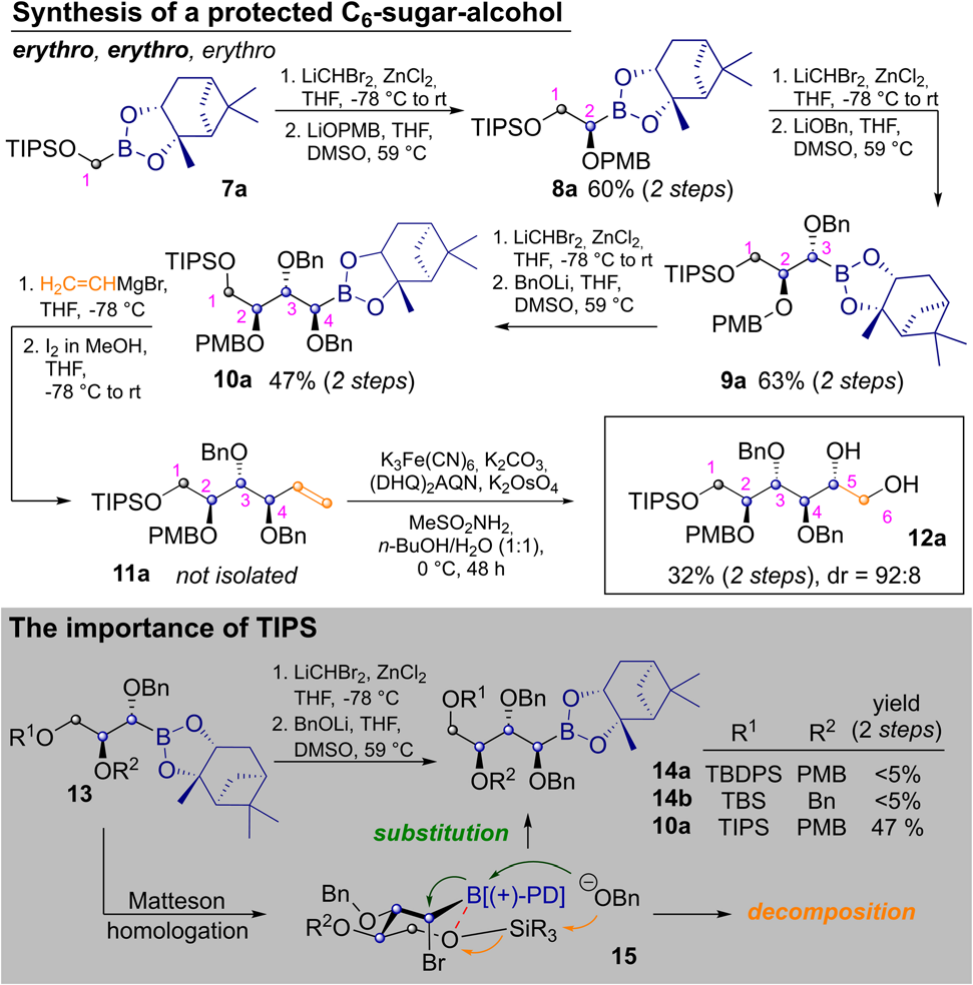
Synthesis of protected allitol and associated challenges.

In order to extend the route to other diastereomers it was necessary to modify the synthesis, so that *threo*-glycols could be obtained. This was a particular challenge for the three stereocenters generated by Matteson homologation as the exchange of the pinanediol director is quite cumbersome.^20^ Thus the route was modified as shown in Scheme 3.

**Scheme 3:**
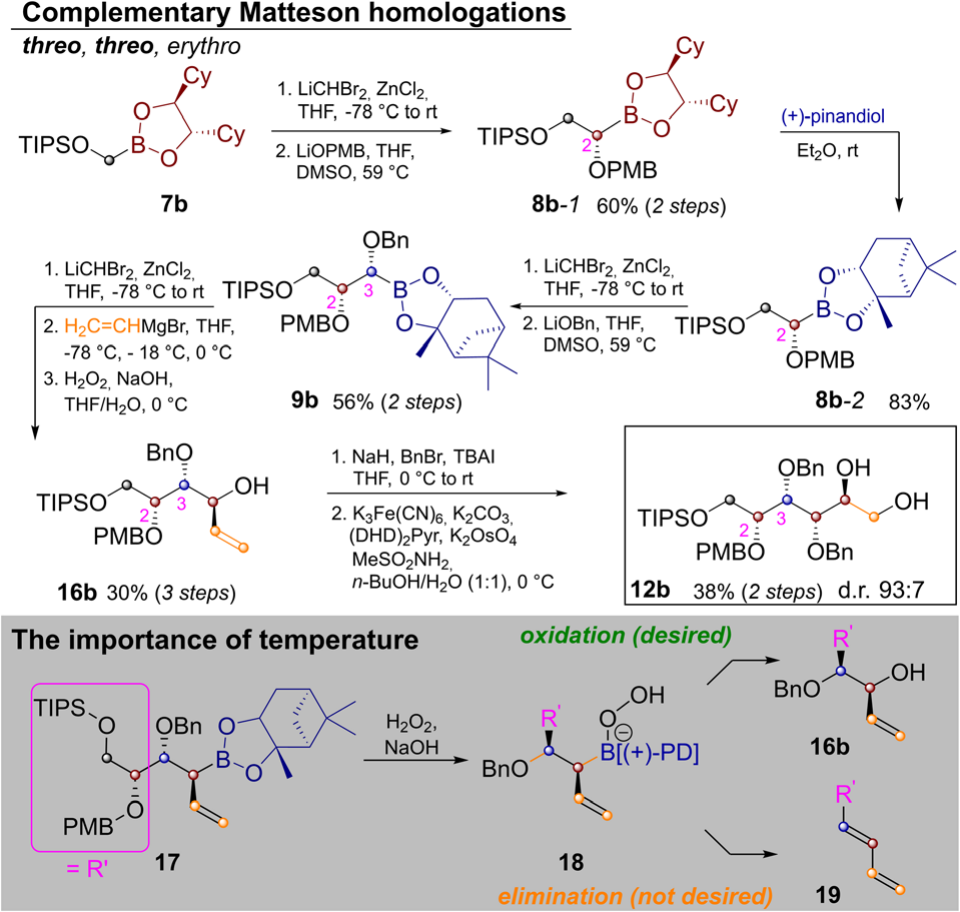
Synthesis of protected l-glucitol and associated challenges.

In order to establish a *threo*-relationship between C^2^ and C^3^ the synthesis starts with *S*,*S*-dicyclohexylethylenediol (*S*,*S*-DICHED)^21^ boronic ester 7b. Homologation and substitution analogously to Scheme 2 delivered 8b-1. The greater thermodynamic stability of pinanediol boronic esters^22^ allowed for transesterification to 8b-2 with (+)-pinanediol in Et_2_O,^15^ as well as recovery of the precious *S*,*S*-DICHED auxiliary. However, once this card had been played and the more stable pinanediol boronate was formed, a different strategy had to be applied. In order to establish a *threo* relationship between C^3^ and C^4^, the third Matteson homologation was followed by substitution with vinylmagnesium bromide^23^ and oxidation to 16b. In contrast to Scheme 2 the C–B bond is now converted into the new C–O bond, while the vinyl group is introduced under inversion. Benzyl protection and Sharpless-bishydroxylation delivered the desired glucitol 12b. Surprisingly the seemingly simple combination of a Matteson sequence and a boronate oxidation in 9b→16b proved to be quite challenging. Competing elimination reactions, such as the one from 18 to the conjugated diene 19 had to be suppressed by a strict temperature regime. Therefore, 9b was homologated as usual. Substitution with vinylmagnesium bromide to 17 required addition of the Grignard reagent at −78 °C, storage in a freezer over night at −18 °C and stirring for 4 h at 0 °C. Instead of isolating 17, H_2_O_2_ was added at 0 °C and the reaction was kept at this temperature for 4 h, before the reaction was quenched by the addition of Na_2_S_2_O_3_ (see ESI‡ for further discussion).

By appropriately combining the two complementary strategies shown in Schemes 2 and 3 all but two aldohexoses should be available. However, both idose and galactose are still elusive at this point, as they have *threo* relationships between C^2^ and C^3^ as well as C^4^ and C^5^ and so far we have only shown how to establish an *erythro* relationship by Sharpless bishydroxylation. Thus Scheme 4 depicts the synthesis of mannitol 12c and its inversion to epoxide 20, which has the desired *threo* relationship between C^4^ and C^5^. This required homologation of 9a, substitution with vinylmagnesium bromide and oxidation to 16c. Benzylation yielded 11c and Sharpless-bishydroxylation delivered mannitol 12c. Acylation of the primary hydroxide, mesylation of the remaining alcohol and epoxide closure by basic estercleavage (with concomitant substitution of the mesylate) produced 20.^27^

**Scheme 4:**
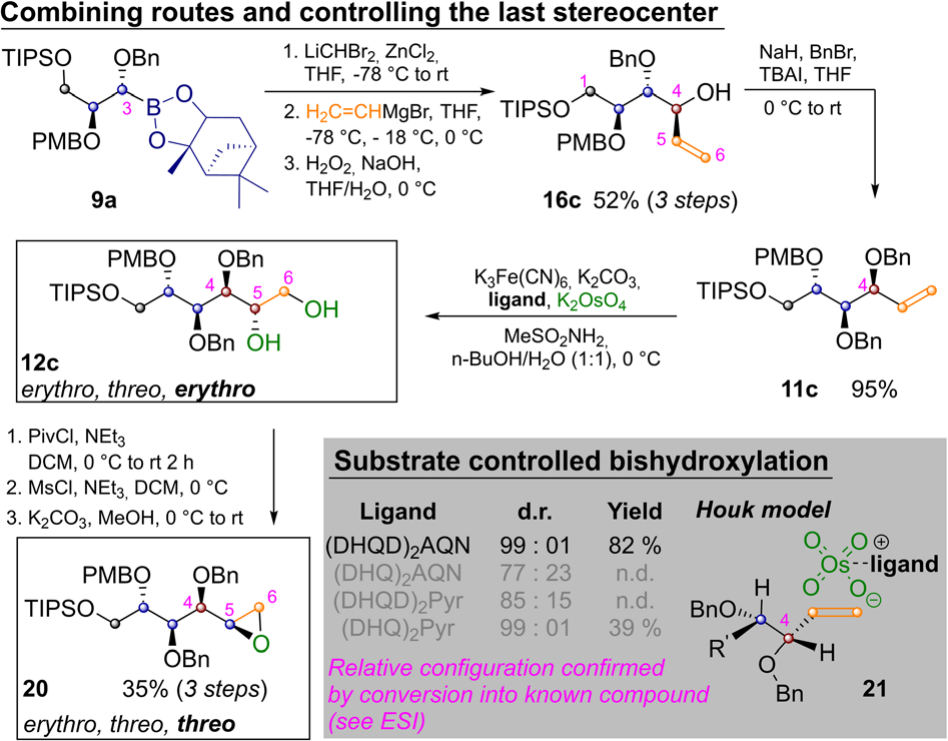
Synthesis of protected l-mannitol 12c and inversion to epoxide 20.

l-Mannitol 12c was chosen as a last example for several reasons: firstly to demonstrate the ease with which the routes in Schemes 2 and 3 can be combined. Secondly chemically labelled mannose-derivatives are notoriously hard to track in biological systems, which makes isotope labelled derivatives of great value.^24^ Finally, several mannitol derivatives are commercially available. This allowed us to confirm the relative configuration of the Sharpless bishydroxylation products by direct comparison (see ESI‡).^25^ In these reactions very strong substrate control is exhibited by the allylic stereocenter favoring the *erythro*-product. This is in accordance with predictions of the Houk-model 21 (Scheme 4, see ESI‡ for complete table and discussion).^26^ While an oxidative method that allows for choosing the desired configuration at this last stereocenter directly would still be preferable, positioning of the diol nevertheless allows for straight forward inversion as demonstrated by conversion into 20.^27^ Finally two methods for converting the prepared sugar-alcohols into carbohydrates were probed on 12c (Scheme 5).^28^

**Scheme 5:**
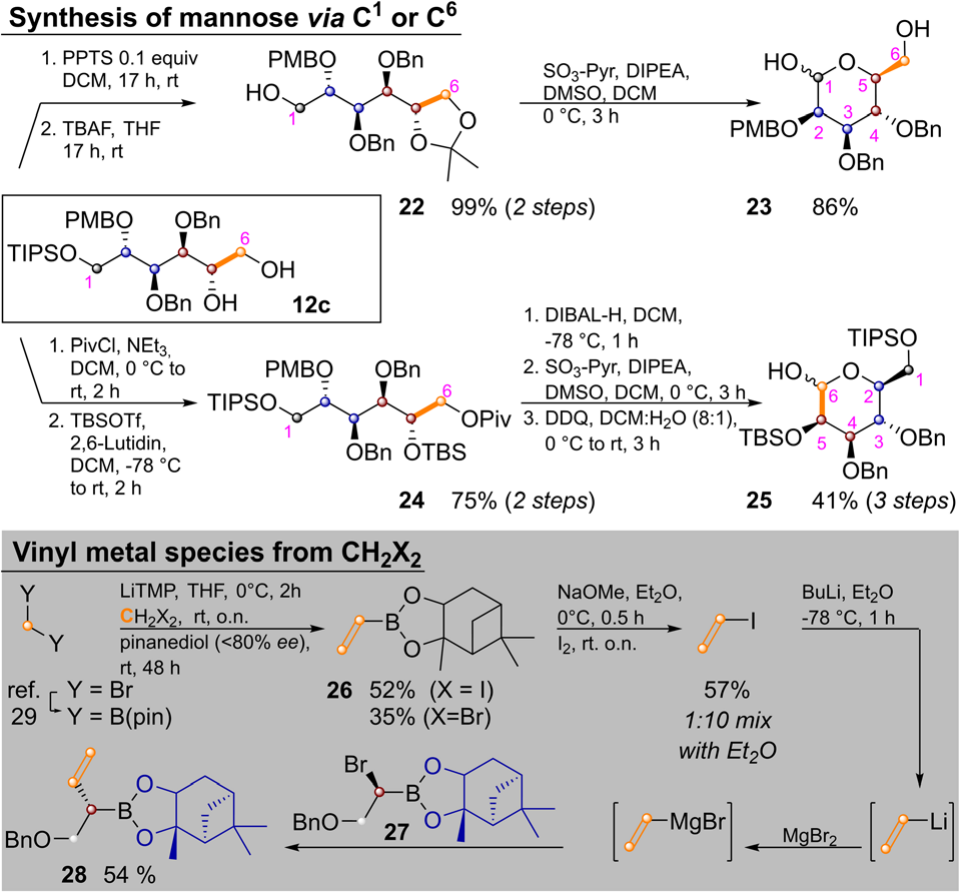
Synthesis of l-mannose and vinyl iodide from C_1_-units.

Temporary ketal protection of diol 12c and selective cleavage of the TIPS group delivered 22 in 99% yield. Parikh–Doering oxidation of the free hydroxide at C^1^ proceeded under ketal cleavage leading to l-mannose derivative 23 in 86% yield. In it only the easily distinguishable primary and anomeric hydroxyl groups are unprotected. The three secondary hydroxyl groups are masked by orthogonal protecting groups, the placement of which could be easily varied. In some cases it might be advantageous to convert sugar alcohols of type 12 into an aldohexose *via* the other terminus (*i.e.*, C^6^). This was achieved by orthogonal protection of 12c, yielding 24 in 75% yield over two steps. Pivaloyl deprotection, Parikh–Doering oxidation and PMB cleavage led to 25 in 41% yield over three steps. These two options could allow for late-stage introduction of isotopic labels, as three of six carbon atoms are introduced in the final homologation/vinylation sequence. To enable this, a route to vinyl metal species from C_1_-building blocks (*i.e.*, CH_2_X_2_) was developed: bis(pinacolato)borylmethane^29^ was lithiated and reacted with either dibromo- or diodomethane as described by Morken and coworkers,^30^ yielding vinyl pinacol boronic ester. This product is highly volatile, but transesterification with pinanediol to 26 enabled purification by flash chromatography. Reaction of 26 with NaOMe and I_2_ yielded vinyl iodide. Vinyllithium (the preferred reagent for Zweifel reactions)^17^ was obtained by iodo–lithium exchange. Efficient reaction with α-bromoboronate 27, required transmetallation with MgBr_2_.

## Conclusions

All in all we have developed a highly modular route that opens up a vast field of opportunities for the synthesis of differentially protected sugar alcohols and carbohydrates. By combining the different approaches depicted in Schemes 2–4 a wide variety of C_6_-sugar alcohols and by extension all natural and unnatural aldohexoses become available. Two consecutive MHs, followed by substitution with an alkoxide lead to *erythro*-C_3_ building block 9a. To introduce a C^2^–C^3^*threo*-relationship the chiral director can be changed from DICHED to pinanediol. The transesterification proceeds readily and allows for recovery of the valuable DICHED auxiliary. Both enantiomers of DICHED and pinanediol are available, so that all stereoisomers of C_3_-building blocks of type 9 are accessible. To gain control over the relationship between C^3^ and C^4^ a strategic crossroad was incorporated in the next homologation. A vinyl group was introduced either by Zweifel olefination (*erythro*) or Matteson substitution (*threo*). Thereby C_3_-building blocks of type 9 can be converted into vinyl tetrol of type 11, again with the potential for making all stereoisomers. For installing the final glycol moiety Sharpless bishydroxylation was employed. Unfortunately overwhelming substrate control only allowed for the direct synthesis of sugar alcohols of type 12 with a C^4^–C^5^-*erythro* configuration. In order to obtain a C^4^–C^5^-*threo* configuration at this position conversion into epoxide 20 was necessary. Some first attempts at epoxide opening to a diol of type 12 (with a C^4^–C^5^-*threo* configuration) were plagued by side reactions (see ESI‡). Fortunately this only affects the synthesis of monosaccharides with both C^2^–C^3^-*threo* and C^4^–C^5^-*threo* configurations (*i.e.*, galactose and idose). For these cases the corresponding epoxides might be better converted into hexoses along the lines of Shapless's carbohydrate synthesis.^31^ In all other cases conversion into the desired aldohexoses can be achieved by appropriate cyclisation *via* C^1^ or C^6^ due to the orthogonal silyloxy group at C^1^ (Scheme 5). Another advantage of these two cyclisation options arises as half of the carbon scaffold is introduced in the last homologation/vinylation sequence. The required vinyl metal species can be prepared from two (CH_2_X_2_ derived) C_1_ building blocks (Scheme 5). By choosing the appropriate cyclisation route an isotopic label could thus be placed at every position in the aldohexose scaffold. Thus this C_1_ based *de novo* approach to aldohexoses is uniquely suited for the synthesis of labelled aldohexoses, which we plan to pursue in the near future.

## Data availability

All experimental and characterization data, as well as pictures of NMR spectra are available in the ESI.‡

## Author contributions

S. K. developed the synthesis of aldohexoses; G. A. developed the synthesis of vinyl metal species from C_1_-units; C. H. conceived the project and wrote the manuscript.

## Conflicts of interest

There are no conflicts to declare.

## Supplementary Material

SC-014-D3SC03778A-s001
