# Supplementary material for: A unified strategy for the synthesis of aldohexoses by boronate assisted assembly of CH2X2 derived C1-building blocks
Source: Chem Sci. 2023 Sep 4;14(36):9838–42. doi: 10.1039/d3sc03778a (PMC10510816; doi:10.1039/d3sc03778a)
Supplement: SC-014-D3SC03778A-s001 [file SC-014-D3SC03778A-s001.pdf]

Electronic Supplementary Material (ESI) for Chemical Science.
This journal is © The Royal Society of Chemistry 2023

Content

Navigation Table 3

General Information 4

Nomenclature 4

Reactions 4

Chromatography 4

Analytics 4

Experimental Procedures 5

Synthesis of C_1-Buildingblocks 7a and 7b 5

2-(Bromomethyl)-4,4,5,5-tetramethyl-1,3,2-dioxaborolane (S1) 5

2-((Benzyloxy)methyl)-4,4,5,5-tetramethyl-1,3,2-dioxaborolane (S2) 6

(*S*)-Pinanediol [(Benzyloxy)methyl]boronate (S3a) 6

(*S*)-Pinanediol (Hydroxymethyl)boronate (S4a) 7

(*S*)-Pinanediol [((triisopropylsilyl))oxy)methyl]boronate (7a) 7

(4S,5S)-4,5-Dicyclohexyl-2-(benzyloxy)methyl-1,3,2-dioxaborolane (S3b) 8

(4S,5S)-4,5-Dicyclohexyl-2-hydroxymethyl-1,3,2-dioxaborolane (S4b) 8

(((4S,5S)-4,5-Dicyclohexyl-1,3,2-dioxaborolan-2-yl)methoxy)triisopropylsilane (7b) 8

Route to Allitol 12a 9

Allitol Series – C_2-Building Block (8a) 9

Allitol Series – C_3-Building Block (9a) 10

Allitol Series – C_4-Building Block (10a) 11

Allitol Seriec – Vinylation and subsequent Bishydroxylation to Allitol 12a 12

Route to Glucitol 12b 13

Glucitol Series – DICHED-C_2 Buildingblock (8b-*1*) 13

Glucitol Series – Pinanediol-C_2 Buildingblock (8b-*2*) 14

Glucitol Series –C_3 Buildingblock (9b) 15

Glucitol Series –Vinylation (16b) 16

Glucitol Series – Benzyl Protection (11b) 17

Glucitol Series – Bishydroxylation (12b) 17

Route to Mannitol 12c 18

Mannitol Series - Vinylation (16c) 18

Mannitol Series – Benzyl protection (11c) 19

Mannitol Series – Bishydroxylation (12c) 20

Inverting the Stereocenter Formed by Bishydroxylation 21
Regioselective Acetate Protection (S5) 21
Regioselective Pivaloyl Protection (S6) 21
Inversion by Epoxidation (20) 22
Attempted Hydrolysis of Epoxide 20 (S7)* 23
Conversion into Aldohexoses 24
Cyclisation *via* C^1 - Acetal Protection of 12c (S8) 24
Cyclisation *via* C^1 - Desilylation (22) 25
Cyclisation *via* C^1 - Parikh-Doering Oxidation to Aldohexose 23 25
Cyclisation *via* C^6 –Protection of the Secondary Hydroxyl Group in S6 (24) 26
Cyclisation *via* C^6 –Liberation of the Primary Hydroxyl Group (S9) 27
Cyclisation *via* C^6 –Synthesis of Aldohexose 25 27
Vinylmetalspecies from CH_2X_2 28
Pinanediol (vinyl)boronate (26) 28
Vinyl iodide 29
Optimization, Mechanistic Discussion and Stereochemical Assignment 32
Matteson-Homologation –Protecting Groups and the 4th Homoloation 32
Zweifel-Olefination with Pinanediol Boronic Esters 34
Matteson Reaction with Vinylmagnesiumbromide and Oxidation 35
Optimization and Stereochemical Assignment of the Bishydroxylation 36
(1R,2R)-1,2-bis(benzyloxy)-1,2-bis((R)-2,2-dimethyl-1,3-dioxolan-4-yl)ethane (S15) 38
Attempts to invert the configuration at C^5 by Mitsunobu-Reaction 42
Attempts to invert the configuration at C^4 by Mitsunobu-Reaction 43
Reaction of Vinyliodide with α-Halo Borononic Esters 45
Selected NMR Spectra 46

How to use this table

1 min. video

Navigation Table

Aldohexose	C_1-Building-block	1. Matteson-Homologation	2. Matteson-Homologation	3. MH and Vinylation	Sugar-Alcohol
**Cyclization *via* C^6**					
L-Allose<br>*for D-Allose use (-)-pinanediol*	7a	8a	9a	11a	12a
D-Gulose<br>*for L-Gulose use R,R-DICHED and (-)-pinanediol*	7b	8b-2	9b	11b	12b
L-Mannose<br>*for D-Mannose use (-)-pinanediol*	7a	8a	9a	11c	12c
D-Talose<br>*for L-Talose use R,R-DICHED and (-)-pinanediol*	7b	8b-2	9b	11d	12d
**Cyclization *via* C^6**					
L-Glucose<br>*for D-Glucose use (-)-pinanediol*	7a	8a	9a	11c	20
D-Galactose<br>*for L-Galactose use R,R-DICHED and (-)-pinanediol*	7b	8b-2	9b	11d	20b
L-Altrose<br>*for D-Altrose use (-)-pinanediol*	7a	8a	9a	11a	20a
D-Idose<br>*for D-Idose use R,R-DICHED and (-)-pinanediol*	7b	8b-2	9b	11b	20b
**Cyclization *via* C^1**					
D-Allose<br>*for L-Allose use (-)-pinanediol*	7a	8a	9a	11a	12a
L-Glucose<br>*for D-Glucose use R,R-DICHED and (-)-pinanediol*	7b	8b-2	9b	11b	12b
L-Mannose<br>*for D-Mannose use (-)-pinanediol*	7a	8a	9a	11c	12c
D-Altrose<br>*for L-Altrose use R,R-DICHED and (-)-pinanediol*	7b	8b-2	9b	11d	12d

General Information

Nomenclature

IUPAC nomenclature was used only for starting materials and in the headlines of procedures for simple compounds. In these cases, atoms were numbered according to the IUPAC rules. Complex compounds were given descriptive names and are defined by a reference to a nearby depiction. In the latter cases, carbon atoms were numbered as in the main article, i.e. according to the order in which they were introduced during iterative synthesis. To differentiate between these systems, numbers referring to the order of introduction are superscript to the carbon atom (e.g. C^4 refers to the forth carbon atom introduced in the synthesis). When building blocks are classified according to their size the numbers are subscript to the carbon atom (e.g. a C_3-building block has a central carbon chain of three atoms).

Reactions

Reactions with oxygen and moisture sensitive materials were carried out in dried glassware under argon. Commercially available reagents were stored as indicated and used without further purification, unless otherwise noted. BuLi, Vinylmagnesium bromide and $ZnCl_2$ were purchased as solutions and stored under argon at +4° C. THF and Et_2O were always freshly distilled under argon from sodium/benzophenone. BnOH, CH_2Cl_2, DMSO, tetramethylpiperidine (TMP) and diisopropylamine (DIPA) were distilled from CaH_2 and stored under argon over molecular sieve as appropriate.

Chromatography

Flash column chromatography was performed with Machery-Nagel SiO_2; 60 M, 0.04 - 0.043 mm. Solvents for chromatography, unless purchased as *pro analysi* (p. a.) grade, were distilled before use with a rotary evaporator. Reversed phase medium performance liquid chromatography (MPLC) was conducted with the following setup: Armen Instrument Spot Liquid Chromatography Flash system (detection wavelength: 263 nm), YMC GEL ODS-AQ 12 nm, S-50 µm in a Kronlab Glass column with a 10 mm diameter and a length of 500 mm. Water for MPLC was obtained from a TKA MicroPure ultrapure water system.

Analytics

Polygram® SOL G/UV254 TLC plates (silica gel, 0.2 mm x 40 mm x 80 mm) were used for thin layer chromatography (TLC). A UV lamp was used to visualize spots at either 254 nm or 365 nm wavelength, after that a staining solution of $KMnO_4$ was employed. ^1H and ^{13}C NMR spectra were recorded with Bruker DMX 300, AV Neo 400 NMR and Bruker DRX 500 spectrometers at room temperature. IR spectra were measured with a Jasco FT/IR-430 spectrometer bearing an ATR attachment. Low and high resolution ESI mass spectra were recorded with Bruker amaZon SL and Bruker maXis 4G spectrometers, respectively. Chiral normal phase analytical high performance liquid chromatography (HPLC) was conducted with the following setup: Erma Degasser ERC-3512, Merck Hitachi Intelligent Pump L-6200A, Chiralcel OD-H column (0.46 x 25 cm), Knauer Smartline UV-Detector 2600 (detection wavelength 225 nm). Reversed phase analytical high performance liquid chromatography (RP-HPLC) was conducted with the following setup: Dionex HPLC system: P680 pump, ASI-100 automated sample injector, UVD-340U UV detector (detection wavelength: 263 nm), UltiMate 3000 Column Compartment; YMC-Pack ODS-Acolumn (3.0 x 150 mm, 5 µm, 12 nm; type: AA12S05-1503QT).

Experimental Procedures

Synthesis of C1-Buildingblocks 7a and 7b

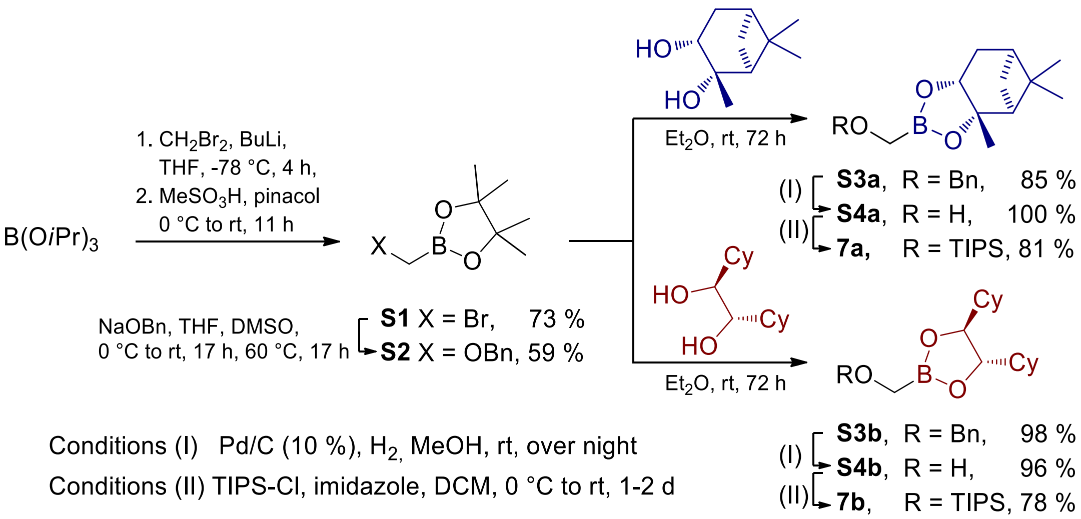

Scheme I: Overview of the synthetic route to the C_1-buildingblocks 7a and 7b.[1]

2-(Bromomethyl)-4,4,5,5-tetramethyl-1,3,2-dioxaborolane (S1)

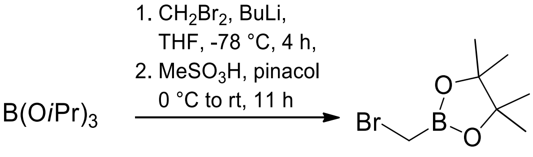

S1 73 %

Under an atmosphere of argon *n*-BuLi (1.6 M in hexane, 160 mL, 256 mmol, 1.0 eq.) was added dropwise to a solution of triisopropyl borate (65 mL, 282 mmol, 1.1 eq.) and CH_2Br_2 (21.6 mL, 308 mmol, 1.2 eq.) in THF (400 mL) at -78 °C over 2 h. The mixture was stirred at rt for 2 h. After that, it was cooled to 0 °C and methanesulfonic acid (16.6 mL, 256 mmol, 1.0 eq.) was added dropwise. Stirring was continued for 15 min at rt and subsequently, pinacol (24.2 g, 256 mmol, 1.0 eq.) was added in portions at 0 °C and stirring was continued for 11 h at rt. The solvent was removed *in vacuo* to give a yellowish white suspension. The suspension was diluted with CH_2Cl_2 (50 mL), filtered over Celite® and the white solid was washed with CH_2Cl_2 (400 mL). The combined organic solvents were removed *in vacuo*. The residue was purified by distillation (head temperature at p = 5.0 mbar: 62 °C; oil bath temperature: 95 °C). Bromide **S1** was obtained as a pungent, lacrimatory colourless oil (45.5 g, 206 mmol, 73%).

^{1}H NMR (300 MHz, $CDCl_3$): δ (ppm) = 2.51 (s, 2H), 1.22 (s, 12H).

^{13}C NMR (75 MHz, $CDCl_3$): δ (ppm) = 84.3, 24.4, *carbon attached to boron not observed*.

Spectroscopic data were consistent with those reported in the literature.[2]

[1] R. P. Singh, D. S. Matteson, *J. Org. Chem.* **2000**, *65*, 6650–6653.

[2] A. Pulis, P. Alexander, V. K. Aggarwal, *J. Am. Chem. Soc.*, **2012**, *134*, 7570–7574.

2-((Benzyloxy)methyl)-4,4,5,5-tetramethyl-1,3,2-dioxaborolane (S2)

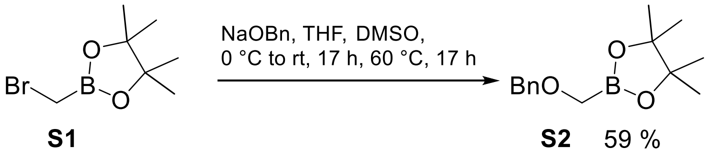

Under an atmosphere of argon NaH (60% dispersion in mineral oil, 13.1 g, 328 mmol, 1.2 eq.) was added slowly at 0 °C to a solution of BnOH (34.1 mL, 328 mmol, 1.2 eq.) in THF and the mixture was stirred for 17 h at rt. Then 2-(bromomethyl)-4,4,5,5-tetramethyl-1,3,2-dioxaborolane **S1** (60.3 g, 273 mmol, 1.0 eq.) was added dropwise at 0 °C and DMSO (28 mL, 355 mmol, 1.3 eq.) was added. The mixture was stirred at 60 °C for 17 h. It was cooled to 0 °C and quenched by addition of HCl (aq., 1M, 300 mL). The aqueous phase was extracted with CH_2Cl_2 (3 x 200 mL). The combined organic layers were dried with $MgSO_4$ and concentrated *in vacuo*. The residue was purified by distillation (b.p 75 °C at p = 0.013 mbar). The product **S2** was obtained as a colourless oil (40 g, 161 mmol, 59%).

^{1}H NMR (300 MHz, $CDCl_3$): δ (ppm) = 7.34 – 7.22 (m, 5H), 4.49 (s, 2H), 3.26 (s, 2H), 1.25 (s, 12H).

^{13}C NMR (75 MHz, $CDCl_3$): δ (ppm) = 138.0, 128.1, 128.0, 127.4, 83.7, 75.6, 24.6, *carbon attached to boron not observed*.

Spectroscopic data were consistent with those reported in the literature.[3]

(*S*)-Pinanediol [(Benzyloxy)methyl]boronate (S3a)

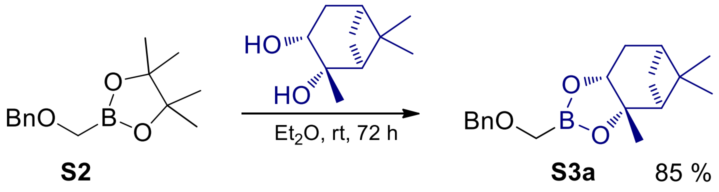

Under an atmosphere of argon pinacol boronic ester **S2** (3.0 g, 12 mmol, 1.0 eq.) was dissolved in Et_2O (50 mL) and (+)-Pinanediol (2.08 g, 12.2 mmol, 1.02 eq.) was added. The mixture was stirred for 72 h at rt. The solvent was removed *in vacuo*. The residue was purified by flash chromatography (SiO_2, CyHex/EtOAc, 10:1, $KMnO_4$), yielding **S3a** (3.08 g, 10.2 mmol, 85%) as a colourless oil.

R_f (EA/CH 1:5) = 0.64.

^{1}H NMR (600 MHz, $CDCl_3$): δ (ppm) = 7.38 – 7.26 (m, 5H), 4.55 (d, *J* = 12.3 Hz, 1H), 4.51 (d, *J* = 12.4 Hz, 1H), 4.33 (dd, *J* = 8.7, 1.8 Hz, 1H), 3.36 (d, *J* = 16.1 Hz, 1H),* 3.34 (d, *J* = 16.1 Hz, 1H),* 2.37 – 2.30 (m, 1H), 2.26 – 2.19 (m, 1H), 2.08 (dd, *J* = 6.0, 4.8 Hz, 1H), 1.94 – 1.88 (m, 2H), 1.41 (s, 3H), 1.29 (s, 3H), 1.16 (d, *J* = 10.9 Hz, 1H), 0.84 (s, 3H).

^{13}C NMR (151 MHz, $CDCl_3$): δ (ppm) = 138.4, 128.4, 128.3, 127.7, 86.5, 78.4, 76.0, 51.3, 39.6, 38.2, 35.3, 28.7, 27.2, 26.6, 24.1, *carbon attached to boron not observed*.

IR (film) ν_{max} (cm^{-1}): 2916, 2868, 2361, 1605, 1495, 1452, 1408, 1377, 1342, 1284, 1243, 1209, 1093, 1076, 1056, 1029, 989, 937, 923, 901, 846, 820, 734, 697.

ESI-HRMS: calc. for $[C_{18}H_{25}BO_3+Na]^+$ 323.1794, found 323.1802.

***Comment:** These diastereotopic CH_2-protons show a strong roof effect and thus appear as singlets in low resolution spectra.[4] Coupling Constants were measured from peaks 1 to 2 and 3 to 4.

---

[3] C. Shu, A. Noble and V. K. Aggarwal, *Angew. Chemie Int. Ed.*, 2019, **58**, 3870–3874.

[4] D. S. Matteson and M. L. Peterson, *J. Org. Chem.*, 1987, **52**, 5116–5121.

(S)-Pinanediol (Hydroxymethyl)boronate (S4a)

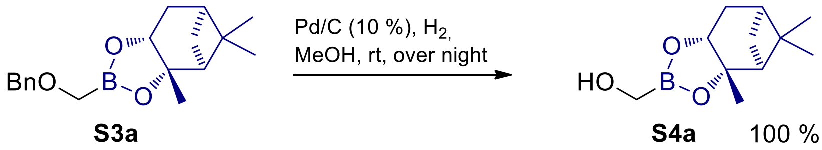

A solution of **S3a** (0.20 g, 0.67 mmol, 1.0 eq.) in MeOH* (10 mL) was hydrogenated over 10% Pd/C (0.08 g) at 1 atm of Hydrogen at 20-25 °C for 17 h. The solution was filtered through Celite®, washed with MeOH (50 mL) and concentrated *in vacuo* yielding **S4a** (0.16 g, 0.67 mmol, quant.) as a white solid.

R$_f$ (EA/CH 1:5) = 0.08.

^{1}H NMR (600 MHz, $CDCl_3$): δ (ppm) = 4.35 (dd, *J* = 8.7, 1.9 Hz, 1H), 3.61 (d, *J* = 19.0 Hz, 1H), 3.58 (d, *J* = 19.2 Hz, 1H), 2.39 – 2.30 (m, 1H), 2.28 – 2.22 (m, 1H), 2.08-2.05 (m, 1H), 1.96 – 1.91 (m, 1H), 1.91 – 1.87 (m, 1H), 1.42 (s, 3H), 1.29 (s, 3H), 1.16 (d, *J* = 11.0 Hz, 1H), 0.85 (s, 3H).

^{13}C NMR (151 MHz, $CDCl_3$): δ (ppm) = 86.9, 78.6, 51.4, 39.6, 38.3, 35.4, 28.7, 27.2, 26.6, 24.1, *carbon attached to boron not observed*.

IR (film) ν_{max} (cm^{-1}): 3422, 2915, 2870, 1723, 1516, 1474, 1414, 1375, 1341, 1283, 1238, 1207, 1155, 1140, 1123, 1076, 1055, 1028, 990, 922, 901, 868, 839, 822, 754, 687, 667, 654, 625.

ESI-HRMS: calc. for $[C_{11}H_{19}BO_3+Na]^+$ 233.1325, found 233.1317.

***Comment:** Hydrogenation in EtOAc delivered **S4a** in 67 % yield, but also 33% of pinandiol borane.

(S)-Pinanediol [((triisopropylsilyl))oxy)methyl]boronate (7a)

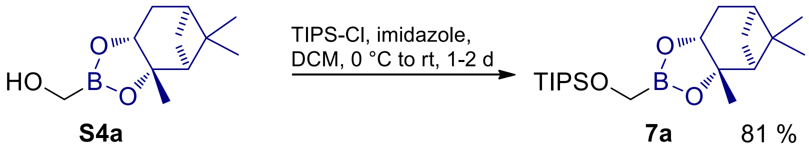

Under an atmosphere of argon imidazole (4.30 g, 63.4 mmol, 1.2 eq.) was added to a solution of **S4a** (11.1 g, 52.8 mmol, 1.0 eq.) in CH_2Cl_2 (50 mL). Triisopropylsilyl chloride (11.2 mL, 52.8 mmol, 1.0 eq.) was added dropwise at 0 °C to the reaction mixture and stirring was continued for 48 h. The reaction was quenched by addition of NH_4Cl (aq. sat., 20 mL) and the aqueous phase was extracted with CH_2Cl_2 (3 x 50 mL). The organic phase was dried over $MgSO_4$ and the solvent was removed *in vacuo*. The residue was purified by flash chromatography (SiO_2, CyHex/EtOAc, 20:1, $KMnO_4$), yielding **7a** (15.7 g, 42.9 mmol, 81%) as a colourless oil.

R$_f$ (EA/CH 1:20) = 0.40.

^{1}H NMR (600 MHz, $CDCl_3$): δ (ppm) = 4.33 (dd, *J* = 8.8, 1.9 Hz, 1H), 3.65 (d, *J* = 16.8 Hz, 1H), 3.63 (d, *J* = 16.4 Hz, 1H) 2.37 – 2.29 (m, 1H), 2.25 – 2.18 (m, 1H), 2.06 – 2.03 (m, 1H), 1.94 – 1.86 (m, 2H), 1.39 (s, 3H), 1.28 (s, 3H), 1.16 (d, J = 10.9 Hz, 1H), 1.15 – 1.04 (m, 21H), 0.84 (s, 3H).

^{13}C NMR (151 MHz, $CDCl_3$): δ (ppm) = 86.3, 78.2, 51.4, 39.6, 38.3, 35.5, 28.7, 27.2, 26.6, 24.1, 18.2, 12.1, *carbon attached to boron not observed*.

IR (film) ν_{max} (cm^{-1}): 2920, 2866, 2374, 2320, 1456, 1377, 1340, 1282, 1241, 1209, 1157, 1142, 1122, 1078, 1055, 1030, 989, 951, 937, 922, 881, 841, 825, 791, 754, 681, 656, 607.

ESI-HRMS: calc. for $[C_{20}H_{39}BO_3Si+Na]^+$ 389.2659, found 389.2653.

(4S,5S)-4,5-Dicyclohexyl-2-(benzyloxy)methyl-1,3,2-dioxaborolane (S3b)

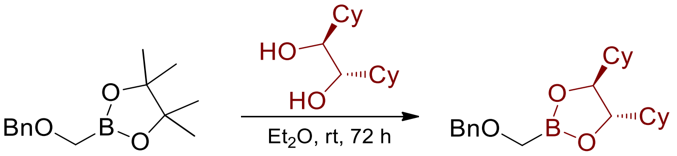

S2 **S3b**, 98 %

Under an atmosphere of argon pinacol boronic ester **S2** (1.60 g, 6.45 mmol, 1.0 eq.) was dissolved in Et_2O (20 mL) and (*S,S*)-DICHED (1.46 g, 6.45 mmol, 1.0 eq.) was added. The mixture was stirred for 72 h. The solvent was removed *in vacuo* and the residue was purified by flash chromatography (SiO_2, CyHex/EtOAc, 9:1, $KMnO_4$), yielding boronic ester **S3b** (2.27 g, 6.37 mmol, 98%) as a colourless oil.

^{1}H NMR (400 MHz, $CDCl_3$): δ (ppm) = 7.40 – 7.22 (m, 5H), 4.54 (d, J = 12.2 Hz, 1H), 4.51 (d, J = 12.2 Hz, 1H), 3.96 – 3.88 (m, 2H), 3.37 (d, J = 16.1 Hz, 1H), 3.33 (d, J = 16.1 Hz, 1H), 1.81 – 1.52 (m, 10 H), 1.43 – 0.89 (m, 12H).

^{13}C NMR (101 MHz, $CDCl_3$): δ (ppm) = 138. 5, 128.4, 128.3, 127.7, 84.0, 75.8, 43.0, 28.4, 27.5, 26.6, 26.1, 26.0.

Spectroscopic data were consistent the literature where CH_2-groups were reported as one signal.[5]

(4S,5S)-4,5-Dicyclohexyl-2-hydroxymethyl-1,3,2-dioxaborolane (S4b)

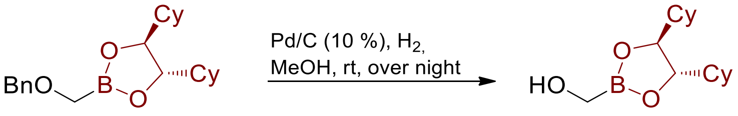

S3b **S4b**, 96 %

A solution of ester **S3b** (2.27 g, 6.4 mmol, 1.0 eq.) in MeOH (20 mL) was hydrogenated over 10% Pd/C (50 mg) at 1 atm at 20-25 °C for 24 h. Filtration over celite and concentration under reduced pressure yielded the product **S4b** (1.70 g, 6.4 mmol, 96%) as a white solid.

^{1}H NMR (400 MHz, $CDCl_3$): δ (ppm) = 3.96 – 3.90 (m, 2H), 3.58 (s, 2H), 1.71 – 0.90 (m, 23H).

^{13}C NMR (101 MHz, $CDCl_3$): δ (ppm) = 84.3, 43.0, 28.5, 27.4, 26.5, 26.1, 25.9.

Spectroscopic data were consistent with those reported in the literature.[5]

(((4S,5S)-4,5-Dicyclohexyl-1,3,2-dioxaborolan-2-yl)methoxy)triisopropylsilane (7b)

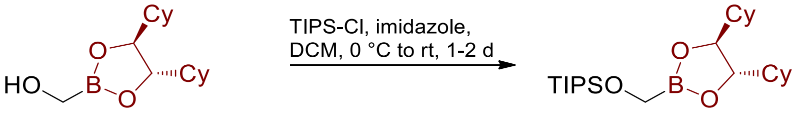

S4b **7b**, 78 %

Under an atmosphere of argon imidazole (6.8 g, 100 mmol, 1.4 eq.) was added to a solution of **S4b** (19 g, 71.4 mmol, 1.0 eq.) in CH_2Cl_2 (400 mL). *tert*-Butyl(chloro)diphenylsilane (15.3 mL, 71.4 mmol, 1.0 eq.) was added dropwise at 0 °C to the reaction mixture and stirring was continued for 17 h. The reaction was quenched by addition of NH_4Cl (aq. sat., 20 mL) and the aqueous phase was extracted with CH_2Cl_2 (3 x 100 mL). The organic phase was dried over $MgSO_4$ and the solvent was removed *in vacuo*. The residue was purified by flash chromatography (SiO_2, CyHex/EtOAc, 9:1, $KMnO_4$), yielding **7b**(23.5 g, 55.6 mmol, 78%) as a colourless oil.

[5] R. P. Singh and D. S. Matteson, *J. Org. Chem.* **2000**, *65*, 6650–6653.

R_f (EA/CH 1:9) = 0.71.

^{1}H NMR (400 MHz, $CDCl_3$): δ (ppm) = 3.93 – 3.86 (m, 2H), 3.65 (d, *J* = 17.3 Hz, 1H), 3.62 (d, *J* = 17.5 Hz, 1H), 1.84 – 1.55 (m, 10 H), 1.39 – 0.89 (m, 33H).

^{13}C NMR (101 MHz, $CDCl_3$): δ (ppm) = 84.0, 43.1, 28.6, 27.5, 26.6, 26.2, 26.0, 18.2, 17.9, 12.4, 12.1, *carbon attached to boron not observed*.

IR (film) v_{max} (cm^{-1}): 2925, 2864, 1510, 1456, 1248, 1097, 883, 683.

ESI-HRMS: calc. for $[C_{24}H_{47}BO_3Si+H]^+$ 423.3466, found 423.3469.

Route to Allitol 12a

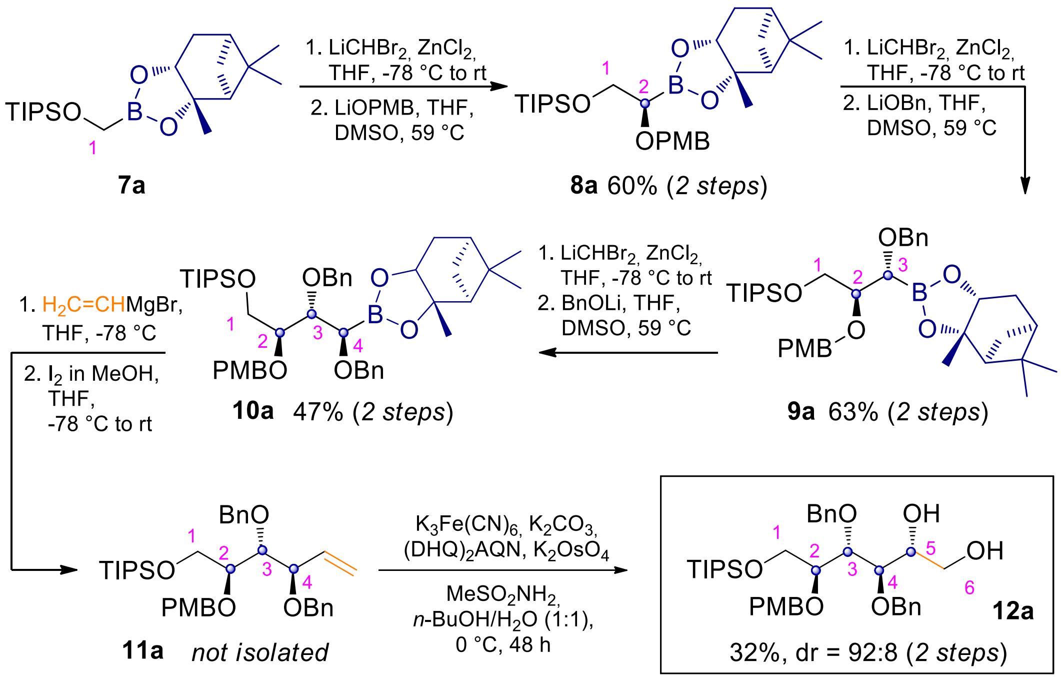

Scheme II: Overview of the synthetic route to Allitol 12a.

Allitol Series – C_2-Building Block (8a)

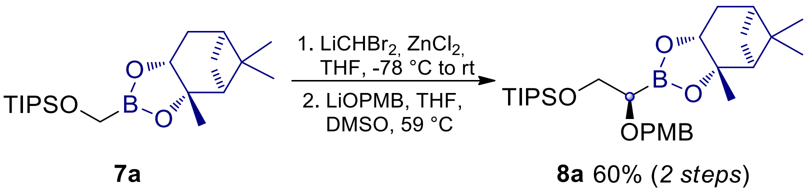

Under an atmosphere of argon LDA was prepared *in situ* by dropwise addition of *n*-BuLi (2.5 M in hexanes, 15.1 mL, 37.7 mmol, 1.3 eq.) to diisopropyl amine (6.5 mL, 46.4 mmol, 1.6 eq.) in THF (28 mL) at -78 °C and stirring for 10 min at rt. This solution was added dropwise to a solution of the boronic ester **7a** (10.6 g, 29.0 mmol, 1.0 eq.) and CH_2Br_2 (20.4 mL, 290 mmol, 10 eq.) in THF (28 mL) under argon at -78 °C. The reaction mixture was stirred for 1 h at -78 °C and then $ZnCl_2$ (1 M in Et_2O, 87 mL, 87 mmol, 3.0 eq.) was added in one portion. The reaction mixture was allowed up to warm up to rt over 17 h. Cyclohexane (50 mL) and NH_4Cl (aq. sat., 50 mL) were added. The mixture was

extracted with Et_2O (3 x 100 mL) and the organic phases were separated and dried over $MgSO_4$. The solvent was removed *in vacuo* to obtain the crude homologation product as a brown oil.

To a solution of 4-methoxybenzyl alcohol* (5.04 mL, 40.6 mmol, 1.4 eq.) in THF (20 mL) under argon, *n*-BuLi (2.5 M in hexanes (16.2 mL, 40.6 mmol, 1.4 eq.) was added dropwise at -78 °C. Stirring was continued for 10 min at rt. This solution of the alcoholate was added dropwise to a solution of the crude homologation product in THF (100 mL) under argon at -78 °C. DMSO (10 mL) was added in one portion and the reaction mixture was heated to 60 °C for 17 h. NH_4Cl (aq. sat., 50 mL) was added and the aqueous phase was extracted with Et_2O (3 x 100 mL). The organic phase was dried over $MgSO_4$ and the solvent was removed *in vacuo*. The residue was purified by flash chromatography (SiO_2, CyHex/EtOAc, 20:1 + 1% NEt_3, $KMnO_4$) yielding **8a** (9.43 g, 18.25 mmol, 63%) as a yellow oil.

R$_f$ (EA/CH 1:20) = 0.22.

$[\alpha]_D^{20}$= +8.1 (0.047, $CHCl_3$).

^{1}H NMR (600 MHz, $CDCl_3$): δ (ppm) = 7.31 – 7.26 (m, 2H), 6.87 – 6.82 (m, 2H), 4.63 (d, *J* f= 12.0 Hz, 1H), 4.58 (d, *J* = 12.0 Hz, 1H), 4.30 (dd, *J* = 8.8, 1.8 Hz, 1H), 3.99 (dd, *J* = 10.6, 4.0 Hz, 1H), 3.93 (dd, *J* = 10.6, 6.1 Hz, 1H), 3.79 (s, 3H), 3.45 (dd, *J* = 6.0, 4.0 Hz, 1H), 2.35 – 2.27 (m, 1H), 2.22 – 2.15 (m, 1H), 2.07 (dd, *J* = 6.0, 4.8 Hz, 1H), 1.92 – 1.85 (m, 2H), 1.39 (s, 3H), 1.28 (s, 3H), 1.21 (d, *J* = 10.9 Hz, 1H), 1.14 – 1.03 (m, 21H), 0.83 (s, 3H).

^{13}C NMR (151 MHz, $CDCl_3$): δ (ppm) = 159.1, 131.5, 129.4, 113.7, 86.5, 78.3, 72.4, 64.9, 55.4, 51.3, 39.6, 38.2, 35.4, 28.8, 27.2, 26.6, 24.1, 18.2, 18.1, 12.1 *carbon attached to boron not observed*.

IR (film) ν_{max} (cm^{-1}): 2939, 2864, 1614, 1512, 1464, 1377, 1342, 1300, 1284, 1246, 1207, 1172, 1095, 1076, 1059, 1030, 1012, 991, 937, 922, 883, 821, 802, 754, 706, 681, 658, 638, 613.

ESI-HRMS: calc. for $[C_{29}H_{49}BO_5Si+Na]^+$ 539.3340, found 539.3334.

***Comment:** Concerning the use of other protecting groups see: (a) D. S. Matteson, A. A. Kandil, *J. Org. Chem.* **1987**, *52*, 5121; (b) D. S. Matteson, K. Mathew. Sadhu, M. L. Peterson, *J. Am. Chem. Soc.* **1986**, *108*, 810; (c) D. S. Matteson, R. Soundararajan, O. C. Ho, W. Gatzweiler, *Organometallics*, **1996**, *15*, 152; (d) K. W. Maurer, R. W. Armstrong, *J. Org. Chem.* **1996**, *61*, 3106. (e) D. S. Matteson, D. Maliakal, L. Fabry-Asztalos, *J. Organomet. Chem.* **2008**, *693*, 2258.

Allitol Series – C3-Building Block (9a)

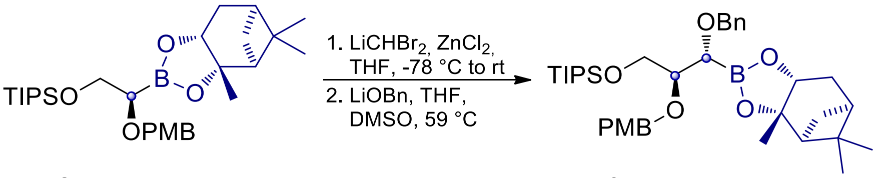

8a **9a** 63% (*2 steps*)

Under an atmosphere of argon LDA was prepared *in situ* by dropwise addition of *n*-BuLi (2.5 M in hexanes, 6.6 mL, 16.4 mmol, 1.3 eq.) to diisopropyl amine (2.8 mL, 20.2 mmol, 1.6 eq.) in THF (13 mL) at -78 °C and stirring for 10 min at rt. This solution was added dropwise under argon to a solution of the boronic ester **8a** (6.50 g, 12.6 mmol, 1.0 eq.) and CH_2Br_2 (8.8 mL, 126 mmol, 10 eq.) in THF (13 mL) at -78 °C. The reaction mixture was stirred for 1 h at -78 °C and then $ZnCl_2$ (1 M in Et_2O, 63 mL, 63 mmol, 5.0 eq.) was added in one portion. The reaction mixture was allowed up to warm up to rt over 17 h. Cyclohexane (50 mL) and NH_4Cl (aq. sat., 50 mL) were added. The mixture was

extracted with Et_2O (3 x 100 mL) and the organic phases were separated and dried over $MgSO_4$. The solvent was removed *in vacuo* to obtain the crude homologation product as a brown oil.

To a solution of benzyl alcohol (1.8 mL, 17.6 mmol, 1.4 eq.) in THF (15 mL) under argon, *n*-BuLi (2.5 M in hexanes, 7.04 mL, 17.6 mmol, 1.4 eq.) was added dropwise at -78 °C and the mixture was stirred for 10 min at rt. This solution of the alcoholate was added dropwise under argon to a solution of the crude homologation product in THF (100 mL) at -78 °C. DMSO (10 mL) was added in one portion and the reaction mixture was heated to 60 °C for 17 h. NH_4Cl (aq. sat., 50 mL) was added and the aqueous phase was extracted with Et_2O (3 x 100 mL). The organic phases were dried over $MgSO_4$ and the solvent was removed *in vacuo*. The residue was purified by flash chromatography (SiO_2, CyHex/EtOAc, 10:1 + 1% NEt_3, $KMnO_4$) yielding **9a** (5.10 g, 8.0 mmol, 63%) as a yellow oil.

R_f (EA/CH 1:9) = 0.38.

$[\alpha]_D^{20}$= +7.6 (0.005, $CHCl_3$).

^{1}H NMR (600 MHz, $CDCl_3$): δ (ppm) = 7.37 – 7.22 (m, 7H), 6.87 – 6.81 (m, 2H), 4.76 – 4.68 (m, 2H), 4.63 – 4.58 (m, 2H), 4.31 (dd, *J* = 8.8, 1.9 Hz, 1H), 3.91 – 3.86 (m, 1H), 3.83 – 3.81 (m, 2H), 3.79 (s, 3H), 3.64 (d, *J* = 2.9 Hz, 1H), 2.34 – 2.26 (m, 1H), 2.16 – 2.07 (m, 1H), 2.09 – 2.03 (m, 1H), 1.90 – 1.82 (m, 2H), 1.36 (s, 3H), 1.28 (s, 3H), 1.25 (d, *J* = 10.9 Hz, 1H), 1.13 – 1.00 (m, 21H), 0.83 (s, 3H).

^{13}C NMR (151 MHz, $CDCl_3$): δ (ppm) = 159.0, 139.2, 131.5, 129.4, 128.3, 127.9, 127.4, 113.6, 86.4, 81.8, 78.3, 72.7, 72.4, 63.8, 55.4, 51.3, 39.6, 38.2, 35.4, 28.8, 27.2, 27.1, 26.4, 24.2, 18.2, 18.2 12.1, 12.0, *carbon attached to boron not observed*.

IR (film) ν_{max} (cm^{-1}): 2939, 2866, 2360, 2341, 1514, 1496, 1489, 1456, 1417, 1377, 1340, 1302, 1282, 1246, 1207, 1172, 1099, 1076, 1030, 883, 821, 798, 735, 683, 658, 615.

ESI-HRMS: calc. for $[C_{37}H_{57}BO_6Si+Na]^+$ 659.3915, found 659.3921.

Allitol Series – C_4-Building Block (10a)

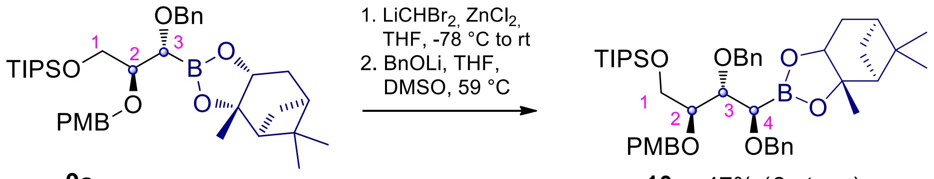

9a **10a** 47% (*2 steps*)

Under an atmosphere of argon LDA was prepared *in situ* by dropwise addition of *n*-BuLi (1.6 M in hexanes, 3.0 mL, 4.8 mmol, 1.3 eq.) to diisopropyl amine (0.83 mL, 5.94 mmol, 1.6 eq.) in THF (3 mL) at -78 °C and stirring for 10 min at rt. This solution was added dropwise to a solution of the boronic ester **9a** (2.36 g, 3.71 mmol, 1.0 eq.) and CH_2Br_2 (2.6 mL, 37 mmol, 10 eq.) in THF (8 mL) under argon at -78 °C. The reaction mixture was stirred for 1 h at -78 °C and then $ZnCl_2$ (1 M in Et_2O, 22.3 mL, 22.3 mmol, 6.0 eq.) was added in one portion. The reaction mixture was allowed up to warm up to rt over 17 h. Cyclohexane (50 mL) and NH_4Cl (aq. sat., 50 mL) were added. The mixture was extracted with Et_2O (3 x 100 mL) and the organic phases were separated and dried over $MgSO_4$. The solvent was removed *in vacuo* to obtain the crude homologation product as a brown oil.

To a solution of benzyl alcohol (0.54 mL, 5.2 mmol, 1.4 eq.) in THF (5 mL) under argon, *n*-BuLi (2.5 M in hexanes, 2.1 mL, 5.2 mmol, 1.4 eq.) was added dropwise at -78 °C and the mixture was stirred for 10 min at rt. This solution of the alcoholate was added dropwise to a solution of the crude homologation product in THF (5 mL) under argon at -78 °C. DMSO (0.5 mL) was added in one portion and the reaction mixture was heated to 60 °C for 17 h. NH_4Cl (aq. sat., 20 mL) was added and the

aqueous phase was extracted with Et_2O (3 x 20 mL). The organic phases were dried over $MgSO_4$ and the solvent was removed *in vacuo*. The residue was purified by flash chromatography (SiO_2, CyHex/EtOAc, 10:1 + 1% NEt_3, $KMnO_4$) yielding **10a** (1.32 mg, 1.74 mmol, 47%) as a yellowish oil.

$\mathbf{R_f}$ (EA/CH 1:10) = 0.50.

$\mathbf{[\alpha]_D^{20}}$= -2.1 (0.007, $CHCl_3$).

^{1}H NMR (600 MHz, $CDCl_3$): δ (ppm) = 7.35 – 7.21 (m, 10H), 7.20 – 7.18 (m, 2H), 6.78 – 6.76 (m, 2H), 4.81 – 4.57 (m, 6H), 4.26 (dd, *J* = 8.8, 2.0 Hz, 1H), 4.02 (dd, *J* = 10.8, 2.3 Hz, 1H), 3.92 (dd, *J* = 8.3, 2.2 Hz, 1H), 3.84 – 3.79 (m, 2H), 3.78 (s, 3H), 3.75 – 3.69 (m, 1H), 2.26 – 2.19 (m, 1H), 2.11 – 2.05 (m, 1H), 2.03 (m, 1H), 1.79 – 1.74 (m, 1H), 1.74 – 1.68 (m, 1H), 1.30 (d, *J* = 10.9 Hz, 1H), 1.28 (s, 3H), 1.26 (s, 3H), 1.13 – 1.00 (m, 21H), 0.80 (s, 3H).

^{13}C NMR (151 MHz, $CDCl_3$): 159.0, 139.4, 139.1, 131.4, 129.8, 128.3, 128.2, 128.0, 127.9, 127.29, 127.26, 113.6, 86.3, 80.8, 79.3, 78.2, 72.9, 72.7, 72.5, 64.7, 55.4, 51.4, 39.6, 38.3, 35.3, 28.7, 27.2, 26.3, 24.2, 18.22, 18.16, 12.13, 12.05.

IR (film) ν_{max} (cm^{-1}): 2940, 2865, 2358, 1616, 1512, 1457, 1375, 1302, 1246, 1095, 1028, 882, 820, 733, 697, 668.

ESI-HRMS: calc. for $[C_{45}H_{65}BO_7Si+Na]^+$ 779.4490, found 779.4494.

AllitolSeriec – Vinylation and subsequent Bishydroxylation to Allitol 12a

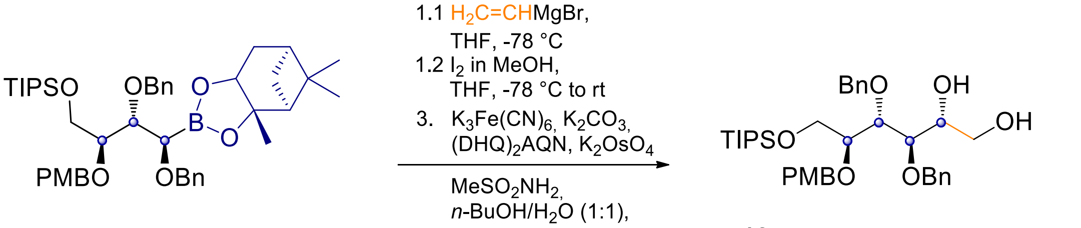

10a **12a** 32%, dr = 92:8 (*2 steps*)

Under an atmosphere of argon vinyl magnesium bromide (0.7 M in THF, 0.059 mmol, 1.0 eq.) was added dropwise to a solution of the boronic ester **10a** (45 mg, 0.059 mmol, 1.0 eq.) in THF (2.0 mL) at -78 °C. The resulting mixture was stirred at rt. for 60 min and cooled to –78 °C. A solution of iodine (16 mg, 0.065 mmol, 1.1 eq.) in MeOH (1 mL) was added dropwise to the reaction mixture at –78 °C. The reaction mixture was then allowed to warm to rt. and stirred for an additional 17 h. It was washed with $Na_2S_2O_3$ solution (sat. aq., 5 mL) and the aqueous layer was extracted with Et_2O (2 × 5 mL). The combined organic layers were dried with $MgSO_4$, filtered and concentrated *in vacuo* to yield the crude alkene **11a**.

To a solution of $K_3[Fe(CN)_6]$ (40 mg, 0.12 mmol, 3.0 eq.), K_2CO_3 (17 mg, 0.12 mmol, 3.0 eq.), $(DHQ)_2AQN$ (3.5 mg, 0.0041 mmol, 10 mol%) and methanesulfonamide (4.0 mg, 0.041 mmol, 1.0 eq.) in 1:1 mixture of *t*-BuOH:H_2O (2 mL) was added Potassium osmate dihydrate (0.3 mg, 0.00082 mmol, 2 mol%) at 0 °C and the mixture was stirred for 30 min. Then the crude alkene **11a** (25 mg, 0.041 mmol, 1.0 eq.) was added dropwise to the reaction mixture at 0 °C, which was allowed to warm up to rt over 24 h. Na_2SO_3 was added at 0 °C and stirring was continued until the yellow colour of the solution turned to grey. The aqueous phase was extracted with EtOAc (3 x 5 mL). The organic phase was dried over $MgSO_4$ and the solvent was removed *in vacuo*. The residue was purified by flash chromatography (SiO_2, CyHex/EtOAc, 4:1, $KMnO_4$) yielding **12a** (12 mg, 0.018 mmol, 32%, d.r.: 92:08, *yield corrected for 12% pinanediol impurity*)* as a colourless oil.

$\mathbf{R_f}$ (EA/CH 1:3) = 0.15.

[α]$_D^{20}$= +7.6 (0.005, $CHCl_3$).

^{1}H NMR (400 MHz, $CDCl_3$): δ (ppm) = 7.37 – 7.19 (m, 12H), 6.90 – 6.82 (m, 2H), 4.76 – 4.48 (m, 6H), 4.06 – 3.49 (m, 10H), 2.20 (s (br.), 2H), 1.15 – 0.93 (m, 21H).

^{13}C NMR (101 MHz, $CDCl_3$): δ (ppm) = 159.5, 138.2, 138.1, 130.2, 129.9, 128.6, 128.2, 128.1, 128.0, 127.9,** 113.9, 79.6, 79.5, 79.4, 73.6, 73.3, 73.2, 71.7, 64.1, 63.6, 55.4, 18.2, 12.1.

IR (film) ν_{max} (cm^{-1}): 2937, 2864, 2360, 1612, 1512, 1465, 1387, 1302, 1246, 1172, 1094, 1030, 882, 820, 668.

ESI-HRMS: calc. for $[C_{37}H_{54}O_7Si+Na]^+$ 661.3536, found 661.3535.

***Comment:** Chromatography was a limiting factor in this multistep reaction. The main fraction was slightly contaminated (yield corrected) and another heavily contaminated product fraction (9 mg) was obtained. However, attempts to isolate **12a** from the latter fraction in reasonable purity were not successful and thus this material is not included in the reported yield.

****Comment:** Not all aromatic resonances were resolved

Route to Glucitol 12b

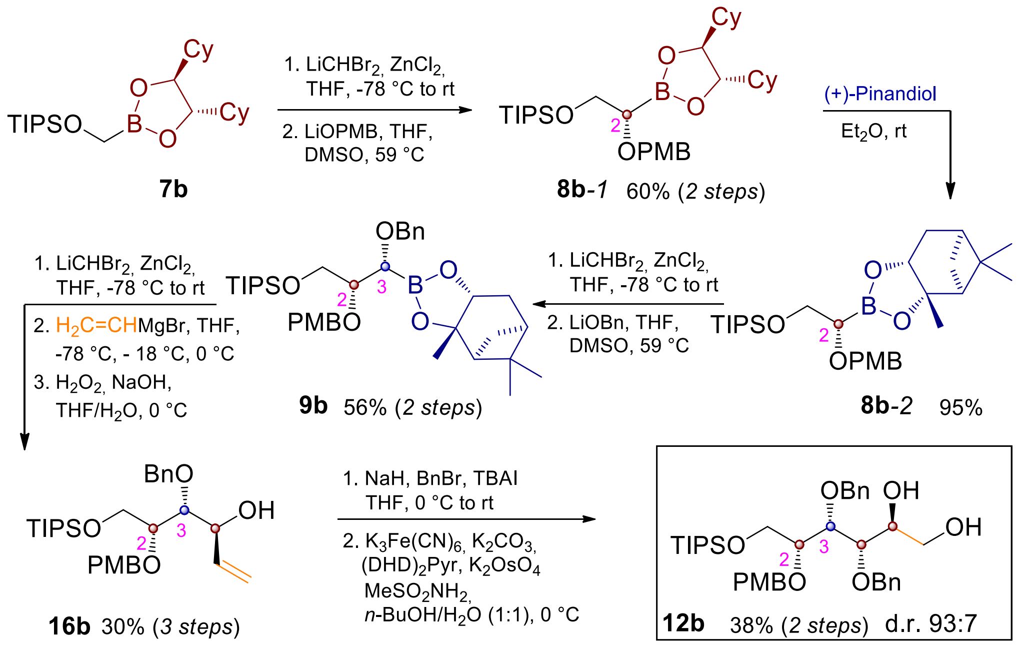

Scheme III Overview of the synthetic route to Glucitol 12b.

Glucitol Series – DICHED-C$_2$Buildingblock (8b-*1*)

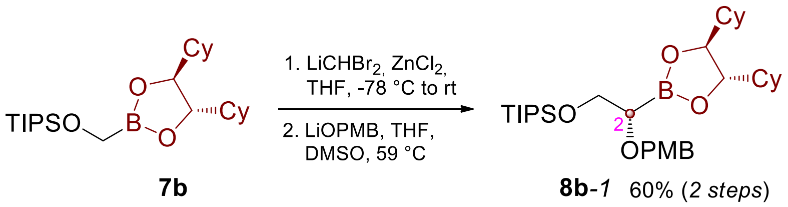

Under an atmosphere of argon LDA was prepared *in situ* by dropwise addition of *n*-BuLi (2.5 M in hexane, 27 mL, 67.6 mmol, 1.3 eq.) to diisopropyl amine (11.7 mL, 83.2 mmol, 1.6 eq.) in THF

(100 mL) at -78 °C and stirring for 10 min at rt. This solution was added dropwise under argon to a solution of boronic ester **7b** (22 g, 52 mmol, 1.0 eq.) and CH_2Br_2 (36.5 mL, 520 mmol, 10 eq.) in THF (400 mL) at -78 °C. The reaction mixture was stirred for 1 h at -78 °C and then $ZnCl_2$ (1 M in Et_2O, 156 mL, 156 mmol, 3.0 eq.) was added in one portion. The reaction mixture was allowed to warm to rt over 17 h. Cyclohexane (50 mL) and NH_4Cl (aq. sat., 50 mL) were added. The mixture was extracted with Et_2O (3 x 100 mL) and the organic phase was separated and dried over $MgSO_4$. The solvent was removed *in vacuo* to obtain the crude homologation product as a brown oil.

To a solution of 4-methoxybenzyl alcohol* (9.0 mL, 72.8 mmol, 1.4 eq.) in THF (100 mL) under argon, *n*-BuLi (2.5 M in hexane, 29.1 mL, 72.8 mmol, 1.4 eq.) was added dropwise at -78 °C and the mixture was stirred for 10 min at rt. This solution of the alcoholate was added dropwise to a solution of the crude homologation product in THF (250 mL) under argon at -78 °C. DMSO (20 mL) was added in one portion and the reaction mixture was heated to 60 °C for 17 h. NH_4Cl (aq. sat., 50 mL) was added and the aqueous phase was extracted with Et_2O (3 x 100 mL). The organic phase was dried over $MgSO_4$ and the solvent was removed *in vacuo*. The residue was purified by flash chromatography (SiO_2, CyHex/EtOAc, 20:1 + 1% NEt_3, $KMnO_4$) yielding **8b**-*1* (17.7 g, 31 mmol, 60%) as a yellow oil.

R_f (EA/CH 1:9) = 0.48.

$[\alpha]_D^{20}$= -19.3 (0.005, $CHCl_3$).

^{1}H NMR (400 MHz, $CDCl_3$): δ (ppm) = 7.29 (d, *J* = 8.6 Hz, 2H), 6.85 (d, *J* = 8.6 Hz, 2H), 4.62 (d, *J* = 11.7 Hz, 1H), 4.58 (d, *J* = 11.8 Hz, 1H), 3.97 – 3.92 (m, 2H), 3.91 – 3.86 (m, 2H), 3,79 (s, 3H), 3.46 (dd, *J* = 6.1, 4.6 Hz, 1H), 1.77 – 1.57 (m, 10H), 1.23 – 0.96 (m, 33H).

^{13}C NMR (101 MHz, $CDCl_3$): δ (ppm) = 159.1, 131.7, 129.4, 113.7, 83.9, 72.6, 65.1, 55.4, 43.0, 28.4, 27.6, 26.6, 26.2, 26.0, 18.2, 12.1, *carbon attached to boron not observed*.

IR (film) ν_{max} (cm^{-1}): 2925, 2864, 1450, 1248, 1103, 883, 683.

ESI-HRMS: calc. for $[C_{33}H_{57}BO_5Si+Na]^+$ 595.3966, found 595.3972.

***Comment:** Concerning the use of other protecting groups see: (a) D. S. Matteson, A. A. Kandil, *J. Org. Chem.*, 1987,**52**, 5121; (b) D. S. Matteson, K. Mathew. Sadhu, M. L. Peterson, *J. Am. Chem. Soc.*, 1986,**108**, 810; (c) D. S. Matteson, R. Soundararajan, O. C. Ho, W. Gatzweiler, *Organometallics*, 1996,**15**, 152; (d) K. W. Maurer, R. W. Armstrong, *J. Org. Chem.*, 1996,**61**, 3106. (e) D. S. Matteson, D. Maliakal, L. Fabry-Asztalos, *J. Organomet. Chem.*, 2008, **693**, 2258.

Glucitol Series – Pinanediol-C_2Buildingblock (8b-*2*)

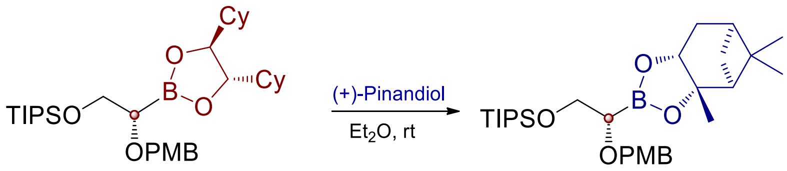

8b-*1* **8b**-*2* 83%

Under an atmosphere of argon boronic ester **8b**-*1* (5.76 g, 13.6 mmol, 1.0 eq.) was dissolved in Et_2O (100 mL) and (+)-Pinanediol (2.6 g, 14.96 mmol, 1.1 eq.) was added. The mixture was stirred for 17 h at rt. The solvent was removed *in vacuo*. The residue was purified by flash chromatography (SiO_2, CyHex/EtOAc, 20:1, $KMnO_4$), yielding the boronic ester **8b**-*2* (5.86 g, 11 mmol, 83%) as a colourless oil.

R_f (EA/CH 1:9) = 0.55.

[α]$_D^{20}$= +0.8 (0.008, $CHCl_3$).

^{1}H NMR (400 MHz, $CDCl_3$): δ (ppm) = 7.31-7.27 (m, 2H), 6.86-6.83 (m, 2H), 4.62 (d, *J* = 11.9 Hz, 1H), 4.57 (d, *J* = 11.9 Hz, 1H), 4.30 (dd, *J* = 8.8, 1.9 Hz, 1H), 3.99 (dd, *J* = 10.6, 4.1 Hz, 1H), 3.93 (dd, *J* = 10.5, 6.0 Hz, 1H), 3.79 (s, 3H), 3.44 (dd, *J* = 5.9, 4.1 Hz, 1H), 2.39 – 2.29 (m, 1H), 2.18 (dtd, *J* = 10.9, 6.1, 2.3 Hz, 1H), 2.06 (dd, *J* = 6.1, 4.8 Hz, 1H), 1.92 – 1.85 (m, 2H), 1.39 (s, 3H), 1.28 (s, 3H), 1.19 (d, *J* = 10.8 Hz, 1H), 1.13 – 0.99 (m, 21H), 0.83 (s, 3H).

^{13}C NMR (101 MHz, $CDCl_3$): δ (ppm) = 159.1, 131.5, 129.4, 113.7, 86.5, 78.3, 72.5, 65.0, 55.4, 51.3, 39.6, 38.2, 35.5, 28.7, 27.2, 26.6, 24.1, 18.2, 18.1, 12.1, 12.0 *carbon attached to boron not observed*.

IR (film) v_{max} (cm^{-1}): 2934, 2864, 1514, 1458, 1377, 1246, 1093, 881, 679.

ESI-HRMS: calc. for $[C_{29}H_{49}BO_5Si+Na]^+$ 539.3340, found 539.3334.

Glucitol Series –C3Buildingblock (9b)

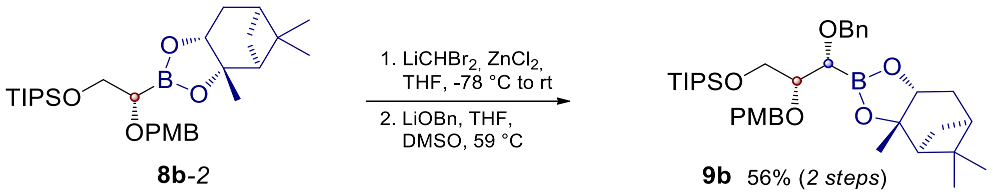

Under an atmosphere of argon LDA was prepared *in situ* by dropwise addition of *n*-BuLi (2.5 M in hexanes, 5.7 mL, 14.3 mmol, 1.3 eq.) to diisopropyl amine (2.5 mL, 17.6 mmol, 1.6 eq.) in THF (10 mL) at -78 °C and stirring for 10 min at rt. This solution was added dropwise under argon to a solution of boronic ester **8b**-*2* (5.69 g, 11 mmol, 1.0 eq.) and CH_2Br_2 (7.7 mL, 110 mmol, 10 eq.) in THF (100 mL) at -78 °C. The reaction mixture was stirred for 1 h at -78 °C and then $ZnCl_2$ (1 M in Et_2O, 55 mL, 55 mmol, 5.0 eq.) was added in one portion. The reaction mixture was allowed up to warm up to rt over 17 h. Cyclohexane (50 mL) and NH_4Cl (aq. sat., 50 mL) were added. The mixture was extracted with Et_2O (3 x 100 mL), the organic phases were separated and dried over $MgSO_4$. The solvent was removed *in vacuo* to obtain the crude homologation product as a brown oil.

To a solution of benzyl alcohol (1.6 mL, 15.4 mmol, 1.4 eq.) in THF (10 mL) under argon, *n*-BuLi (2.5 M in hexanes, 6.2 mL, 15.4 mmol, 1.4 eq.) was added dropwise at -78 °C and the mixture was stirred for 10 min at rt. This solution of the alcoholate was added dropwise to a solution of the crude homologation product in THF (100 mL) under argon at -78 °C. DMSO (10 mL) was added in one portion and the reaction mixture was heated to 60 °C for 17 h. NH_4Cl (aq. sat., 100 mL) was added and the aqueous phase was extracted with Et_2O (3 x 100 mL). The organic phase was dried over $MgSO_4$ and the solvent was removed *in vacuo*. The residue was purified by flash chromatography (SiO_2, CyHex/EtOAc, 20:1 + 1% NEt_3, $KMnO_4$) yielding **9b** (3.94 g, 6.2 mmol, 56%) as a yellow oil.

R$_f$ (EA/CH 1:9) = 0.42.

[α]$_D^{20}$= +1.65 (0.006, $CHCl_3$).

^{1}H NMR (400 MHz, $CDCl_3$) δ = 7.39 – 7.19 (m, 7H), 6.90 – 6.78 (m, 2H), 4.73 – 4.51 (m, 4H), 4.29 (dd, *J* = 8.7, 1.9, 1H), 4.00 – 3.89 (m, 1H), 3.85 – 3.74 (m, 5H), 3.59 – 3.52 (m, 1H), 2.37 – 2.25 (m, 1H), 2.23 – 2.11 (m, 1H), 2.10 – 2.02 (m, 1H), 1.94 – 1.82 (m, 2H), 1.36 (s, 3H), 1.28 (s, 3H), 1.20 (d, *J* = 10.9, 1H), 1.05 (dt, *J*=5.1, 3.2, 21H), 0.83 (s, 3H).

^{13}**C NMR** (101 MHz, $CDCl_3$): 159.1, 139.3, 131.6, 129.6, 128.2, 128.0, 127.4, 113.6, 86.4, 81.8, 78.3, 73.6, 72.9, 63.8, 55.4, 51.3, 39.6, 38.3, 35.4, 28.8, 27.2, 26.6, 24.2, 18.2, 17.9, 12.11, 12.05 *carbon attached to boron not observed*.

IR (film) ν_{max} (cm^{-1}): 2939, 2866, 1246, 1068, 1030, 881, 733, 681.

ESI-HRMS: calc. for $[C_{37}H_{57}BO_6Si+Na]^+$ 659.3915, found 659.3912.

Glucitol Series –Vinylation (16b)

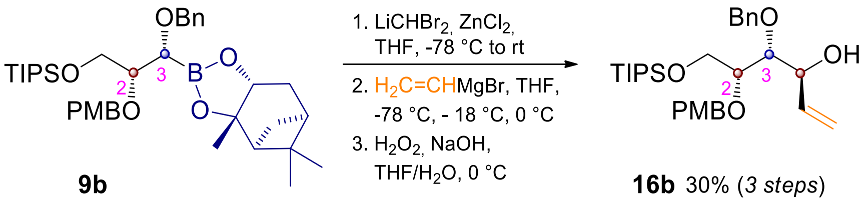

Under an atmosphere of argon LDA was prepared *in situ* by dropwise addition of *n*-BuLi (2.5 M in hexane, 3.18 mL, 7.96 mmol, 1.3 eq.) to diisopropyl amine (1.37 mL, 9.79 mmol, 1.6 eq.) in THF (5 mL) at -78 °C and stirring for 10 min at rt. This solution was added dropwise under argon to a solution of boronic ester **9b** (3.9 g, 6.12 mmol, 1.0 eq.) and CH_2Br_2 (4.3 mL, 61.2 mmol, 10 eq.) in THF (100 mL) at -78 °C. The reaction mixture was stirred for 1 h at -78 °C and then $ZnCl_2$ (1 M in Et_2O, 36.7 mL, 36.7 mmol, 6.0 eq.) was added in one portion. The reaction mixture was allowed up to warm up to rt over 17 h. Cyclohexane (50 mL) and NH_4Cl (aq. sat., 50 mL) were added. The mixture was extracted with Et_2O (3 x 100 mL) and the organic phase was separated and dried over $MgSO_4$. The solvent was removed *in vacuo* to obtain the crude homologation product as a brown oil.

Under an atmosphere of argon vinyl magnesium bromide (0.7 M in THF, 15.8 mL, 11 mmol, 1.8 eq.) was added dropwise to a solution of the boronic ester in THF (100 mL) at -78 °C. After stirring for 30 min at -78 °C, the reaction mixture was stored in the freezer (-18 °C) for 17 h. The solution was then stirred at 0 °C for 4 h and then H_2O_2 (30 wt%, 11 mL, 122 mmol, 20 eq.) and NaOH (4 M, 24.5 mL, 122 mmol, 20 eq.) were added dropwise at 0 °C. The reaction was stirred for 4 h at the same temperature. $Na_2S_2O_3$ (aq. sat., 20 mL) was slowly added at 0 °C and the aqueous phase was extracted with EtOAc (3 x 50 mL). The organic phase was dried over $MgSO_4$ and the solvent was removed *in vacuo*. The residue was purified by flash chromatography (SiO_2, CyHex/EtOAc, 9:1, $KMnO_4$) yielding **16b** (950 mg, 1.84 mmol, 30%) as a pale yellow oil.

$\mathbf{R_f}$ (EA/CH 1:9) = 0.16.

$[\alpha]_D^{20}$= -9.7 (0.004, $CHCl_3$).

^{1}**H NMR** (400 MHz, $CDCl_3$) δ = 7.36 – 7.27 (m, 5H), 7.26 – 7.22 (m, 2H), 6.86 – 6.83 (m, 2H), 5.88 (ddd, *J*=17.2, 10.5, 5.6, 1H), 5.31 (ddd, *J*=17.2, 1.6, 1.6, 1H), 5.16 (ddd, *J*=10.5, 1.6, 1.6, 1H), 4.74 (d, *J*=11.3, 1H), 4.65 (d, *J*=7.9, 1H), 4.62 (d, *J*=7.8, 1H), 4.53 (d, *J*=11.4, 1H), 4.35 – 4.30 (m, 1H), 3.88 (d, *J*=5.2, 2H), 3.80 (s, 3H), 3.65 (dd, *J*=5.1, 5.1, 1H), 2.79 (s(br.), 1H), 3.60 (dd, *J*=4.8, 4.2, 1H), 1.10 – 1.02 (m, 21H).

^{13}**C NMR** (101 MHz, $CDCl_3$): δ (ppm) = 159.4, 138.5, 130.7, 129.8, 128.5, 128.3, 127.9, 116.1, 113.9, 81.8, 80.3, 75.1, 72.9, 72.4, 63.0, 55.4, 18.16, 18.15, 12.0 *one sp^2-carbon was not observed at this resolution*.

IR (film) ν_{max} (cm^{-1}): 2941, 2866, 1514, 1458, 1248, 1066, 1037, 881, 683.

ESI-HRMS: calc. for $[C_{30}H_{46}O_5Si+Na]^+$ 537.3012, found 537.3003.

Glucitol Series – Benzyl Protection (11b)

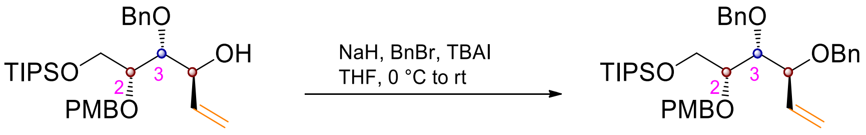

16b **11b** 94%

Under an atmosphere of Argon sodium hydride (60% dispersion in mineral oil, 86 mg, 2.17 mmol, 1.4 eq.) was added to a solution of **16b** (800 mg, 1.55 mmol, 1.0 eq.) in THF (15 mL) at 0 °C. The mixture was stirred for 30 min at rt. Then benzyl bromide (0.26 mL, 2.17 mmol, 1.4 eq.) and TBAI (57 mg, 0.15 mmol, 10 mol%) were added dropwise at 0 °C. The reaction mixture was allowed to warm up to rt over 17 h. NH_4Cl (aq. sat. 15 mL) was added and the aqueous phase was extracted with EtOAc (3 x 15 mL). The organic phase was dried over $MgSO_4$ and the solvent was removed *in vacuo*. The residue was purified by flash chromatography (SiO_2, CyHex/EtOAc, 20:1, $KMnO_4$) yielding **11b** (882 mg, 1.45 mmol, 94%) as a yellow oil.

R_f (EA/CH 1:9) = 0.33.

$[\alpha]_D^{20}$= +3.4 (0.005, $CHCl_3$).

^{1}H NMR (400 MHz, $CDCl_3$) δ = 7.31 – 7.10 (m, 12H), 6.78 – 6.68 (m, 2H), 5.70 (ddd, *J*=17.2, 10.5, 7.7, 1H), 5.20 – 5.09 (m, 2H), 4.72 (d, *J*=11.5, 1H), 4.60 (d, *J*=11.4, 1H), 4.56 (d, *J*=11.5, 1H), 4.53 (d, *J*=11.7, 1H), 4.39 (d, *J*=11.4, 1H), 4.30 (d, *J*=11.6, 1H), 4.13 – 4.05 (m, 1H), 3.72 – 3.66 (m, 1H), 3.69 (s, 3H), 3.62 – 3.57 (m, 2H), 3.55 – 3.51 (m, 1H), 0.92 (m, 21H).
^{13}C NMR (101 MHz, $CDCl_3$): δ (ppm) = 159.2, 139.1, 138.7, 135.9, 131.3, 129.7, 128.5, 128.4, 128.3, 128.1, 127.6, 127.5, 118.7, 113.7, 81.8, 81.2, 79.8, 75.4, 72.8, 72.3, 63.2, 55.4, 18.2, 12.0.

IR (film) ν_{max} (cm^{-1}): 2941, 2864, 1514, 1456, 1248, 1089, 1066, 733, 694.

ESI-HRMS: calc. for $[C_{37}H_{52}O_5Si+Na]^+$ 627.3482, found 627.3472.

Glucitol Series – Bishydroxylation (12b)

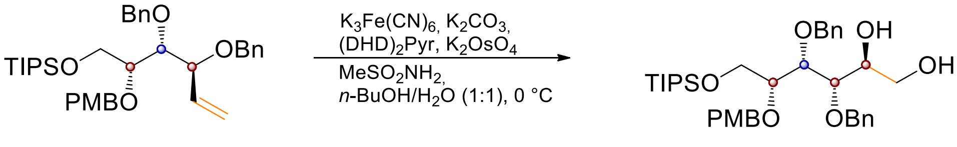

11b **12b** 40% d.r. 93:7

To a solution of $K_3[Fe(CN)_6]$ (988 mg, 3.0 mmol, 3.0 eq.), K_2CO_3 (414 mg, 3.0 mmol, 3.0 eq.), $(DHQ)_2Pyr$ (88 mg, 0.1 mmol, 10 mol%) and methanesulfonamide (95 mg, 1.0 mmol, 1.0 eq.) in a 1:1 mixture of *t*BuOH:H_2O (20 mL) was added Potassium osmate dihydrate (7.4 mg, 0.02 mmol, 2 mol%) at 0 °C. The mixture was stirred for 30 min and the alkene **11b** (604 mg, 1.0 mmol, 1.0 eq.) was added dropwise at 0 °C. Stirring was continued for 48 h at 0 °C. Na_2SO_3 was added at 0 °C and the mixture was stirred until its yellow colour turned to grey. The aqueous phase was extracted with EtOAc (3 x 15 mL). The organic phase was dried over $MgSO_4$ and the solvent was removed *in vacuo*. The residue was purified by flash chromatography (SiO_2, CyHex/EtOAc, 4:1, $KMnO_4$) yielding **12b** (259 mg, 0.4 mmol, 40%, d.r. 93:7) as a colourless oil.

R_f (EA/CH 1:3) = 0.15.

$[\alpha]_D^{20}$= -18.3 (0.013, $CHCl_3$).

^{1}H NMR (400 MHz, $CDCl_3$): δ (ppm) = 7.31 – 7.18 (m, 12H), 6.85 – 6.76 (m, 2H), 4.64 – 4.39 (m, 6H), 3.87 (dt, *J* = 5.0, 1.2 Hz, 1H), 3.82 – 3.73 (m, 7H), 3.71 – 3.64 (m, 2H), 3.58 (dd, *J* = 11.3, 4.6 Hz, 1H), 1.09 – 0.93 (m, 21H).

^{13}C NMR (101 MHz, $CDCl_3$): δ (ppm) = 159.5, 138.0, 137.9, 130.2, 129.9, 128.58, 128.56, 128.2, 128.1, 128.0,* 113.8, 78.5, 77.6, 76.7 (from HSQC),** 74.0, 73.6, 73.1, 72.0, 64.0, 62.4, 55.4, 18.2, 12.0.

IR (film) ν_{max} (cm^{-1}): 3446, 2941, 2866, 1514, 1456, 1248, 1064, 696.

ESI-HRMS: calc. for $[C_{37}H_{54}O_7Si+Na]^+$ 661.3536, found 661.3533.

***Comment:** Not all aromatic resonances were resolved

****Comment:** The resonance signal at 76.7 ppm is hidden under the $CDCl_3$-triplett. Its position was ascertained by HSQC.

Route to Mannitol 12c

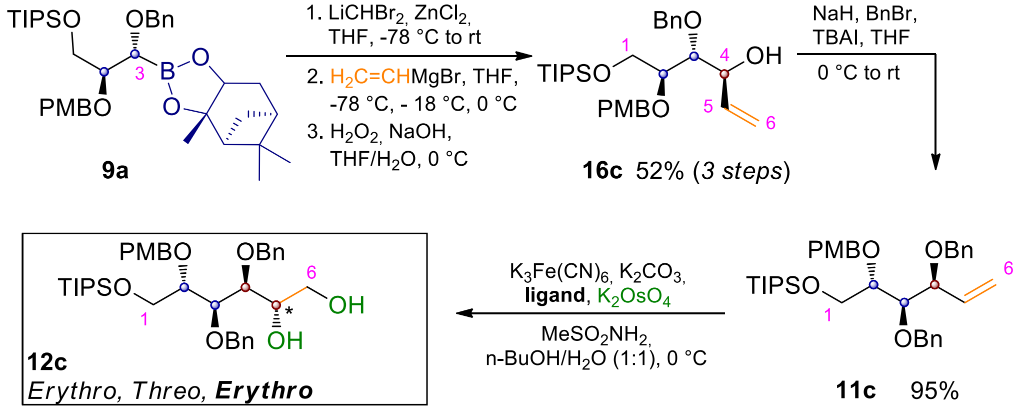

Scheme IV: Overview for the Synthesis of Mannitol 12c.

Mannitol Series - Vinylation (16c)

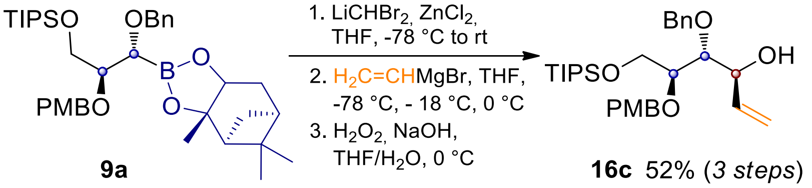

Under an atmosphere of Argon LDA was prepared by dropwise addition of *n*-BuLi (2.5 M in hexane, 1.04 mL, 2.6 mmol, 1.3) to diisopropyl amine (0.45 mL, 3.2 mmol, 1.6 eq.) in THF (5 mL) at -78 °C and stirring for 10 min at rt. This solution was added dropwise under argon to a solution of boronic ester **9a** (1.27 g, 2.0 mmol, 1.0 eq.) and CH_2Br_2 (1.4 mL, 20 mmol, 10 eq.) in THF (15 mL) at -78 °C. The reaction mixture was stirred for 1 h at -78 °C and then $ZnCl_2$ (1 M in Et_2O, 12 mL, 12 mmol, 6.0 eq.) was added in one portion. The reaction mixture was allowed to warm up to rt over 17 h. Cyclohexane (50 mL) and NH_4Cl (aq. sat., 50 mL) were added. The mixture was extracted with Et_2O (3 x 100 mL) and the organic phase was separated and dried over $MgSO_4$. The solvent was removed *in vacuo* to obtain the crude homologation product as a brown oil.

Under an atmosphere of argon vinyl magnesium bromide (0.7 M in THF, 5.7 mL, 4.0 mmol, 2.0 eq.) was added dropwise to a solution of the crude homologation product in THF (15 mL) at -78 °C. After stirring for 30 min. at -78 °C, the reaction mixture was stored in the freezer (-18 °C) for 17 h. The

solution was stirred at 0 °C for 4 h and then H_2O_2 (30 wt%, 3.1 mL, 40 mmol, 20 eq.) and NaOH (3.75 M, 10.7 mL, 40 mmol, 20 eq.) were added dropwise at 0 °C. The reaction mixture was stirred for 3 h at 0 °C, diluted with water (100 mL) and extracted with EtOAc (3 x 25 mL). The organic phase was dried over $MgSO_4$ and the solvent was removed *in vacuo*. The residue was purified by flash chromatography (SiO_2, CyHex/EtOAc, 9:1, $KMnO_4$) yielding **16c** (534 mg, 1.04 mmol, 52%) as a pale yellow oil.

R_f (EA/CH 1:9) = 0.34.

$[\alpha]_D^{20}$= -2.3 (0.006, $CHCl_3$).

^{1}H NMR (400 MHz, $CDCl_3$) δ = 7.37 – 7.21 (m, 7H), 6.91 – 6.82 (m, 2H), 6.00 (ddd, *J*=17.2, 10.6, 4.9, 1H), 5.39 (ddd, *J*=17.3, 1.8, 1.8, 1H), 5.22 (ddd, *J*=10.6, 1.7, 1.7, 1H), 4.73 (d, *J*=10.7, 1H), 4.64 (d, *J*=11.6, 1H), 4.61 (d, *J*=11.6, 1H), 4.49 (d, *J*=10.8, 1H), 4.41 (dddd, *J*=5.0, 3.4, 1.8, 1.8, 1H), 3.99 (dd, *J*=10.8, 3.6, 1H), 3.83 (dd, *J*=10.7, 5.2, 1H), 3.80 (s, 3H), 3.70 (ddd, *J*=6.7, 5.2, 3.6, 1H), 3.63 (dd, *J*=6.6, 3.3, 1H), 3.08 (s (br.), 1H,)1.17 – 0.99 (m, 21H).

^{13}C NMR (151 MHz, $CDCl_3$): δ (ppm) = 159.5, 138.5, 138.2, 130.4, 129.9, 128.5, 128.2, 128.0, 115.7, 114.0, 80.5, 79.9, 73.8, 73.0, 71.6, 63.5, 55.4, 18.2, 12.1.

IR (film) ν_{max} (cm^{-1}): 2942, 2865, 1512, 1457, 1419, 1387, 1302, 1247, 1173, 1090, 1063, 1036, 995, 920, 881, 820, 736, 682, 660.

ESI-HRMS: calc. for $[C_{30}H_{46}BO_5Si+Na]^+$ 537.3012, found 537.3003.

Mannitol Series – Benzyl protection (11c)

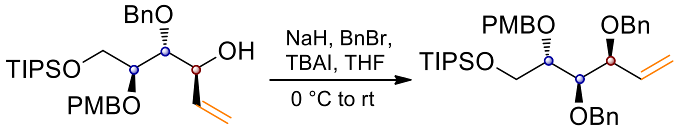

16c **11c** 95%

Under an atmosphere of argon sodium hydride (60% dispersion in mineral oil, 15 mg, 0.38 mmol, 1.4 eq.) was added to a solution of **16c** (142 mg, 0.27 mmol, 1.0 eq.) in THF (2 mL) at 0 °C and the mixture was stirred for 30 min at rt. Then benzyl bromide (0.045 mL, 0.38 mmol, 1.4 eq.) and subsequently TBAI (4.3 mg, 0.0135 mmol, 5 mol%) were added dropwise at 0 °C. The reaction mixture was allowed to warm up to rt over 17 h. NH_4Cl (aq. sat. 5 mL) was added and the aqueous phase was extracted with EtOAc (3 x 5 mL). The organic phase was dried over $MgSO_4$ and the solvent was removed *in vacuo*. The residue was purified by flash chromatography (SiO_2, CyHex/EtOAc, 20:1, $KMnO_4$) yielding **11c**(174 mg, 0.26 mmol, 95%) as a yellow oil.

R_f (EA/CH 1:20) = 0.18.

$[\alpha]_D^{20}$= +6.87 (0.033, $CHCl_3$).

^{1}H NMR (400 MHz, $CDCl_3$) δ = 7.42 – 7.22 (m, 10H), 7.25 – 7.11 (m, 2H), 6.88 – 6.76 (m, 2H), 5.90 (ddd, *J*=17.7, 10.3, 7.6, 1H), 5.33 (ddd, *J*=17.4, 1.8, 1.0, 1H), 5.27 (ddd, *J*=10.5, 1.6, 0.5, 1H), 4.69 (s, 2H), 4.65 (d, *J*=11.0, 1H), 4.62 (d, *J*=11.9, 1H), 4.36 (d, *J*=7.5, 1H), 4.33 (d, *J*=8.3, 1H), 4.10 (dd, *J*=7.5, 4.7, 1H), 4.03 (dd, *J*=10.6, 3.1, 1H), 3.84 (dd, *J*=10.6, 6.1, 1H), 3.81 – 3.73 (m, 1H), 3.77 (s, 3H), 3.64 (dd, *J*=5.8, 4.5, 1H), 1.15 – 0.98 (m, 21H).

^{13}C NMR (101 MHz, $CDCl_3$): δ (ppm) = 159.1, 138.9, 138.8, 136.4, 131.2, 129.5, 128.5, 128.3, 128.2, 128.0, 127.9, 127.5, 118.4, 113.7, 82.1, 80.7, 79.9, 74.8, 72.6, 70.7, 63.9, 55.4, 18.22, 18.15, 12.1.

IR (film) v_{max} (cm^{-1}): 2941, 2864, 1612, 1585, 1514, 1454, 1421.28, 1248, 1172, 1089, 1066, 1030, 1012, 997, 927, 881, 819, 806, 733, 694, 659.

ESI-HRMS: calc. for $[C_{37}H_{52}O_5Si+Na]^+$ 627.3482, found 627.3476.

Mannitol Series – Bishydroxylation (12c)

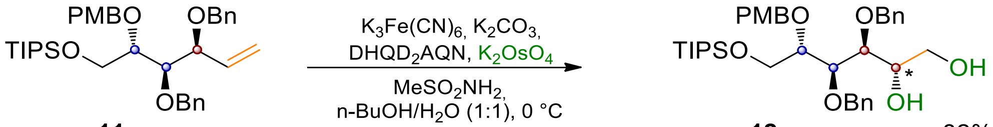

11c **12c** 82% d.r. 99:1

To a solution of $K_3[Fe(CN)_6]$ (79 mg, 0.24 mmol, 3.0 eq.), K_2CO_3 (33 mg, 0.24 mmol, 3.0 eq.), $(DHQD)_2AQN$ (6.8 mg, 0.008 mmol, 10 mol%) and methanesulfonamide (7.6 mg, 0.08 mmol, 1.0 eq.) in a 1:1 mixture of *t*BuOH:H_2O (2 mL) was added potassium osmate dihydrate (0.5 mg, 0.0016 mmol, 2 mol%) at 0 °C and the mixture was stirred for 30 min. Then alkene **11c** (50 mg, 0.08 mmol, 1.0 eq.) was added dropwise to the reaction mixture at 0 °C and the mixture was stirred over 48 h at 0 °C. Na_2SO_3 was added at 0 °C and the mixture was stirred until the yellow colour of the solution turned to grey. The aqueous phase was extracted with EtOAc (3 x 5 mL). The organic phase was dried over $MgSO_4$ and the solvent was removed *in vacuo*. The residue was purified by flash chromatography (SiO_2, CyHex/EtOAc, 4:1, $KMnO_4$) yielding **12c** (41 mg, 0.064 mmol, 80%, d.r.: 99:1) as a colourless oil.

$\mathbf{R_f}$ (EA/CH 1:3) = 0.15.

$[\alpha]_D^{20}$= +0.97 (0.047, $CHCl_3$).

^{1}H NMR (400 MHz, $CDCl_3$): δ (ppm) = 7.39 - 7.16 (m, 12 H), 6.92 - 6.77 (m, 2 H), 4.78 (d, *J* = 11.1 Hz, 1 H), 4.65 (s, 2 H), 4.62 (d, *J* = 12.2 Hz, 1 H), 4.52 (d, *J* = 11.1 Hz, 1 H), 4.48 (d, *J* = 11.4 Hz, 1 H), 4.05 (dd, *J* = 3.7, 10.8 Hz, 1 H), 3.91 (dd, *J* = 6.3, 11.0 Hz, 1 H), 3.88 - 3.80 (m, 3 H), 3.79 (s, 3 H), 3.72 (dd, *J* = 4.4, 7.3 Hz, 1 H), 3.68 (dd, *J* = 3.5, 11.3 Hz, 1 H), 3.62 (dd, *J* = 4.9, 11.6 Hz, 1 H), 1.10 - 0.98 (m, 21 H).

^{13}C NMR (101 MHz, $CDCl_3$): δ (ppm) = 159.4, 138.23, 138.20, 130.4, 129.8, 128.5, 128.12, 128,1, 127.9, 127.91, 127.89,* 113.8, 80.6, 79.2, 78.6, 73.9, 73.6, 73.1, 71.9, 64.0, 63.7, 55.4, 18.2, 12.0.

IR (film) v_{max} (cm^{-1}): 3406, 2941, 2866, 2360, 2341, 1612, 1514, 1462, 1329, 1304,1248, 1091, 1066, 1037, 883, 819, 735, 696.

ESI-HRMS: calc. for $[C_{37}H_{54}O_7Si+Na]^+$ 661.3536, found 661.3530.

Comment: Resolution of the resonances at 127.91 and 127.89 required application of a Lorenz-Gaussian window function.

Inverting the Stereocenter Formed by Bishydroxylation

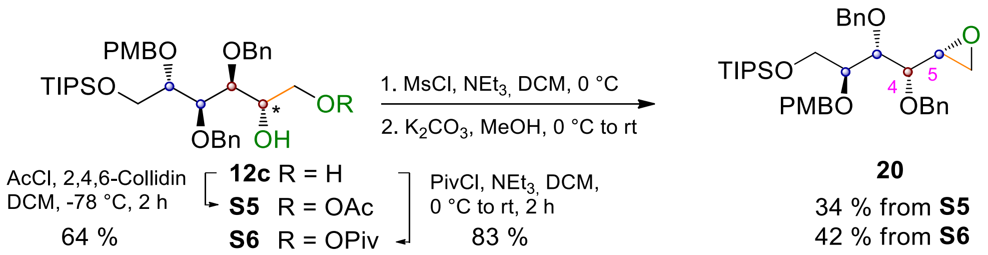

Scheme V Inversion of the stereocenter at C[5] was achieved by acetylation, mesylation, and epoxide formation.

Regioselective Acetate Protection (S5)

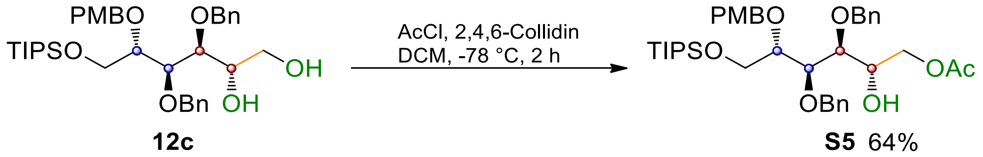

Acyl chloride (1 M in CH_2Cl_2, 0.4 mL, 0.4 mmol, 2.6 eq.) was added in a drop wise manner to a solution of **12c** (100 mg, 0.16 mmol, 1.0 eq.) and 2,4,6-collidine (0.17 mL, 1.32 mmol, 8.28 eq.) in CH_2Cl_2 (4 mL) under argon at -78 °C and the mixture was stirred for 1 h. Et_2O (5 mL) was added and the reaction mixture was allowed to warm up to rt. After that $NaHCO_3$ (sat. aq., 5 mL) was added and the mixture was extracted with Et_2O (3 x 5 mL). The combined organic phases were dried over $MgSO_4$ and the solvents were removed *in vacuo*. The residue was purified by flash chromatography (SiO_2, CyHex/EtOAc, 2:1, $KMnO_4$) yielding **S5** (71 mg, 0.104 mmol, 65%) as a colorless oil.

R_f (EA/CH 1:4) = 0.03.

^{1}H-NMR (400 MHz, $CDCl_3$): δ (ppm) = 7.34 – 7.20 (m, 12H), 6.85 – 6.79 (m, 2H), 4.78 (d, *J* = 11.3 Hz, 1H), 4.66 (s, 2H), 4.58 (d, *J* = 11.4 Hz, 1H), 4.50 (d, *J*=11.3, 1H), 4.47 (d, *J*=11.4, 1H), 4.25 (dd, *J* = 11.5, 2.8 Hz, 1H), 4.18 – 3.98 (m, 3H), 3.95 – 3.88 (m, 2H), 3.85 – 3.80 (m, 1H), 3.78 (s, 3H), 3.71 (dd, *J* = 7.3, 4.1 Hz, 1H), 3.10 (s (br.), 1H), 2.03 (s, 3H), 1.16 – 1.00 (m, 21H).

^{13}C-NMR (101 MHz, $CDCl_3$): δ (ppm) = 171.4, 159.3, 138.3, 138.1, 130.6, 129.7, 128.5, 128.13, 128.06, 127.9, 113.9, 80.3, 78.6, 78.4, 73.8, 73.7, 72.9, 70.4, 66.3, 63.6, 55.4, 21.1, 18.2, 12.1.

IR (film) ν_{max} (cm^{-1}): 3467, 2945, 2866, 2358, 2312, 1739, 1514, 1454, 1248, 1093, 1047, 1008, 883, 800, 735, 696.

ESI-HRMS: calc. for $[C_{39}H_{56}O_8Si+Na]^+$ 703.3642, found 703.3638.

Regioselective Pivaloyl Protection (S6)

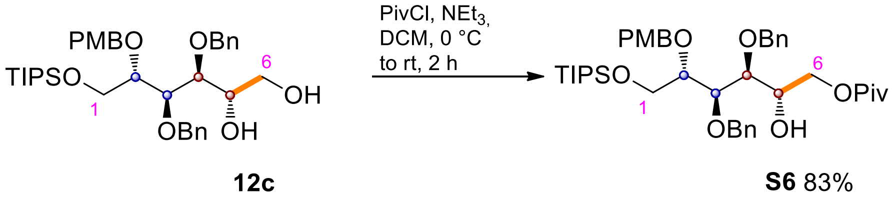

To a solution of **12c** (128 mg, 0.2 mmol, 1.0 eq.) and PivCl (0.037 mL, 0.3 mmol, 1.5 eq.) in CH_2Cl_2 (1.5 mL), NEt_3 (0.055 mL, 0.4 mmol, 2.0 eq.) was added under an atmosphere of argon at 0 °C. The mixture was stirred for 17 h. The solvents were removed *in vacuo* and the residue was purified by flash chromatography (SiO_2, CyHex/EtOAc, 9:1, $KMnO_4$) yielding **S6** (120 mg, 0.166 mmol, 83%) as a colourless oil.

R_f (EA/CH 1:9) = 0.14.

$[\alpha]_D^{20}$= -1.7 (0.027, $CHCl_3$).

^{1}H NMR (400 MHz, $CDCl_3$): δ (ppm) = 7.35 – 7.19 (m, 12H), 6.87 – 6.78 (m, 2H), 4.80 (d, *J* = 11.3 Hz, 1H), 4.68 (s, 2H), 4.56 (d, *J* = 11.4 Hz, 1H), 4.50 (d, *J*=11.2, 1H), 4.47 (d, *J*=11.2, 1H), 4.30 (dd, *J* = 11.6, 3.0 Hz, 1H), 4.18 (dd, *J* = 11.7, 5.6 Hz, 1H), 4.09 (dd, *J* = 10.8, 3.3 Hz, 1H), 4.06 – 4.00 (m, 1H), 3.95 – 3.89 (m, 2H), 3.85 – 3.73 (m, 5H), 2.92 (s (br.), 1H), 1.19 (s, 9H), 1.09 – 1.03 (m, 21H).

^{13}C NMR (101 MHz, $CDCl_3$): δ (ppm) = 178.9, 159.2, 138.4, 138.3, 130.7, 129.5, 128.5, 128.2, 127.9, 127.85, 127.80, 113.9, 80.3, 78.6, 78.3, 73.9, 73.8, 72.7, 70.4, 66.1, 63.7, 55.4, 39.0, 27.4, 18.2, 12.1.

IR (film) ν_{max} (cm^{-1}): 3527, 2943, 2866, 1732, 1614, 1541, 1514, 1458, 1284, 1248, 1159, 1093, 1068, 1035, 883, 735, 696.

ESI-HRMS: calc. for $[C_{42}H_{62}O_8Si+Na]^+$ 745.4112, found 745.4111.

Inversion by Epoxidation (20)

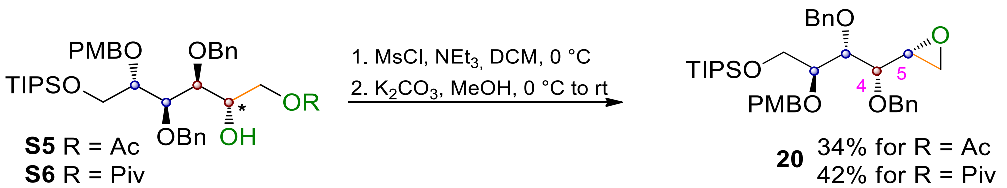

S5 R = Ac
S6 R = Piv

20 34% for R = Ac
42% for R = Piv

To a solution of **S5** (132 mg, 0.19 mmol, 1.0 eq.), NEt_3 (0.052 mL, 0.38 mmol, 2.0 eq.) and DMAP (2 mg, 10 mol%) in CH_2Cl_2 (2 mL), MsCl (0.029 mL, 0.38 mmol, 2.0 eq.) were added under argon at 0 °C. After stirring for 2 h, water was added and the aqueous phase was extracted with Et_2O (3 x 10 mL). The organic phase was dried over $MgSO_4$ and the solvent was removed *in vacuo*. The residue was redissolved in MeOH (3 mL) and K_2CO_3 (52 mg, 0.38 mmol, 2.0 eq.) was added. After stirring for 2 h, water was added, and the aqueous phase was extracted with EtOAc (3 x 10 mL). The organic phase was dried over $MgSO_4$ and the solvent was removed *in vacuo*. The residue was purified by flash chromatography (SiO_2, CyHex/EtOAc, 9:1, $KMnO_4$) yielding **20** (40 mg, 0.064 mmol, 34%) as a colourless oil. When the reaction was performed with **S6** on a 0.23 mmol scale, the yield was 42%.

R_f (EA/CH 1:9) = 0.32.

$[\alpha]_D^{20}$= +22.5 (0.002, $CHCl_3$).

^{1}H NMR (400 MHz, $CDCl_3$): δ (ppm) = 7.38 – 7.22 (m, 10H), 7.14 – 7.06 (m, 2H), 6.79 (d, *J* = 8.6 Hz, 2H), 4.86 (d, *J* = 11.9 Hz, 1H), 4.74 (d, *J* = 11.6 Hz, 1H), 4.64 (d, *J* = 11.0, 1H), 4.61 (d, *J* = 11.6, 1H), 4.54 (d, *J* = 11.9 Hz, 1H), 4.31 (d, *J* = 11.0 Hz, 1H), 4.13 – 4.06 (m, 1H), 3.93 – 3.75 (m, 5H), 3.71 (dd, *J* = 6.5, 3.3 Hz, 1H), 3.30 (dd, *J* = 7.1, 3.3 Hz, 1H), 2.49 – 2.39 (m, 2H), 1.13 – 1.00 (m, 21H).

^{13}C NMR (101 MHz, $CDCl_3$): δ (ppm) = 159.1, 138.6, 138.4, 130.8, 129.6, 128.5, 128.4, 128.1, 127.9, 127.8, 127.6, 113.8, 80.7, 79.8, 79.6, 74.2, 72.8, 72.2, 63.4, 55.4, 54.0, 43.1, 18.2, 18.1, 12.12, 12.06.

IR (film) ν_{max} (cm^{-1}): 2945, 2866, 2360, 1514, 1456, 1248, 1097, 912, 739.

ESI-HRMS: calc. for $[C_{37}H_{52}O_6Si+Na]^+$ 643.3431, found 643.3434.

Attempts at Opening Epoxide 20 (S7)*

*The following procedure by Schmidt and co-workers[6] for epoxide hydrolysis was tested several times in order to obtain **S7a** (i.e. the C^5 epimer to **12c**). The reactions lead to PMB-cleavage among numerous other side reactions. One attempt at epoxide opening with an alkoxide proved slightly more promising **S7b**. No further attempts at epoxide opening were undertaken, as **20** should constitute an excellent precursor for carbohydrate synthesis by thiolate opening and Pummerer-reaction along the lines of the Sharpless carbohydrate synthesis.[7]*

Attempted Hydrolysis of Epoxide 20

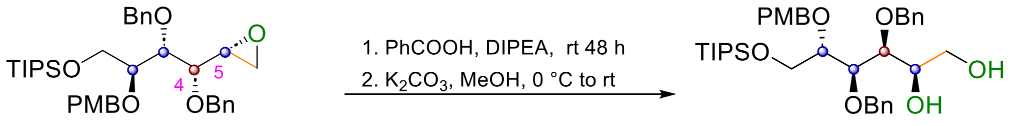

20 **S7a** < 9% (2 steps)

To a solution of **20** (52 mg, 0.084 mmol, 1.0 eq.) in DIPEA (0.029 mL, 2.0 eq.) was added benzoic acid (41 mg, 0.34 mmol, 4.0 eq.) and the mixture was stirred for 48 h. The residue was diluted with DCM (5 mL), quenched by addition of H_2O (5 mL) and extracted with Et_2O (2 x 5 mL). The combined organic phases were washed with $NaHCO_3$ (sat. aq., 2 x 5 mL) and dried over $MgSO_4$. The solvent was removed *in vacuo* and the residue was purified by flash chromatography (SiO_2, CyHex/EtOAc, 9:1, $KMnO_4$) yielding the benzoate (6 mg, 0.008 mmol, 9%.) as a colourless oil. This was again dissolved in MeOH (2 mL) and K_2CO_3 (23 mg, 0.168, 2.0 eq.) was added. The solvent was removed *in vacuo*, diluted with Et_2O (5 mL) and washed with H_2O (3 x 5 mL). The organic phase was dried over $MgSO_4$ and the solvents were removed *in vacuo* yielding a contaminated sample of product **S7a** (8 mg, quant.).

$\mathbf{R_f}$ (EA/CH 1:3) = 0.15.

^{1}H NMR (400 MHz, $CDCl_3$): δ (ppm) = 7.38 – 7.19 (m, 12H), 6.91 – 6.80 (m, 2H), 4.79 – 4.43 (m, 6H), 4.04 (dd, J = 9.7, 4.6 Hz, 1H), 4.01 – 3.46 (m, 12H), 1.11 – 1.02 (m, 21H).

IR (film) ν_{max} (cm^{-1}): 3446, 2941, 2866, 1514, 1456, 1248, 1064, 696.

ESI-HRMS: calc. for $[C_{37}H_{54}O_7Si+Na]^+$ 661.3536, found 661.3534.

Epoxide Opening with NaOAllyl (S7b)

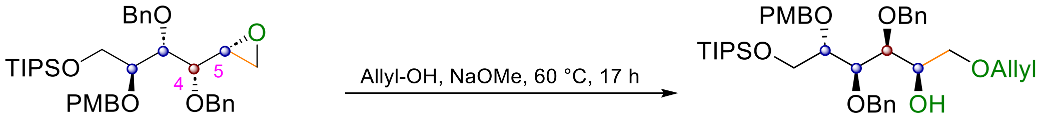

20 **S7b** 15 %

To a solution of **20** (40 mg, 0.06 mmol, 1.0 eq.) in allylic alcohol (2 mL) was added NaOMe (50 mg) and heated to 60 °C for 17 h. The mixture was quenched with water, extracted with Et_2O (2 x 5 mL) and dried over $MgSO_4$. The solvent was removed *in vacuo* and the residue was purified by flash chromatography (SiO_2, CyHex/EtOAc, 9:1, $KMnO_4$) yielding **S7b** (6 mg, 0.009 mmol, 15%.) as a colourless oil.

[6] B. Schmidt, O. Kunz, A. Biernat, *J. Org. Chem.* **2010**, *75*, 2389–2394.

[7] (a) S. Y. Ko, A. W. M. Lee, S. Masamune, L. A. Reed, K. Barry Sharpless, F. J. Walker, *Tetrahedron* **1990**, *46*, 245–264; (b) X. Yu, G. O'Doherty, *ACS Symp. Ser.* **2008**, 3–28; (c) A. Z. Aljahdali, P. Shi, Y. Zhong, G. A. O'Doherty, *Adv. Carbohydr. Chem. Biochem.* **2013**, 55–123.

R_f (EA/CH 1:9) = 0.26.

^{1}H NMR (400 MHz, $CDCl_3$): δ (ppm) = 7.34 – 7.22 (m, 12H), 6.89 – 6.80 (m, 2H), 5.93 – 5.78 (m, 1H), 5.28 – 5.11 (m, 2H), 4.79 – 4.50 (m, 6H), 4.08 – 3.76 (m, 11H), 3.72 (dd, *J*=8.2, 5.4, 1H), 3.50 – 3.32 (m, 2H), 1.17 – 1.00 (m, 21H).

IR (film) v_{max} (cm^{-1}): 2941, 2866, 1514, 1458, 1248, 1066, 1037, 881, 683.

ESI-HRMS: calc. for $[C_{40}H_{58}O_7Si+Na]^+$ 701.3850, found 701.3844.

Conversion into Aldohexoses

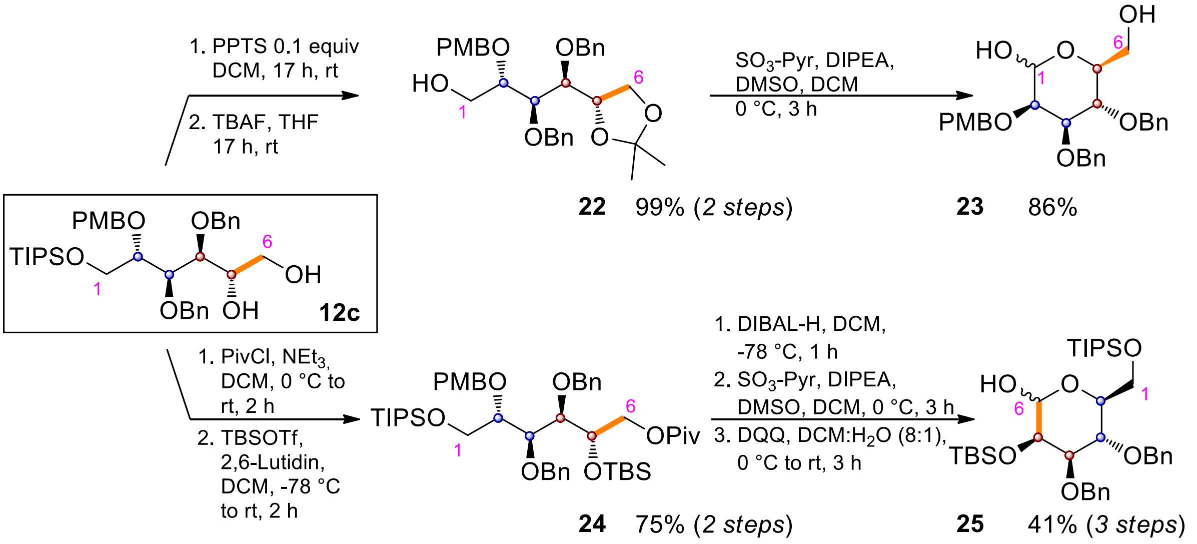

Scheme VI Overview of the conversion of mannitol 12c into aldohexoses.

Cyclisation *via* C1 - Acetal Protection of 12c (S8)

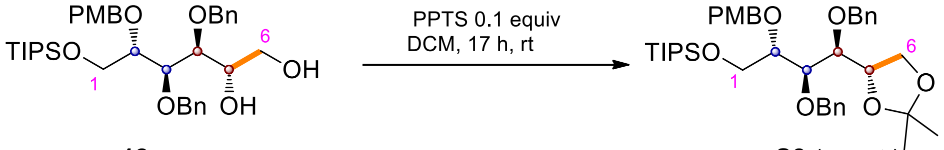

12c **S8** (quant.)

To a solution of **12c** (100 mg, 0.16 mmol, 1.0 eq.) under argon in CH_2Cl_2 (0.7 mL) and acetone (0.1 mL) PPTS (1 mg)* was added and the mixture was stirred for 17 h. The solvents were removed *in vacuo*. The residue was purified by flash chromatography (SiO_2, CyHex/EtOAc, 9:1, $KMnO_4$) yielding **S8** (108 mg, 0.16 mmol, quant.) as a colourless oil.

R_f (EA/CH 1:9) = 0.33.

$[\alpha]_D^{20}$= -3.0 (0.003, $CHCl_3$).

^{1}H NMR (400 MHz, $CDCl_3$): δ (ppm) = 7.41 – 7.12 (m, 12H), 6.88 – 6.78 (m, 2H), 4.77 – 4.55 (m, 5H), 4.40 (d, *J* = 11.2 Hz, 1H), 4.26-4.24 (m, 1H), 4.11 (dd, *J* = 11.0, 2.3 Hz, 1H), 3.96 (dd, *J* = 5.4, 2.8 Hz, 1H), 3.92 – 3.86 (m, 3H), 3.83 – 3.75 (m, 5H), 1.39 (s, 3H), 1.30 (s, 3H), 1.15 – 1.02 (m, 21H).

^{13}C NMR (101 MHz, $CDCl_3$): δ (ppm) = 159.2, 138.9, 138.6, 131.0, 129.3, 128.41, 128.36, 128.0, 127.8, 127.7, 127.6, 113.8, 108.5, 79.9, 79.4, 79.2, 76.8, 74.4, 74.3, 72.2, 66.5, 63.5, 55.4, 26.7, 25.3, 18.2, 12.1.

IR (film) v_{max} (cm^{-1}): 2943, 2866, 1514, 1456, 1248, 1095, 1068, 696.

ESI-HRMS: calc. for $[C_{40}H_{58}O_7Si+Na]^+$ 701.3850, found 701.3848.

***Comment:** The use of *p*-TSA or larger amounts of PPTS (1 quiv.) lead to PMB cleavage.

Cyclisation *via* C¹ - Desilylation (22)

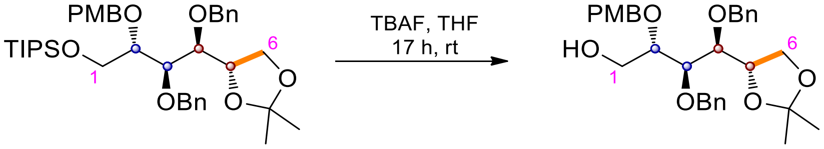

S8 **22** (quant.)

To a solution of **S8** (59 mg, 0.08 mmol, 1.0 eq.) in THF (2 mL), TBAF (1 M in THF, 0.12 mL, 0.12 mmol, 1.4 eq.) was added at 0 °C and the mixture was stirred for 17 h. The solvents were removed. The residue was purified by flash chromatography (SiO_2, CyHex/EtOAc, 4:1, $KMnO_4$) yielding **22** (44 mg, 0.08 mmol, quant.) as a colourless oil.

$\mathbf{R_f}$ (EA/CH 1:4) = 0.03.

$[\alpha]_D^{20}$= -18.0 (0.00067, $CHCl_3$).

^{1}**H NMR** (400 MHz, $CDCl_3$): 7.40 – 7.18 (m, 12H), 6.91 – 6.84 (m, 2H), 4.79 – 4.66 (m, 3H), 4.59 (d, *J* = 11.6 Hz, 1H), 4.52 (d, *J* = 11.1 Hz, 1H), 4.37 (d, *J* = 11.1 Hz, 1H), 4.24 (ddd, *J* = 6.9, 6.3, 5.6 Hz, 1H), 4.02 – 3.87 (m, 4H), 3.86 – 3.68 (m, 6H), 1.42 (s, 3H), 1.31 (s, 3H).

^{13}**C NMR** (101 MHz, $CDCl_3$): δ (ppm) = 159.5, 138.5, 138.2, 130.2, 129.5, 128.5, 128.5, 128.3, 127.93, 127.90, 127.8, 114.1, 108.6, 79.4, 79.1, 78.7, 76.7, 74.7, 74.4, 71.3, 66.7, 60.1, 55.4, 26.7, 25.3.

IR (film) v_{max} (cm^{-1}): 2981, 2869, 2360, 1516, 1456, 1250, 1070.

ESI-HRMS: calc. for $[C_{31}H_{38}O_7+Na]^+$ 545.2515, found 545.2519.

Cyclisation *via* C¹ - Parikh-Doering Oxidation to Aldohexose 23

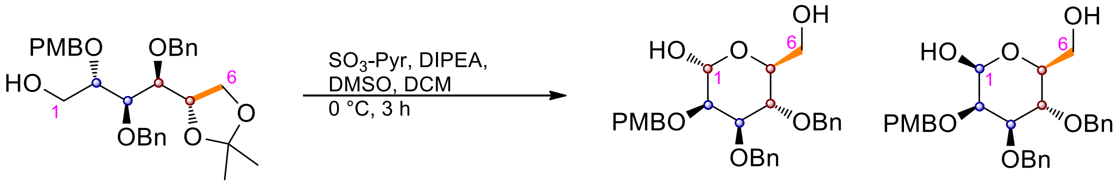

22 **23** 86% d.r. **2 : 1**

To a solution of **22** (50 mg, 0.096 mmol, 1.0 eq.) in CH_2Cl_2 (2 mL), DIPEA (0.05 mL, 0.32 mmol, 3.3 eq.), DMSO (0.05 mL, 0.67 mmol, 7.0 eq.) and SO_3Pyr complex (51 mg, 0.32 mmol, 3.3 eq.) were added sequentially at 0 °C and the mixture was stirred at 0 °C for 1 h. After TLC control, cold HCl (aq. 1 N, 5 mL) was added and the phases were separated. The aqueous phase was extracted with Et_2O (3 x 10 mL). The organic phase was dried over $MgSO_4$ and the solvent was removed *in vacuo*. The residue was purified by flash chromatography (SiO_2, CyHex/EtOAc, 2:1, $KMnO_4$) yielding **23** (40 mg, 0.08 mmol, 86%, α:β = 2:1) as a colourless oil.

$\mathbf{R_f}$ (EA/CH 1:2) = 0.08.

$[\alpha]_D^{20}$= -5.25 (0.001, $CHCl_3$).

^{1}**H NMR** (400 MHz, $CDCl_3$): δ (ppm) = 7.40 – 7.26 (m, 12H + 12H, CH), 6.95 – 6.87 (m, 2H, CH), 6.93 – 6.78 (m, 2H, CH), 5.18 (d, *J* = 1.8 Hz, 1H, CHO$_2$), 5.04 (d, *J* = 11.4 Hz, 1H, CH$_2$Ar), 4.95 (d, *J* = 10.9 Hz, 1H, CH$_2$Ar), 4.92 (d, *J* = 11.0 Hz, 1H, CH$_2$Ar), 4.80 – 4.54 (m, 5H, CH$_2$Ar, 4H, CH$_2$Ar, 1H, CHO$_2$), 3.97 –

3.62 (m, 9H, 7H), 3.63 (dd, J = 9.5, 2.8 Hz, 1H), 3.33 (ddd, J = 9.5, 4.8, 2.8 Hz, 1H), 2.72 (s (br.), 2H, 2H).

^{13}C NMR (101 MHz, $CDCl_3$): δ (ppm) = 159.7 (C^{ipso}), 159.4 (C^{ipso}), 138.6 (C^{ipso}), 138. 5 (C^{ipso}), 138.2 (C^{ipso}), 138.1 (C^{ipso}), 130.3 (CH), 130.12 (CH), 130.09 (CH), 129.8 (CH), 128.7 (CH), 128.6 (CH), 128.54 (CH), 128.51 (CH), 128.2 (CH), 128.04 (CH), 128.01 (CH), 127.9 (CH), 127.79 (CH), 127.75 (CH), 127.7 (CH), 114.2 (CH), 113.9 (CH), 93.8 (CHO_2), 93.1 (CHO_2), 83.1 (CHOH), 79.8 (CHOH), 76.1 (CHOH), 75.7 (CHOH), 75.33, 75.29, 74.7, 74.5, 73.0, 72.8, 72.37, 72.34 (4x CH_2Ar, 4x CHOH), 62.6 (CH_2OH), 62.1 (CH_2OH), 55.40 (OCH_3), 55.38 (OCH_3).

IR (film) ν_{max} (cm^{-1}): 3446, 2869, 1512, 1248, 1086, 1028, 735, 696.

ESI-HRMS: calc. for $[C_{28}H_{32}O_7Na]^+$ 503.2046, found 503.2042.

Cyclisation *via* C6 –Protection of the Secondary Hydroxyl Group in S6 (24)

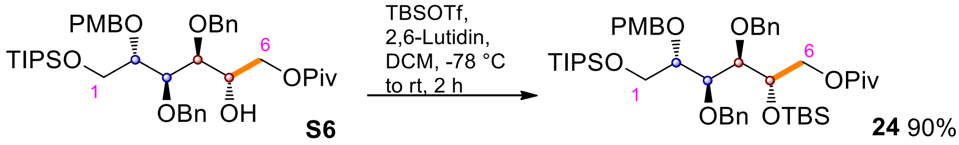

To a solution of **S6** (110 mg, 0.15 mmol, 1.0 eq.) and 2,6-lutidine (0.05 mL, 0.45 mmol, 3.0 eq.) in CH_2Cl_2 (2 mL), TBSOTf (0.075 mL, 0.33 mmol, 2.2 eq.) was added under an atmosphere of argon at -78 °C. The mixture was allowed to warm up to rt over 17 h. Water (5 mL) was added, and the aqueous phase was extracted with CH_2Cl_2 (3 x 5 mL). The organic phase was dried over $MgSO_4$ and the solvent was removed *in vacuo*. The residue was purified by flash chromatography (SiO_2, CyHex/EtOAc, 20:1, $KMnO_4$) yielding **24** (113 mg, 0.135 mmol, 90%.) as a colourless oil.

R_f (EA/CH 1:9) = 0.55.

$[\alpha]_D^{20}$= -2.25 (0.001, $CHCl_3$).

^{1}H NMR (400 MHz, $CDCl_3$): δ (ppm) = 7.36 – 7.19 (m, 12H), 6.86 – 6.82 (m, 2H), 4.83 – 4.69 (m, 4H), 4.59 (d, J = 11.4 Hz, 1H), 4.46 (d, J = 11.4 Hz, 1H), 4.39 (dd, J = 12.0, 2.5 Hz, 1H), 4.26 (dd, J = 12.0, 5.6 Hz, 1H), 4.15 - 4.12 (m, 1H), 4.09 (dd, J = 11.1, 2.9 Hz, 1H), 3.96 (dd, J = 11.0, 5.8 Hz, 1H), 3.92 – 3.88 (m, 2H), 3.83 -4.79 (m, 4H), 1.23 (s, 9H), 1.12 – 1.01 (m, 21H), 0.92 (s, 9H), 0.07 (s, 3H), 0.06 (s, 3H).

^{13}C NMR (101 MHz, $CDCl_3$): δ (ppm) = 178.5, 159.1, 138.9, 138.8, 131.2, 129.1, 128.4, 127.8, 127.6, 127.53, 127.47, 113.7, 81.9, 80.3, 78.8, 74.6, 73.8, 72.3, 72.2, 66.7, 63.8, 55.4, 38.9, 27.5, 27.1, 26.0, 18.22, 18.15, 12.1, 12.0, -4.32.

IR (film) ν_{max} (cm^{-1}): 3751, 2958, 2858, 2358, 1734, 1683, 1653, 1647, 1635, 1616, 1558, 1541, 1516, 1506, 1471, 1458, 1250, 1111, 835, 740, 669.

ESI-HRMS: calc. for $[C_{48}H_{76}O_8Si_2+Na]^+$ 859.4976, found 859.4975.

Cyclisation *via* C6 –Liberation of the Primary Hydroxyl Group (S9)

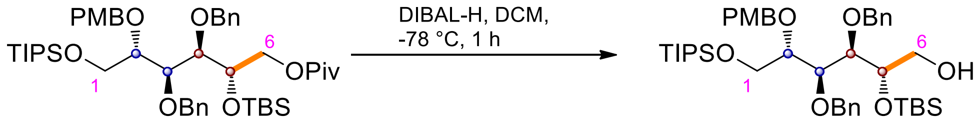

24 **S9** 96%

To a solution of **24*** (100 mg, 0.12 mmol, 1.0 eq.) in CH_2Cl_2 (2 mL), DIBAL-H (1 M in hexane, 0.3 mL, 0.3 mmol, 2.5 eq.) was added under argon at -78 °C. The mixture was allowed to warm up to rt over 17 h. MeOH (1 mL) was added and the reaction mixture was transferred onto a saturated aq. solution of Rochelle's salt (10 mL) at 0 °C. After stirring for 30 min, the aqueous phase was extracted with EtOAc (3 x 5 mL). The organic phase was dried over $MgSO_4$ and the solvent was removed *in vacuo*. The residue was purified by flash chromatography (SiO_2, CyHex/EtOAc, 9:1, $KMnO_4$) yielding **S9** (87 mg, 0.115 mmol, 96%.) as a colourless oil.

R$_f$ (EA/CH 1:9) = 0.05.

[α]$_D^{20}$= +3.9 (0.004, $CHCl_3$).

^{1}H NMR (400 MHz, $CDCl_3$): δ (ppm) = 7.35 – 7.16 (m, 12H), 6.86 – 6.80 (m, 2H), 4.78 – 4.60 (m, 5H), 4.39 (d, *J* = 11.2 Hz, 1H), 4.08 (dd, *J* = 11.0, 2.9 Hz, 1H), 4.00 – 3.87 (m, 2H), 3.86 – 3.79 (m, 5H), 3.74 – 3.66 (m, 3H), 1.57 (s (br.), 1H), 1.12 – 1.03 (m, 21H), 0.90 (s, 9H), 0.08 – -0.00 (m, 6H).

^{13}C NMR (101 MHz, $CDCl_3$): δ (ppm) = 159.3, 139.0, 138.8, 130.7, 129.5, 128.44, 128.38, 128.0, 127.8, 127.6, 127.5, 113.9, 81.9, 80.7, 78.1, 74.8, 74.3, 73.2, 72.4, 63.7, 63.6, 55.4, 26.1, 18.2, 12.1, -4.36, -4.42.

IR (film) ν_{max} (cm^{-1}): 3751, 2958, 2858, 2358, 2312, 1653, 1558, 1541, 1514, 1458, 1251, 1099, 837, 737, 669.

ESI-HRMS: calc. for $[C_{43}H_{68}O_7Si_2+Na]^+$ 775.4401, found 775.4396.

***Comment:** We explored different protecting groups on **24**. Using OAc instead of OPiv led to partial TBS-migration upon deacylation (K_2CO_3, MeOH, 0 °C to rt, 2h). Attempts to selectively desilylate a disiyilylated derivative selectively (OTBS instead of OPiv) with PTSA, PPTS or CSA were unsuccessful.

Cyclisation *via* C6 –Synthesis of Aldohexose 25

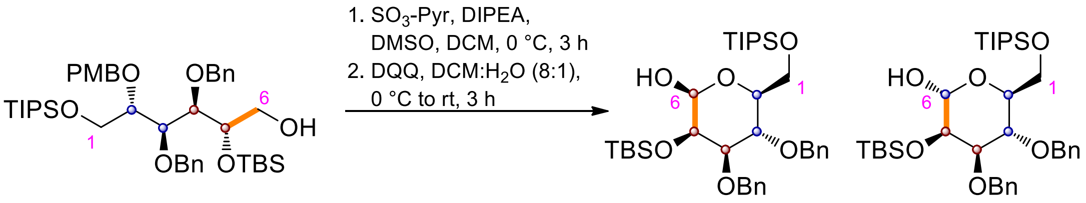

S9 **25** 52% (*2 steps*) d.r. **6 : 4**

To a solution of **S9** (100 mg, 0.12 mmol, 1.0 eq.) in CH_2Cl_2 (2 mL), DIPEA (0.05 mL, 0.28 mmol, 3.3 eq.), DMSO (0.042 mL, 0.59 mmol, 7.0 eq.) and SO_3Pyr complex (45 mg, 0.28 mmol, 3.3 eq.) were added sequentially at 0 °C. The mixture was stirred at 0 °C for 1 h. After TLC control indicated complete conversion, cold HCl (aq. 1 N, 5 mL) was added and the phases were separated. The aqueous phase was extracted with Et_2O (3 x 10 mL). The organic phase was dried over $MgSO_4$ and the solvent was removed *in vacuo*. The residue was dissolved in CH_2Cl_2 (4.5 mL) and PBS buffer (pH = 7.4, 0.5 mL). DDQ (29 mg, 0.13 mmol, 1.5 eq.) was added at 0 °C. The mixture was stirred for 17 h. $NaHCO_3$ (5 mL) was added, and the aqueous phase was extracted with EtOAc (3 x 5 mL). The organic phase was dried

over $MgSO_4$ and the solvent was removed *in vacuo*. The residue was purified by flash chromatography (SiO_2, CyHex/EtOAc, 9:1, $KMnO_4$) yielding **25**(28 mg, 0.044 mmol, 52%, α:β = 6:4) as a colourless oil.

R_f (EA/CH 1:9) = 0.17.

$[\alpha]_D^{20}$= -5.1 (0.021, $CHCl_3$).

^{1}H NMR (400 MHz, $CDCl_3$): δ (ppm) = 7.40 – 7.19 (m, 10H, 10H, CH), 5.05 (d, *J* = 2.0 Hz, 1H, CHO_2), 4.84 (d, *J* = 10.9 Hz, 1H, CH_2Ar), 4.83 (d, *J* = 10.8 Hz, 1H, CH_2Ar), 4.76 – 4.59 (m, 3H, CH_2Ar, 3H, CH_2Ar, 1H, CHO_2), 4.08 – 3.75 (m, 6H, 4H), 3.46 (dd, *J* = 9.5, 2.7 Hz, 1H, CHO), 3.37 (d (br.), *J* = 12.5 Hz, 1H, OH), 3.25 (ddd, *J* = 9.1, 3.3, 2.1 Hz, 1H, CHO), 2.58 (s (br.), 1H), 1.15 – 0.98 (m, 21H, 21H), 0.88 (d, *J* = 11.7 Hz, 9H, 9H), 0.14 – -0.00 (m, 6H, 6H).
^{13}C NMR (101 MHz, $CDCl_3$): δ (ppm) = 138.9 (C^{ipso}), 138.7 (C^{ipso}), 138.2 (C^{ipso}), 128.5 (CH), 128.44 (CH), 128.43 (CH), 128.3 (CH), 128.2 (CH), 128.1 (CH), 128.0 (CH), 127.8 (CH), 127.7 (CH), 127.6 (CH), 127.4 (CH), 95.6 (CHO_2), 94.5 (CHO_2), 82.2 (CHOH), 79.9 (CHOH), 76.4 (CHOH), 75.0, 74.9, 74.4, 73.9, 73.8 73.5, 72.5, 72.2 (4x CH_2Ph and 4x CHOH), 70.3 (CHOH), 63.2 (CH_2OH), 62.6 (CH_2OH), 26.2 (CCH_3), 25.9 (CCH_3), 18.6 (CCH_3), 18.3 (CCH_3), 18.21 ($CHCH_3$), 18.18 ($CHCH_3$), 12.1 ($CHCH_3$), -3.9 ($SiCH_3$), -4.4 ($SiCH_3$), -4.8 ($SiCH_3$), -4.9 ($SiCH_3$).

IR (film) ν_{max} (cm^{-1}): 2945, 2866, 2360, 1699, 1558, 1541, 1521, 1508, 1456, 1111.

ESI-HRMS: calc. for $[C_{35}H_{58}O_6Si_2+Na]^+$ 653.3670, found 653.3668.

Vinylmetalspecies from CH_2X_2

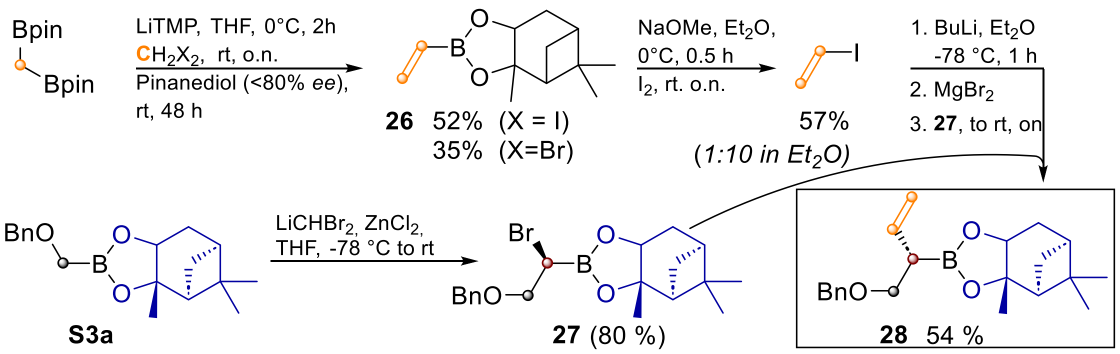

Pinanediol (vinyl)boronate (26)

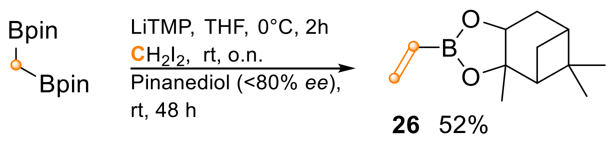

Immediately before the reaction, LiTMP was prepared in a separate Schlenk tube by adding a solution of *n*-BuLi (2.5 M in hexanes, 2.94 mL, 7.34 mmol, 1.05 eq.) in hexanes to a solution of dry TMP (1.3 mL, 7.7 mmol, 1.1 eq.) in dry THF (13 mL) at 0 °C. The LiTMP solution was stirred for 20 min. at this temperature and added dropwise under ice bath cooling (0 °C) to a solution of $CH_2(Bpin)_2$ (1.868 g, 6.97 mmol, 1.0 eq.) in dry THF (45 mL). After the addition was complete the reaction mixture was stirred for 2 h at 0 °C before CH_2I_2 (1.18 mL, 14.68 mmol, 2.1 eq.) was added dropwise. The reaction mixture was allowed to slowly warm to rt. overnight, while being stirred. Afterwards, pinanediol with an *ee.* <80 %* (1.872 g, 11.0 mmol, 1.6 eq.) was added and stirring at rt.

was continued for another 48 h. Then the mixture was poured onto saturated aq. NH_4Cl. The aqueous layer was extracted twice with Et_2O (10 mL) and the combined organic layers were washed with saturated aq. NaCl, dried over $MgSO_4$, filtered and concentrated *in vacuo*. The residue was purified by column chromatography (silica gel; CyHex/EtOAc, 99:1) yielding **26** (743 mg, 3.61 mmol, 52 %) as a colorless oil.

R_f= 0.17 (silica gel; CyHex/EtOAc, 99:1).

^{1}H NMR(400 MHz, $CDCl_3$) δ 6.16 (dd, *J* = 19.4, 4.2 Hz, 1H), 6.03 (dd, *J* = 13.5, 3.7 Hz,1H), 5.89 (dd, *J* = 19.4, 13.7 Hz, 1H), 4.32 (dd, *J* = 8.8, 1.7 Hz, 1H), 2.40 – 2.31 (m, 1H), 2.26 – 2.19 (m, 1H), 2.07 (t, *J* = 5.5 Hz, 1H), 1.95 – 1.85 (m, 2H), 1.42 (s, 3H), 1.29 (s,3H), 1.15 (d, *J* = 10.9 Hz, 1H), 0.85 (s, 3H) ppm.

^{13}C NMR (100 MHz, $CDCl_3$) δ 137.2, 85.9, 77.9, 51.5, 39.6, 38.3, 35.6, 28.8, 27.2, 26.6, 24.2 ppm.

The carbon atom attached to boron was reported at 53.6 ppm but not observed here. The rest of the spectroscopic data were consistent with those reported in the literature.[8]

***Comment:** Transesterification to Pinanediol facilitates purification by column chromatography, by raising the boiling point of the boronic ester. Thus, cheap material of low *ee.* can be employed. Pinanediol of high *ee.* is generated by recrystallizing the bishydroxylation product of pinene (80% *ee.*) from heptane. "Leftovers" from such recrystallizations (isolated from the mother liquor) can be put to good use here.

Vinyl iodide

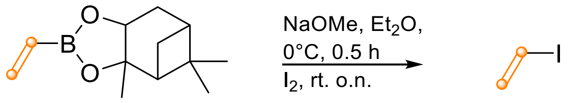

26 57% (*1:10 in Et_2O*)

NaOMe (765 mg, 14 mmol, 2.2 eq.) was dried in a Schlenk flask under *vacuum* by heating. The solid was cooled to rt. and dissolved in dry Et_2O (7 mL). The solution was cooled to 0°C. Boronate **26** (1.292 g, 6.27 mmol, 1.0 eq.) was added dropwise, after which the mixture was stirred for 0.5 h at this temperature. Afterwards, iodine (3.988 g, 15.7 mmol, 2.5 eq.) was added and the mixture was allowed to warm up to rt. overnight. Dried* $Na_2S_2O_3$ (2.012 g, 12.7 mmol, 2.0 eq.) was added to quench the remaining iodine. Distillation of the residue (80 °C under 1 atm. of argon) delivered vinyl iodide as a 1:9.6 mixture with Et_2O (552 mg, 3.58 mmol, 57 %).

^{1}H NMR (400 MHz, $CDCl_3$) δ 6.59-6.50 (m, 2H), 6.27 (d, *J* = 14.9 Hz, 1H) ppm.

^{13}C NMR(100 MHz, $CDCl_3$)δ 130.7 (s), 85.4 (s) ppm.

Spectroscopic data were consistent with those reported in the literature.[9]

***Comment:** Dried under vacuum over P_2O_5.

[8] P. Dominguez-Molano, G. Bru, O. Salvado, R. J. Maza, J. J. Carbó, E. Fernández, *Chem. Commun.*, **2021**, *57*, 13361

[9] (a) R.E. Mayo, J.H. Goldstein, *J.Mol. Spectrosc.*, **1964**, *14*, 173; (b) [3] J. Stothers, *Carbon-13 NMR Spectroscopy: Organic Chemistry, A Series of Monographs*, Elsevier, **2012**, *24*, 184.

Model Matteson-Homologation Product 27

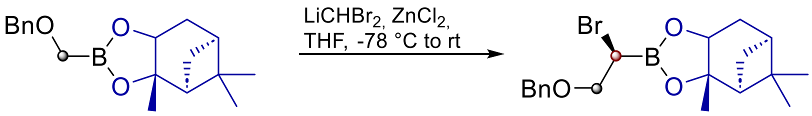

S3a **27** (80 %)

A solution of LDA was freshly prepared in a Schlenk tube by adding *n*-BuLi (2.5 M solution in hexanes, 1.04 mL, 2.6 mmol, 1.3 eq.) to dry diisopropylamine (0.45 mL, 3.2 mmol, 1.6 eq.) in dry THF (6.5 mL) at –78 °C and stirring for 30 minutes at this temperature. The resulting LDA solution was added dropwise to a mixture of benzyloxymethyl boronate **S3a** (300 mg, 1.0 mmol, 1.0 eq.) and CH_2Br_2 (1.40 mL, 20 mmol, 10 eq.) in dry THF (13,5 mL) at – 78 °C. After stirring for 1 h at this temperature, a solution of $ZnCl_2$ in Et_2O (1 M, 8.0 mL, 8.0 mmol, 4.0 eq.) was added at –78 °C and the reaction mixture was allowed to slowly warm up to rt. overnight. The reaction mixture was diluted with CyHex (20 mL), washed with saturated aq. NH_4Cl and the aqueous layer was reextracted twice with a mixture of CyHex/Et_2O = 4:1. The combined organic phases were dried with Na_2SO_4, filtered, and concentrated *in vacuo*, yielding crude α-bromoboronate **27** containing 20% of **S3a** (623 mg, 1.6 mmol,80 %, *de* >95 %*, orange oil).

R_f = 0.11 (silica gel; CyHex/EtOAc, 49:1).

^{1}H NMR (400 MHz, $CDCl_3$): δ = 7.39 – 7.26 (m, 5 H), 4.60 (s, 2 H), 4.36 (dd, *J* = 8.8, 1.7 Hz, 1 H), 3.91 – 3.75 (m, 2 H), 3.53 (dd, *J* = 8.1, 6.4 Hz, 1 H), 2.39 – 2.30 (m, 1 H), 2.27 –2.18 (m, 1 H), 2.12 – 2.05 (m, 1 H), 1.95 – 1.85 (m, 2 H), 1.41 (s, 3 H), 1.29 (s, 3 H), 1.26 (d, *J* = 11.1 Hz, 1 H), 0.84 (s, 3 H) ppm.

^{13}C NMR (100 MHz, $CDCl_3$): δ = 138.2, 128.5, 127.8, 127.7, 86.9, 78.7, 73.2, 71.6, 51.4, 39.4, 38.4, 35.3, 28.5, 27.2, 26.4, 24.1 ppm, *carbon attached to boron not observed.*

NMR data was consistent with that reported in literature.[10]

Comment: The *de* was determined as described by D. Matteson from the ^{13}C NMR spectrum by integrating the signals at 86.9 and 87.0 ppm.[10]

---

[10] D. S. Matteson, M. L. Peterson, J. Org. Chem. **1987**, *52*, 5116–5121.

(3aS,4S,6S)-2-[(S)-1-(Benzyloxy)but-3-en-2-yl]-3a,5,5-trimethylhexahydro-4,6-metha- nobenzo[d][1,3,2]dioxaborole (6)

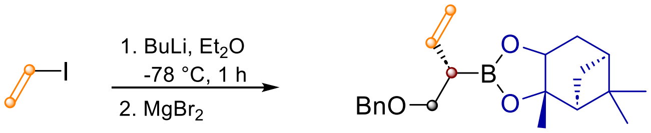

28 54 % (from **27**)

A fresh solution of vinyllithium was prepared in a Schlenk tube by dropwise addition of *n*-BuLi (2.5 M solution in hexanes, 0.59 mL, 1.5 mmol, 1.8 eq.)to a solution of vinyliodide in Et_2O (1.7 M, 0.9 mL, 1.5 mmol, 1.8 eq.) at –78 °C. The mixture was stirred for 1 h at this temperature.A solution of dry $MgBr_2$ was prepared simultaneously in a separate dried flask as described by V. Aggerwal and coworkers.[11]Therefore magnesium turnings (54 mg, 2.25 mmol, 2.7 eq.) were suspended in dry Et_2O (7.5 mL) inside a dried flask under argon. 1,2-Dibromoethane (0.13 mL, 1.5 mmol, 1.8 eq.) was added dropwise and the reaction mixture was gently heated to initiate the reaction and stirred until reflux has stopped. The biphasic mixture was added dropwise to the solution of vinyllithium at -78 °C. The mixture was stirred for 40 min. at this temperature. The resulting solution of vinylmagnesium bromide was added dropwise to another solution of boronate**27** (326 mg, 0.83 mmol, 1.0 eq.) in dry THF (10 mL) at –78 °C.The reaction mixture was allowed to slowly warm up to rt.overnight. The reaction mixture was then diluted with CyHex (20 mL), washed with saturated aq. NH_4Cl and the aqueous layer was extracted twice with Et_2O (10 mL). The combined organic phases were dried over Na_2SO_4, filtered, and concentrated *in vacuo*. The residue was purified by flash chromatography (silica gel; CyHex/EtOAc, 49:1) yielding vinylboronate**28** (153 mg, 0.45 mmol, 54 %) as a colourless oil.

R_f= 0.19 (silica gel; CyHex/EtOAc, 49:1).

^{1}H NMR (400 MHz, $CDCl_3$): δ = 7.37 – 7.24 (m, 5 H), 5.88 (ddd, *J* = 17.4, 10.2, 8.5 Hz, 1H), 5.03-5.19 (m, 2 H), 4.53 (s, 2 H), 4.28 (dd, *J* = 8.8, 1.8 Hz, 1 H),3.70 – 3.62 (m, 2 H), 2.41 – 2.28 (m, 2 H), 2.21 – 2.11 (m, 1 H), 2.05 (t, *J* = 5.5 Hz, 1 H),1.93 – 1.81 (m, 2 H), 1.38 (s, 3 H), 1.28 (s, 3 H), 1.16 (d, *J* = 10.9 Hz, 1 H), 0.83 (s, 3 H) ppm.

^{13}C NMR(100 MHz, $CDCl_3$): δ = 138.8, 136.8, 128.4, 127.7, 127.5, 115.1, 86.0, 78.0, 72.9, 71.2, 51.4, 39.6, 38.3, 35.5, 28.7, 27.2, 26.4, 26.2, 24.1 ppm.

NMR data was consistent with that reported in literature.[12]

[11] D. Leonori, V. K. Aggarwal, *Acc. Chem. Res.,* **2014**, *47*, 10, 3174–3183.

[12] F. R. Struth, C. Hirschhäuser, *Eur.J. of Org. Chem.,* **2016**, *2016*, 958-964.

Optimization, Mechanistic Discussion and Stereochemical Assignment

Matteson-Homologation –Protecting Groups and the 4thHomoloation

As indicated in the main manuscript several protecting group patterns were evaluated. As an orthogonal protecting group at R^1 was essential for our synthetic plan, the use of TBDPS-, TBS- and Trityl- was explored. The results of these efforts are summarized in Table 1. In none of these cases a 3rd homologation and substitution to **H** was achieved. Since homologation of **G** with $LiCHBr_2$ was confirmed in the TBDPS-series, we suspected formation of a six membered adduct of type **15** hampering subsequent substitution. We rationalized that formation of this intermediate suppressed the desired attack at the boron-atom. At the same time the silicon atom is activated for nucleophilic attack. This reasoning prompted the of the more shielded TIPS-group, which allowed for the synthesis of **10a** (**H** with R^1 = TIPS, R^2 = PMB, R^3 = Bn, R^4 = OBn) in a yield of 47% over two steps.

Table 1 Matteson-Homologations with alternative Protecting Group Patterns (yields not optimized).

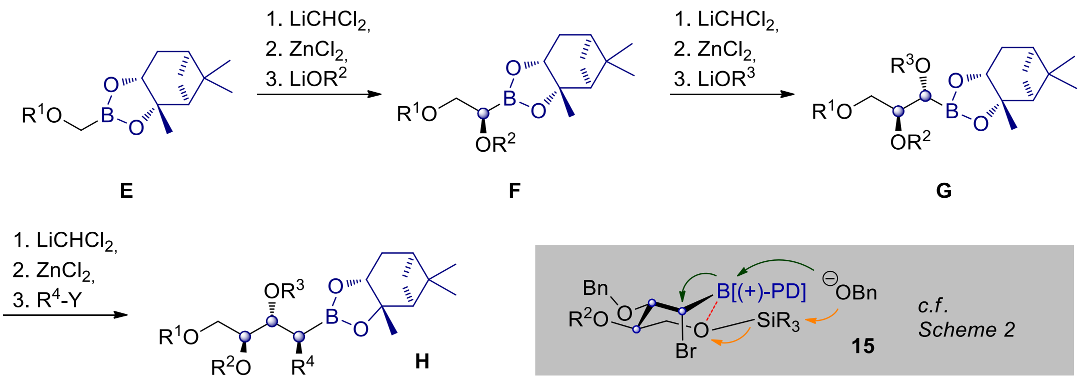

Entry	Product	R^1	R^2	R^3	R^4	Yield
1	**F1**	TBDPS	PMB	-	-	65%
2	**F2**	TBS	Bn	-	-	48%
3	**F3**	Trityl	PMB	-	-	18%
4	**G1**	TBDPS	PMB	Bn	-	32%
5	**G2**	TBS	Bn	Bn	-	49%
6	**G3**	Trityl	PMB	Bn	-	17%
7	**H1**	TBDPS	PMB	Bn	OBn	0%
8	**H2**	TBDPS	PMB	Bn	Vinyl	0%
9	**H3**	TBDPS	PMB	Bn	Allyl	0%
10	**H4**	TBS	Bn	Bn	OBn	0%
11	**H5**	Trityl	PMB	Bn	OBn	0%

The use of these bulky protecting groups at R^1 was also explored in order to test, whether a fundamental limitation in Matteson´s synthesis of Ribose could be overcome. He reported that “only” three homologation/substitution sequences could be realized when benzyl protecting groups were used at R^1-R^4. This limited Matteson´s attempt at carbohydrate synthesis to ribose, and more fundamentally to aldopentoses in general (Scheme VII). He found that starting from **5**, homologation

and subsequent substitution with LiOBn proceeded thrice, yielding the C_4-buildingblock **6a**. A forth homologation was unsuccessful with $LiCHBr_2$ and yielded only traces of product with $LiCHCl_2$.

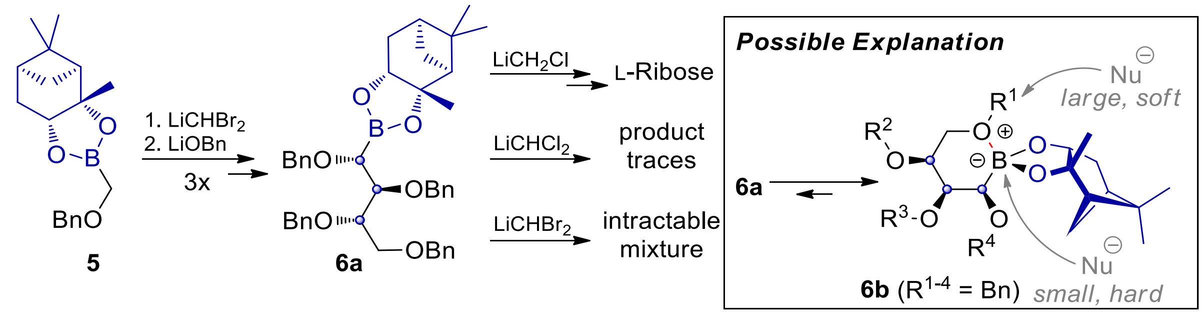

Scheme VII Matteson´s Carbohydrate Synthesis was limited to Ribose.

As we had witnessed the formation of stable 6-membered intramolecular ate complexes in our earlier work,[13] we formulated the hypothesis[14] that Matteson´s forth homologation was hampered by the formation of **6b**. Formation of this cyclic adduct could again block attack of the nucleophilic carbenoid at lower temperatures and thus interfere with the finely tuned interplay of different reaction rates necessary for a successful Matteson Reaction. (In a nutshell, ate complex formation needs to be faster than carbenoid decomposition, which in turn needs to be faster than the 1,2-rearrangement.)[9] Formation of **6b** slows down the desired nucleophilic attack at the boron-atom and leads to an activation of the benzylic stereocenter (R^1) towards nucleophilic attack. At the activated benzylic center one would expect S_N2 substitution by larger/softer nucleophiles such as $LiCHX_2$ or X^-. This interpretation is supported by the fact that Matteson achieved a forth homologation with $LiCH_2Cl$. This result looks somewhat counterintuitive at a first glance, as the less thermodynamically stable $LiCH_2Cl$ would have less time to react with the reduced amount of uncyclized **6a** available in the equilibrium. However, the nucleophilic carbenoid might also be able to attack the boron atom in **6b** directly in an S_N2-type fashion. This would require an attack from the concave side of the pinandiolboronate. Thus it stands to reason that a small and hard nucleophile such as $LiCH_2Cl$ might be able to do this, while a larger and softer nucleophile like $LiCHX_2$ preferably attacks the activated benzylic center, or undergoes decomposition. Based on these considerations we had suspected that a bulkier protecting group might be able to shift the equilibrium between the ring **6b** and its open equivalent of type **6a** in favour of the open structure. This should favour ate complex formation with larger carbenoids such as $LiCHBr_2$. This is another reason why the large protecting groups shown in Table 1 were chosen. However, at least for the use of the silyl groups TBS and TBDPS this plan was thwarted, (likely) due to the formation of **15** in the third substitution step. To spell out the irony: while taking the potential formation of a six membered adduct (**6b**) into account in order to achieve one more homologation-/substitution-sequence than Matteson himself, another six membered adduct (**15**) ensured that we achieved one less.

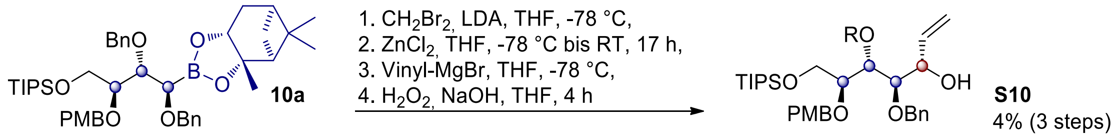

Scheme VIII 4th Homologation of 10a using an unoptimized procedure.

[13] F. R. Struth, C. Hirschhäuser, *Eur. J. Org. Chem.* **2016**, *2016*, 958.
[14] S. Kirupakaran, H.-G. Korth, C. Hirschhäuser, *Synthesis* **2018**, *50*, 2307.

However, using the base resistant and bulky TIPS-group should solve both of these problems! As mentioned above it allowed for the synthesis of **10a**, by slowing down the basic desilylation reaction *via* **15**. Its bulk should also disfavour formation of the corresponding cyclic intermediate of type **6b** and thus allow for a forth homologation. To test this hypothesis we attempted homologation of **10a**, followed by substitution with vinylmagnesiumbromide and oxidation to **S10** as shown in Scheme VIII. To our delight this did indeed yield **S10** after oxidation, though only in 4 % yield over three steps. Since we attempted this reaction at an early stage of the project, we did not make use of the optimized temperature regime, which had to be developed for the homologation of **9a** (Table 3, see below). It is likely that reaction of **10a**, which is the C_4-congener of the C_3-building block **9a**, suffers from similar side reactions as encountered with **9a**. Therefore better yields are probably achievable for this reaction. However, as a forth Matteson-Homologation was incompatible with our final synthetic plan no further optimization of this mechanistically interesting reaction was attempted.

Zweifel-Olefination with Pinanediol Boronic Esters

To the best of our knowledge Zweifel olefination of **10a** constitutes the first use of a pinandio-boronic ester in this reaction. As the introduction of a vinyl group can be problematic even with simpler boronic esters[15] optimization of the reaction was conducted as shown in Table 2. The yields given in Table 2 are partially based on NMR analysis, as purification of **11a** by chromatography proved to be quite difficult. In the final procedure isolation of **11a** was avoided and the bishydroxylation reaction was conducted immediately afterwards (see above).

Table 2 Optimization of the Zweifel Olefination for 10a.

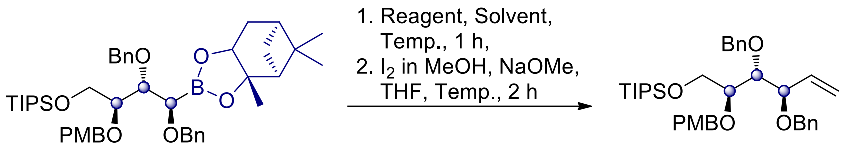

10a **11a**

Entry	Reagent	eq.	Solvent	NaOMe / eq.	I_2 / eq.	Temp.	Yield
1	VinylMgBr	4.0	THF	4.0	4.0	-78 °C to rt	0% (decomp.)
2	VinylMgBr	1.2	THF : DMSO (1:1)	1.2	1.2	-78 °C to rt	10%
3	VinylMgBr	1.2	THF : DMSO (1:1)	1.2	1.2	0 °C to rt	0%
4	VinylLi	4.0	THF	4.0	4.0	-78 °C to rt	0% (decomp.)
5	VinylLi	2.0	THF	2.0	2.0	-78 °C to rt	0% (decomp.)
6	VinylMgBr	1.8	THF	1.8	1.8	-78 °C to rt	60%
7	VinylMgBr	1.8	THF	-	1.8	-78 °C to rt	78%
8	VinylMgBr	1.0	THF	-	1.0	-78 °C to rt	67%

[15] R. J.Armstrong, W. Niwetmarin, V. K. Aggarwal *Org. Lett.* **2017**, *19*, 2762

Matteson Reaction with Vinylmagnesiumbromide and Oxidation

Matteson Homologation of **9a**, reaction with vinylmagnesiumbromide (**11c**) and subsequent oxidation to alcohol **16c** proved to be surprisingly difficult and required optimization (Table 3). The key was as a strict temperature regime in each of the three steps.

Table 3 Optimization of Substitution with Vinylmagnesiubromide and subsequent Oxidation.

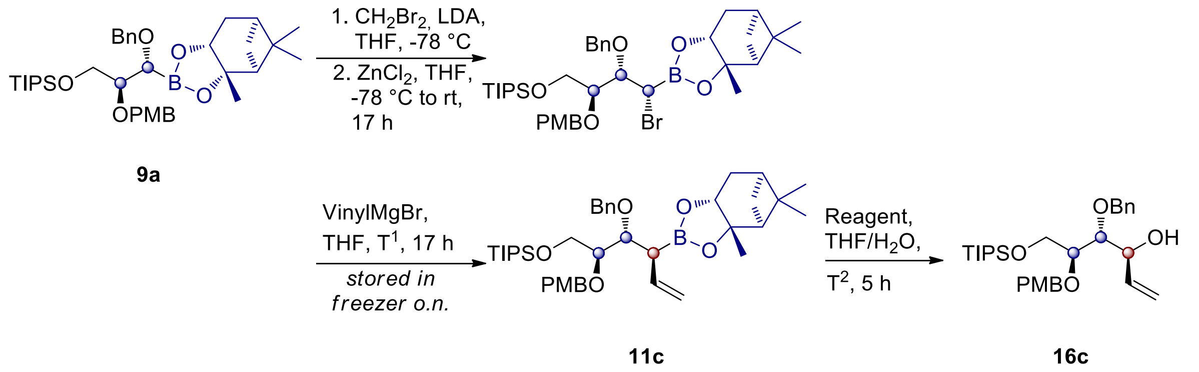

Entry	VinylMgBr (equiv.)	T^1	Work up of 11c?	Reagent	T^2	Yield (from 9a)
**1**	4.0	-78 °C to 20°C	yes	H_2O_2/NaOH	0 °C to 20 °C	0%
**2**	3.6	-78 °C to 20°C	yes	H_2O_2/NaOH	0 °C to 20 °C	0%
**3**	3.0	-78 °C to 20°C	yes	H_2O_2/NaOH	0 °C to 20 °C	10%
**4**	2.0	-78 °C to 20°C	no	H_2O_2/NaOH	0 °C to 20 °C	24%
**5**	1.0	-78 °C to 20°C	no	$NaBO_3$	0 °C to 20 °C	0%
**6**	2.0	-78 °C to 20°C	no	$NaBO_3$	0 °C to 20 °C	14%
**7**	4.0	-78 °C to 20°C	no	$NaBO_3$	0 °C to 20 °C	0%
**8**	2.5	-78 °C to 10°C	no	H_2O_2/NaOH	0 °C to 20 °C	6%
**9**	2.0	-78 °C to 10°C	no	H_2O_2/NaOH	0 °C to 20 °C	27%
**10**	2.0	-78 °C to 0°C	no	H_2O_2/NaOH	0 °C to 20 °C	31%
**11**	1.8	-78 °C to 0°C	no	H_2O_2/NaOH	0 °C to 20 °C	24%
**12**	2.0	-78 °C to 0°C	no	H_2O_2/NaOH	0 °C to 20 °C	33%[a]
**14**	1.3	-78 °C to 0°C	no	H_2O_2/NaOH	0 °C to 20 °C	20%
**14**	1.0	-78 °C to 0°C	no	H_2O_2/NaOH	0 °C to 20 °C	0%
**15**	1.8	-78 °C to 0°C	no	H_2O_2/NaOH	0 °C to 10 °C	30%
**16**	2.0	-78 °C to 0°C	no	H_2O_2/NaOH	0 °C to 10 °C	34%
**17**	**2.0**	**-78 °C to 0°C**	**no**	**H_2O_2/NaOH**	**0 °C**	**52%**

[a] Vinylation reaction conducted in freezer over 6 weeks.

Usually Matteson-Homologations and substitutions can be conducted by forming the corresponding ate complexes at low temperatures and allowing the reaction mixtures to reach room temperature e.g. over night. While this was still possible for the homologation of **9a** with lithiated dibromomethane, substitution and subsequent oxidation required a much greater amount of care. In early attempts (Entry 1-7) vinylgrignard was added at -78 °C and the mixture was allowed to warm to rt over night. Similarly the oxidation was carried out by adding the oxidant under ice bath cooling and allowing the reaction mixture to warm up to rt. Several undesired side products were identified from these reactions, the structure and formation of which is shown in Scheme IX. It is likely that the Aldehyde **S11** was formed from leftover α-bromo boronate after oxidation with H_2O_2. Since an access

of vinylmagnesiumbromide was used on most occasions, this indicates that the desired reaction to **11c** was incomplete. This could occur for example if the 1,2-rearrangement of the corresponding ate complex to **11c** is unusually fast. Subsequent reaction with another equivalent of vinylmagnesiumbromide would lead to **S12** and **S13**, both of which were observed (**S13** was observed in a crude NMR before oxidation). To avoid the thermodynamically favourable reaction to the conjugated diene **S12**, addition of vinylmagnesiumbromide was conducted slowly and instead of allowing the reaction mixture to warm up to room temperature, the flask was stored inside a freezer (-18 °C) over night. After that it was transferred into an ice bath, so that strict temperature control was achieved.

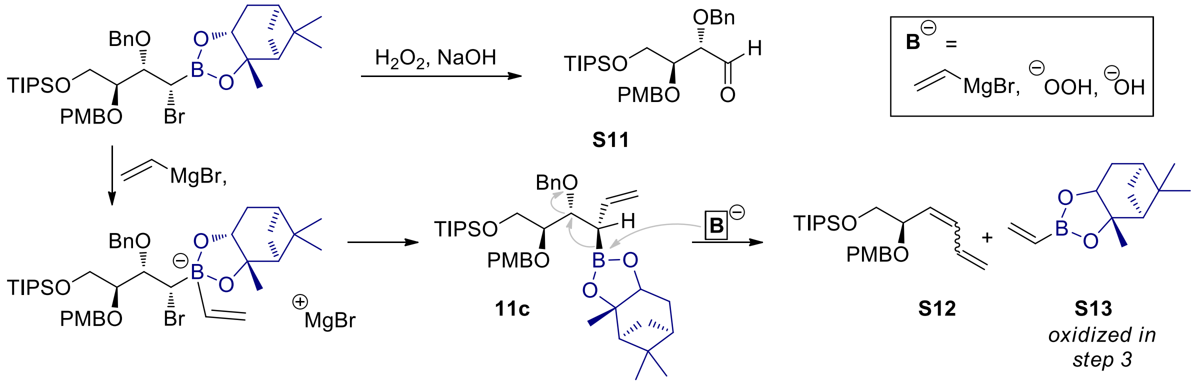

Scheme IX Side products observed in the synthesis of 16c and rational for their formation.

Oxidation of **11c** also involves formation of an ate complex. Thus to much heat in this step also leads to elimination. Therefore slow addition of base and oxidant, as well as constant cooling, were necessary to finally achieve a yield of 52% over three steps (Entry 17). It should be mentioned that upon using vinylgrignard reagents in Matteson reactions vinylboronates like **S13** can also be formed without suitable leaving groups in the β-position. This is most likely occurs by protodeborylation of the α-halo boron ate complex.[16]

Optimization and Stereochemical Assignment of the Bishydroxylation

Sharpless-Bishydroxylation was employed in order to control the last stereocenter. The reaction was first explored on a small test system with commercially available AD-Mix (Table 4). Even in this small molecule a strong matched/miss-matched effect was visible, although not as strong as in the alkenes of type **11**.

Table 4 Sharpless Bishydroxylation of *S*-1,2 Dibenzyloxibut-3-en with commercially available AD-Mix.

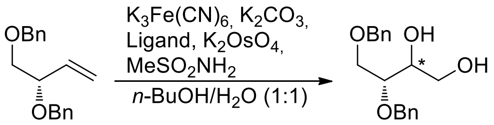

Entry	Ligand	Ligand/ mol%	K_2OsO_4/ mol%	Temp.	d.r.	Yield
**1**	$(DHQD)_2$PHAL (AD-Mix ß)	3.0	1.4	0 bis 20 °C	19:81	64%
**2**	$(DHQ)_2$PHAL (AD-Mix α)	3.0	1.4	0 bis 20 °C	58:42	68%

[16] T. Kisinger, U. Kazmaier, *Org. Lett.* **2022**, *24*, 3599.

The bishydroxylation of **11c**, leading to mannitol **12c** was optimized most extensively and was the basis for our stereochemical assignment. Thus these results will be discussed first. As shown in Table 5, both commercially available, premixed reagents, as well as “selfmade” mixtures were tested. The latter gave significantly better yields and d.r. values. In all cases, however, strong substrate control was visible as both DHQD and DHQ ligands predominantly produced the same product.

Table 5 Sharpless Bisyhdroxylation of 11c.

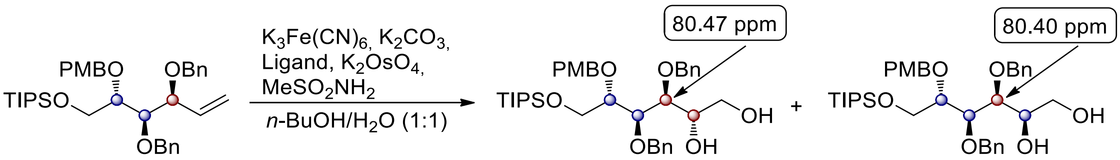

**Entry**	**Ligand**	**Ligand/ mol%**	**K_2OsO_4/ mol%**	**Time**	**Temp.**	**d.r. (12c : S5)**	**Yield**
**1**	$(DHQD)_2$PHAL (AD-Mix)	3.0	1.4	17 h	0 to 20 °C	70:30	23%
**2**	$(DHQ)_2$PHAL (AD-Mix)	3.0	1.4	17 h	0 to 20 °C	77:23	47%
**3**	$(DHQD)_2$PHAL (AD-Mix)	24	11	17 h	0 to 20 °C	79:21	50%
**4**	$(DHQD)_2$PHAL (AD-Mix)	24	11	48 h	0 to 20 °C	81:19	67%
**5**	$(DHQD)_2$PHAL (AD-Mix)	24	11	96 h	0 to 20 °C	88:12	64%
**6**	$(DHQD)_2$AQN	10	2	48 h	0 to 20 °C	92:8	80%
**7**	$(DHQ)_2$AQN	10	2	48 h	0 to 20 °C	77:23	n.b
**8**	$(DHQD)_2$Pyr	10	2	48 h	0 to 20 °C	85:15	n.b
**9**	$(DHQ)_2$Pyr	10	2	48 h	0 to 20 °C	95:5	39%
**10**	$(DHQD)_2$AQN	10	2	48 h	0 to 5 °C	**99:1**	**82%**
**11**	$(DHQ)_2$Pyr	10	2	48 h	0 to 5 °C	**99:1**	**56%**

Assessment of the diastereomeric ratios from ^{1}H NMR data was not feasible, but integration of corresponding resonances at 80.47 and 80.40 ppm in ^{13}C NMRs of sufficient concentration allowed for swift d.r. assessment. The reliability of the values obtained this way was confirmed by comparison to data obtained from HPLC (Table 6).

Table 6 HPLC analysis of Entry 5 gave a d.r. (87:13), which corresponded well to the value obtained by NMR (88:12).

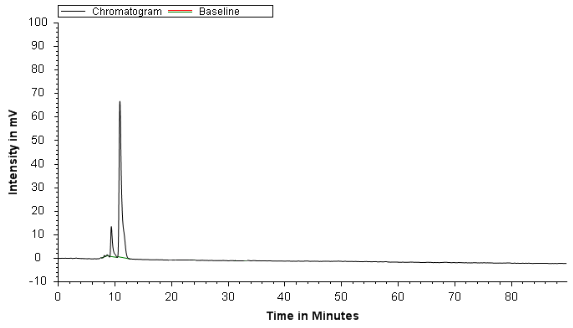

**Peak Nr.**	**Retentiontime**	**Hight**	**Area**	**%**
**1**	9.333333	12.99718	305564.6	12.62
**2**	10.84167	66.01249	2116339	87.38

Given the high degree of substrate control in this reaction, assignment of the relative configuration of **11a** and **S7** using the classic Sharpless mnemonics[17] seemed somewhat unreliable. In order to allow for easy confirmation of the relative configuration of the final sugar alcohol, the protecting group pattern in **12** was chosen in such a way that it allowed for comparison with a commercially available mannitol derivative.

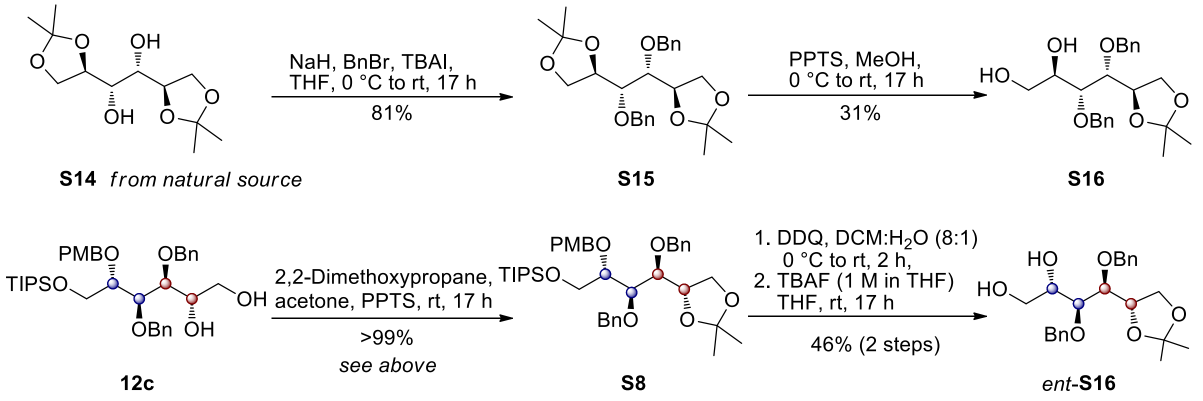

Scheme X Confirming the relative configuration of 12c by comparison to a mannitol derivative from a natural source.

As shown in Scheme X conversion of commercially available **S14** into the corresponding benzyl ether **S15** was straight forward. Acetal cleavage was interrupted before completion, so that **S16** could be isolated. The corresponding enantiomer *ent*-**S16** was available from **12c** after acetal protection of the newly formed diol (yielding **S8**) and subsequent removal of the PMB- and TIPS-groups. Both ^{1}H and ^{13}C NMR spectra of these compounds matched exactly (Figure 1). This way the relative configuration of **12c** was confirmed as the one predicted by the Houk-model shown in Scheme 4.

(1R,2R)-1,2-bis(benzyloxy)-1,2-bis((R)-2,2-dimethyl-1,3-dioxolan-4-yl)ethane (S15)

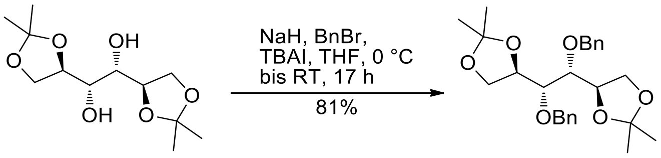

S14 **S15**

Sodium hydride (60% dispersion in mineral oil, 1.1 g, 28.5 mmol, 2.5 eq.) was added to a solution of **S14** (3.0 g, 11.4 mmol, 1.0 eq.) in THF (50 mL) at 0 °C under argon and the mixture was stirred for 30 min at rt. Then benzyl bromide (3.38 mL, 28.5 mmol, 2.5 eq.) was added dropwise at 0 °C and subsequently TBAI (421 mg, 1.14 mmol, 10 mol%) was added. The mixture was allowed to warm up to rt over 17 h. NH_4Cl (aq. sat. 50 mL) was added and the aqueous phase was extracted with EtOAc (3 x 50 mL). The organic phase was dried over $MgSO_4$ and the solvent was removed *in vacuo*. The residue was purified by flash chromatography (SiO_2, CyHex/EtOAc, 9:1, $KMnO_4$) yielding **S15**(4.1 g, 9.267 mmol, 81%) as a colourless oil.

^{1}H NMR (400 MHz, $CDCl_3$): δ (ppm) = 7.31 – 7.17 (m, 10H), 4.64 (s, 4H), 4.23 – 4.13 (m, 2H), 3.94 (dd, J = 8.4, 6.2 Hz, 2H), 3.79 (dd, J = 8.4, 6.5 Hz, 2H), 3.76 – 3.69 (m, 2H), 1.36 (s, 6H), 1.27 (s, 6H).

^{13}C NMR (101 MHz, $CDCl_3$): δ (ppm) = 138.4, 128.5, 128.2, 127.9, 108.7, 80.1, 76.0, 74.8, 66.9, 26.8, 25.4.

[17] (a) E. N. Jacobsen ,I. Marko , W. S. Mungall , G. Schroeder , K. B. Sharpless , *J. Am. Chem. Soc.* **1988**, *110*,1968 –1970; (b) H. C. Kolb, P. G. An-dersson, K. B. Sharpless,*J. Am. Chem. Soc.***1994**,*116*, 1278–1291; c) P.-O. Norrby, H. C. Kolb, K. B. Sharpless, *J. Am.Chem. Soc.* **1994**,*116*, 8470–8478

Spectroscopic data were consistent with those reported in the literature.[18a]

(2R,3R,4R)-3,4-bis(benzyloxy)-4-((R)-2,2-dimethyl-1,3-dioxolan-4-yl)butane-1,2-diol (S16)

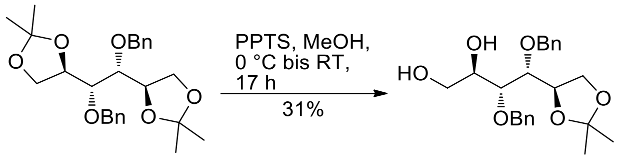

S15 **S16**

To a solution of **S15** (362 mg, 0.82 mmol, 1.0 eq.) in MeOH (5 mL) was added PPTS (123 mg, 0.82 mmol, 1.0 eq.) was added and the mixture stirred for 17 h. The solvent was removed *in vacuo* and the residue was purified by flash chromatography (SiO_2, CyHex/EtOAc, 1:1, $KMnO_4$) yielding **S16** (102 mg, 0.25 mmol, 31%.) as a colourless oil.

^{1}H NMR (400 MHz, $CDCl_3$): δ (ppm) = 7.41 – 7.26 (m, 10H), 4.82 – 4.57 (m, 4H), 4.33-4.29 (m, 1H), 4.07 (dd, J = 8.3, 6.4 Hz, 1H), 4.01 – 3.92 (m, 2H), 3.85 – 3.59 (m, 4H), 2.80 (s (br.), 1H), 1.81 (s (br.), 1H), 1.45 (s, 3H), 1.35 (s, 3H).

^{13}C NMR (101 MHz, $CDCl_3$): δ (ppm) = 137.88, 137,82, 128.72, 128.67, 128.41, 128.26, 128.19, 108.74, 79.32, 78.67, 76.16, 74.65, 74.01, 71.30, 66.52, 63.72, 26.69, 25.23.

Spectroscopic data were consistent with those reported in the literature.[18b]

(2S,3S,4S)-3,4-bis(benzyloxy)-4-((S)-2,2-dimethyl-1,3-dioxolan-4-yl)butane-1,2-diol (*ent*-S16)

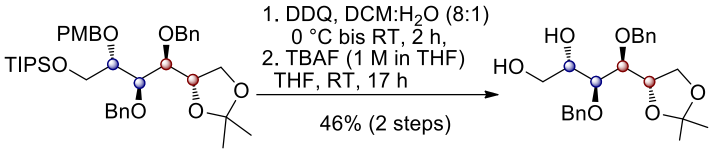

S8 *ent*-**S16**

To solution of **S8** (100 mg, 0.15 mmol, 1.0 eq.) in CH_2Cl_2 (4 mL) and PBS buffer (pH = 7.4, 0.5 mL), DDQ (47 mg, 0.21 mmol, 1.4 eq.) was added at 0 °C and the mixture was stirred for 1 h. Saturated aqueous $NaHCO_3$ (5 mL) was added, and the aqueous phase was extracted with EtOAc(3 x 5 mL). The organic phase was dried over $MgSO_4$ and the solvent was removed *in vacuo*. The residue was purified by flash chromatography (SiO_2, CyHex/EtOAc, 9:1, $KMnO_4$) yielding the PMB-deprotected product (89 mg, 0.15 mmol, quant.) as a colourless oil. To a solution of this product (75 mg, 0.134 mmol, 1.0 eq.) in THF (2 mL), TBAF (1 M in THF, 0.2 mL, 0.19 mmol, 1.4 eq.) was added at 0 °C and the mixture was stirred for 17 h. The solvents were removed *in vacuo*. The residue was purified by flash chromatography (SiO_2, CyHex/EtOAc, 2:1, $KMnO_4$) yielding *ent*-**S16** (25 mg, 0.06 mmol, 46%) as a colourless oil.

R_f (EA/CH 1:2) = 0.09.

$[\alpha]_D^{20}$= -18.9 (0.016, $CHCl_3$).

---

[18] a) C.-G. Mabiala-Bassiloua, G. Arthus-Cartier, V. Hannaert, H. Thérisod, J. Sygush, M. Thérisod, *ACS Med. Chem. Lett.* **2011**, 2, *11*, 804-808. b) M. Chandrasekhar, K. L. Chandra, V. K. Singh, *Arkivoc* **2002**, *2002*, 34

^{1}H NMR (400 MHz, $CDCl_3$): δ (ppm) = 7.40 – 7.27 (m, 10H), 4.78 – 4.59 (m, 4H), 4.34 – 4.29 (m, 1H), 4.07 (dd, J = 8.3, 6.4 Hz, 1H), 4.01 – 3.91 (m, 2H), 3.81-3.63 (m, 4H), 2.83 (s (br.), 1H), 1.82 (s (br.), 1H) 1.45 (s, 3H), 1.35 (s, 3H).

^{13}C NMR (101 MHz, $CDCl_3$): δ (ppm) = 137.87, 137.82, 128.72, 128.67, 128.41, 128.27, 128.19, 108.74, 79.33, 78.63, 76.17, 74.66, 74.01, 71.32, 66.52, 63.72, 26.70, 25.23.

IR (film) ν_{max} (cm^{-1}): 3446, 1450, 1064, 735, 698.

ESI-HRMS: calc. for $[C_{23}H_{30}O_6+Na]^+$ 425.1935, found 425.1940.

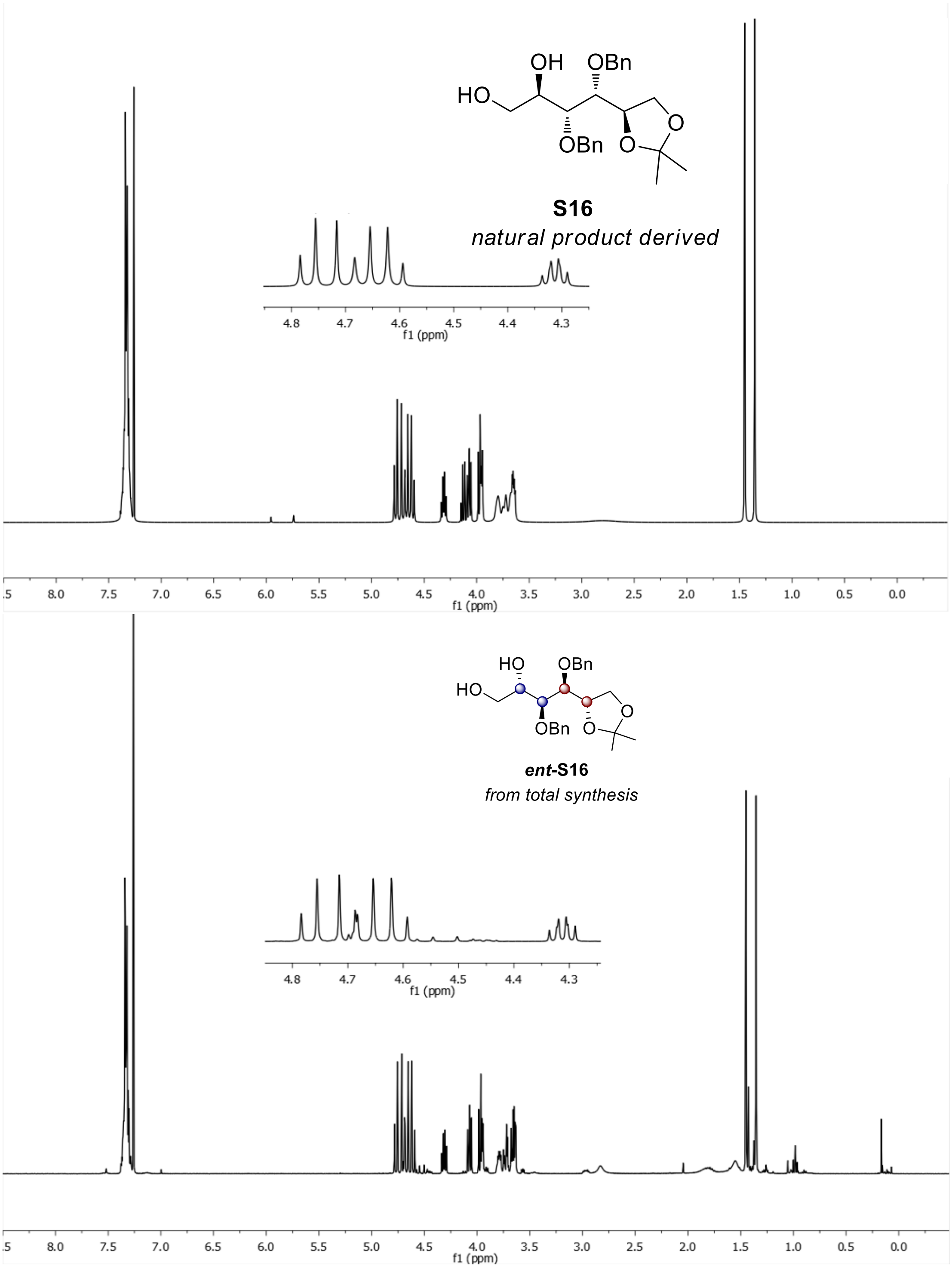

Figure 1 ^{1}H NMR Spectra of natural product derived S16 (top) and *ent*-S16 prepared by total synthesis (bottom).

After the unambiguous assignment of **12c** *via* conversion into *ent*-**S16** (Figure 1), interpretation of the bishydroxylation results for **11b** and **11a** (Table 7) was straight forward. As **11b** has the same configuration at C^4 as **11c**, the Houk-model predicts the preferred formation of **12b** and thus a matched case for the same ligands (Table 5). As can be seen in Table 7, Entries 1 and 3, both $(DHQD)_2AQN$ and $(DHQ)_2Pyr$ delivered better d.r. values than their counterparts, as was the case in Table 5, Entries 10 and 11. In the case of **11a**, configuration at C^4 is inverted and so are the matched and mismatched cases. In the bishydroxylation of **11a** $(DHQ)_2AQN$, which was a mismatched ligand for **11b** (Table 7, Entry 2) and **11c** (Table 5, Entry 7), delivered a higher d.r. value than $(DHQD)_2AQN$, i.e. the matched Ligand for **11b** (Table 7, Entry 1) and **11c** (Table 5, Entry 10). Thus predictions of the Houk-model are consistent with the matched/mismatched cases of all bishydroxylation reactions.

Table 7 Bishydroxylation experiments for 11b and 11a as well as differentially protected derivative S17

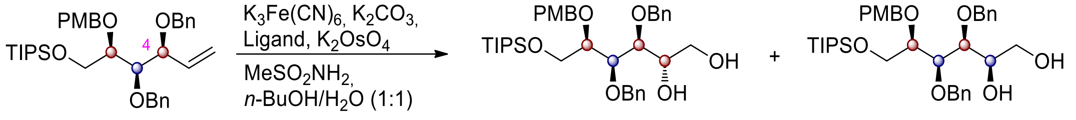

11b **12b** ***epi*-12b**

Entry	Ligand	Ligand/ mol%	K_2OsO_4/ mol%	Time	Temp.	d.r.(12b: *epi*-12b)	Yield
**1**	$(DHQD)_2AQN$	10	2	48 h	0 - 5 °C	**83:17**	**55%**
**2**	$(DHQ)_2AQN$	10	2	48 h	0 - 5 °C	63:37	43%
**3**	$(DHQ)_2Pyr$	10	2	48 h	0 - 5 °C	**93:7**	**40%**

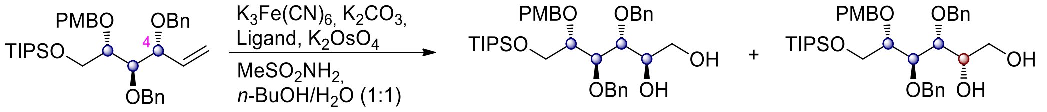

11a **12a** ***epi*-12a**

Entry	Ligand	Ligand/ mol%	K_2OsO_4/ mol%	Time	Temp.	d.r.(12a: *epi*-12a)	Yield
**4**	$(DHQD)_2AQN$	10	2	48 h	0 - 5 °C	78:22	n.d.
**5**	$(DHQ)_2AQN$	10	2	48 h	0 - 5 °C	**92:8**	**32%**[a]

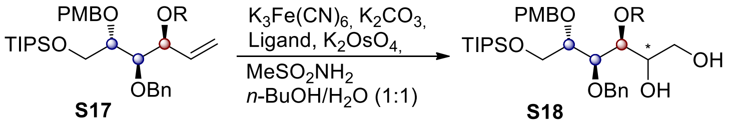

S17 **S18**

Entry	R	Ligand	Ligand/ mol%	K_2OsO_4/ mol%	Time	Temp.	d.r.[b] (major:minor)	Yield
**6**	TBS	$(DHQD)_2Pyr$	10	2	48 h	0 °C bis 5 °C	75:25	63%
**7**	TBS	$(DHQ)_2Pyr$	10	2	48 h	0 °C bis 5 °C	**90:10**	**45%**
**8**	TBS	$(DHQD)_2AQN$	10	2	48 h	0 °C bis 5 °C	77:23	50%
**9**	TBS	$(DHQ)_2AQN$	10	2	48 h	0 °C bis 5 °C	85:15	41%
**10**	SEM	$(DHQ)_2Pyr$	10	2	48 h	0 °C bis 5 °C	88:12	20%
**11**	SEM	$(DHQD)_2AQN$	10	2	48 h	0 °C bis 5 °C	50:50	29%
**12**	SEM	$(DHQ)_2AQN$	10	2	48 h	0 °C bis 5 °C	70:30	47%
**13**	TIPS	$(DHQ)_2Pyr$	10	2	48 h	0 °C bis 5 °C	81:19	47%
**14**	TIPS	$(DHQD)_2AQN$	10	2	48 h	0 °C bis 5 °C	77:23	44%
**15**	TIPS	$(DHQ)_2AQN$	10	2	48 h	0 °C bis 5 °C	87:13	59%

[a] Yield over 3 steps (from **10a**). [b] Reliable assignment of the diastereomers not possible based on the available data.

Furthermore bishydroxylation of differentially protected derivatives of type **S17** to diols of type **S18** was explored (Table 7, Entries 6-15). These derivatives are of interest, when other protecting group patterns are necessary, and might even lead to different stereochemical outcomes. Therefore protecting groups of varying steric demand (TBS, SEM and TIPS) were tested. Unfortunately the stereochemical assignment of diastereomeric diols of type **S18** could not be based on a comparison to the corresponding benzyl congeners of type **12**. Therefore diastereomeric ratios (major to minor) are reported without assignment for Entries 6-15. Compared to the bishydroxylation of **12c** (Table 5) a switch from matched to mismatched cases was observed for the SEM and TIPS protected derivatives (Entries 11 and 12, as well as 14 and 15 respectively). This might indicate a predominant formation of the *threo* diastereomere, which could be developed into a more efficient way for installing a *threo* relationship between C^4 and C^5, than the inversion route described in the main article (Scheme 4).

In the hope of finding a faster route to a C^4-C^5*threo* product we also attempted Sharpless-epoxidation of **16c**, which reacted surprisingly slowly under the tested conditions (Table 8).

Table 8 Attempts at Sharpless-Epoxidation of 16c

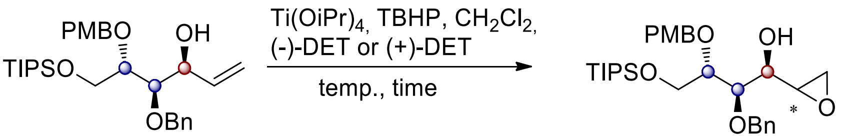

16c

Entry	Reagent	temp.	time	Yield
**1**	(-)-DET	0 °C to rt	24 h	0% (100% SM)
**2**	(+)-DET	0 °C to rt	24 h	0% (100% SM)
**3**	(-)-DET	- 78 °C to rt	96 h	2% (98% SM)
**4**	(+)-DET	- 78 °C to rt	96 h	2% (98% SM)

Attempts to invert the configuration at C5 by Mitsunobu-Reaction

Attempts to obtain C^4-C^5*threo* products of type **S7** by inversion of **11b** through Mitsunobu reaction were unsuccessful (Table 9).

Table 9 Attempts at preparing C^4-C^5*threo* products by Mitsunobu Reaction.

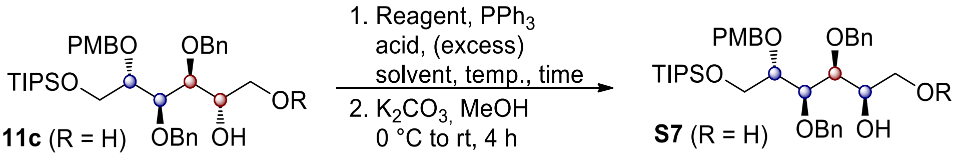

Entry	R	Reagent	Acid	Solvent	Yield
**1**	H	DIAD	*p*-NO_2-PhCOOH	THF	0% (retention of configuration)
**2**	H	DEAD	*p*-NO_2-PhCOOH	THF	0% (retention of configuration)
**3**	H	DIAD	*p*-NO_2-PhCOOH	$PhCH_3$	0% (retention of configuration)
**4**	H	DEAD	*p*-NO_2-PhCOOH	$PhCH_3$	0% (retention of configuration)
**5**	H	DEAD	HOAc	THF	0% (retention of configuration)
**6**	H	DEAD	PhCOOH	THF	0% (retention of configuration)
**7**	TBS	DEAD	*p*-NO_2-PhCOOH	THF	0% (decomp.)
**8**	Piv	DEAD	*p*-NO_2-PhCOOH	THF	0% (decomp.)

Attempts to invert the configuration at C4 by Mitsunobu-Reaction

Inversion of the alcohol **16c** by Mitsunobu-reaction was explored as an alternative to the synthesis of Allitol *via* Zweifel-Olefination (Table 10). While Mitsunobu reaction with acetic acid and benzoic acid was unsuccessful (Entries 6 and 8), reaction with *para*-nitro benzoic acid delivered the desired alcohols in good yields with both DIAD and DEAD (Entries 1-2). The diastereomeric ratio, however, was 75:25 although diastereomerically pure **16c** was used in all cases.

Table 10 Synthesis of 16a by Mitsunobu Reaction

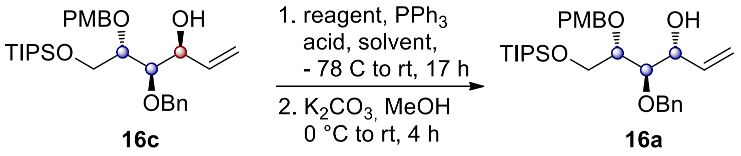

Entry	Method[a]	reagent	Acid	Solvent	Yield	d.r. (16a:16c)
**1**	A	DIAD	*p*-NO_2-PhCOOH	THF	80%	75:25
**2**	A	DEAD	*p*-NO_2-PhCOOH	THF	72%	75:25
**3**	B	DIAD	*p*-NO_2-PhCOOH	THF	78%	75:25
**4**	B	DEAD	*p*-NO_2-PhCOOH	$PhCH_3$	73%	75:25
**5**	B	DIAD	*p*-NO_2-PhCOOH	$PhCH_3$	68%	75:25
**6**	B	DIAD	HOAc	THF	decomp.	-
**7**	B	DIAD	PhCOOH	THF	decomp.	-

[a]Method A: All in one-pot, B: DEAD+PPh_3/DIAD+PPh_3 in solvent first, rest added later.

This observation is incompatible with a diastereospecific S_N2-mechanism. But it is reasonable to assume that the reaction proceeds *via* an allylic cation, which is stabilized by neighbouring group participation (Scheme XI). Formation of this cation could be fostered by the strong acid (*p*-NO_2-PhCOOH), but neither the premixing of DIAD and PPh_3 (to generate a buffer before the addition of *p*-NO_2-PhCOOH, Entry 3), nor the use of toluene (Entries 4 and 5) led to better diastereomic ratios.

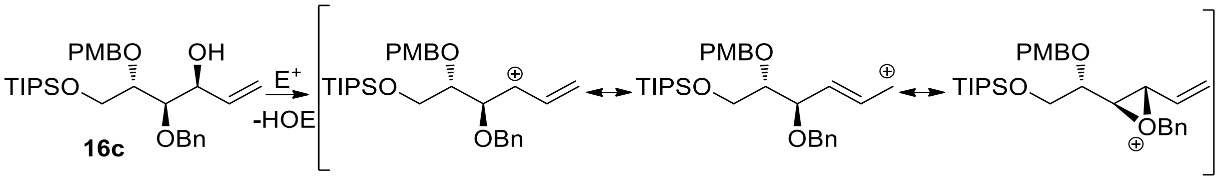

Scheme XI Proposed explanation for the d.r. observed upon Mitsunobu Reaction of 16c with *p*-NO_2-PhCOOH.

Optimization of Vinyl Iodide Synthesis

Optimization of the route to vinyl boronic esters from C_1-building blocks is shown in Table 11. Since vinylpinacol boronic ester is quite volatile, transesterification with pinanediol was usually conducted to facilitate isolation by chromatography (Entry 1-4). For this pinanediol with a low *ee* can be employed. This is a waste byproduct from the preparation of enantiomerically pure pinanediol. Pinandiol prepared from natural (+)- or (-)-pinene has to be recrystallized as the terpene precursor only comes with 80% *ee*. Thus low *ee* pinanediol can be reisolated from the mother liquor of said recrystallization. Isolation of Vinylpinacol boronic ester was attempted by distillation yielding a moderately contaminated sample after the first distillation attempt (Entry 5).

Table 11 Optimization of the vinylboronate synthesis.

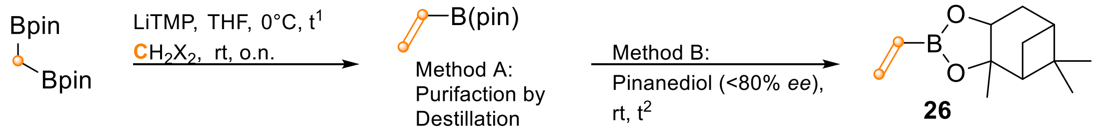

Entry	X	t^1	t^2	Isolation Method	Yield
**1**	Br	0,5 h	o.n.	B	35 %[a]
**2**	I	0,5 h	o.n.	B	51 %[a]
**3**	I	1,5 h	60 h	B	71 %[a]
**4**	I	2 h	48 h	B	52 %
**5**	I	1,5 h	–	A	<73 %

[a] Yield determined by ^{1}H NMR analysis (400 MHz), based on internal standard.

Optimizing the Iodination of Vinyl Boronic Esters

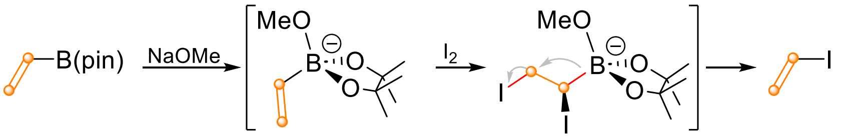

A quick optimization for the iodination of vinylboronic esters was carried out on commercially available vinyl pinacol boronate. Initially the formation of the product was monitored by ^{1}H NMR using an internal standard. However, distillation completed the elimination and led to better yields. Adding NaOMe before I_2 led to better yields, presumably as the more electron rich ate complex is more reactive towards electrophilic addition of iodine.

Table 12Optimizing the iodination of vinyl boronic esters.

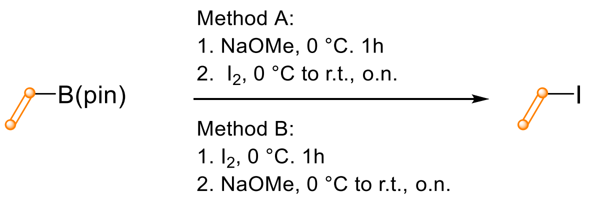

Entry	I_2(equiv.)	Method	Yield
**1**	2.5	A	65 %[a]
**2**	2.5	B	45 %
**3**	1.0	B	55 %
**4**	2.5	A	90 %

[a] Yield determined by ^{1}H NMR analysis (400 MHz), based on internal standard.

Reaction of Vinyliodide with α-Halo Borononic Esters

As vinyliodide generated from C_1-building blocks is relatively precious and likely to be applied in small quantities, generation of vinyllithium and vinylgrignard reagents by transmetallation is more comfortable than the reaction with lithium or magnesium metal. While iodo-lithium exchange proceeded readily, several attempts at iodo-magnesium exchange were unsuccessful in our hands. While this is likely to be of little concern for the Zweifel-route, Mg^{2+} is known to play an important role in 1,2-migration chemistry.[11] Therefore $MgBr_2$ was freshly prepared from Magnesium-metal and dibromoethane and added to the homologation. Results for the optimization are shown in Table 13.

Table 13 Optimization of Substitution with Vinyllithium.

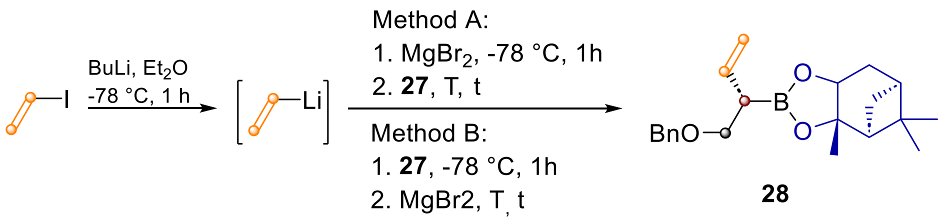

Entry	Vinyl-Li(equiv.)	$MgBr_2$(equiv.)	Method	T	t	Yield
**1**	1.8	2.4	B	-20 °C	3 d	22 %
**2**	1.8	3.6	B	-78 °C to rt	o. n.	31 %
**3**	2.4	2.4	A	-78 °C to rt	o. n.	35 %
**4**	1.8	1.8	A	-78 °C to rt	o. n.	48 %
**5**	1.8	2.4	A	-84 °C to rt	o. n.	55 %
**6**	1.8	3.6	A	-78 °C to rt	o. n.	54 %

NMR Spectra

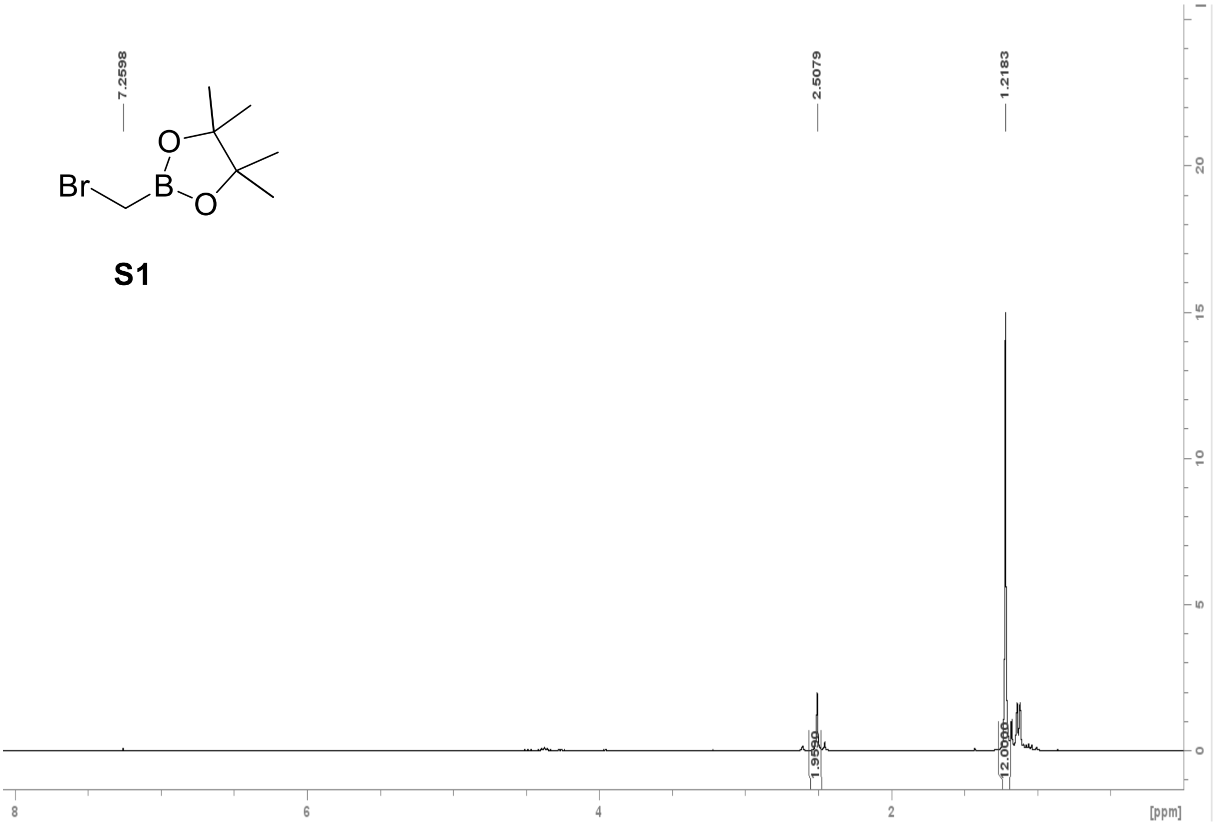

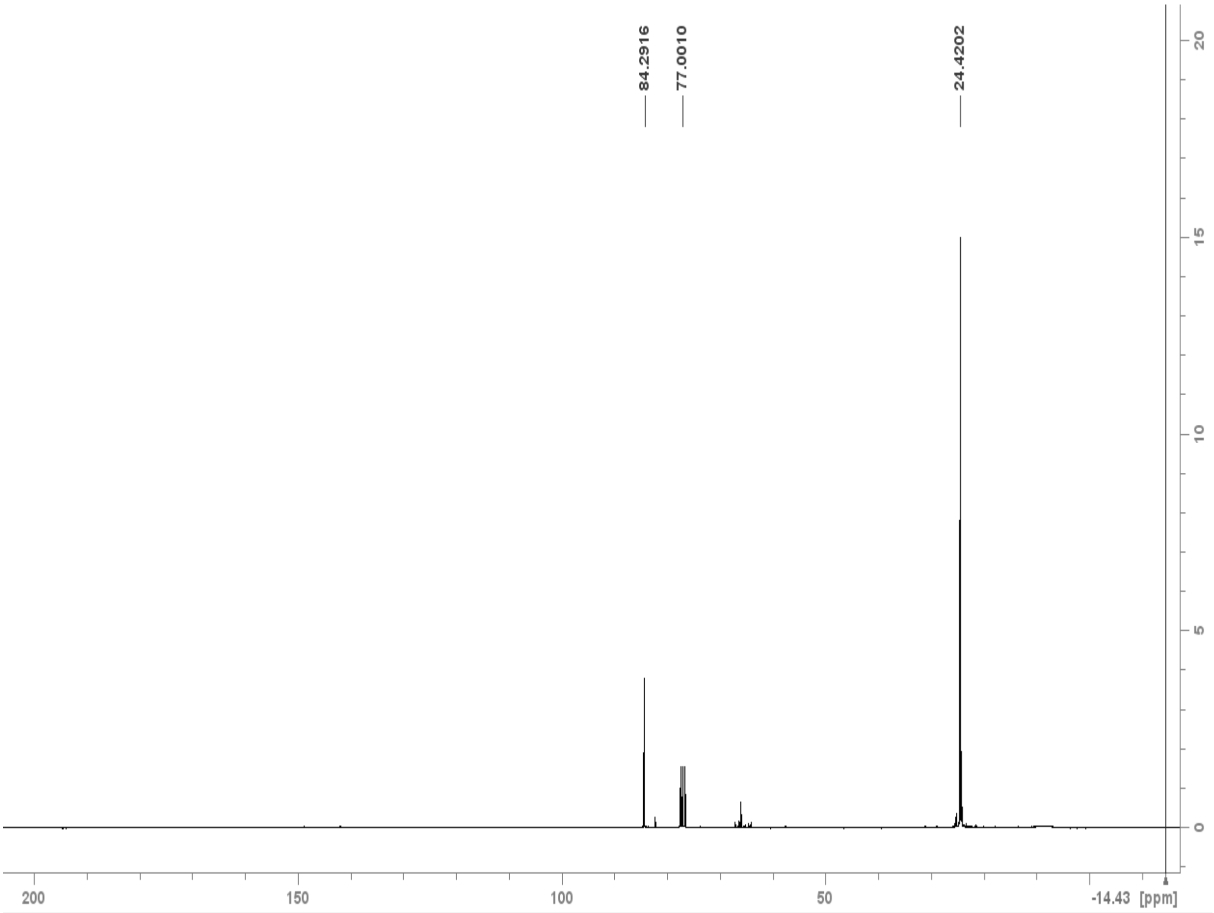

Contains traces of *i*PrOB(pin) and/or *i*PrOCH$_2$-B(pin) which were removed after the next step.

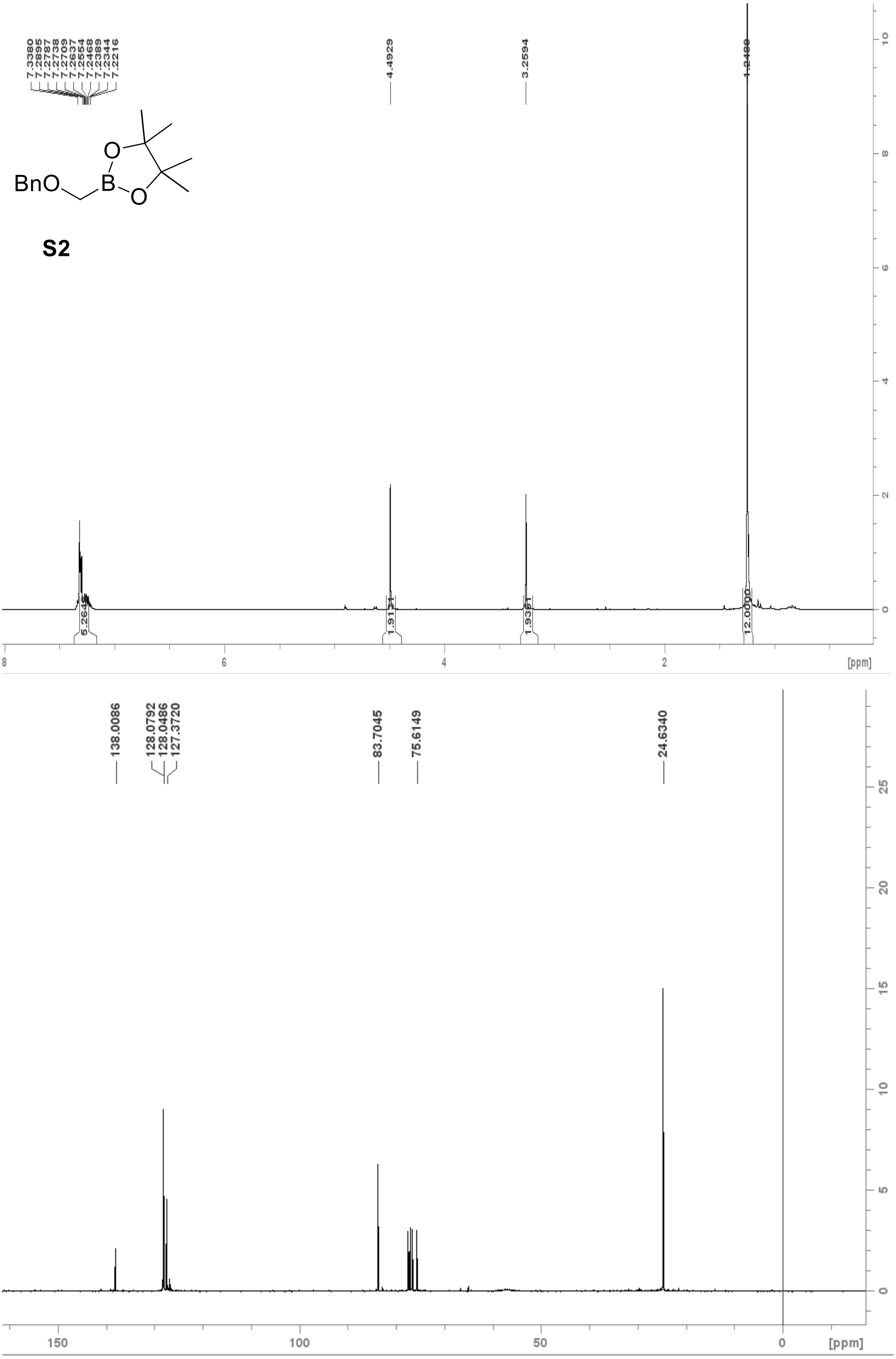
BnO
B
O
O
S2
7.3380
7.2895
7.2787
7.2738
7.2709
7.2637
7.2554
7.2468
7.2389
7.2344
7.2216
4.4929
3.2594
1.2488
5.264
1.9171
1.9361
12.0000
[ppm]
138.0086
128.0792
128.0486
127.3720
83.7045
75.6149
24.6340
150
100
50
0
[ppm]

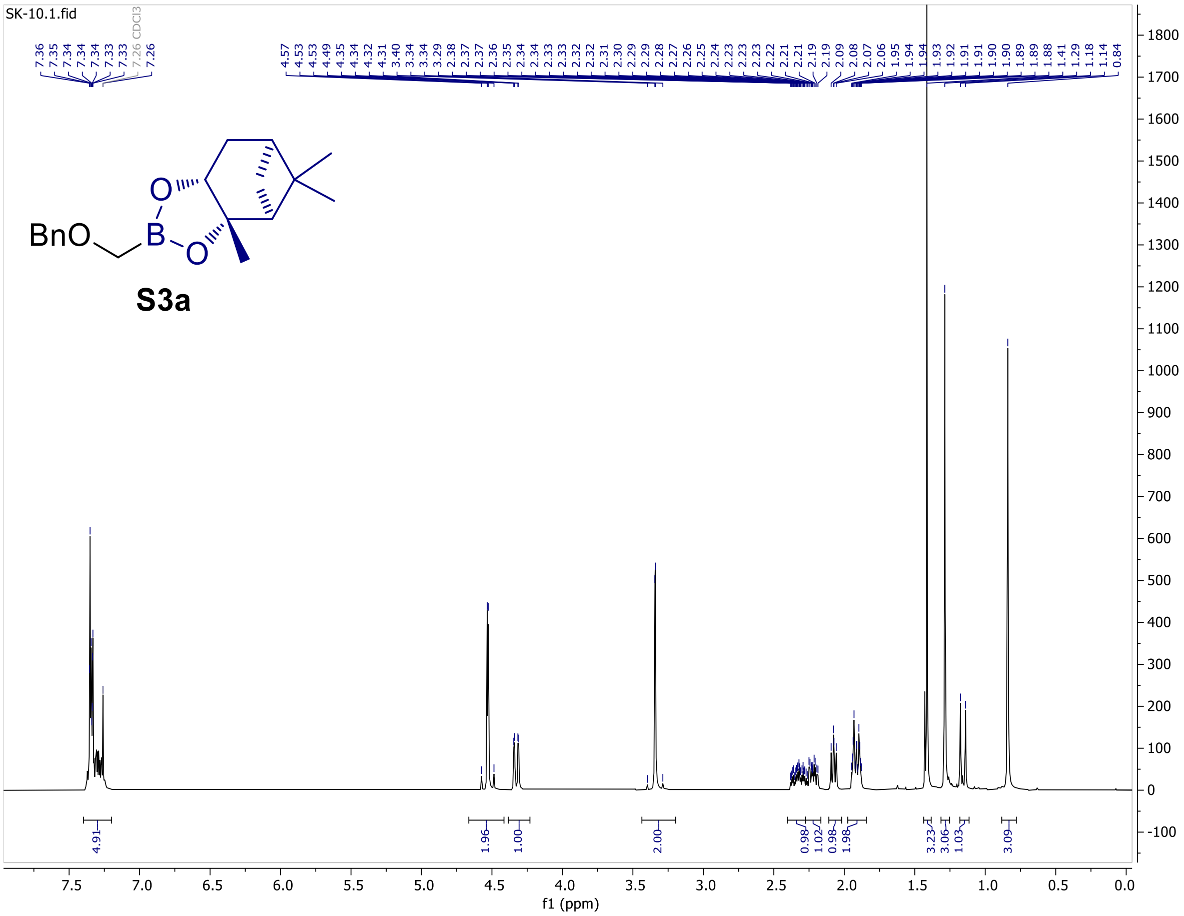
SK-10.1.fid
7.36
7.35
7.34
7.34
7.34
7.33
7.33
7.26 CDCl3
7.26
4.57
4.53
4.53
4.49
4.35
4.34
4.32
4.31
3.40
3.34
3.34
3.29
2.38
2.37
2.37
2.36
2.35
2.34
2.34
2.34
2.33
2.33
2.32
2.32
2.31
2.30
2.29
2.29
2.28
2.27
2.26
2.25
2.24
2.23
2.23
2.23
2.22
2.21
2.21
2.19
2.19
2.09
2.08
2.07
2.06
1.95
1.94
1.94
1.93
1.92
1.91
1.91
1.90
1.90
1.89
1.89
1.88
1.41
1.29
1.18
1.14
0.84
BnO
B
O
O
S3a
4.91
1.96
1.00
2.00
0.98
1.02
0.98
1.98
3.23
3.06
1.03
3.09
f1 (ppm)

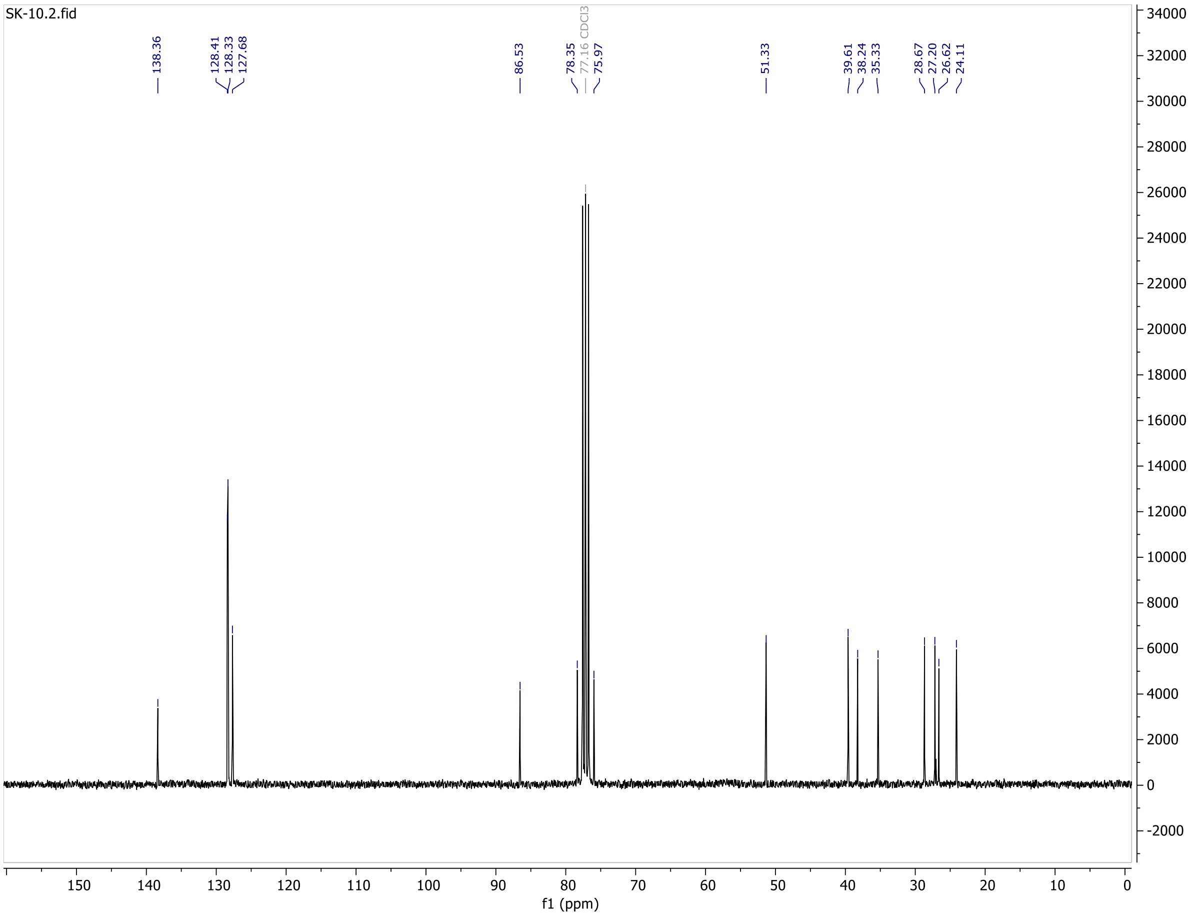
SK-10.2.fid
138.36
128.41
128.33
127.68
86.53
78.35
77.16 CDCl3
75.97
51.33
39.61
38.24
35.33
28.67
27.20
26.62
24.11
f1 (ppm)

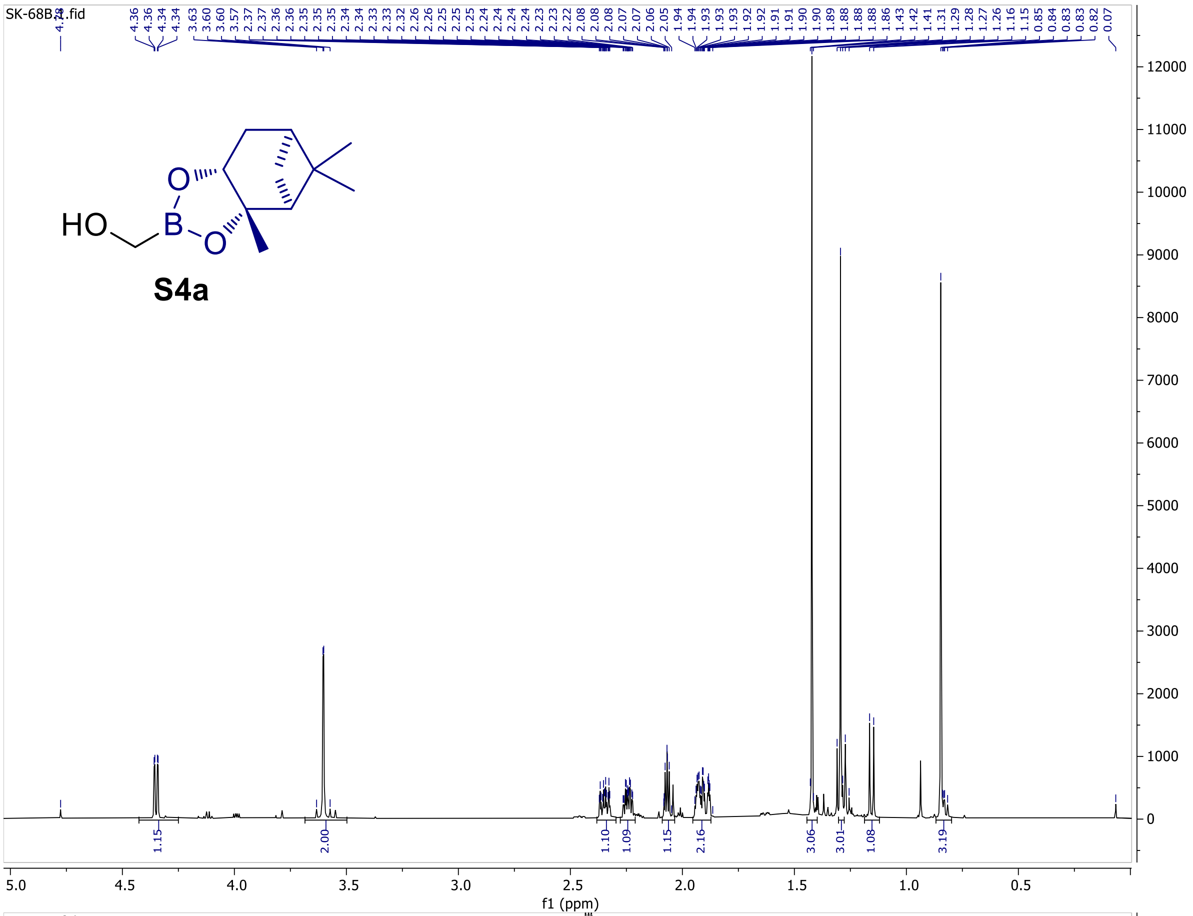
SK-68B2.fid
4.36
4.36
4.34
4.34
3.63
3.60
3.57
2.37
2.37
2.36
2.36
2.35
2.35
2.35
2.34
2.34
2.33
2.33
2.32
2.26
2.26
2.25
2.25
2.25
2.24
2.24
2.24
2.23
2.23
2.22
2.22
2.08
2.08
2.08
2.07
2.07
2.06
2.05
1.94
1.94
1.93
1.93
1.93
1.92
1.92
1.91
1.91
1.91
1.90
1.90
1.89
1.88
1.88
1.88
1.86
1.43
1.42
1.41
1.31
1.29
1.28
1.27
1.26
1.16
1.15
0.85
0.84
0.83
0.83
0.82
0.07
HO
B
O
O
S4a
1.15
2.00
1.10
1.09
1.15
2.16
3.06
3.01
1.08
3.19
f1 (ppm)

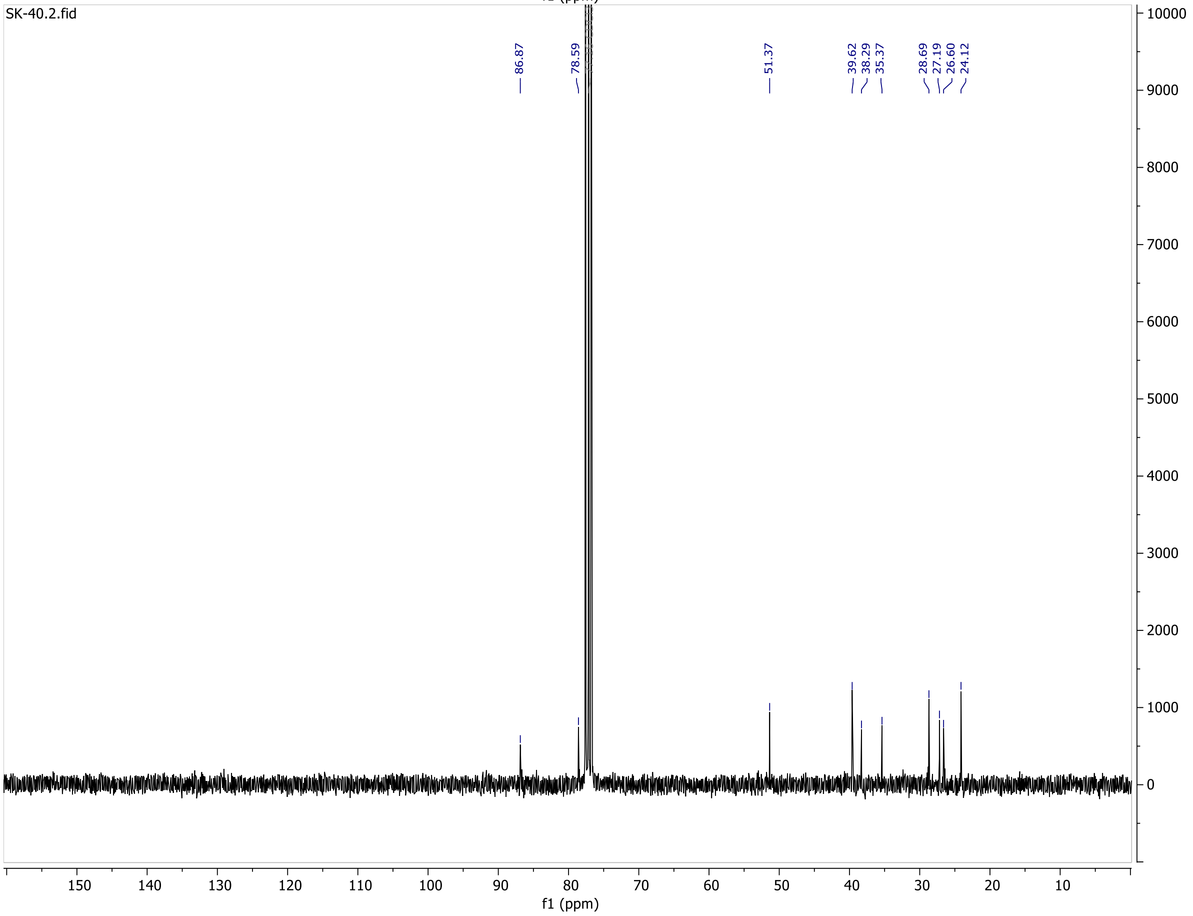
SK-40.2.fid
86.87
78.59
51.37
39.62
38.29
35.37
28.69
27.19
26.60
24.12
f1 (ppm)

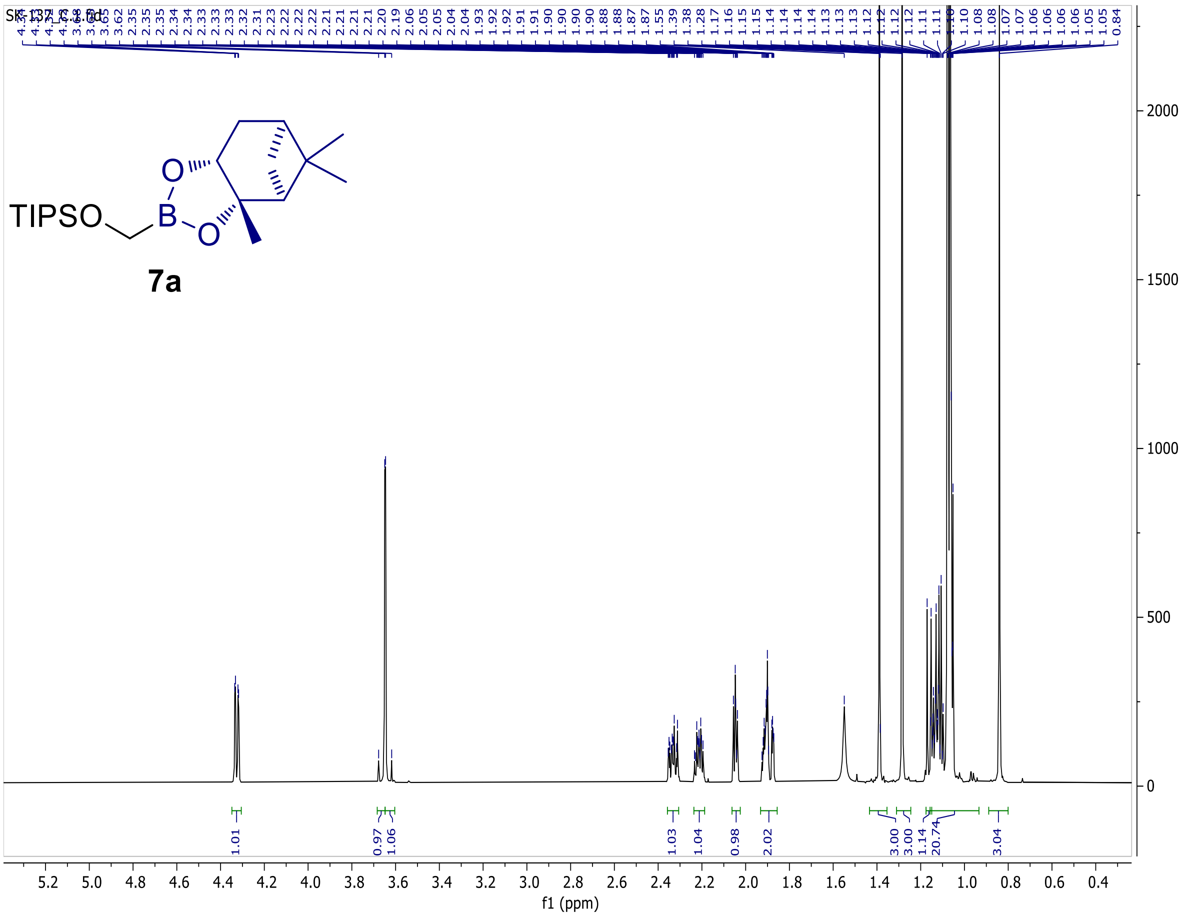
TIPSO
7a
f1 (ppm)

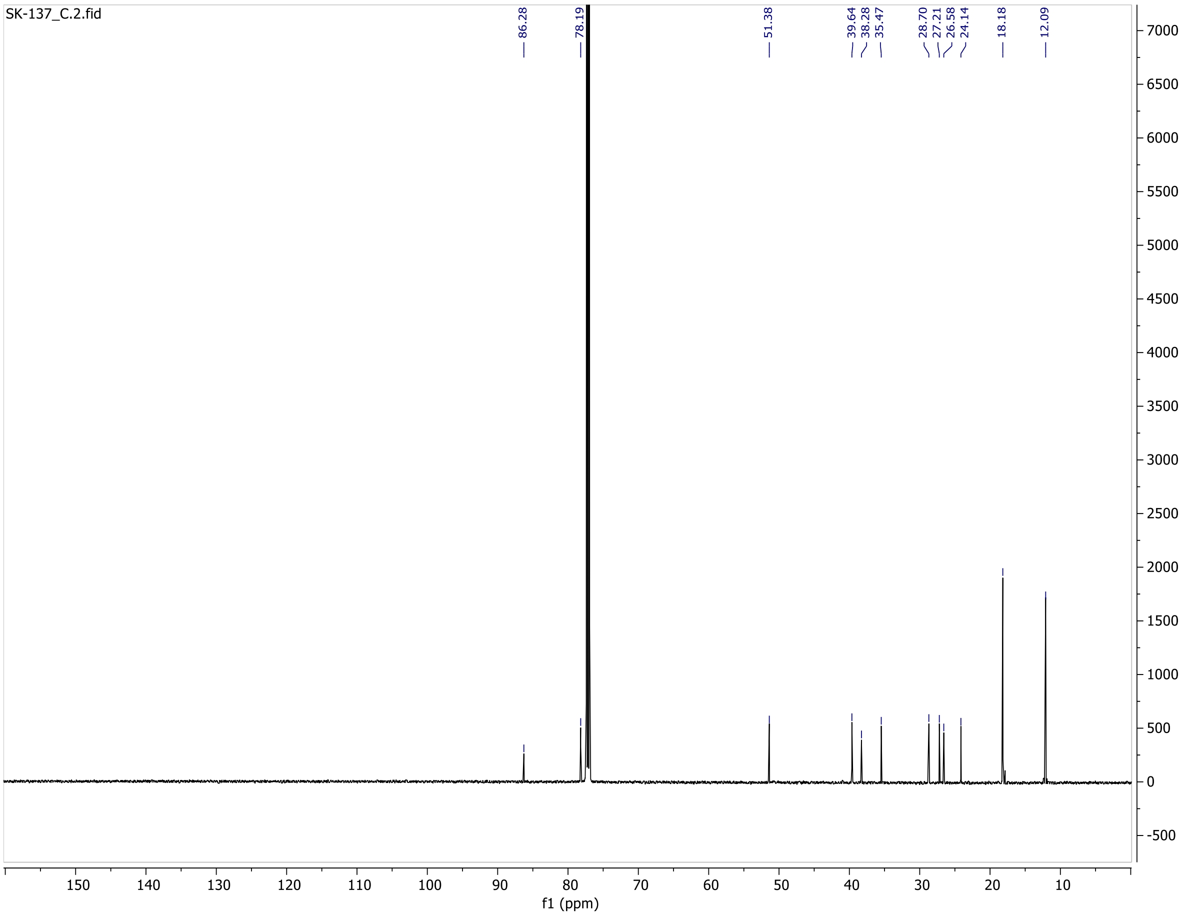
SK-137_C.2.fid
f1 (ppm)

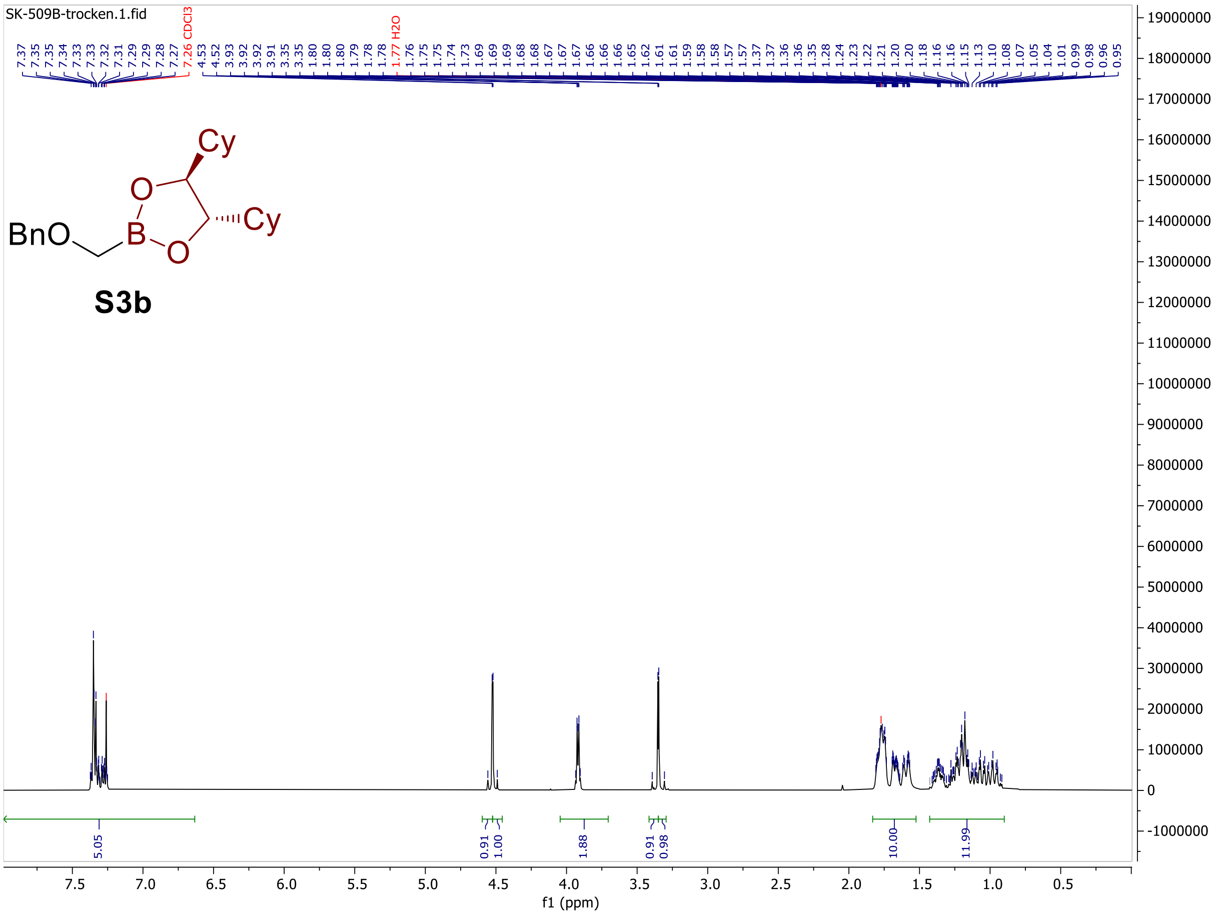
SK-509B-trocken.1.fid
7.37 7.35 7.35 7.34 7.33 7.33 7.32 7.31 7.29 7.29 7.28 7.27 7.26 CDCl3 4.53 4.52 3.93 3.92 3.92 3.91 3.35 3.35 1.80 1.80 1.80 1.79 1.78 1.78 1.77 H2O 1.76 1.75 1.75 1.74 1.73 1.69 1.69 1.69 1.68 1.67 1.67 1.67 1.66 1.66 1.65 1.62 1.61 1.61 1.59 1.58 1.58 1.57 1.37 1.37 1.36 1.36 1.35 1.28 1.24 1.23 1.22 1.21 1.20 1.20 1.18 1.16 1.16 1.15 1.13 1.10 1.08 1.07 1.05 1.04 1.01 0.99 0.98 0.96 0.95
Cy
O
BnO
B
O
Cy
S3b
5.05
0.91
1.00
1.88
0.91
0.98
10.00
11.99
f1 (ppm)

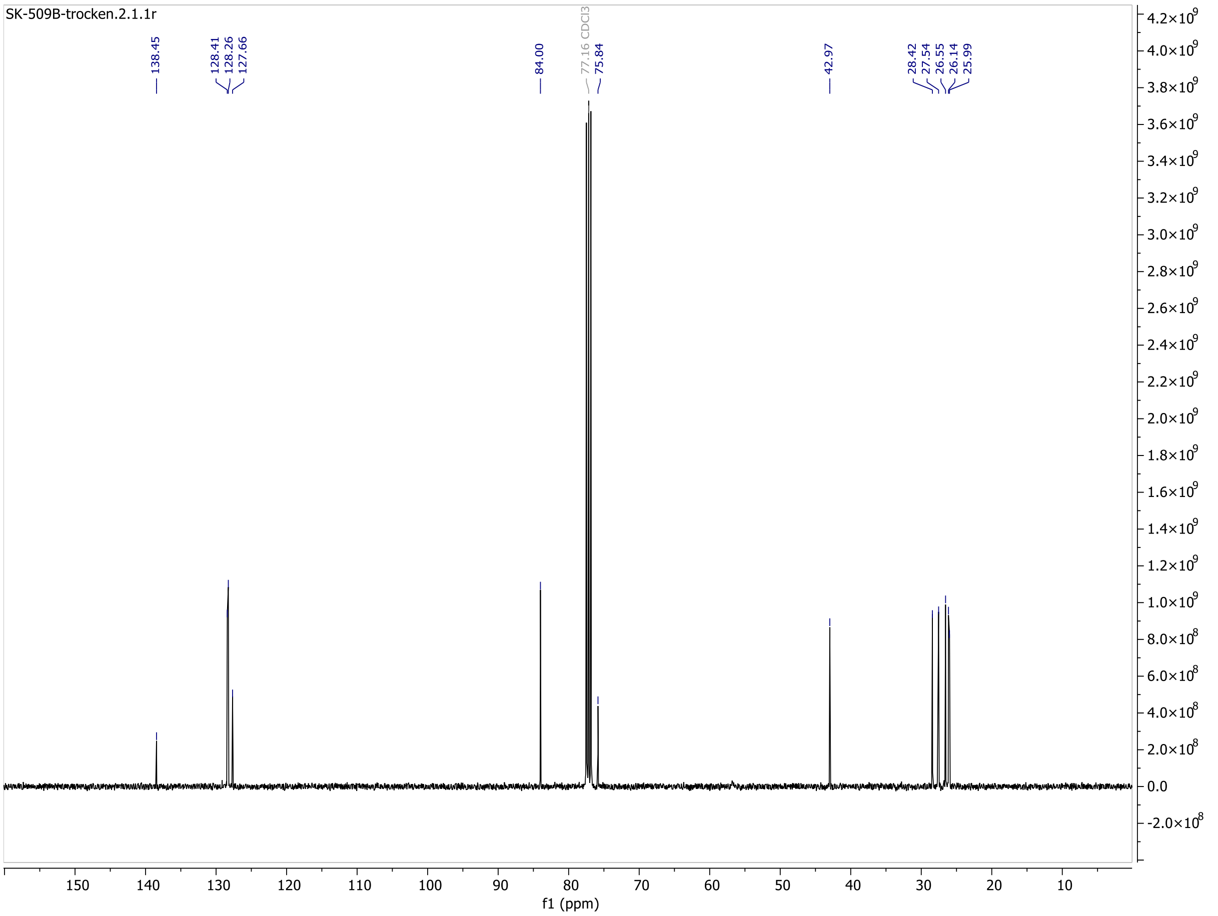
SK-509B-trocken.2.1.1r
138.45
128.41
128.26
127.66
84.00
77.16 CDCl3
75.84
42.97
28.42
27.54
26.55
26.14
25.99
f1 (ppm)

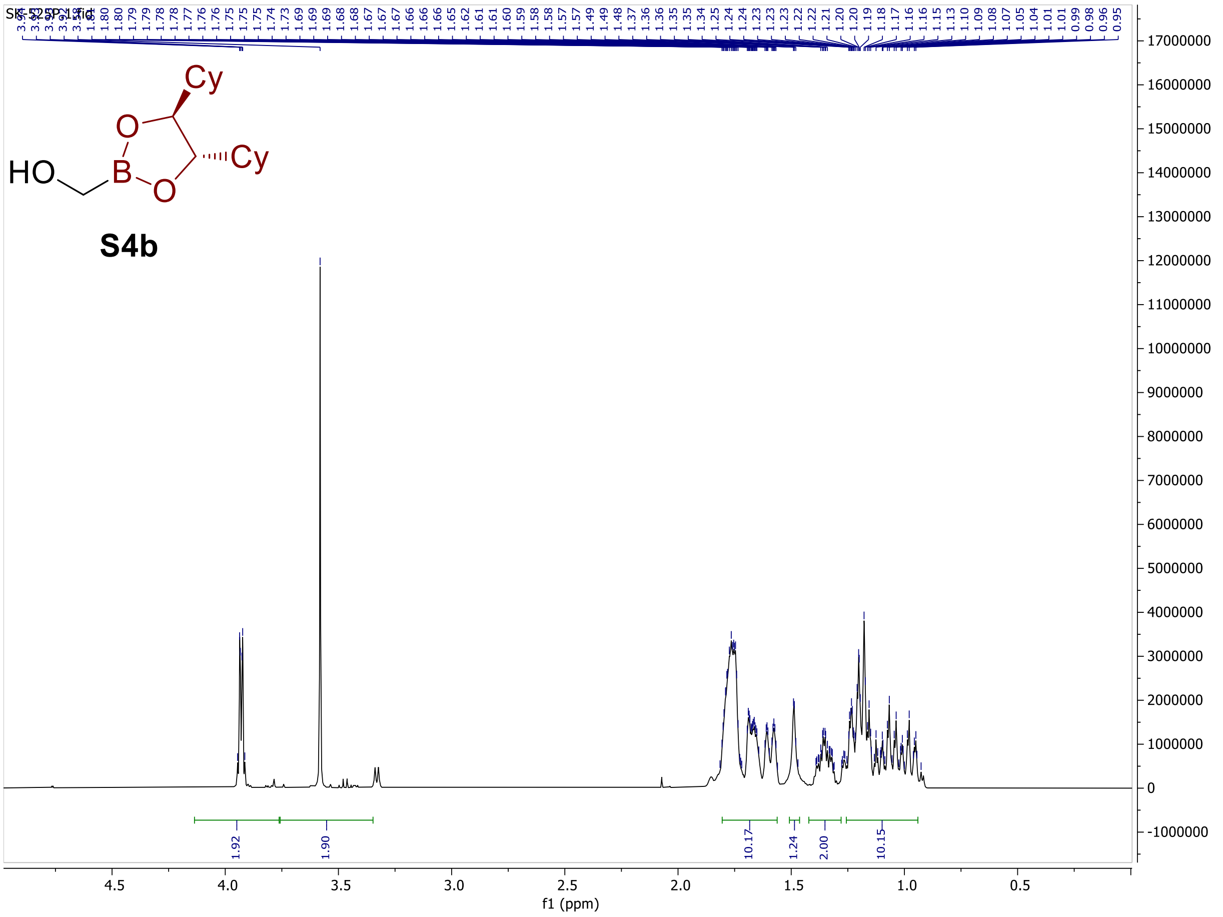
SK-525P.1.fid
Cy
O
HO
B
O
Cy
S4b
1.92
1.90
10.17
1.24
2.00
10.15
f1 (ppm)

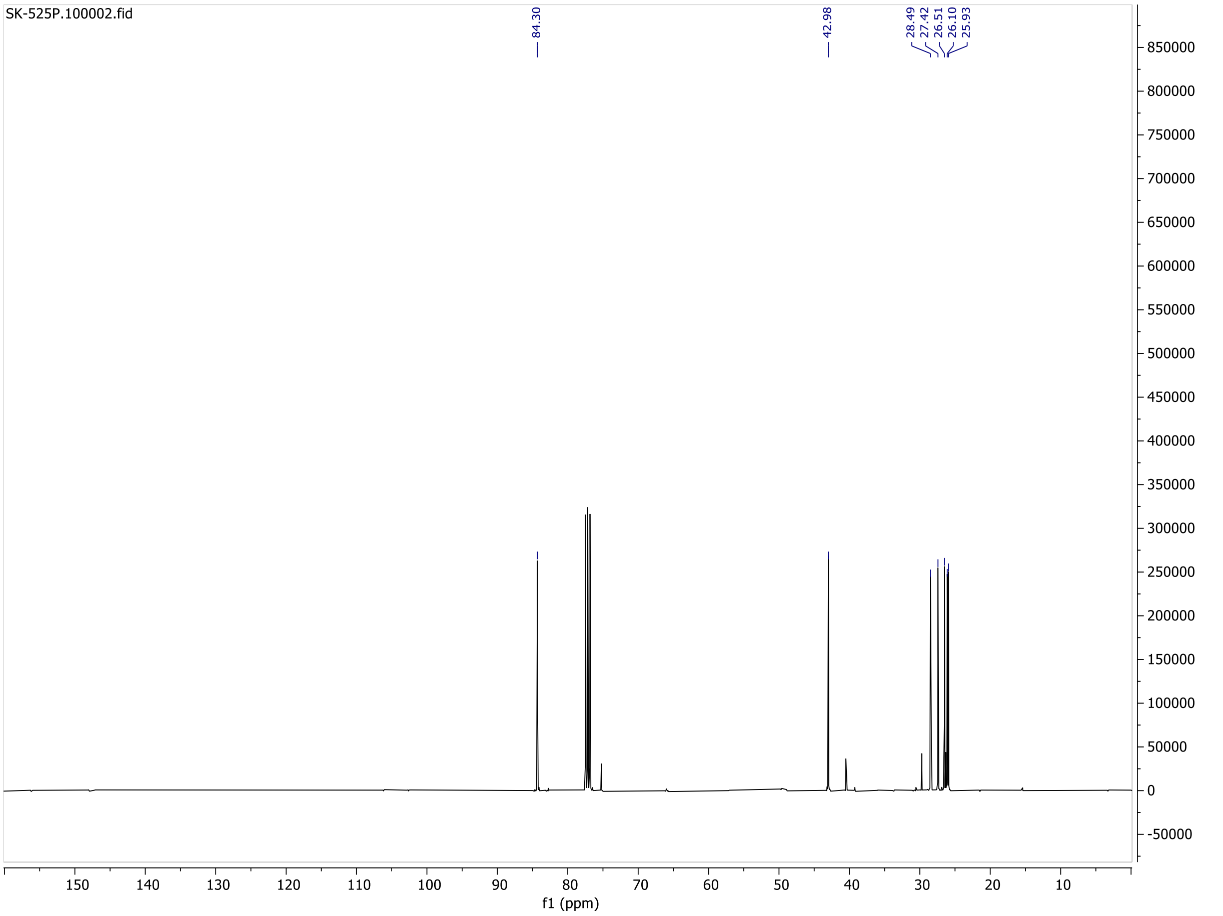
SK-525P.100002.fid
84.30
42.98
28.49
27.42
26.51
26.10
25.93
f1 (ppm)

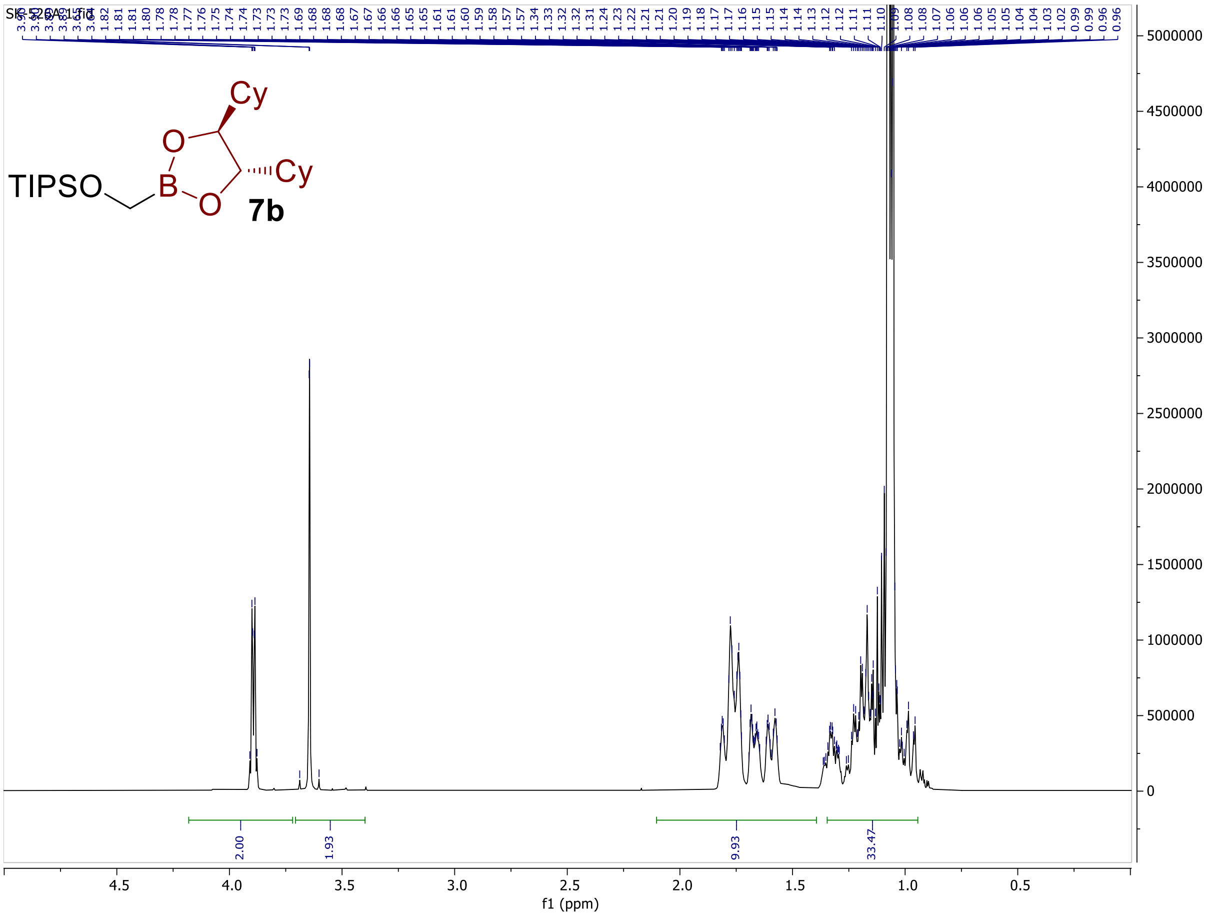

TIPSO
B
O
O
Cy
Cy
7b
f1 (ppm)
2.00
1.93
9.93
33.47

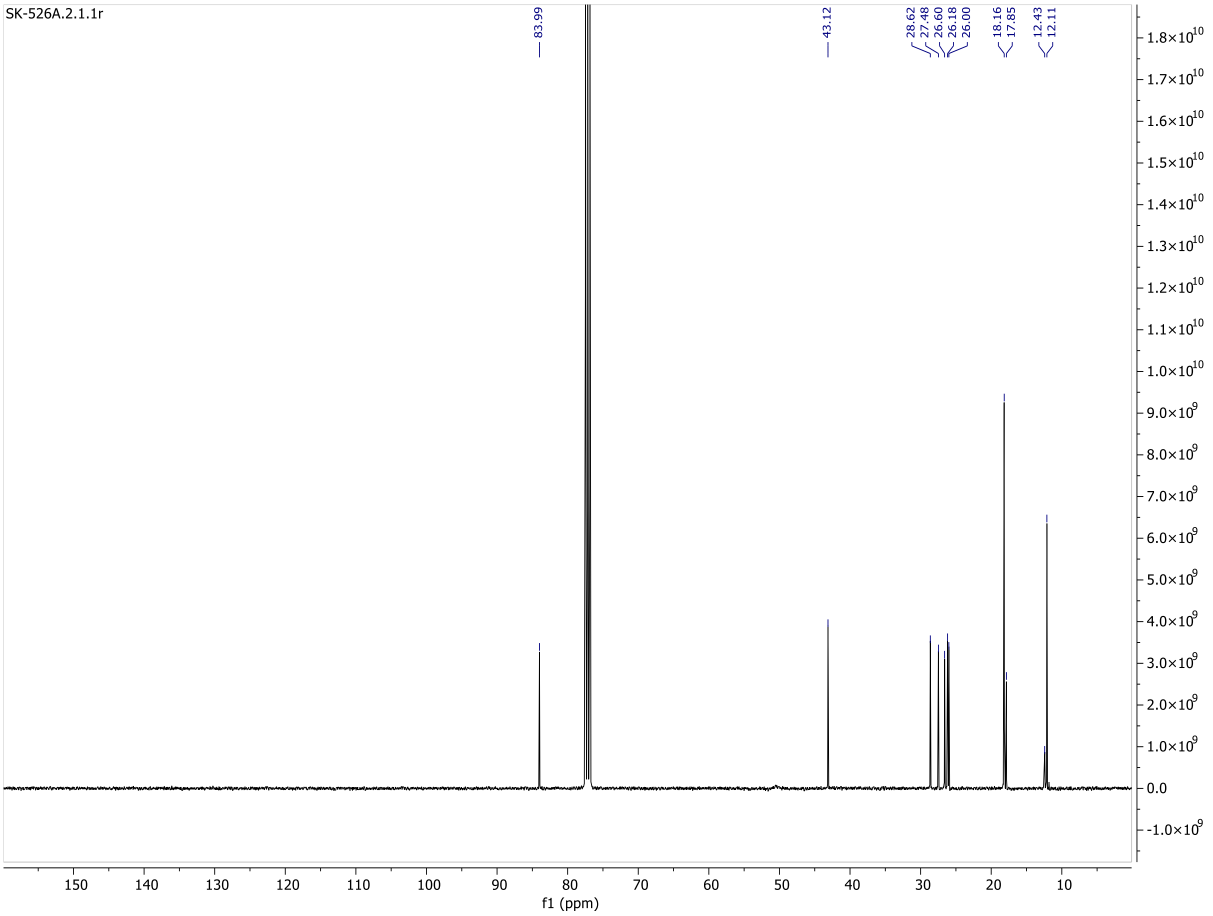

SK-526A.2.1.1r
83.99
43.12
28.62
27.48
26.60
26.18
26.00
18.16
17.85
12.43
12.11
f1 (ppm)

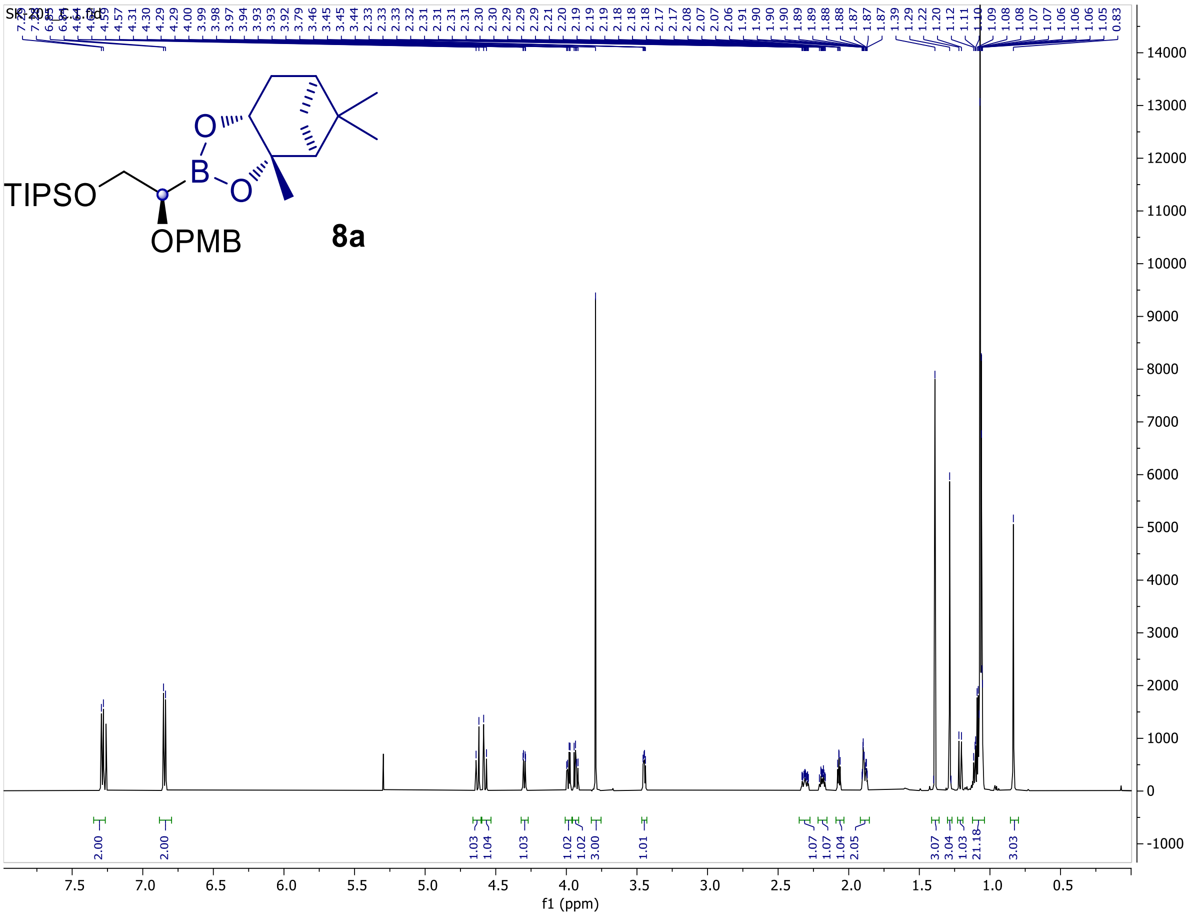
SK-201_C.1.fid
TIPSO
OPMB
8a
f1 (ppm)

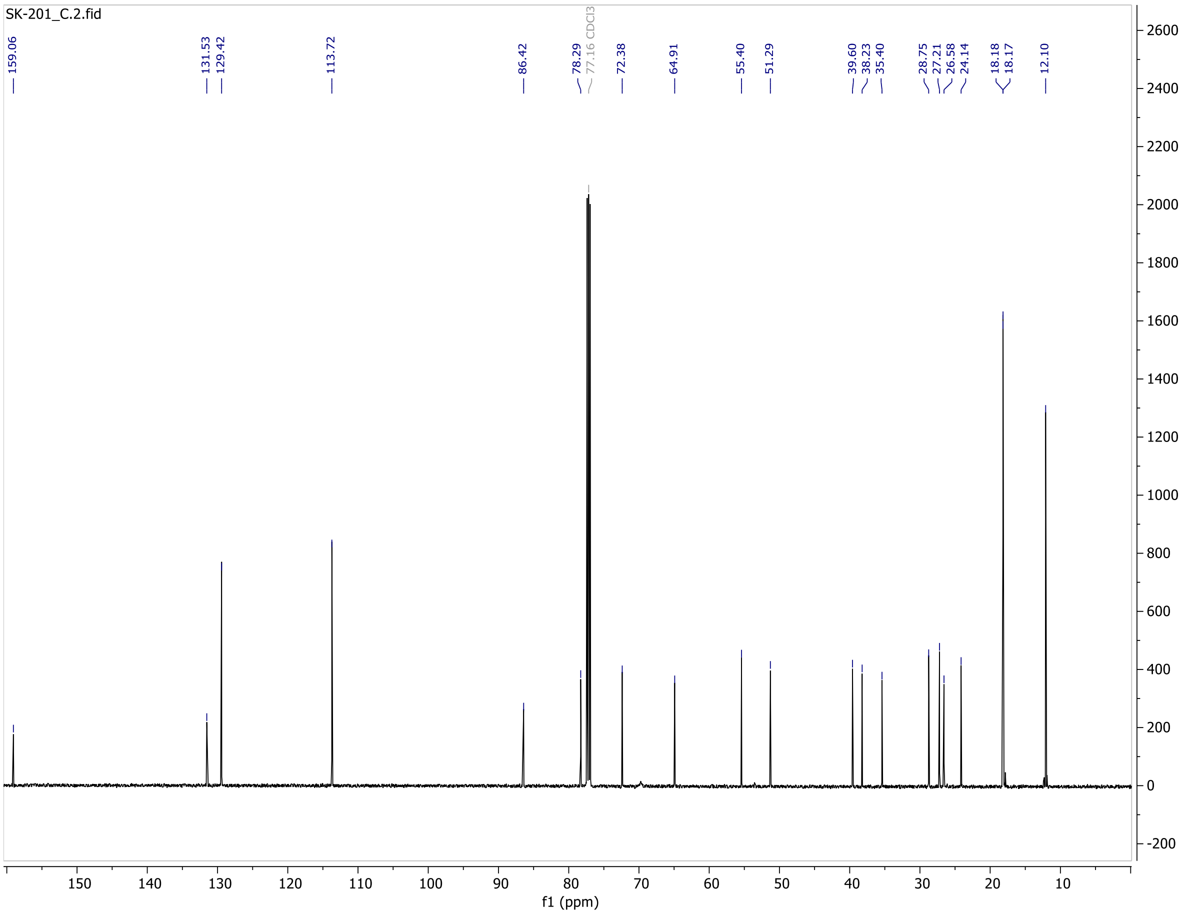
SK-201_C.2.fid
159.06
131.53
129.42
113.72
86.42
78.29
77.16 CDCl3
72.38
64.91
55.40
51.29
39.60
38.23
35.40
28.75
27.21
26.58
24.14
18.18
18.17
12.10
f1 (ppm)

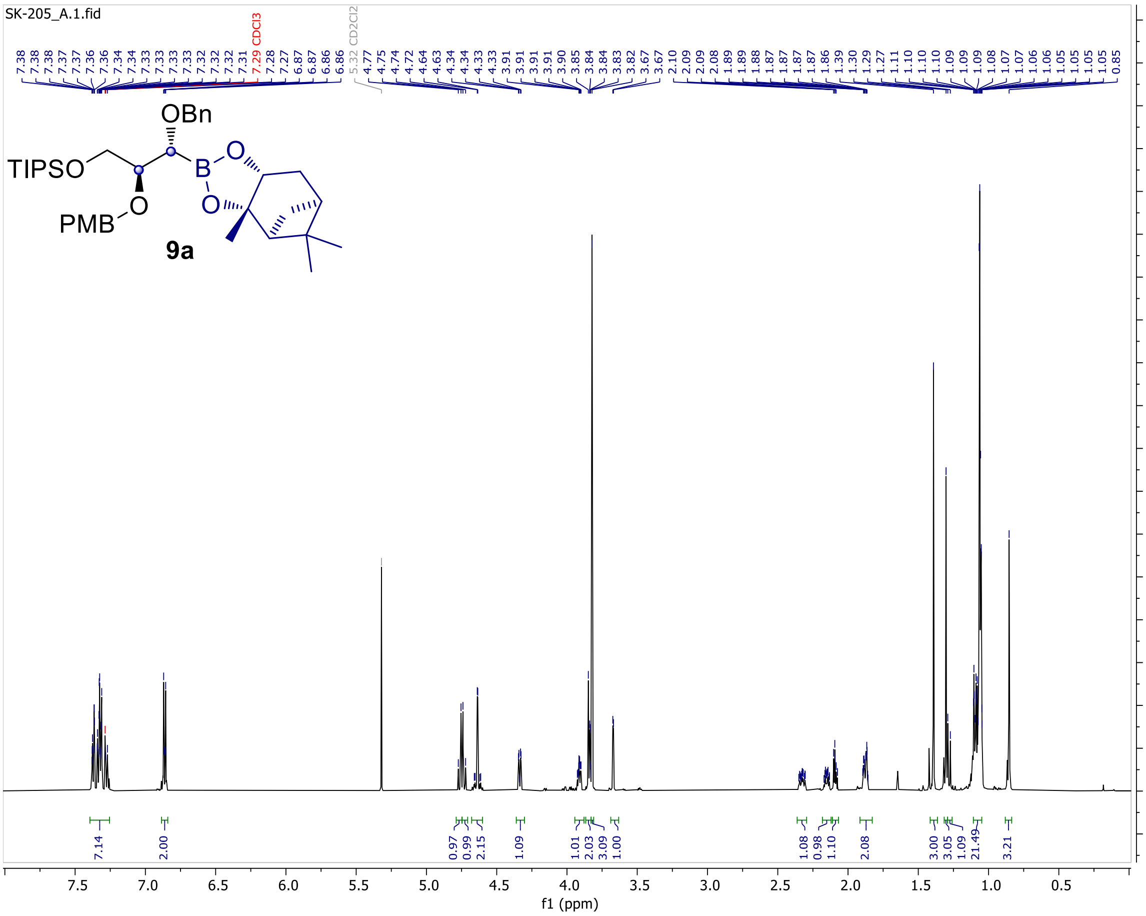
SK-205_A.1.fid
7.38 7.38 7.38 7.37 7.37 7.36 7.36 7.34 7.34 7.33 7.33 7.33 7.33 7.32 7.32 7.31 7.29 CDCl3 7.28 7.27 6.87 6.87 6.86 6.86
5.32 CD2Cl2
4.77 4.75 4.74 4.72 4.64 4.63 4.34 4.34 4.33 3.91 3.91 3.91 3.90 3.85 3.84 3.84 3.83 3.82 3.67 3.67 2.10 2.09 2.08 1.89 1.88 1.87 1.87 1.87 1.86 1.39 1.30 1.29 1.27 1.11 1.10 1.10 1.10 1.09 1.09 1.09 1.08 1.07 1.07 1.06 1.05 1.05 1.05 1.05 0.85
OBn
TIPSO
B
O
O
PMB
O
9a
7.14 2.00 0.97 0.99 2.15 1.09 1.01 2.03 3.09 1.00 1.08 0.98 1.10 2.08 3.00 3.05 1.09 21.49 3.21
f1 (ppm)

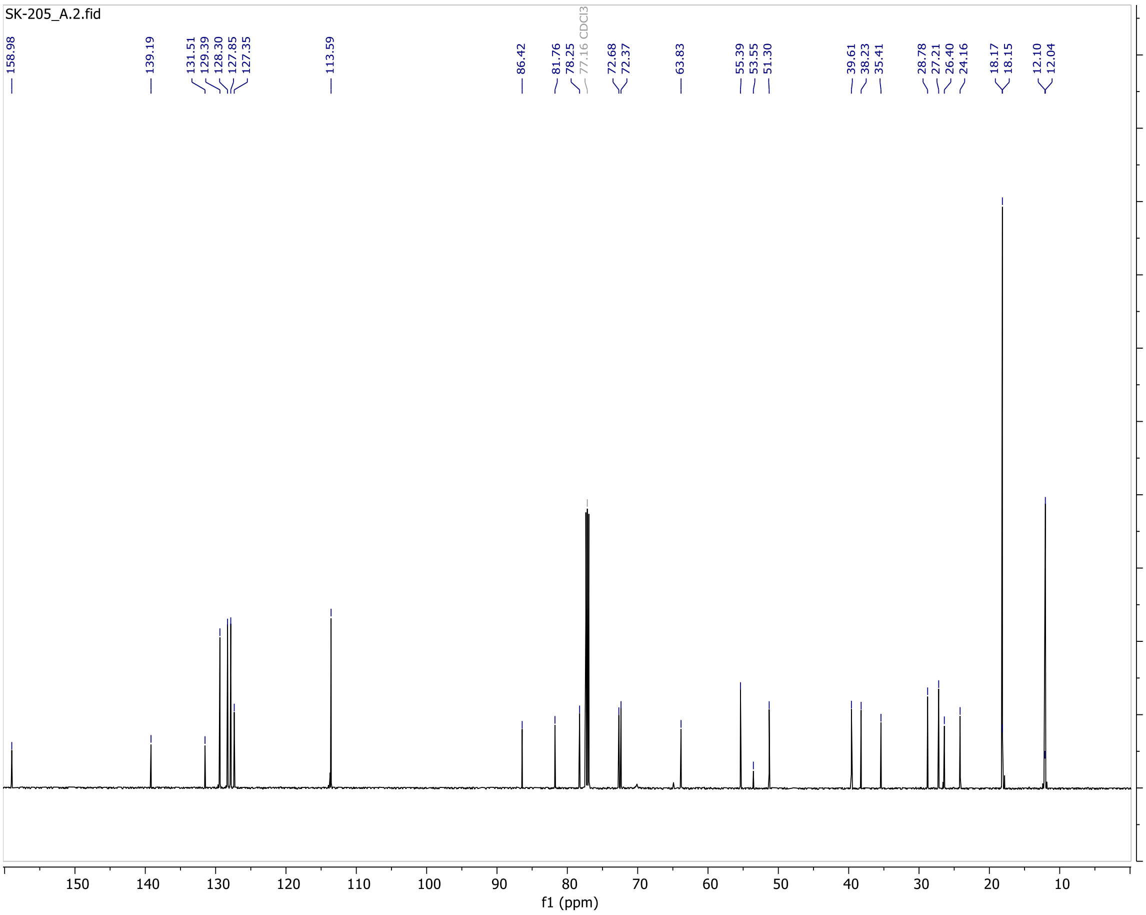
SK-205_A.2.fid
158.98 139.19 131.51 129.39 128.30 127.85 127.35 113.59 86.42 81.76 78.25 77.16 CDCl3 72.68 72.37 63.83 55.39 53.55 51.30 39.61 38.23 35.41 28.78 27.21 26.40 24.16 18.17 18.15 12.10 12.04
f1 (ppm)

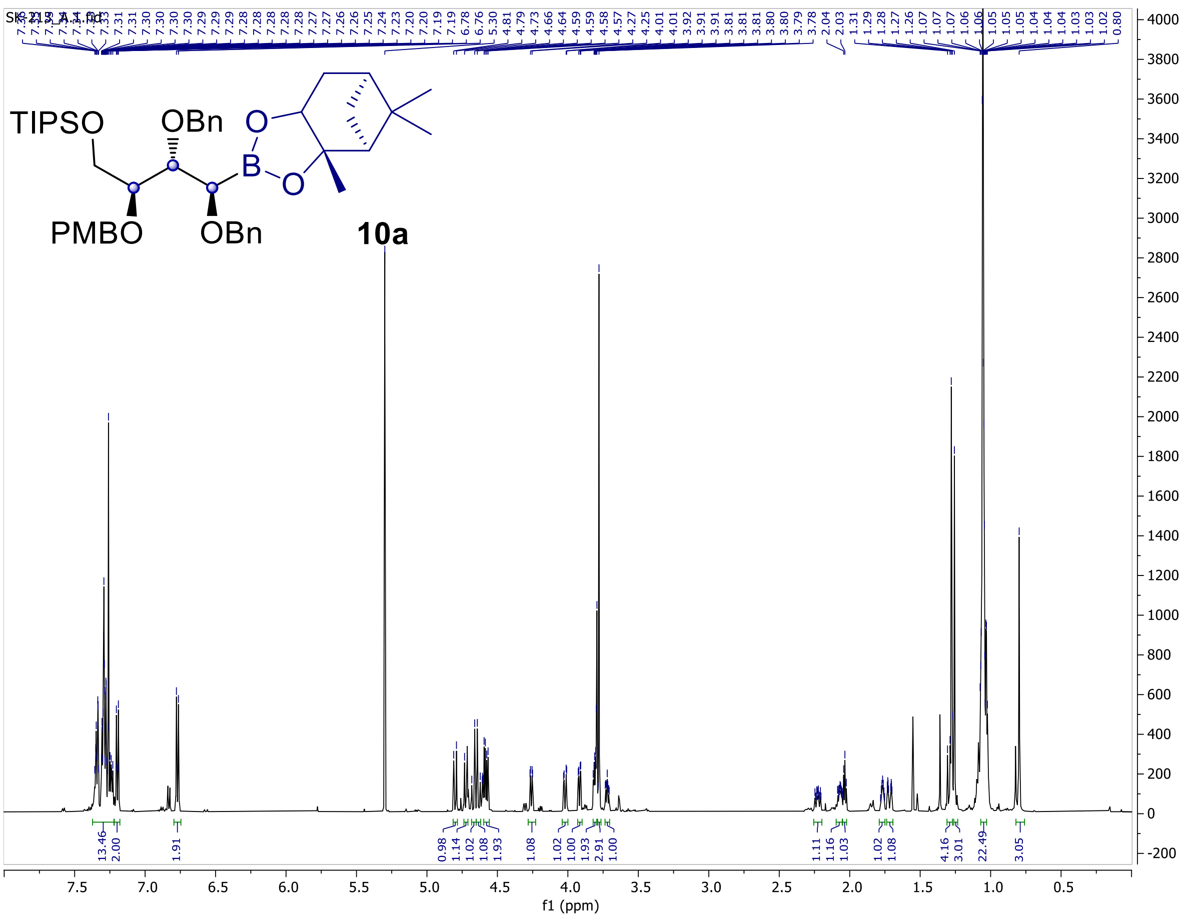

SK-213_A.1.fid
TIPSO
OBn
PMBO
OBn
O
B
O
10a
13.46
2.00
1.91
0.98
1.14
1.02
1.08
1.93
1.08
1.02
1.00
1.93
2.91
1.00
1.11
1.16
1.03
1.02
1.08
4.16
3.01
22.49
3.05
f1 (ppm)

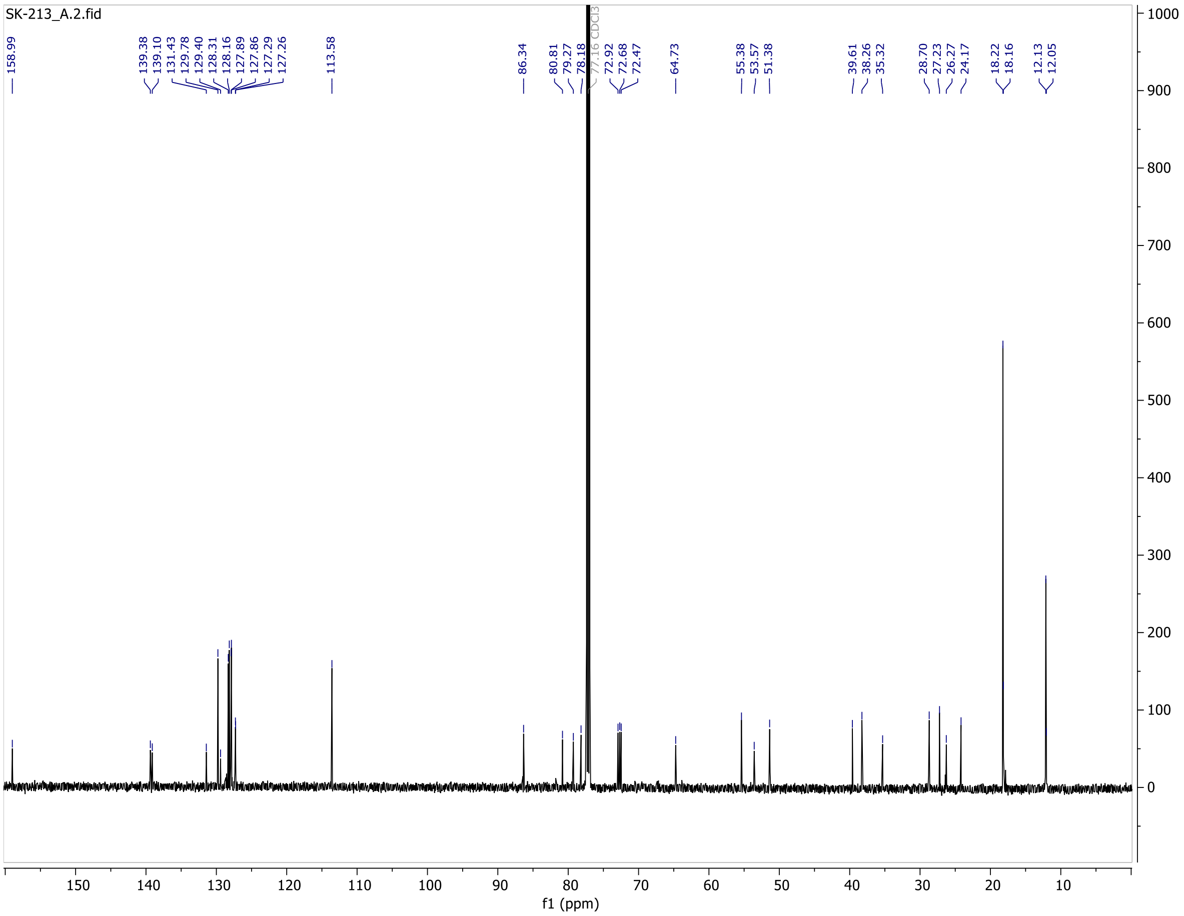

SK-213_A.2.fid
158.99
139.38
139.10
131.43
129.78
129.40
128.31
128.16
127.89
127.86
127.29
127.26
113.58
86.34
80.81
79.27
78.18
77.16 CDCl3
72.92
72.68
72.47
64.73
55.38
53.57
51.38
39.61
38.26
35.32
28.70
27.23
26.27
24.17
18.22
18.16
12.13
12.05
f1 (ppm)

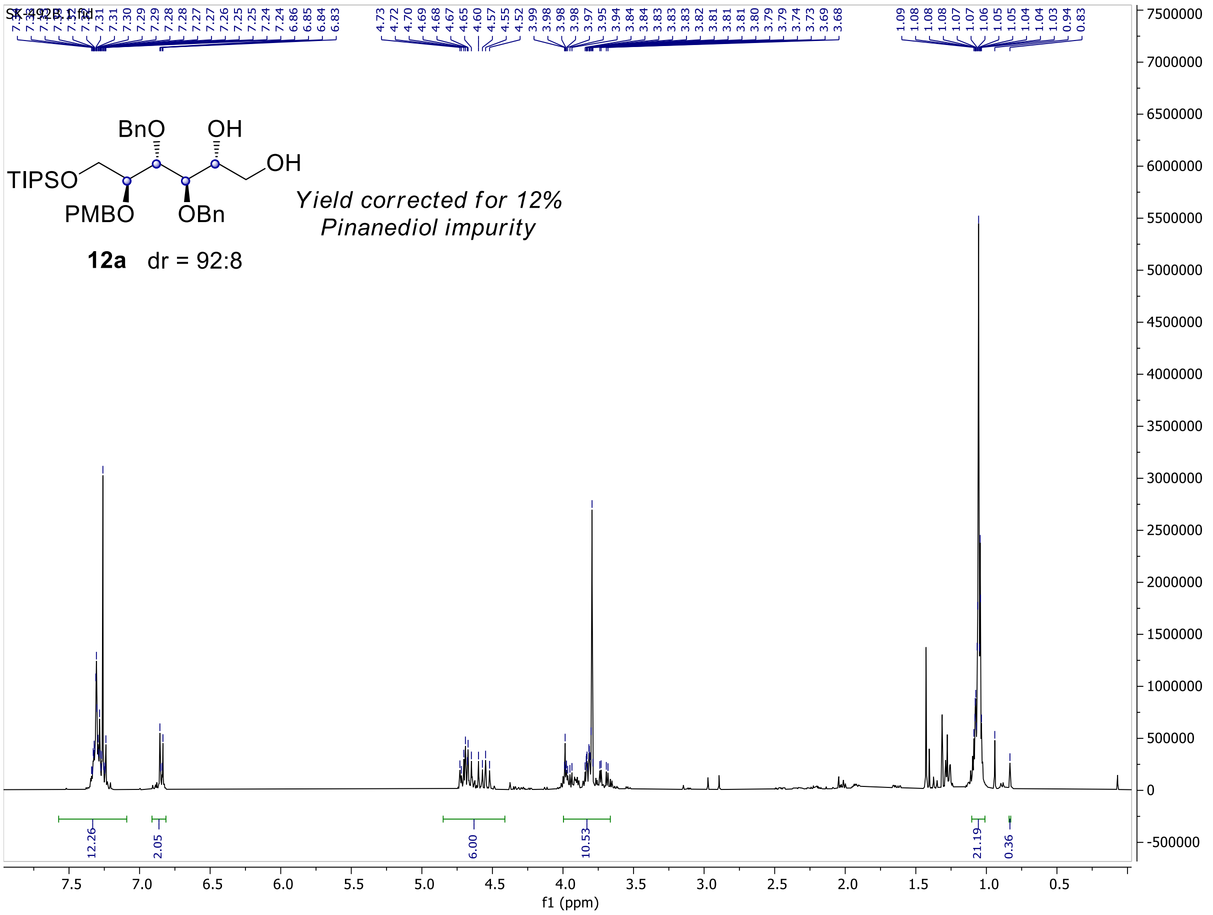
7.31 7.30 7.29 7.29 7.28 7.28 7.27 7.27 7.26 7.25 7.25 7.24 7.24 6.86 6.85 6.84 6.83
4.73 4.72 4.70 4.69 4.68 4.67 4.65 4.60 4.57 4.55 4.52 3.99 3.98 3.98 3.98 3.97 3.95 3.94 3.84 3.83 3.83 3.83 3.82 3.81 3.81 3.81 3.80 3.79 3.79 3.74 3.73 3.69 3.68
1.09 1.08 1.08 1.08 1.07 1.07 1.06 1.05 1.05 1.04 1.04 1.03 0.94 0.83
BnO
OH
OH
TIPSO
PMBO
OBn
12a dr = 92:8
Yield corrected for 12% Pinanediol impurity
12.26
2.05
6.00
10.53
21.19
0.36
f1 (ppm)

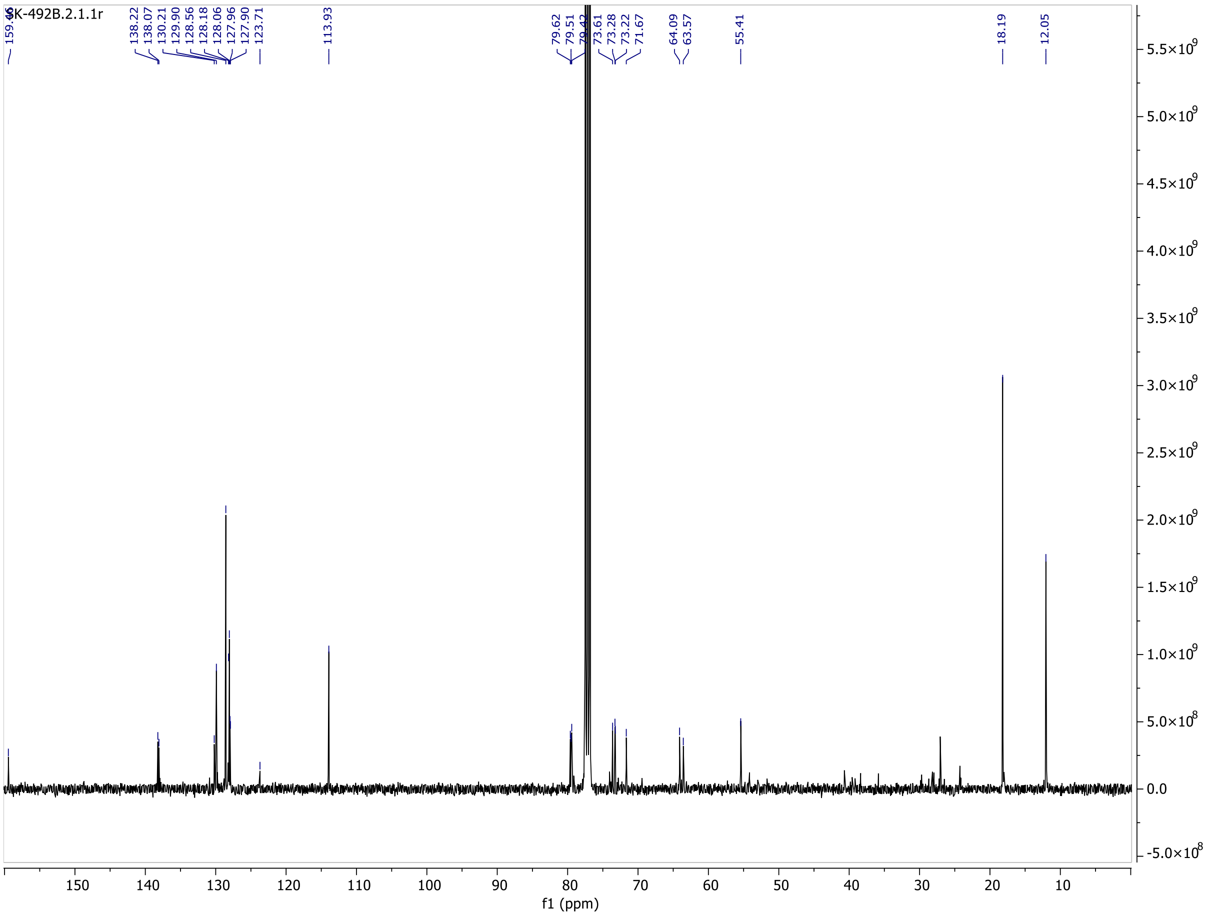
SK-492B.2.1.1r
138.22 138.07 130.21 129.90 128.56 128.18 128.06 127.96 127.90 123.71
113.93
79.62 79.51
73.61 73.28 73.22 71.67
64.09 63.57
55.41
18.19
12.05
f1 (ppm)

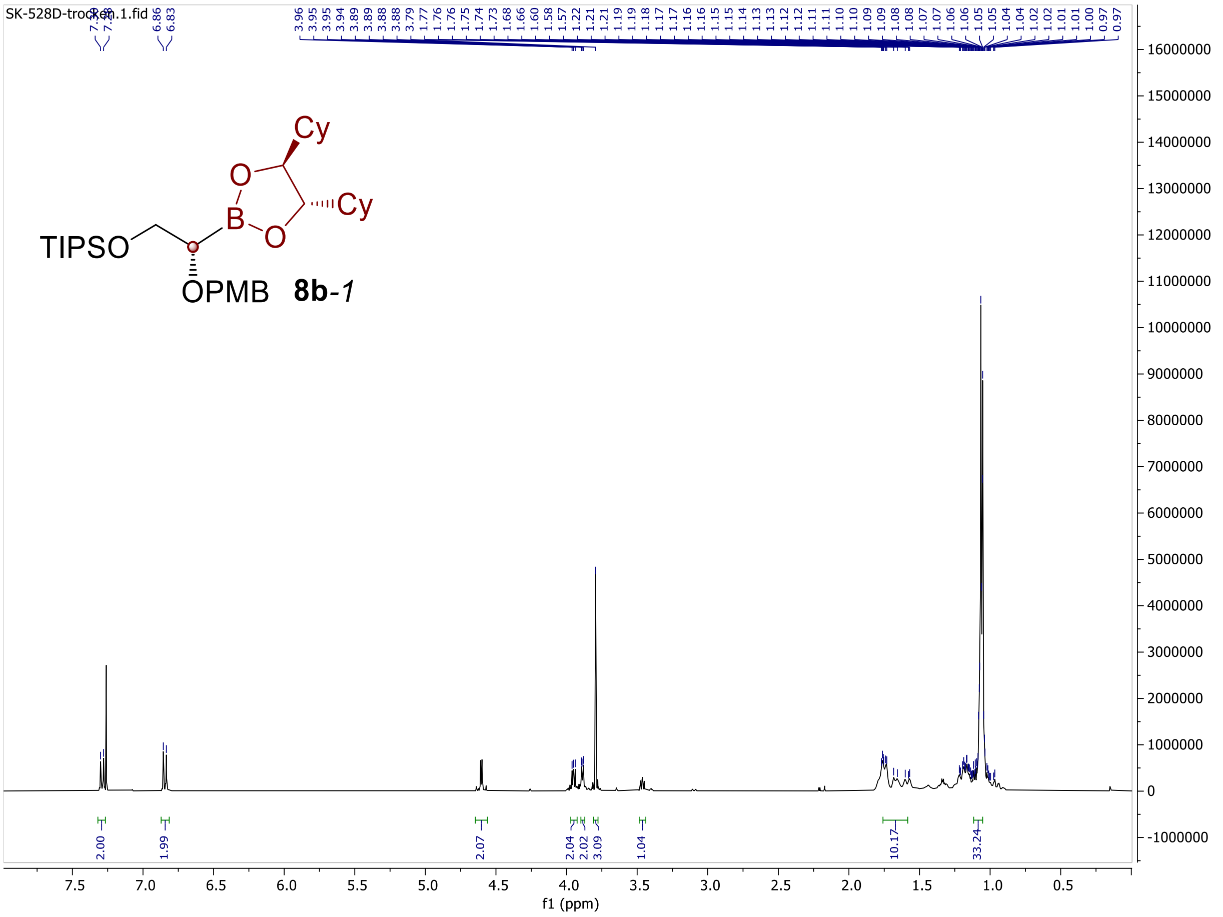

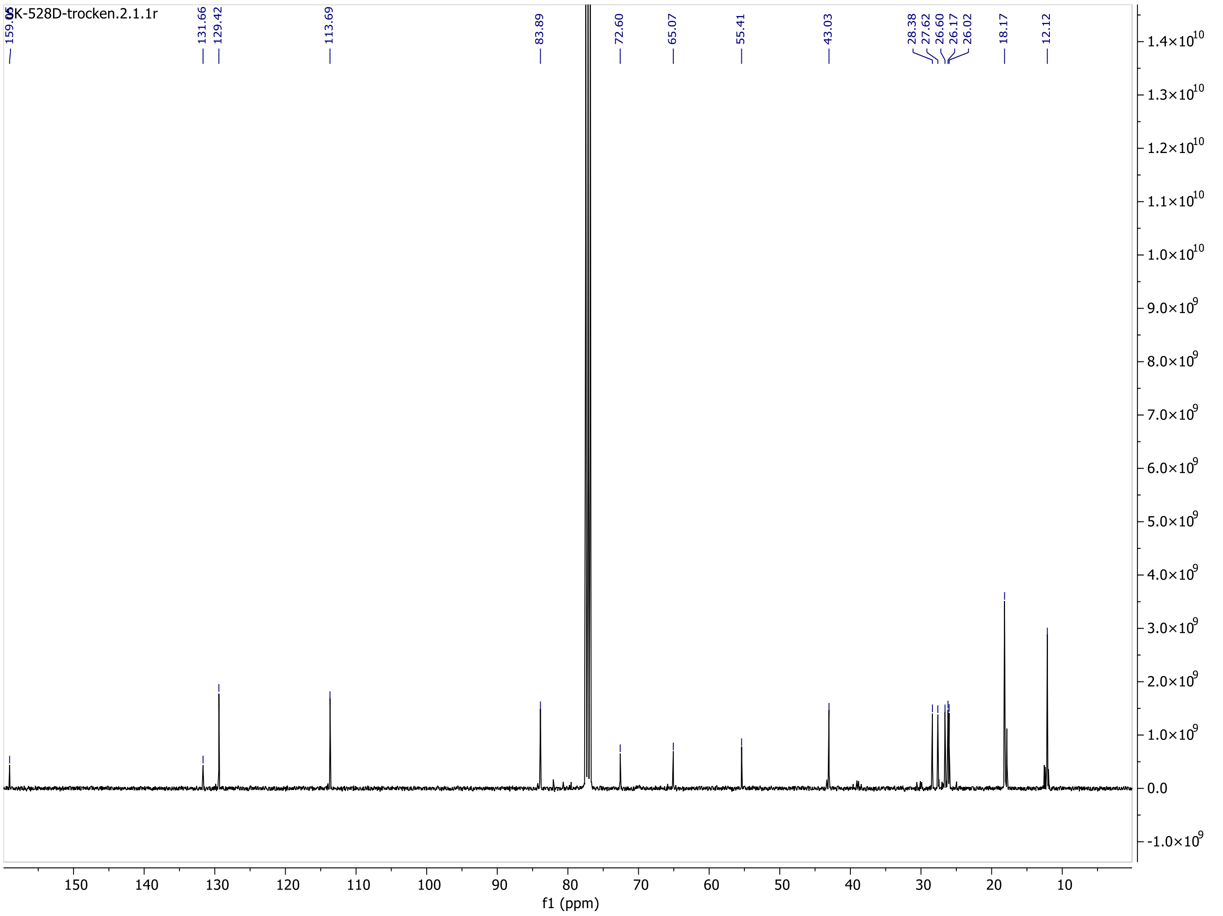

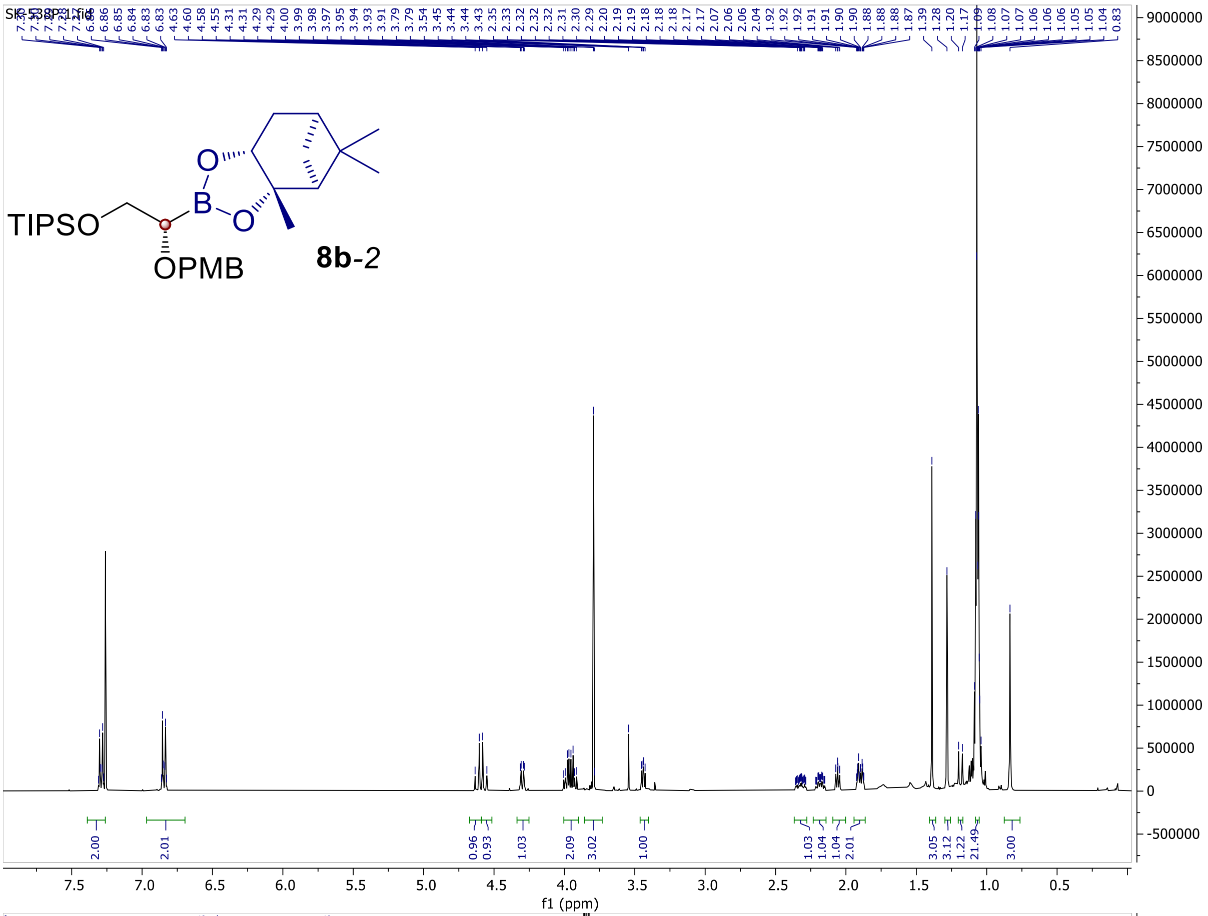
TIPSO
B
O
O
OPMB
8b-2
4.63 4.60 4.58 4.55 4.31 4.31 4.29 4.29 4.00 3.99 3.98 3.97 3.95 3.94 3.93 3.91 3.79 3.79 3.54 3.45 3.44 3.44 3.43 2.35 2.33 2.32 2.32 2.32 2.31 2.30 2.29 2.20 2.19 2.19 2.18 2.18 2.18 2.17 2.17 2.07 2.06 2.06 2.04 1.92 1.92 1.92 1.91 1.91 1.90 1.88 1.88 1.88 1.87 1.39 1.28 1.20 1.17 1.09 1.08 1.07 1.07 1.06 1.06 1.06 1.05 1.05 1.04 0.83
6.86 6.85 6.84 6.83 6.83
2.00 2.01 0.96 0.93 1.03 2.09 3.02 1.00 1.03 1.04 1.04 2.01 3.05 3.12 1.22 21.49 3.00
f1 (ppm)

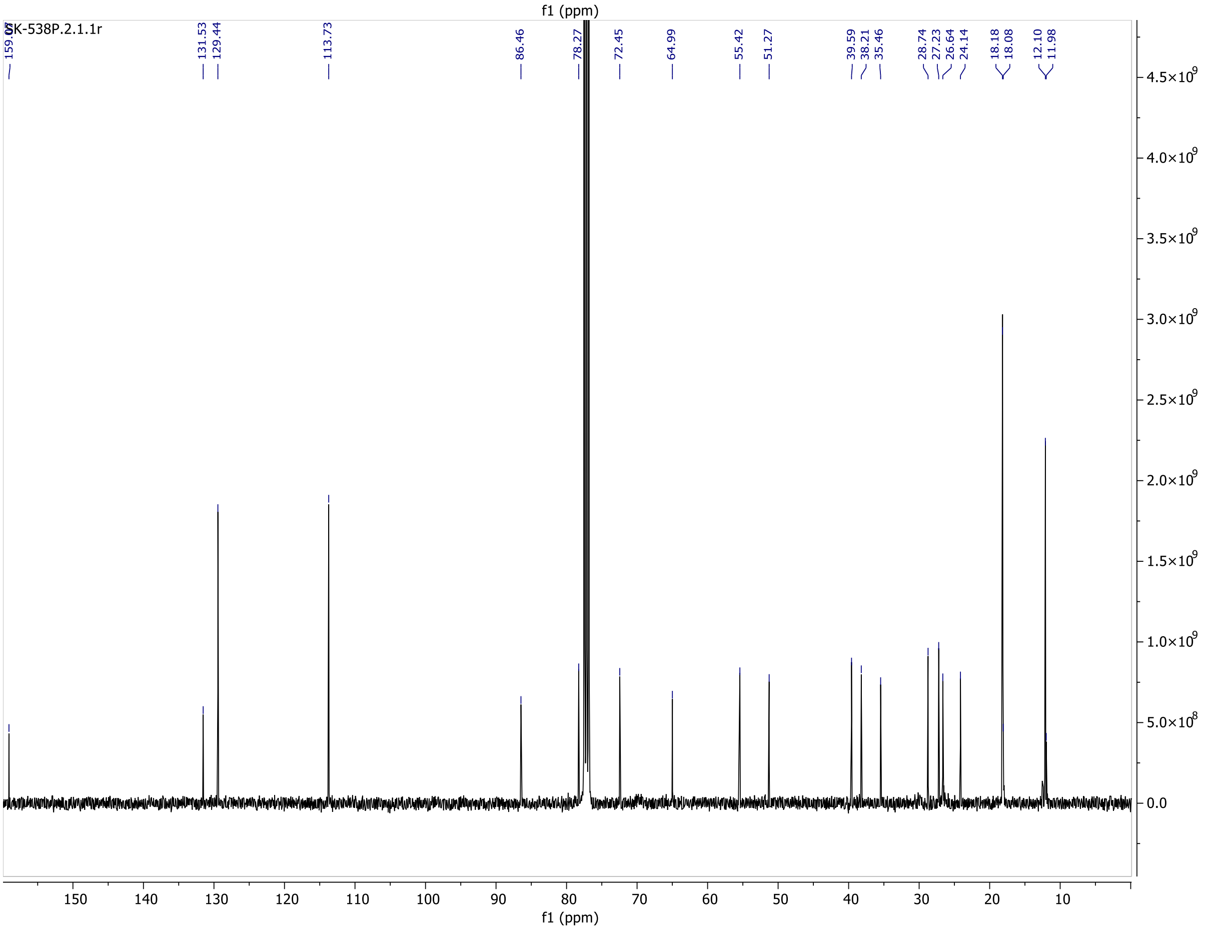
SK-538P.2.1.1r
131.53 129.44 113.73 86.46 78.27 72.45 64.99 55.42 51.27 39.59 38.21 35.46 28.74 27.23 26.64 24.14 18.18 18.08 12.10 11.98
f1 (ppm)

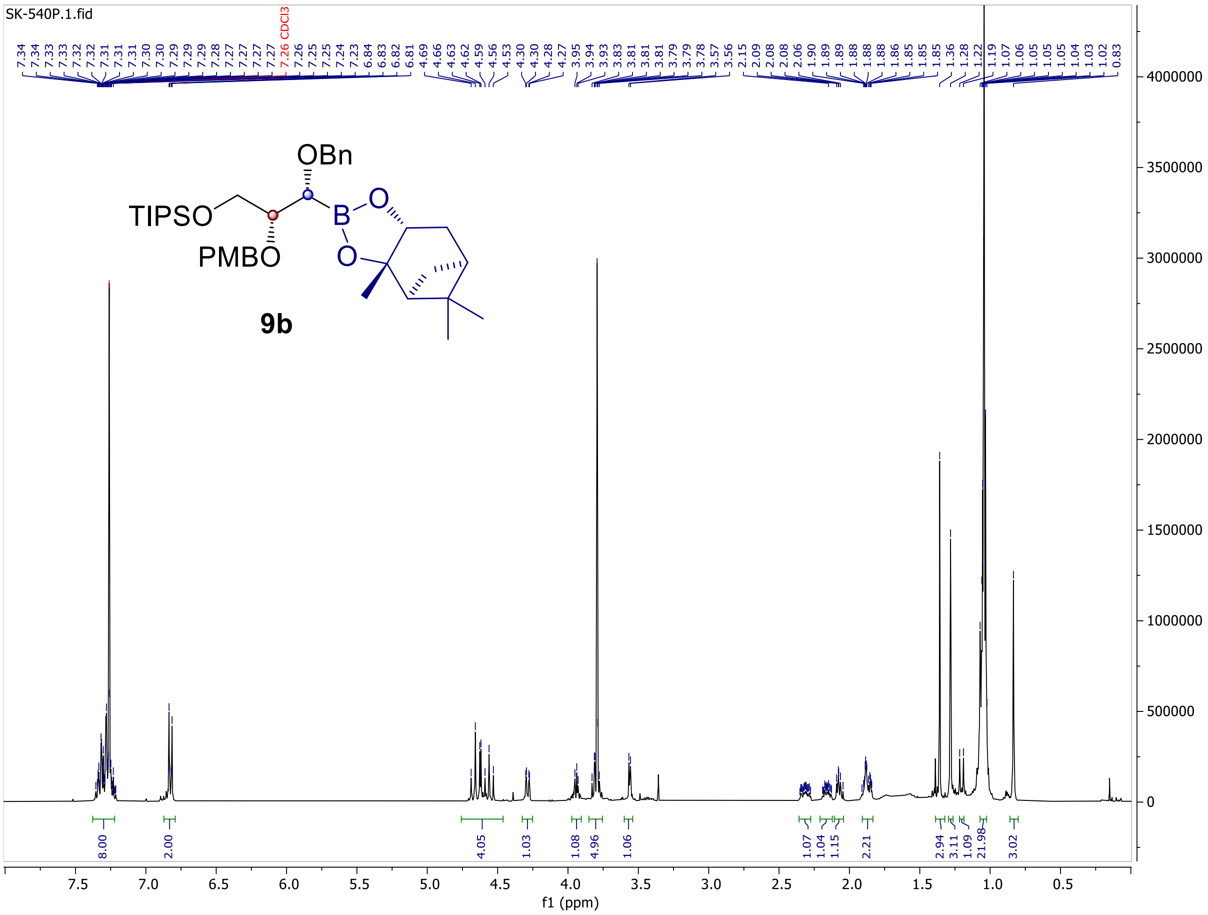

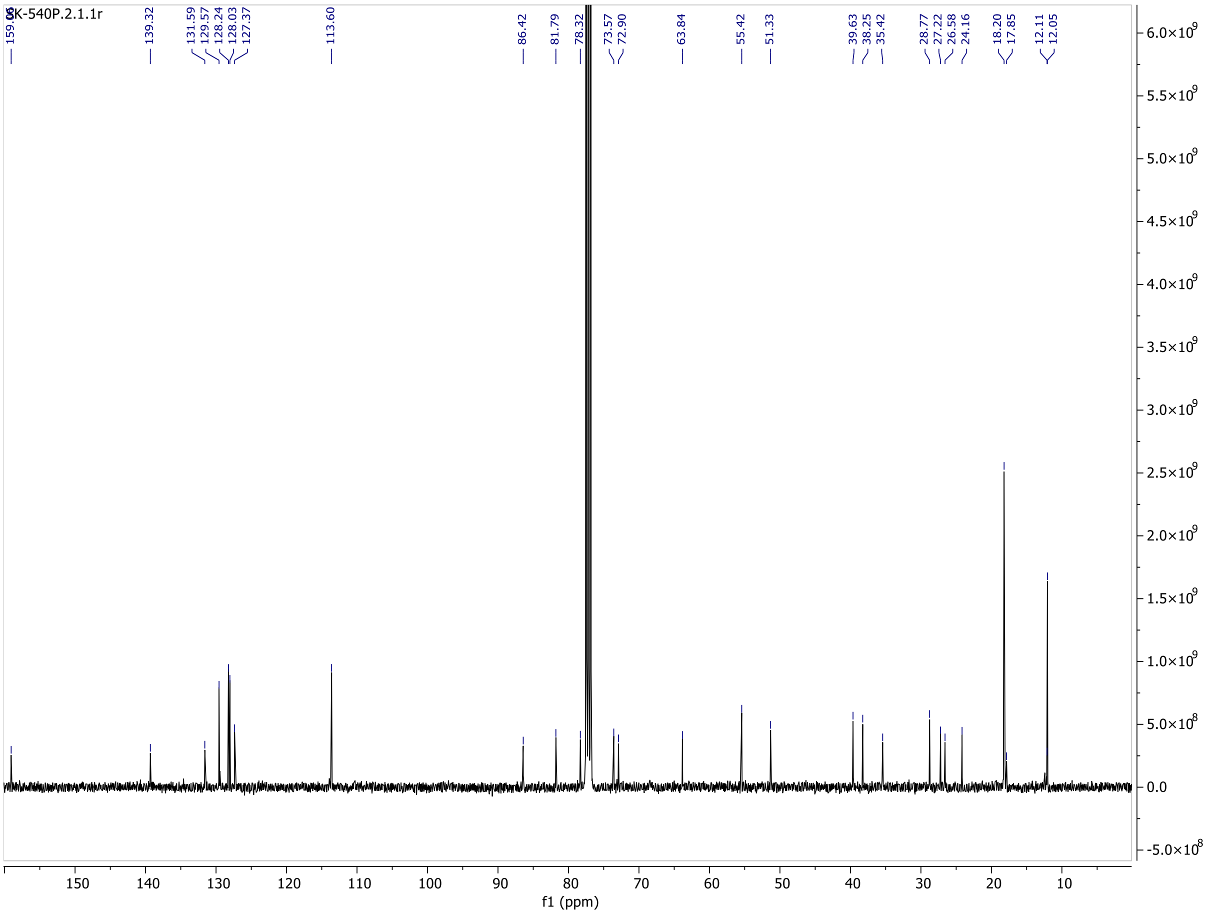

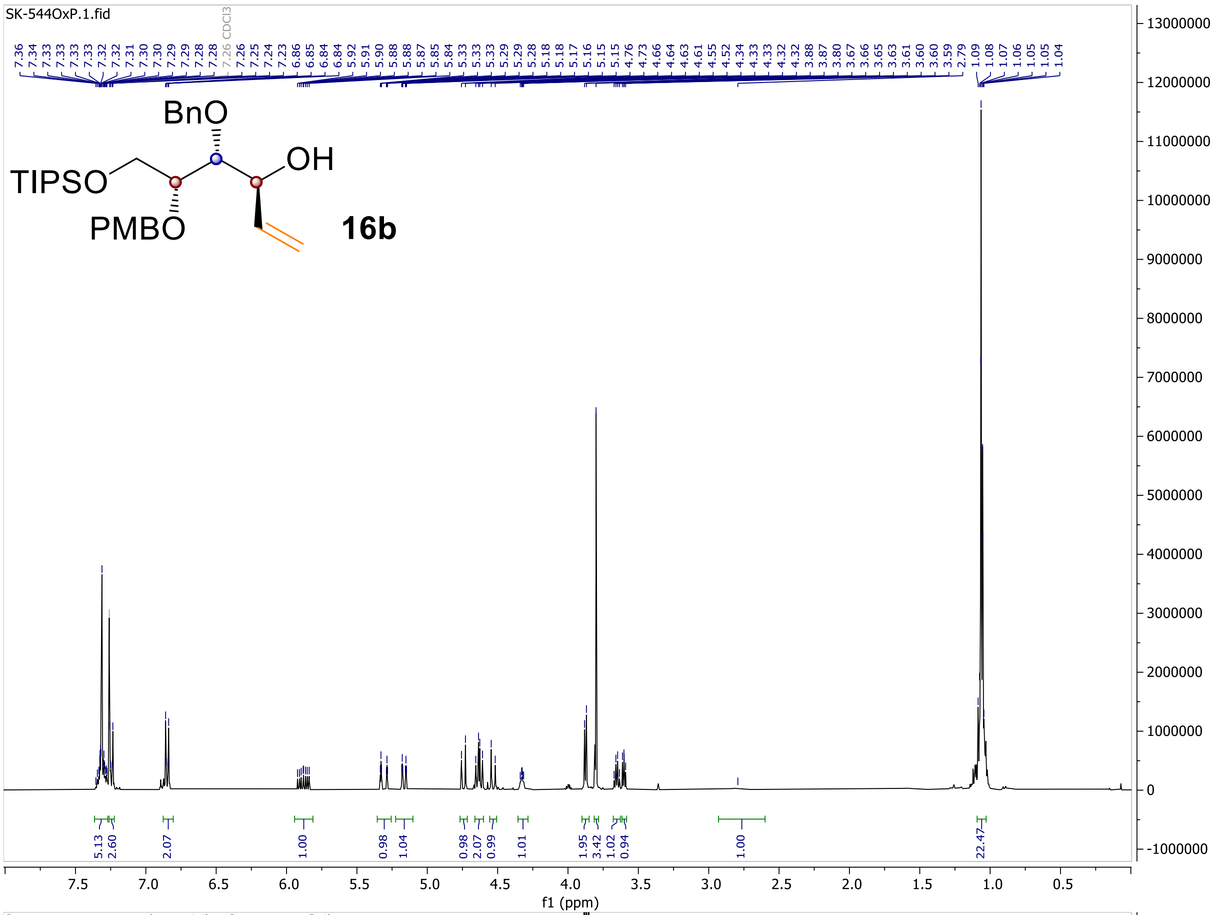
SK-544OxP.1.fid
7.36 7.34 7.33 7.33 7.33 7.32 7.32 7.31 7.30 7.30 7.29 7.29 7.28 7.26 CDCl3 7.26 7.25 7.24 7.23 6.86 6.85 6.84 6.84 5.92 5.91 5.90 5.88 5.88 5.87 5.85 5.84 5.33 5.33 5.33 5.29 5.29 5.28 5.18 5.18 5.17 5.16 5.15 5.15 4.76 4.73 4.66 4.64 4.63 4.61 4.55 4.52 4.34 4.33 4.33 4.32 4.32 3.88 3.87 3.80 3.67 3.66 3.65 3.63 3.61 3.60 3.60 3.59 2.79 1.09 1.08 1.07 1.06 1.05 1.05 1.04
BnO
OH
TIPSO
PMBO
16b
5.13 2.60 2.07 1.00 0.98 1.04 0.98 2.07 0.99 1.01 1.95 3.42 1.02 0.94 1.00 22.47
f1 (ppm)

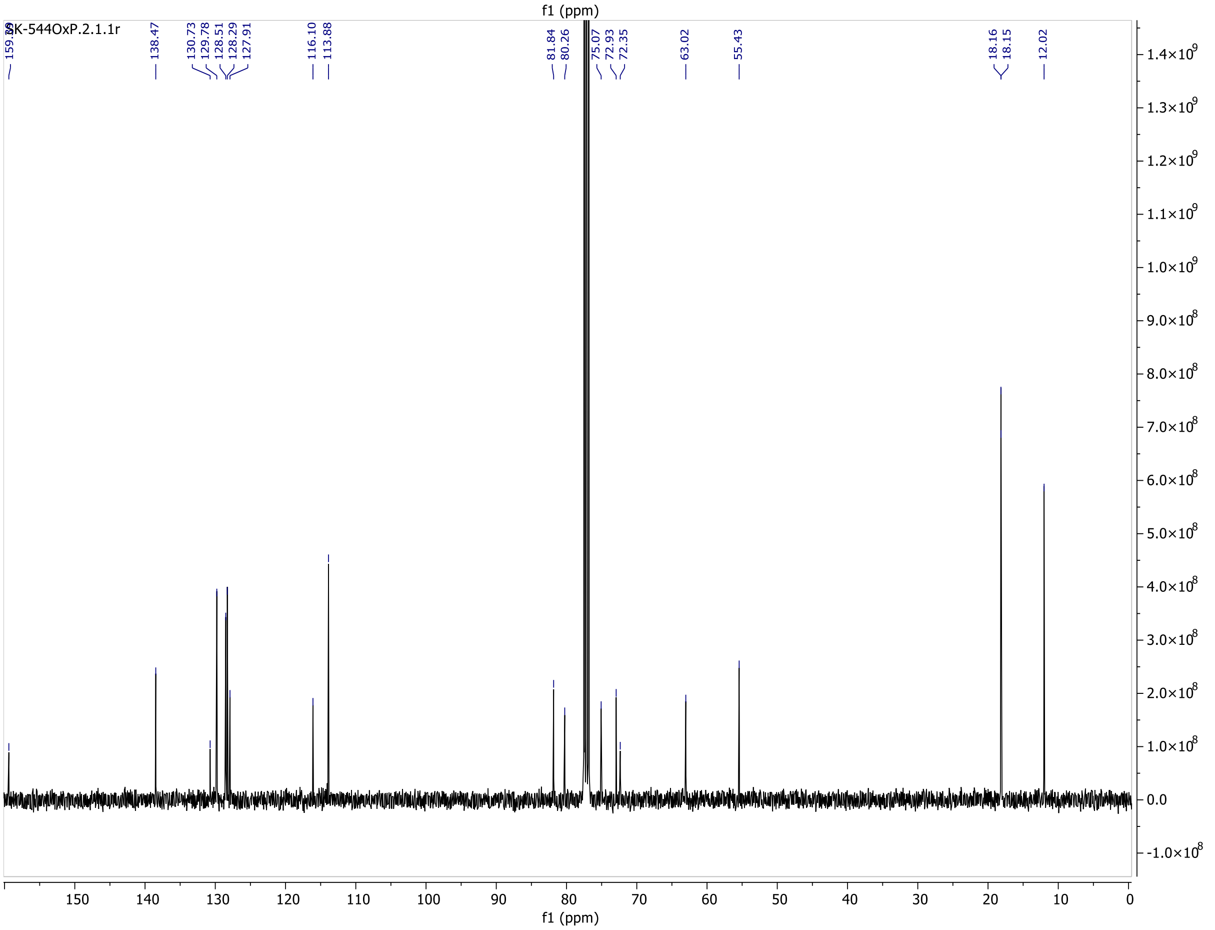
SK-544OxP.2.1.1r
159.19 138.47 130.73 129.78 128.51 128.29 127.91 116.10 113.88 81.84 80.26 75.07 72.93 72.35 63.02 55.43 18.16 18.15 12.02
f1 (ppm)

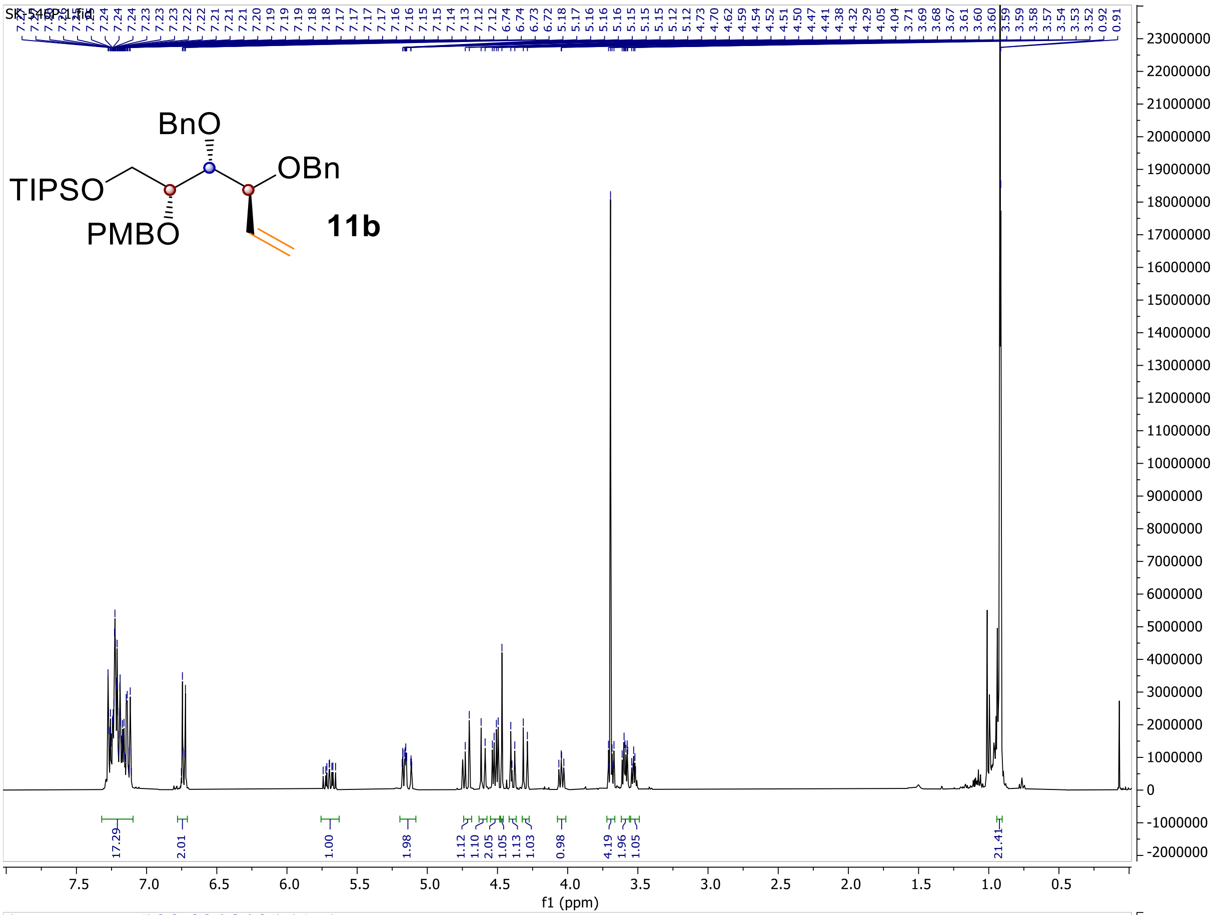

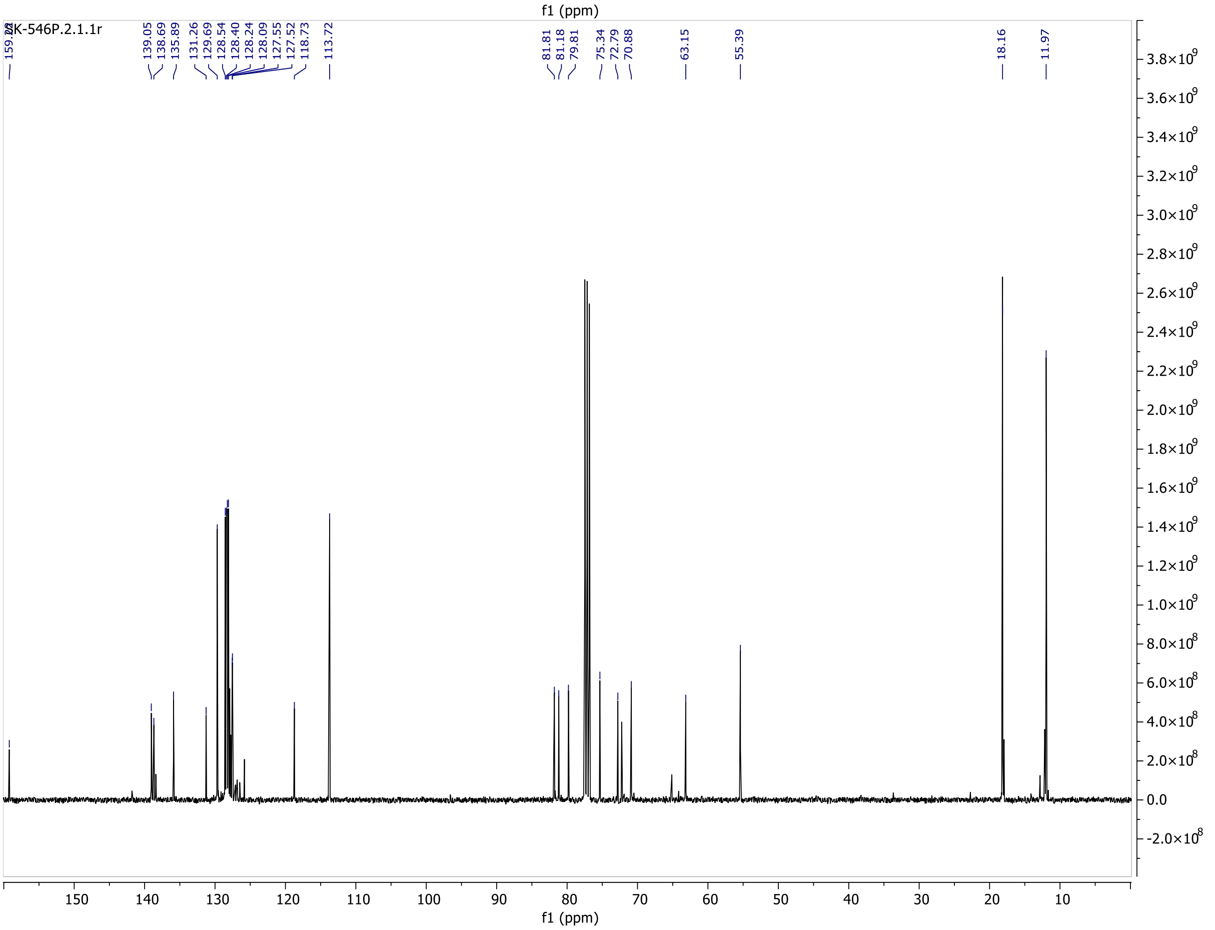

contaminated with ca. 25% BnOBn, removed after next step.

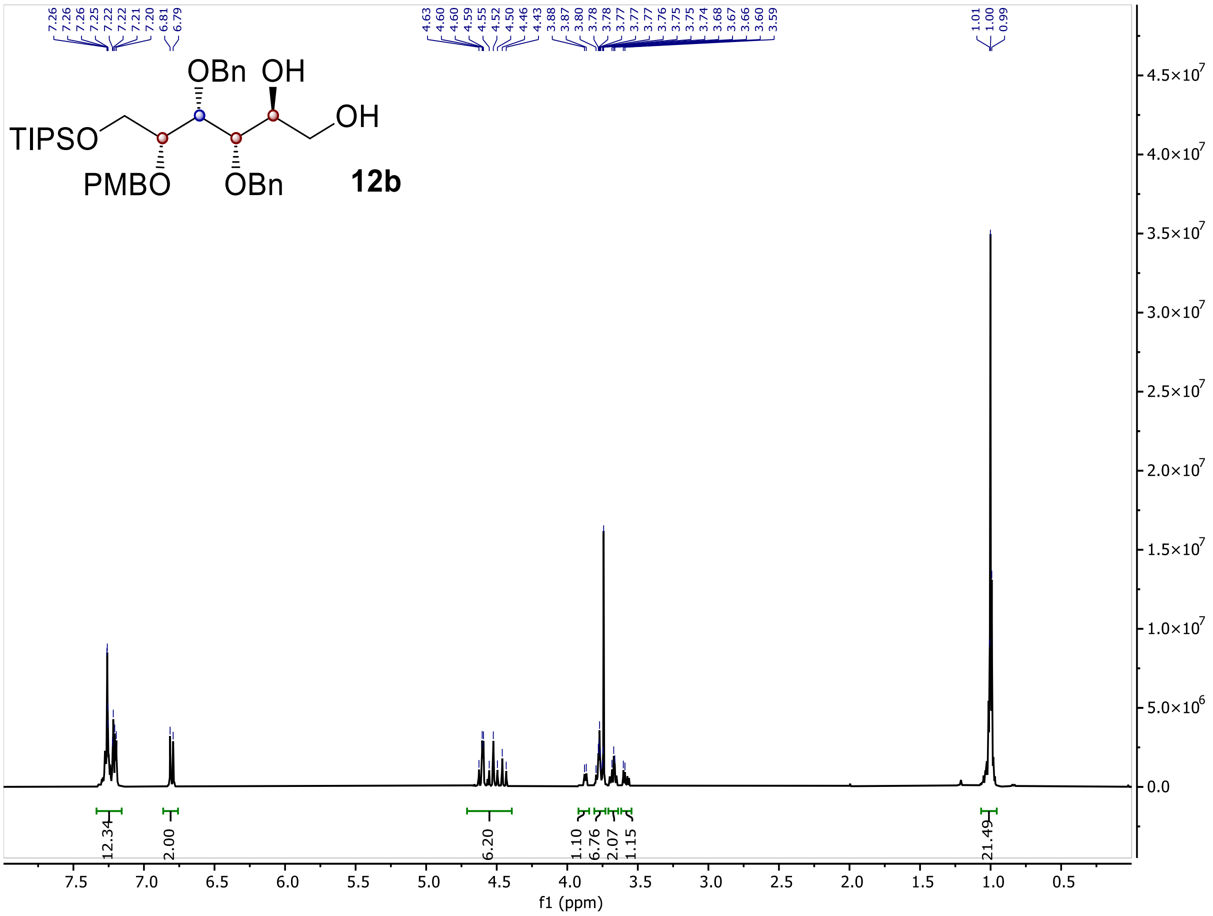
OBn OH
TIPSO
OH
PMBO
OBn
12b
7.26 7.26 7.25 7.22 7.22 7.21 7.20 6.81 6.79
4.63 4.60 4.59 4.55 4.52 4.50 4.46 4.43 3.88 3.87 3.80 3.78 3.78 3.77 3.77 3.76 3.75 3.75 3.74 3.68 3.67 3.66 3.60 3.59
1.01 1.00 0.99
12.34
2.00
6.20
1.10
6.76
2.07
1.15
21.49
f1 (ppm)

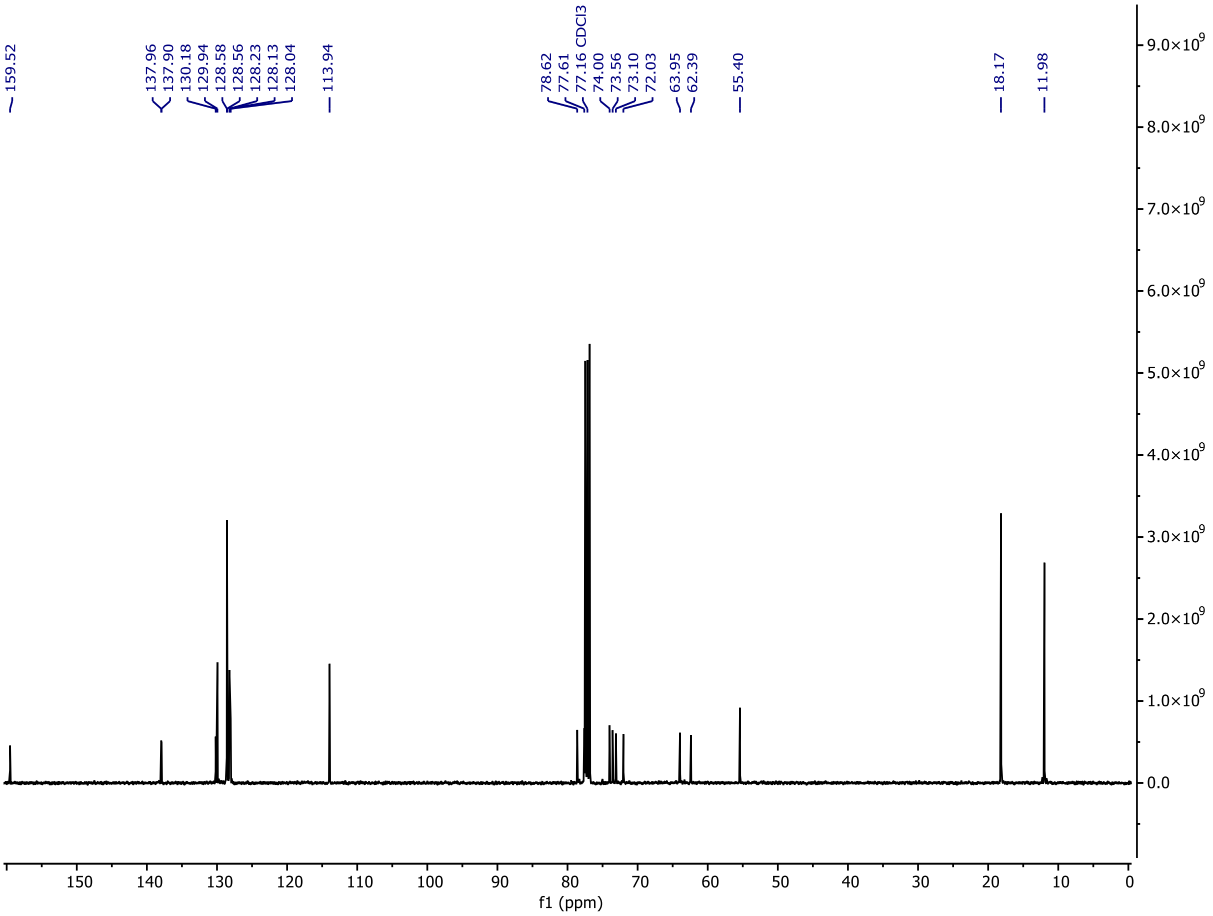
159.52
137.96 137.90 130.18 129.94 128.58 128.56 128.23 128.13 128.04
113.94
78.62 77.61 77.16 CDCl3 74.00 73.56 73.10 72.03
63.95 62.39
55.40
18.17
11.98
f1 (ppm)

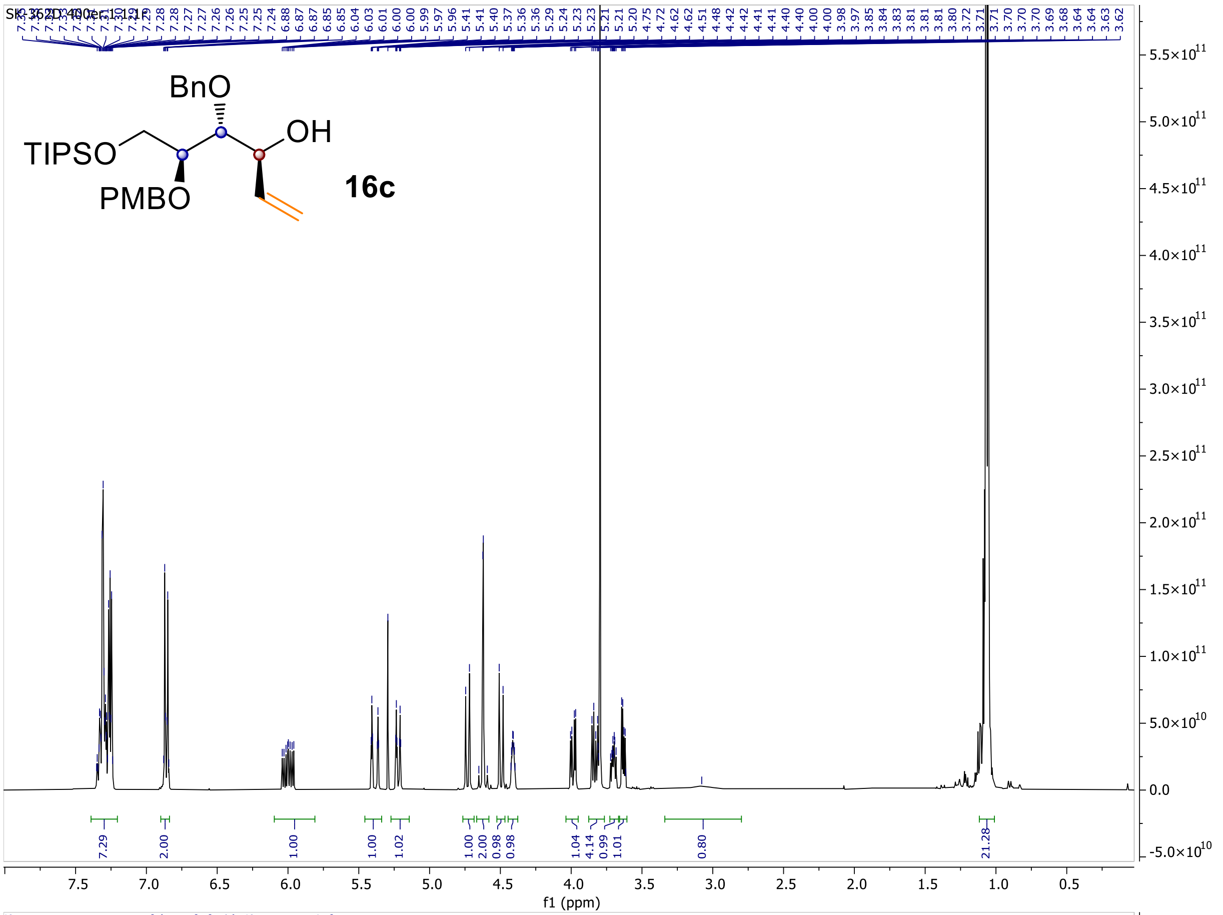
BnO
OH
TIPSO
PMBO
16c
7.29
2.00
1.00
1.00
1.02
1.00
2.00
0.98
0.98
1.04
4.14
0.99
1.01
0.80
21.28
f1 (ppm)

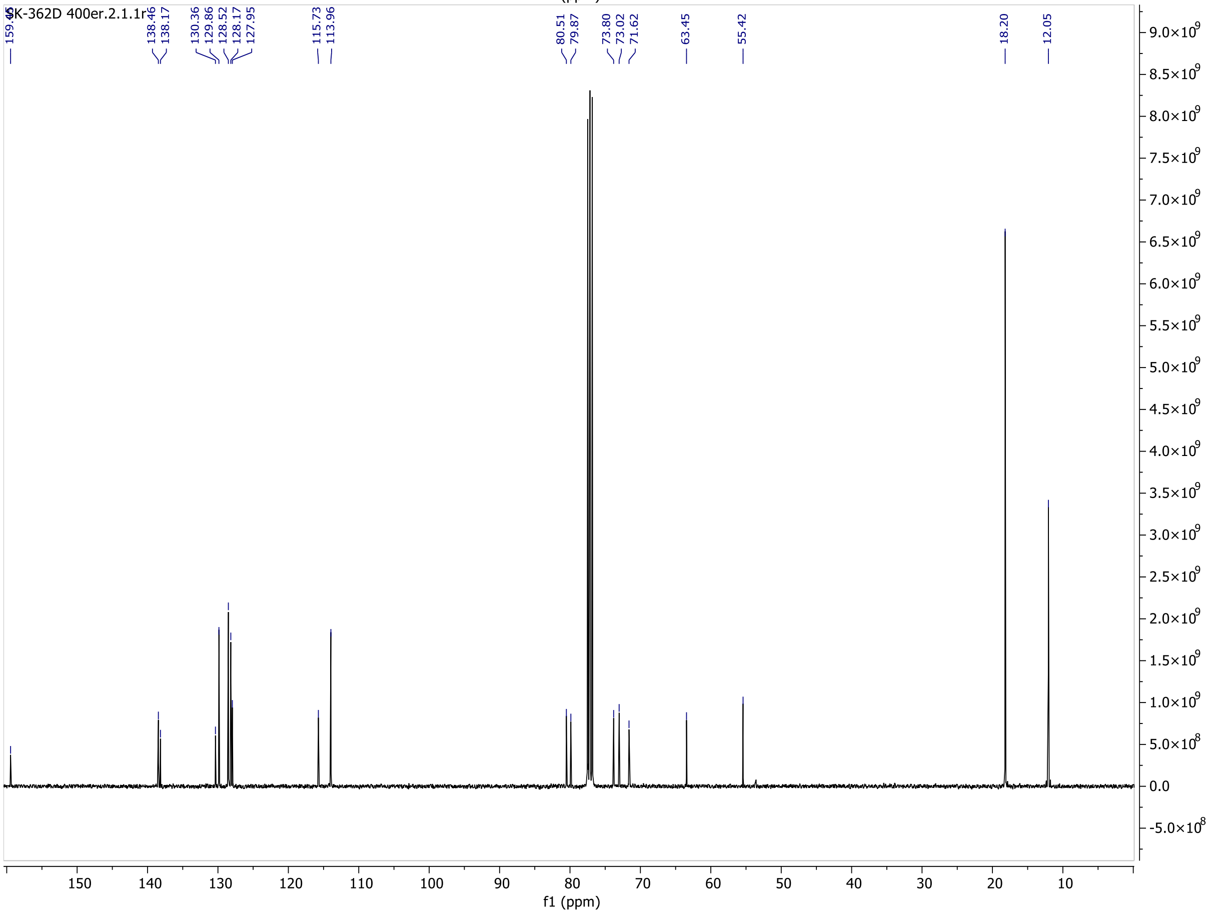
SK-362D 400er.2.1.1r
138.46
138.17
130.36
129.86
128.52
128.17
127.95
115.73
113.96
80.51
79.87
73.80
73.02
71.62
63.45
55.42
18.20
12.05
f1 (ppm)

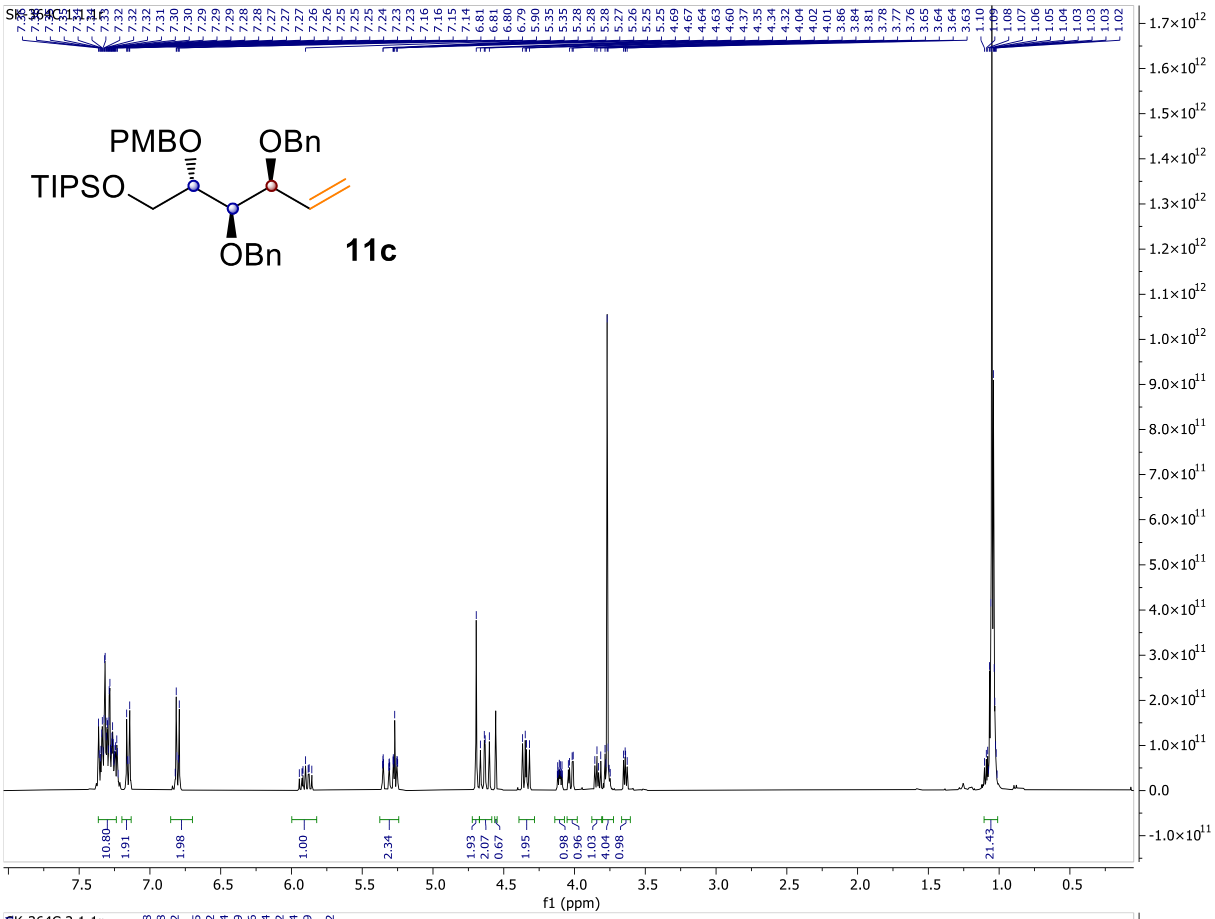

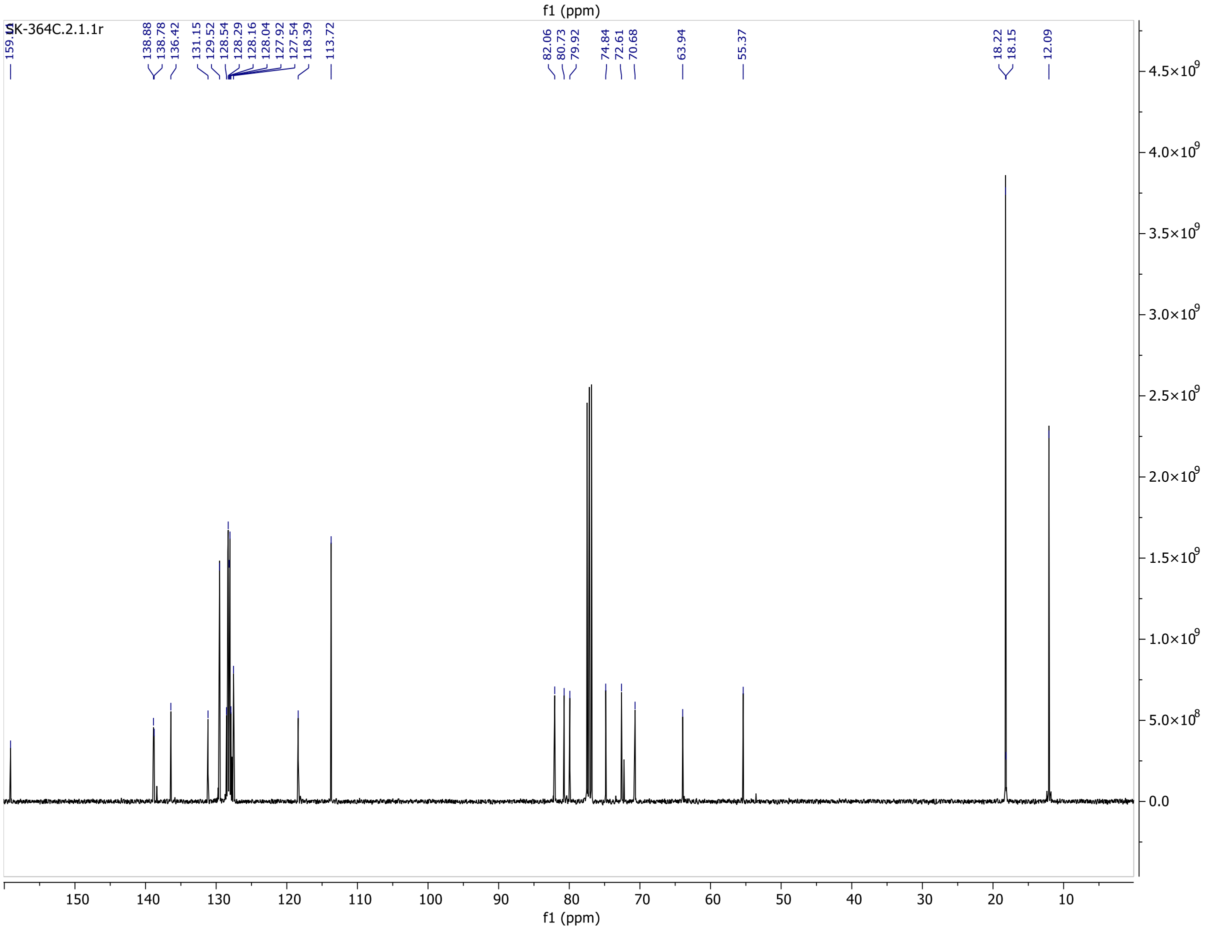

contaminated with ca. 17% BnOBn, removed after next step.

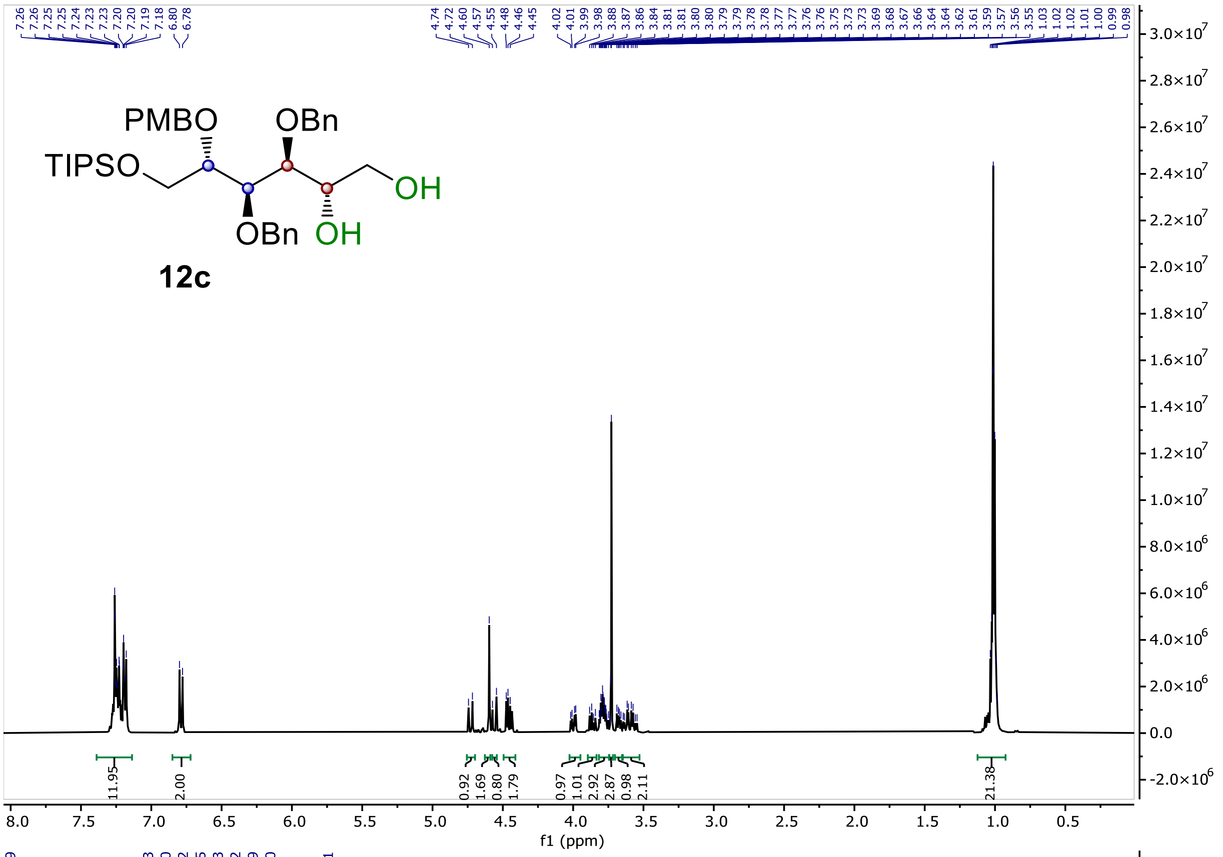
PMBO
OBn
TIPSO
OH
OBn
OH
12c
f1 (ppm)

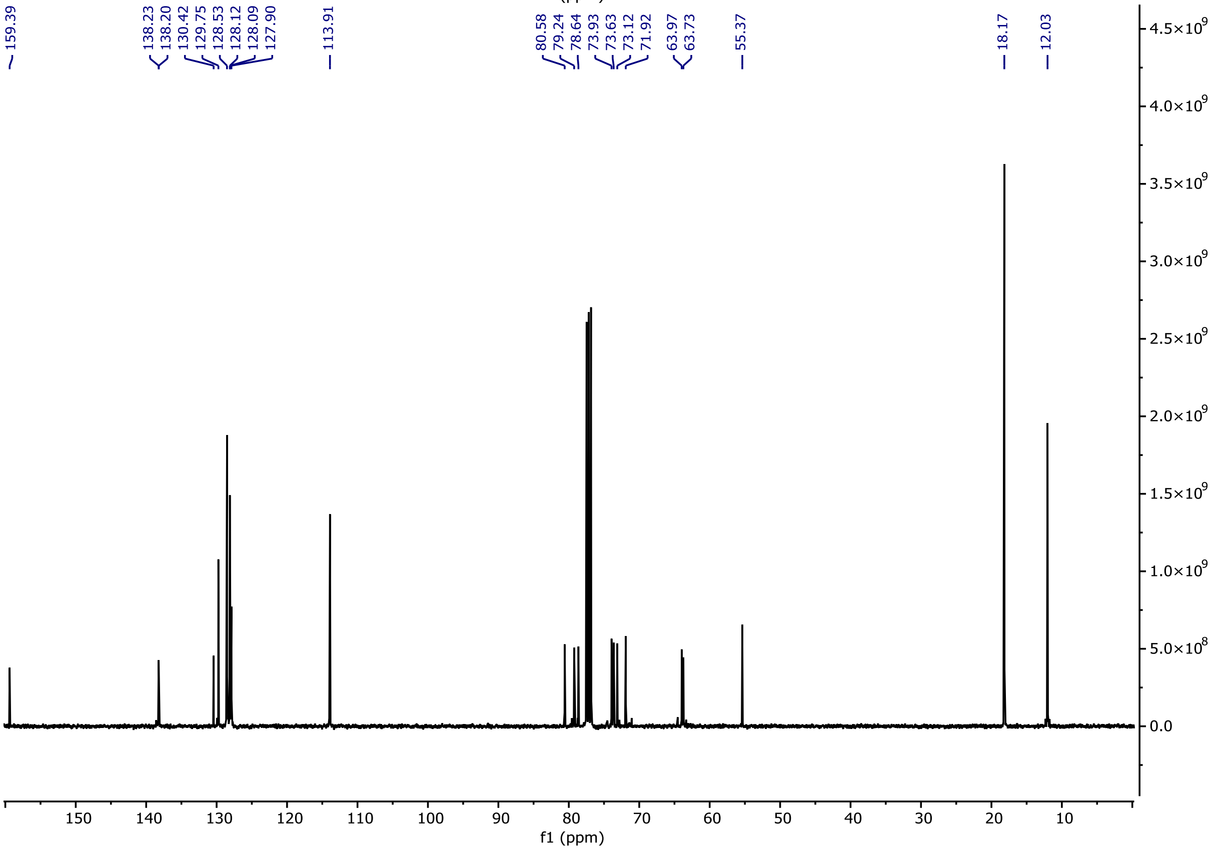
159.39
138.23
138.20
130.42
129.75
128.53
128.12
128.09
127.90
113.91
80.58
79.24
78.64
73.93
73.63
73.12
71.92
63.97
63.73
55.37
18.17
12.03
f1 (ppm)

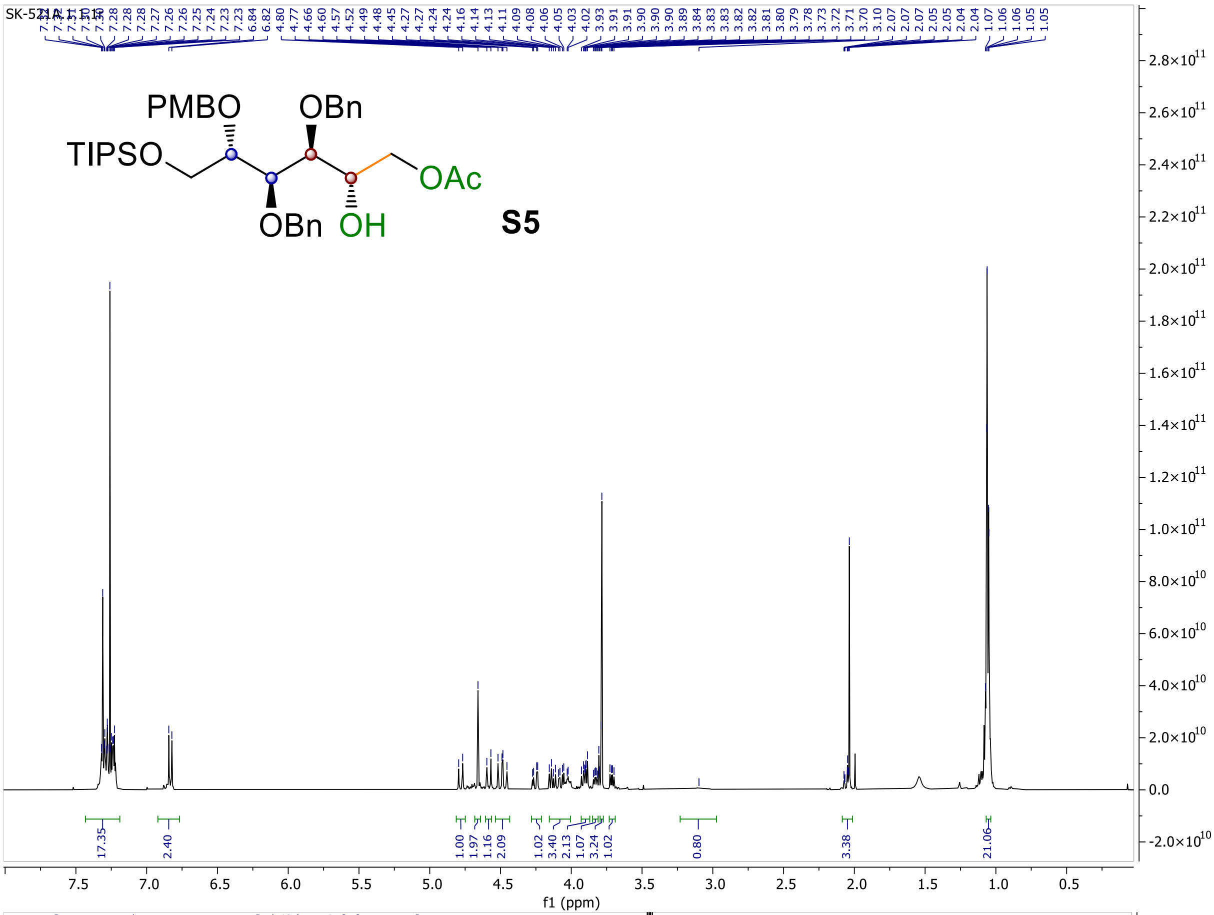
SK-521A.1.1r
PMBO
OBn
TIPSO
OAc
OBn
OH
S5
f1 (ppm)

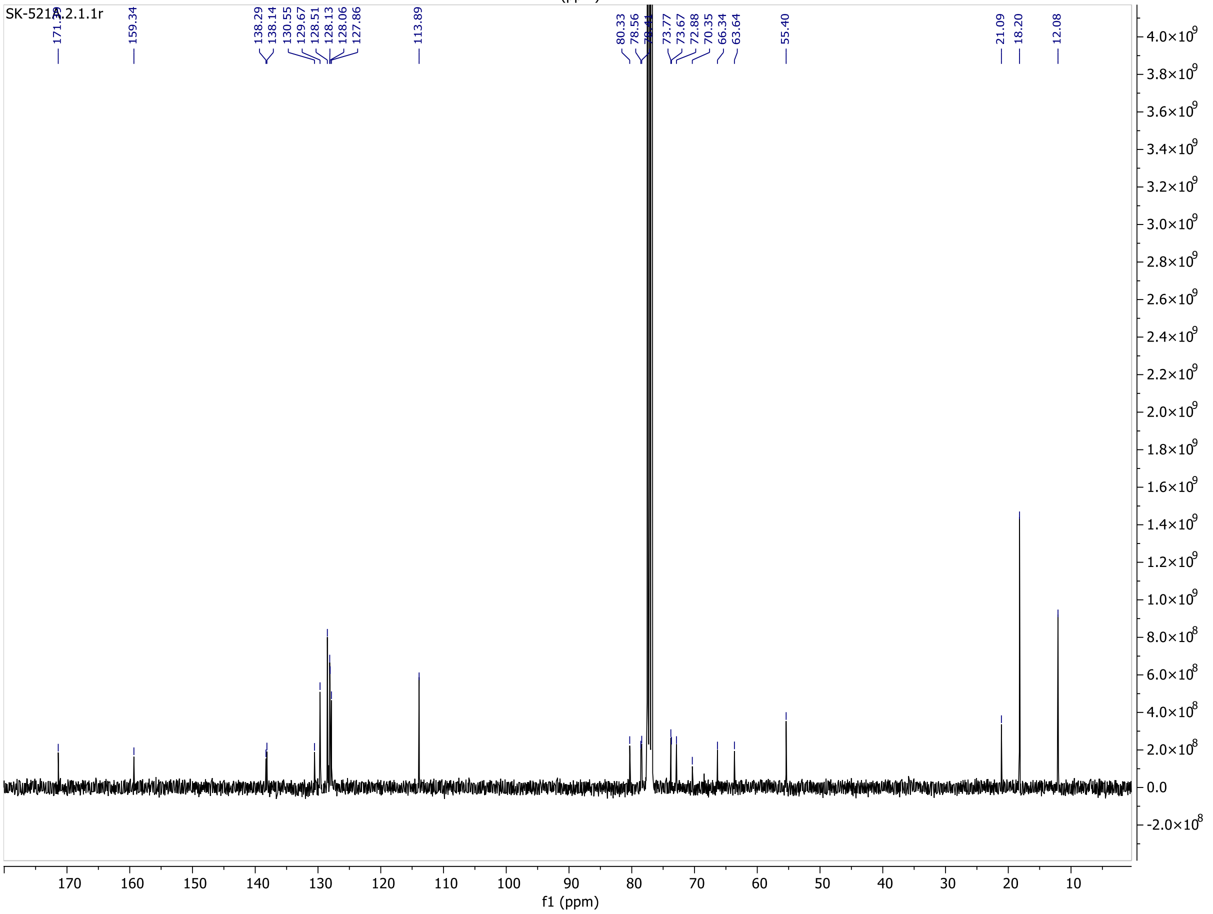
SK-521A.2.1.1r
159.34
113.89
55.40
21.09
18.20
12.08
f1 (ppm)

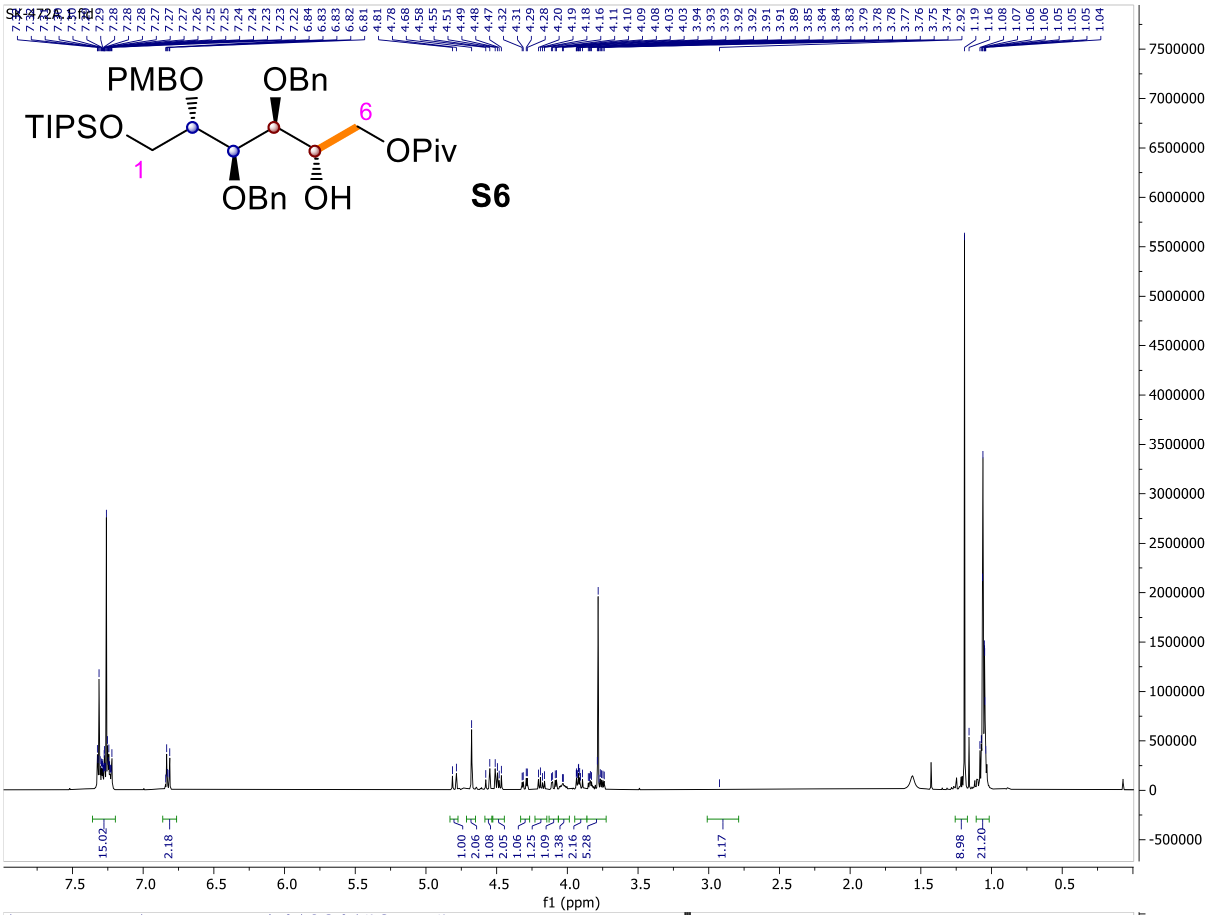
PMBO
OBn
TIPSO
6
OPiv
1
OBn
OH
S6
15.02
2.18
1.00
2.06
1.08
2.05
1.06
1.25
1.09
1.38
2.16
5.28
1.17
8.98
21.20
f1 (ppm)

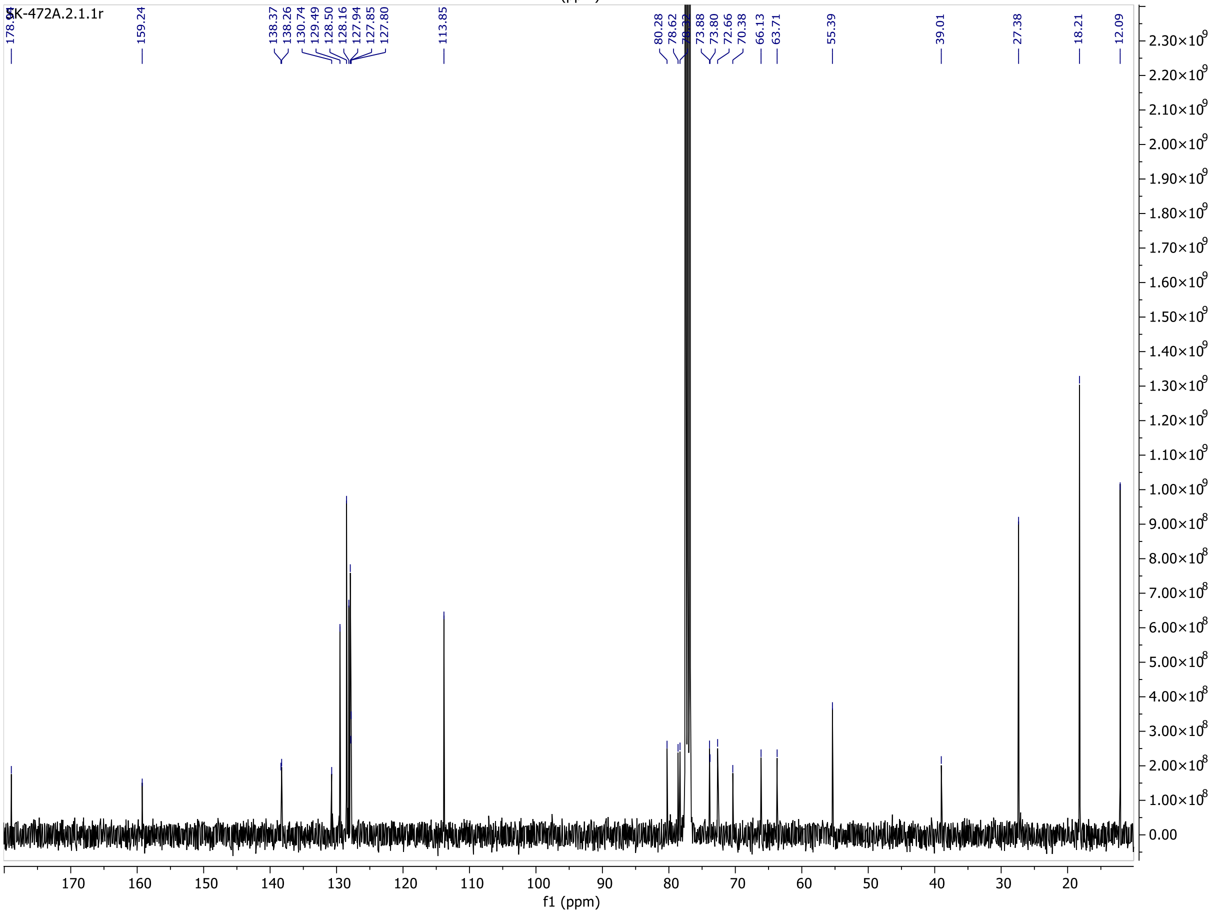
SK-472A.2.1.1r
159.24
138.37
138.26
130.74
129.49
128.50
128.16
127.94
127.85
127.80
113.85
80.28
78.62
73.88
73.80
72.66
70.38
66.13
63.71
55.39
39.01
27.38
18.21
12.09
f1 (ppm)

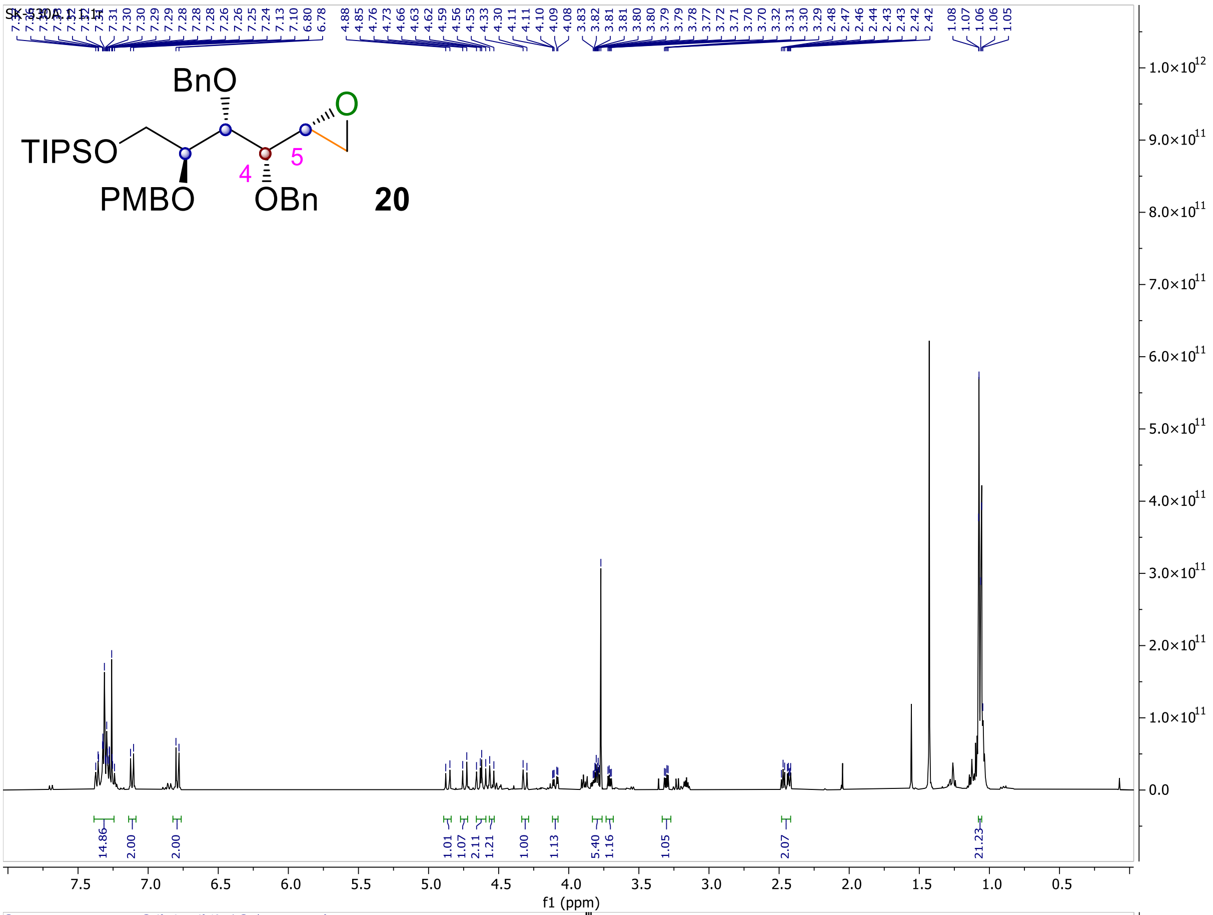
SK-530A.1.1.1r
7.31 7.30 7.30 7.29 7.29 7.28 7.28 7.28 7.26 7.26 7.25 7.24 7.13 7.10 6.80 6.78
4.88 4.85 4.76 4.73 4.66 4.63 4.62 4.59 4.56 4.53 4.33 4.30 4.11 4.11 4.10 4.09 4.08 3.83 3.82 3.81 3.81 3.80 3.79 3.78 3.77 3.72 3.71 3.70 3.32 3.31 3.30 3.29 2.48 2.47 2.46 2.44 2.43 2.42 2.42
1.08 1.07 1.06 1.06 1.05
BnO
TIPSO
PMBO
OBn
O
4
5
20
14.86 2.00 2.00 1.01 1.07 2.11 1.21 1.00 1.13 5.40 1.16 1.05 2.07 21.23
f1 (ppm)

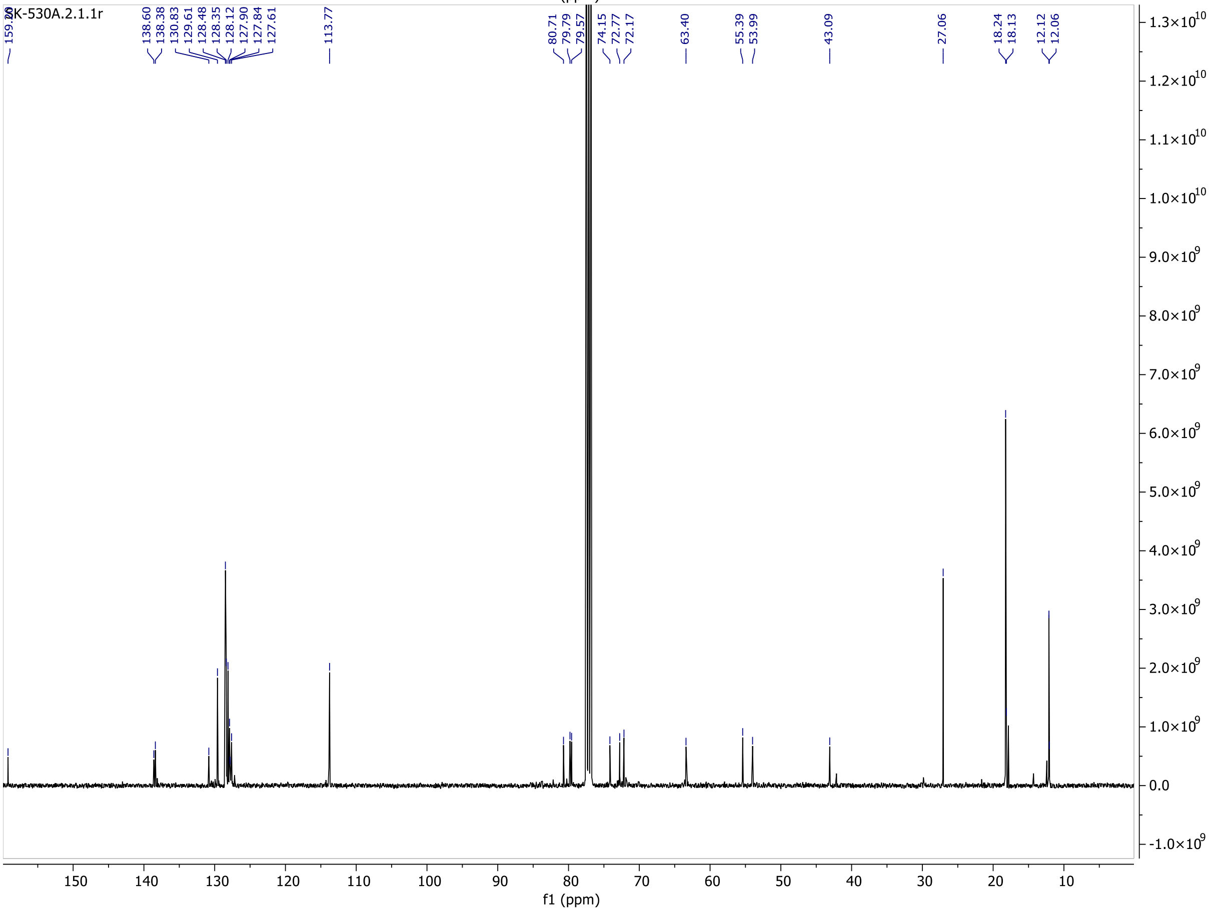
SK-530A.2.1.1r
138.60 138.38 130.83 129.61 128.48 128.35 128.12 127.90 127.84 127.61 113.77
80.71 79.79 79.57 74.15 72.77 72.17 63.40 55.39 53.99 43.09 27.06 18.24 18.13 12.12 12.06
f1 (ppm)

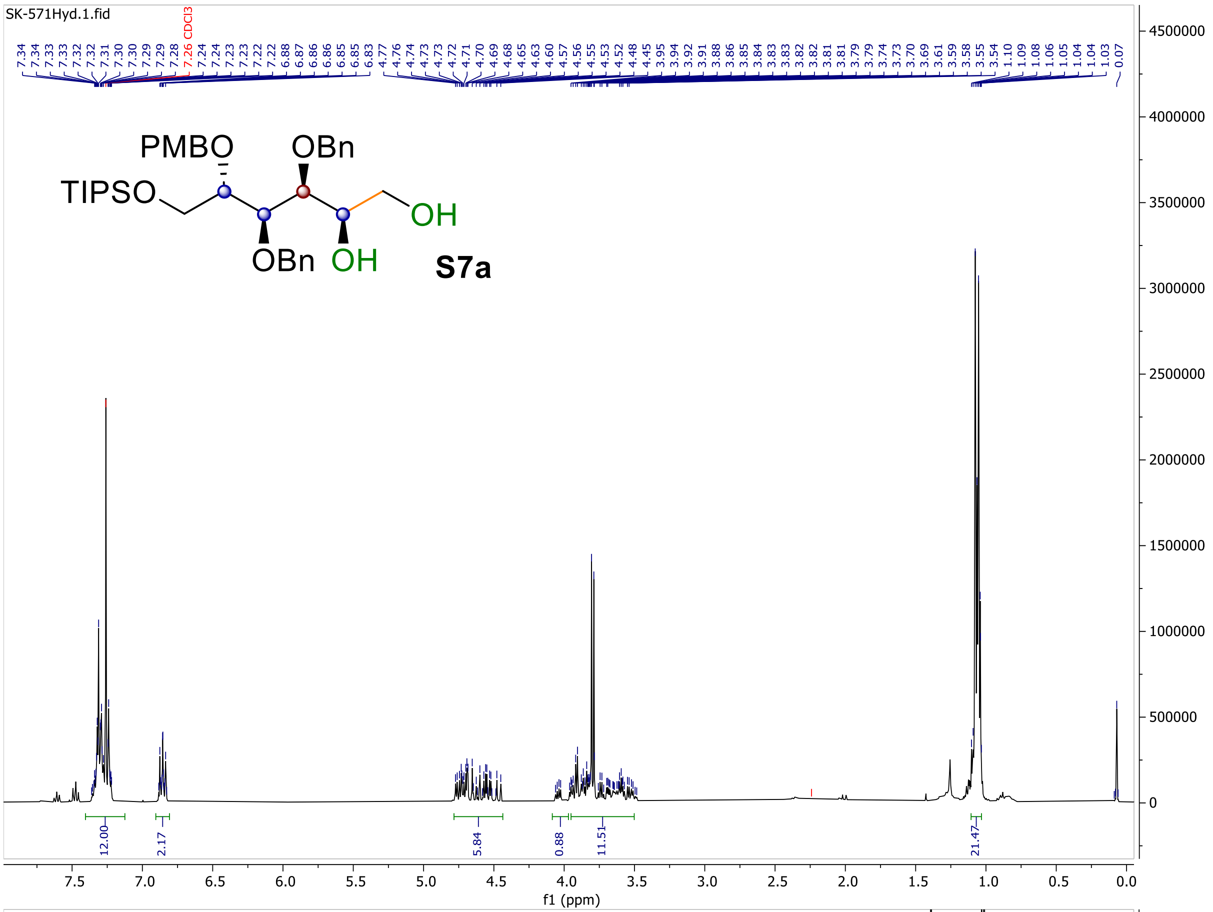
SK-571Hyd.1.fid
PMBO
OBn
TIPSO
OH
OBn
OH
S7a
12.00
2.17
5.84
0.88
11.51
21.47
f1 (ppm)

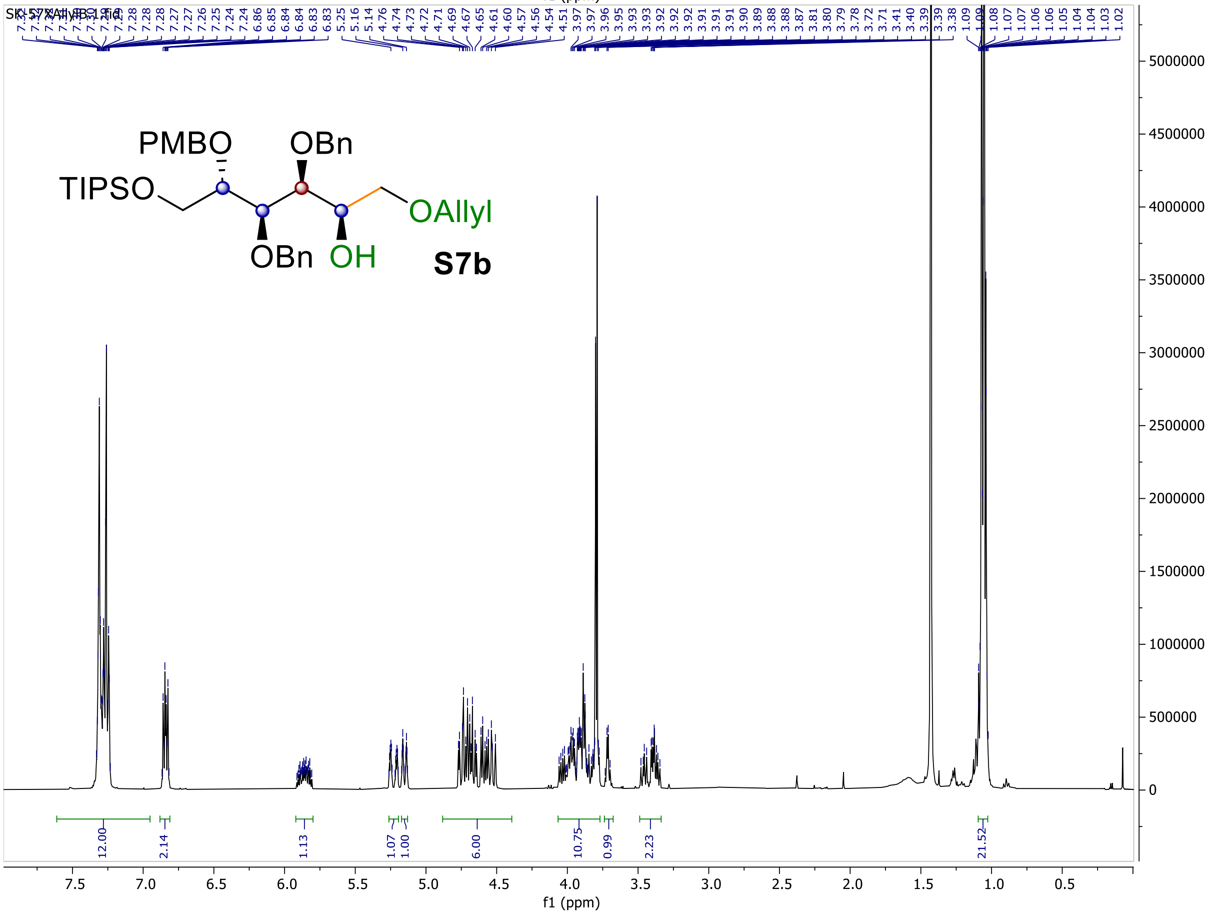
PMBO
OBn
TIPSO
OAllyl
OBn
OH
S7b
12.00
2.14
1.13
1.07
1.00
6.00
10.75
0.99
2.23
21.52
f1 (ppm)

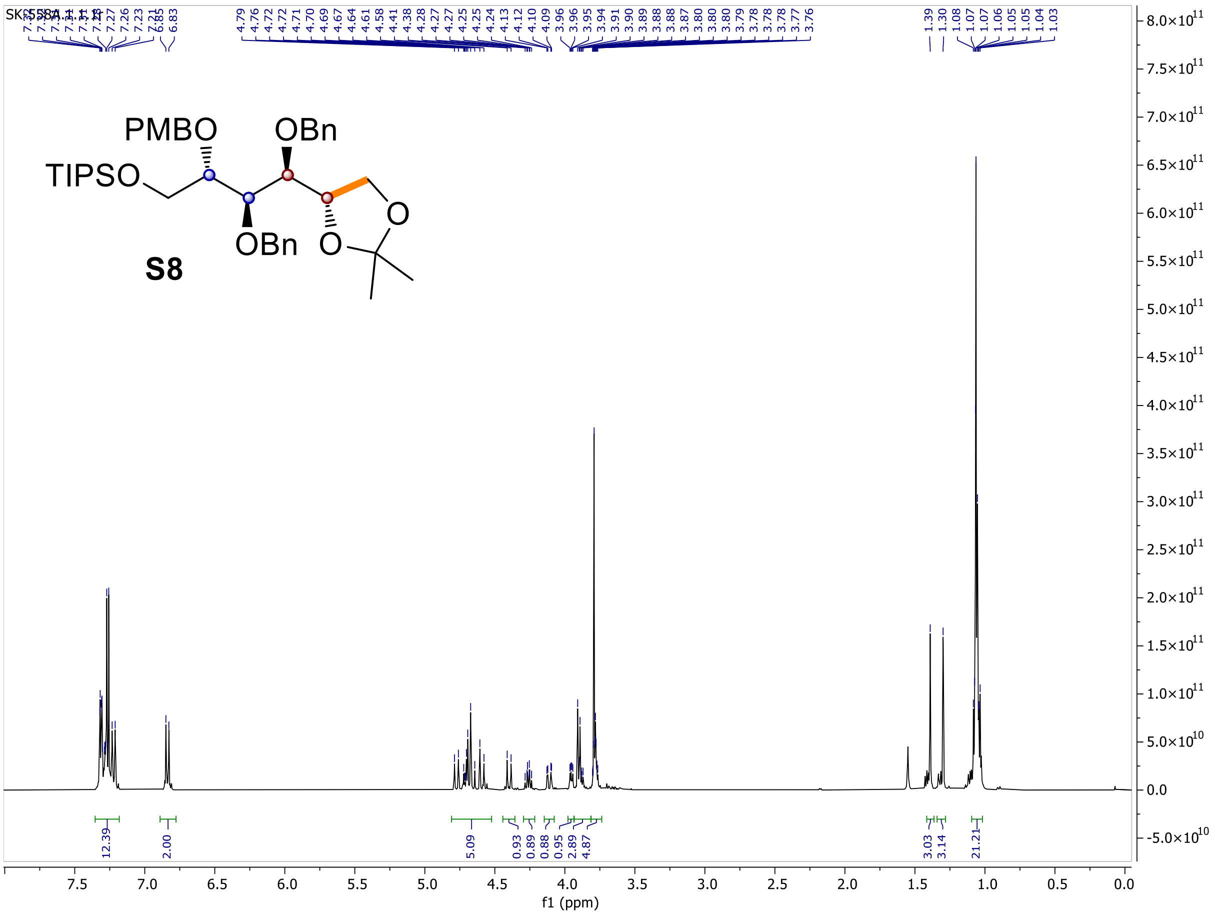
SK-558A.1.1.1r
PMBO
OBn
TIPSO
OBn
O
O
S8
6.85
6.83
4.79
4.76
4.72
4.72
4.71
4.70
4.69
4.67
4.64
4.61
4.58
4.41
4.38
4.28
4.27
4.27
4.25
4.25
4.24
4.13
4.12
4.10
4.09
3.96
3.96
3.95
3.94
3.91
3.90
3.89
3.88
3.88
3.87
3.80
3.80
3.80
3.79
3.78
3.78
3.78
3.77
3.76
1.39
1.30
1.08
1.07
1.07
1.06
1.05
1.05
1.04
1.03
12.39
2.00
5.09
0.93
0.89
0.88
0.95
2.89
4.87
3.03
3.14
21.21
f1 (ppm)

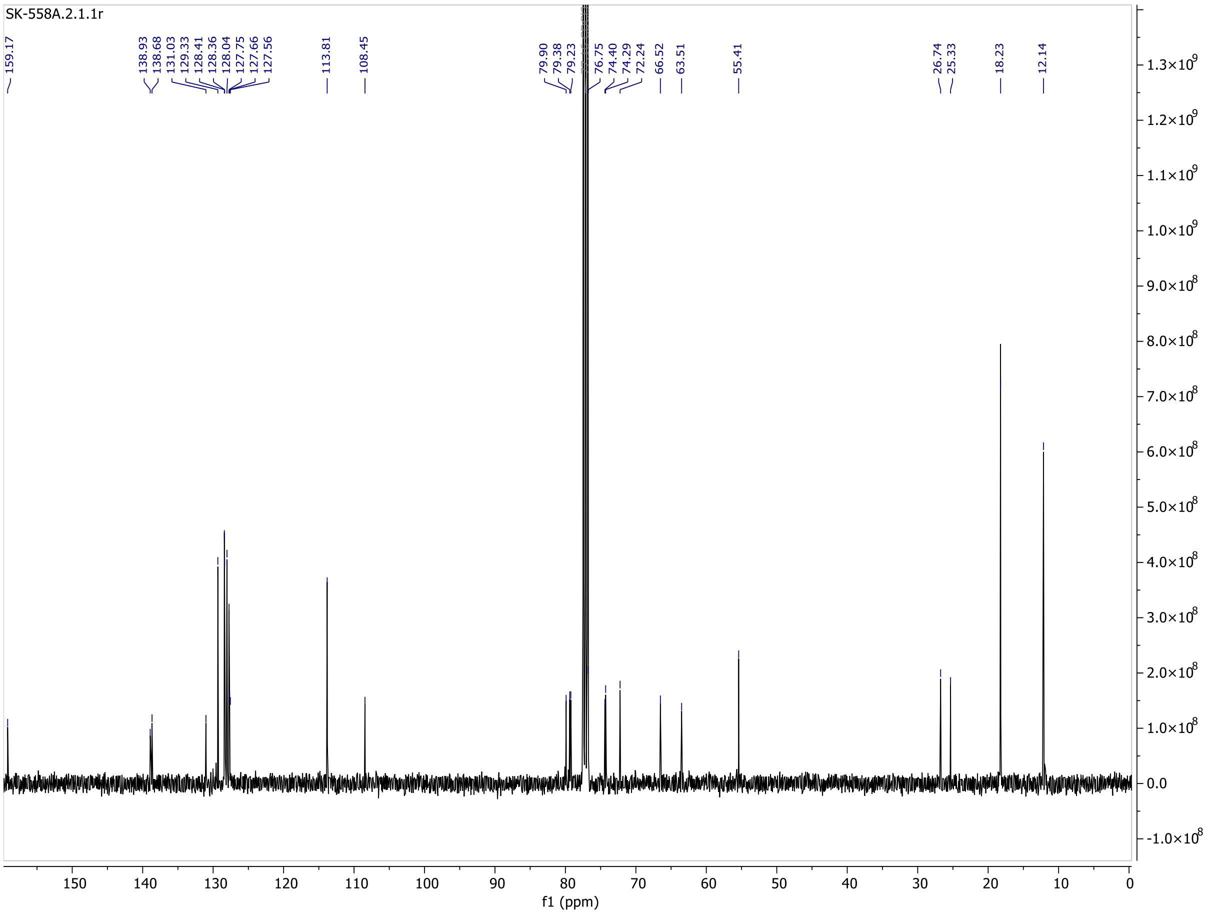
SK-558A.2.1.1r
159.17
138.93
138.68
131.03
129.33
128.41
128.36
128.04
127.75
127.66
127.56
113.81
108.45
79.90
79.38
79.23
76.75
74.40
74.29
72.24
66.52
63.51
55.41
26.74
25.33
18.23
12.14
f1 (ppm)

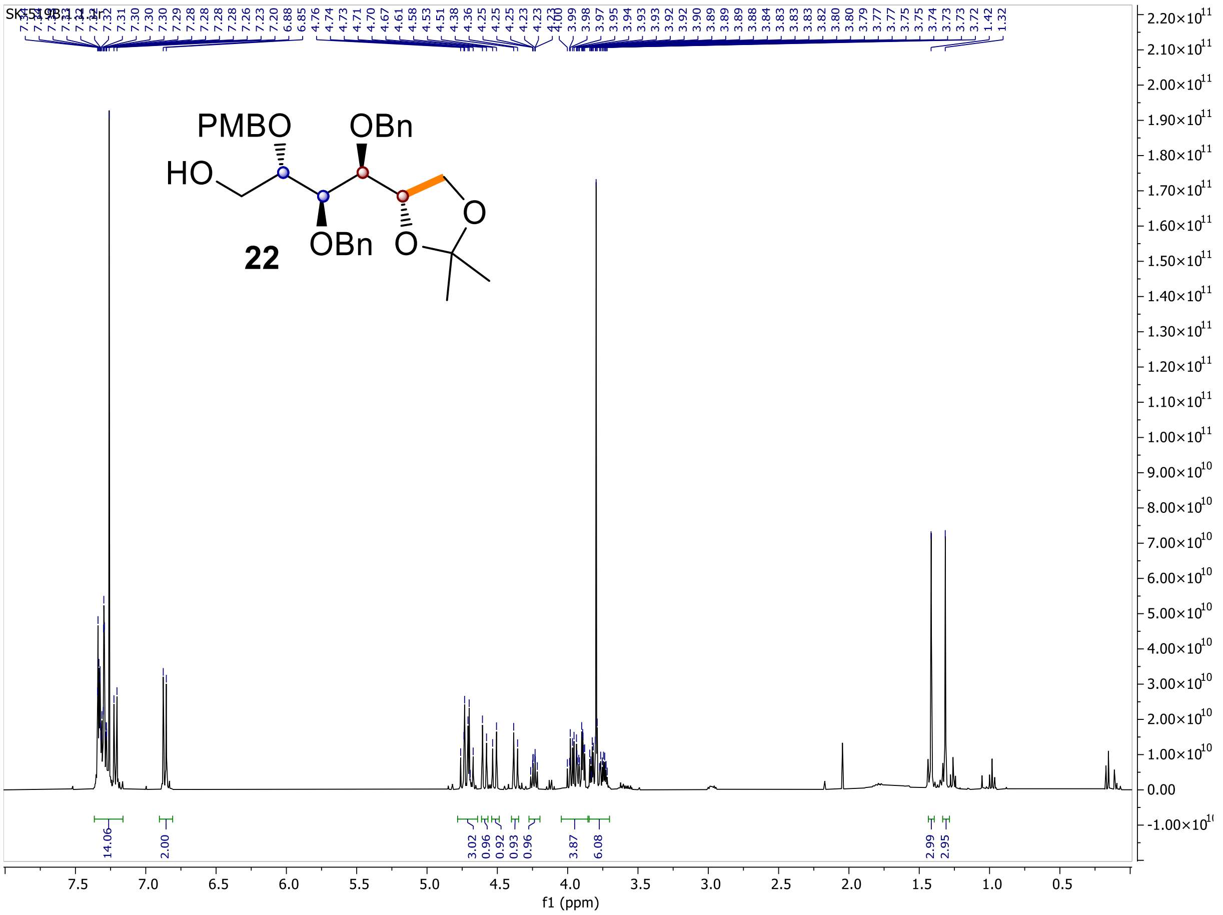
PMBO
OBn
HO
OBn
O
O
22
f1 (ppm)

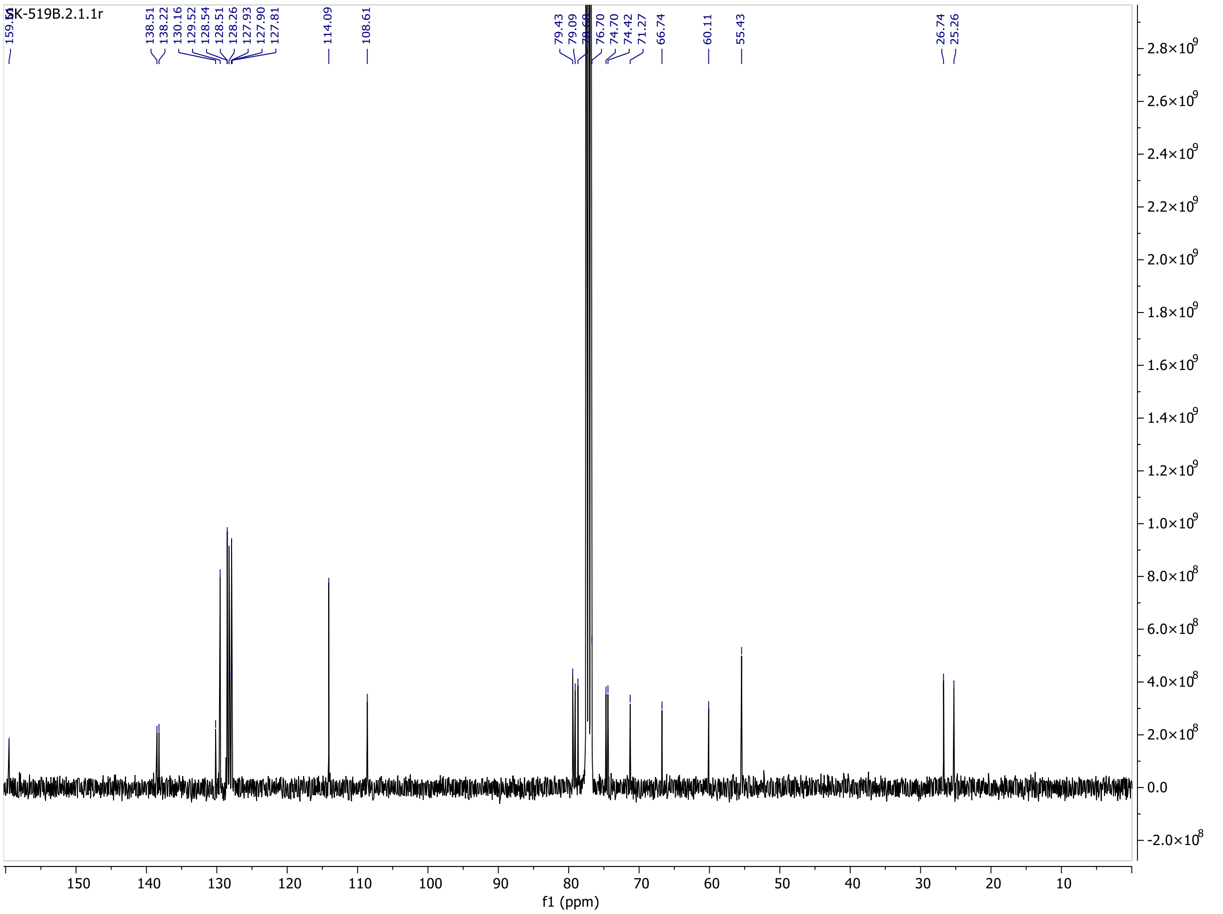
SK-519B.2.1.1r
f1 (ppm)

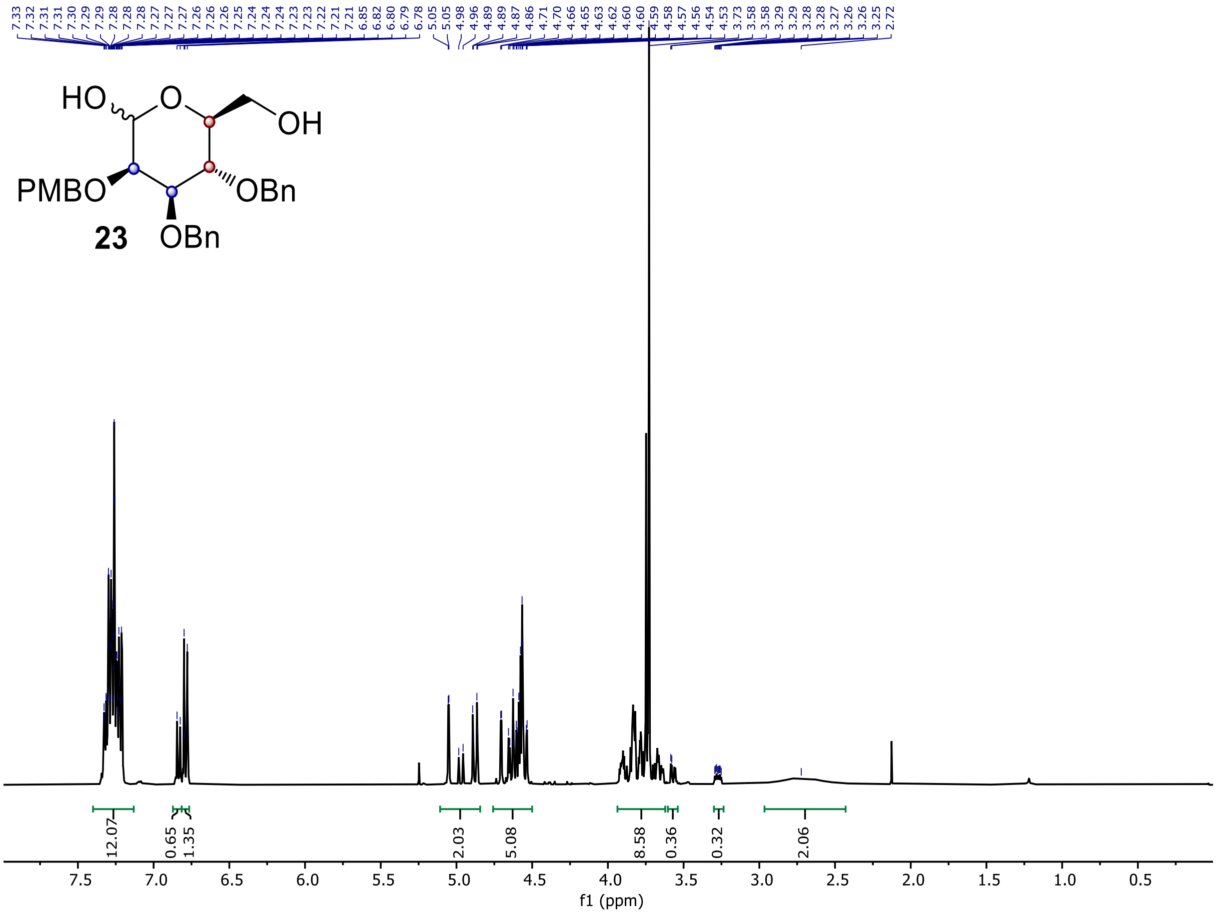
7.33 7.32 7.31 7.30 7.29 7.29 7.28 7.28 7.28 7.27 7.27 7.26 7.26 7.25 7.24 7.24 7.24 7.23 7.23 7.22 7.21 7.21 6.85 6.82 6.80 6.79 6.78 5.05 5.05 4.98 4.96 4.89 4.89 4.87 4.86 4.71 4.70 4.66 4.65 4.63 4.62 4.60 4.60 4.59 4.58 4.57 4.56 4.54 4.53 3.73 3.58 3.58 3.29 3.29 3.28 3.28 3.27 3.26 3.26 3.25 2.72
HO
O
OH
PMBO
OBn
23
OBn
12.07
0.65
1.35
2.03
5.08
8.58
0.36
0.32
2.06
7.5 7.0 6.5 6.0 5.5 5.0 4.5 4.0 3.5 3.0 2.5 2.0 1.5 1.0 0.5
f1 (ppm)

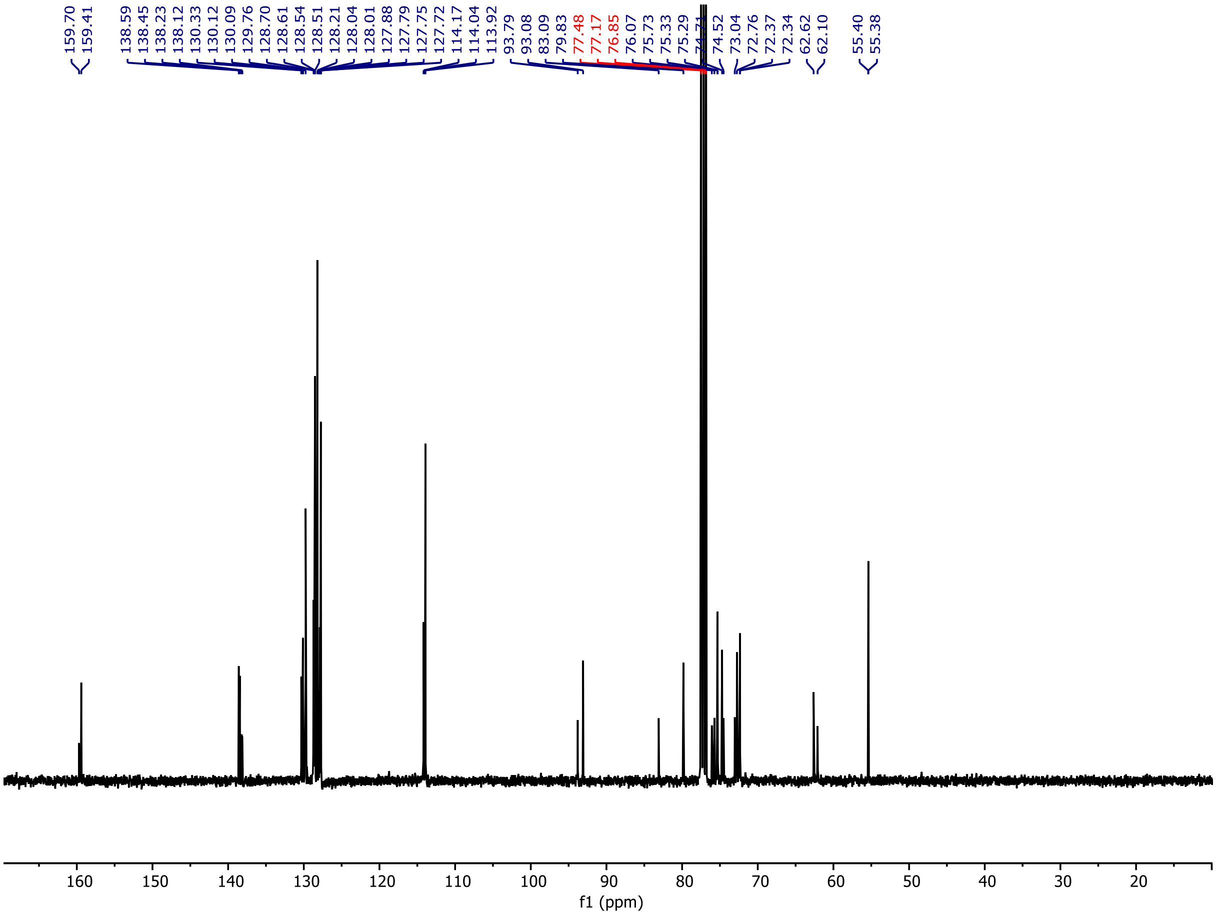
159.70 159.41 138.59 138.45 138.23 138.12 130.33 130.12 130.09 129.76 128.70 128.61 128.54 128.51 128.21 128.04 128.01 127.88 127.79 127.75 127.72 114.17 114.04 113.92 93.79 93.08 83.09 79.83 77.48 77.17 76.85 76.07 75.73 75.33 75.29 [illegible] 74.52 73.04 72.76 72.37 72.34 62.62 62.10 55.40 55.38
160 150 140 130 120 110 100 90 80 70 60 50 40 30 20
f1 (ppm)

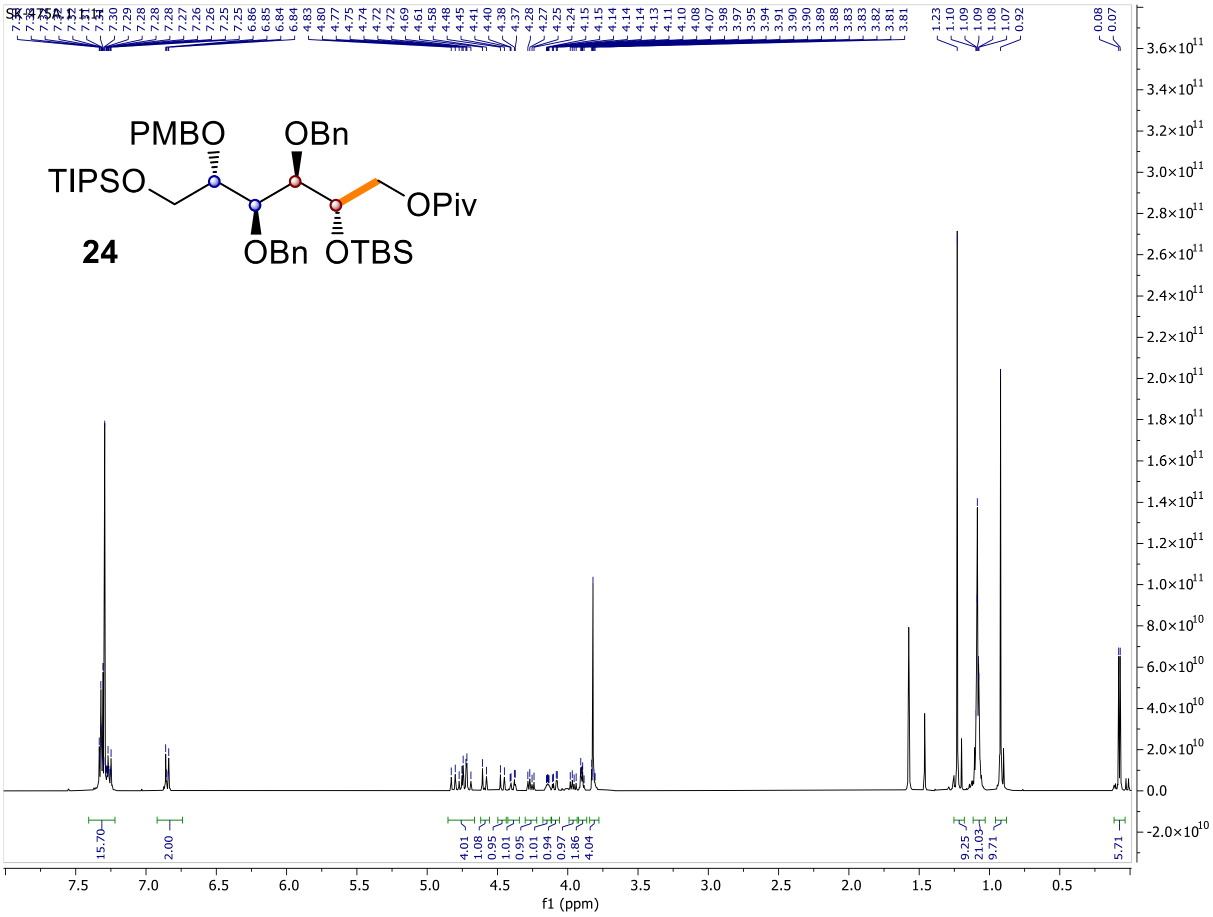
PMBO
OBn
TIPSO
OPiv
24
OBn
OTBS
f1 (ppm)

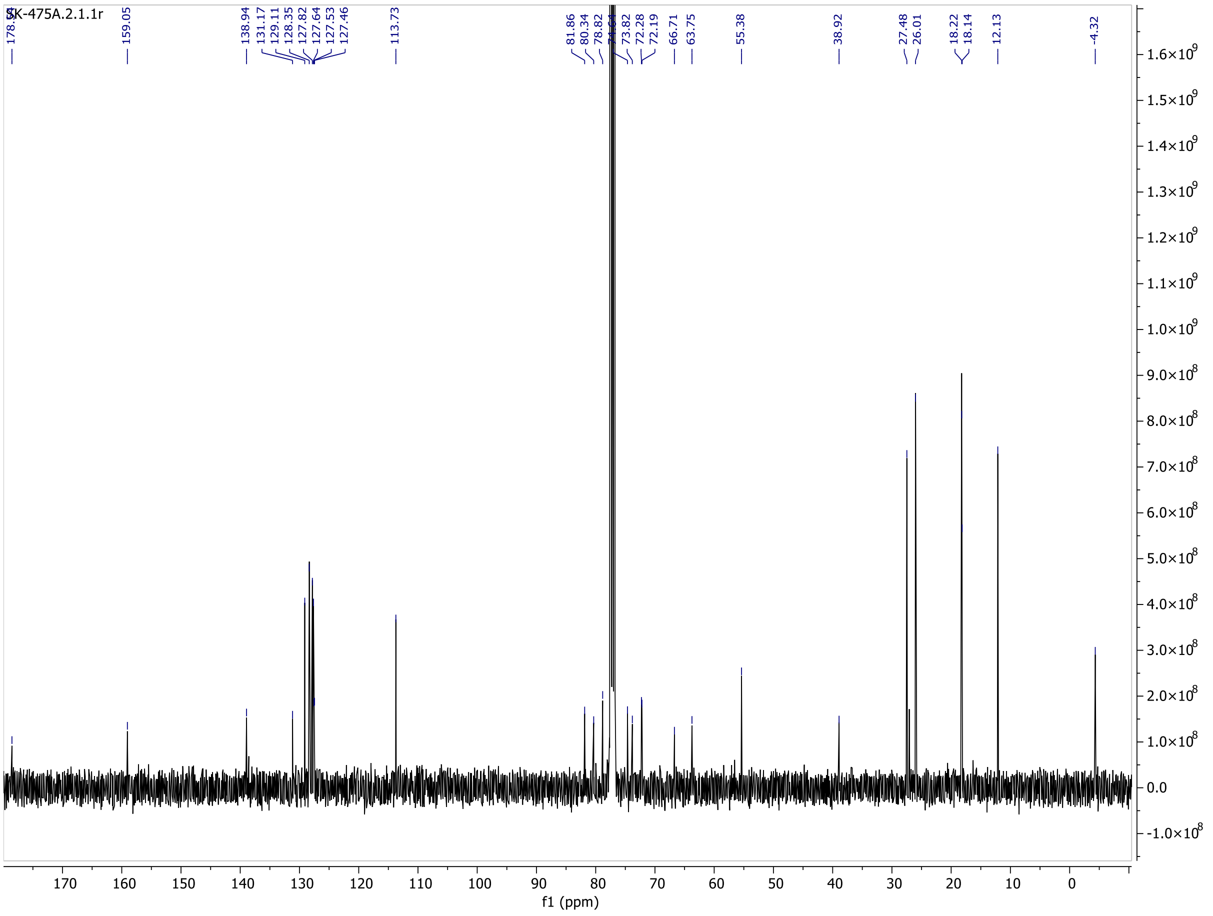
SK-475A.2.1.1r
f1 (ppm)

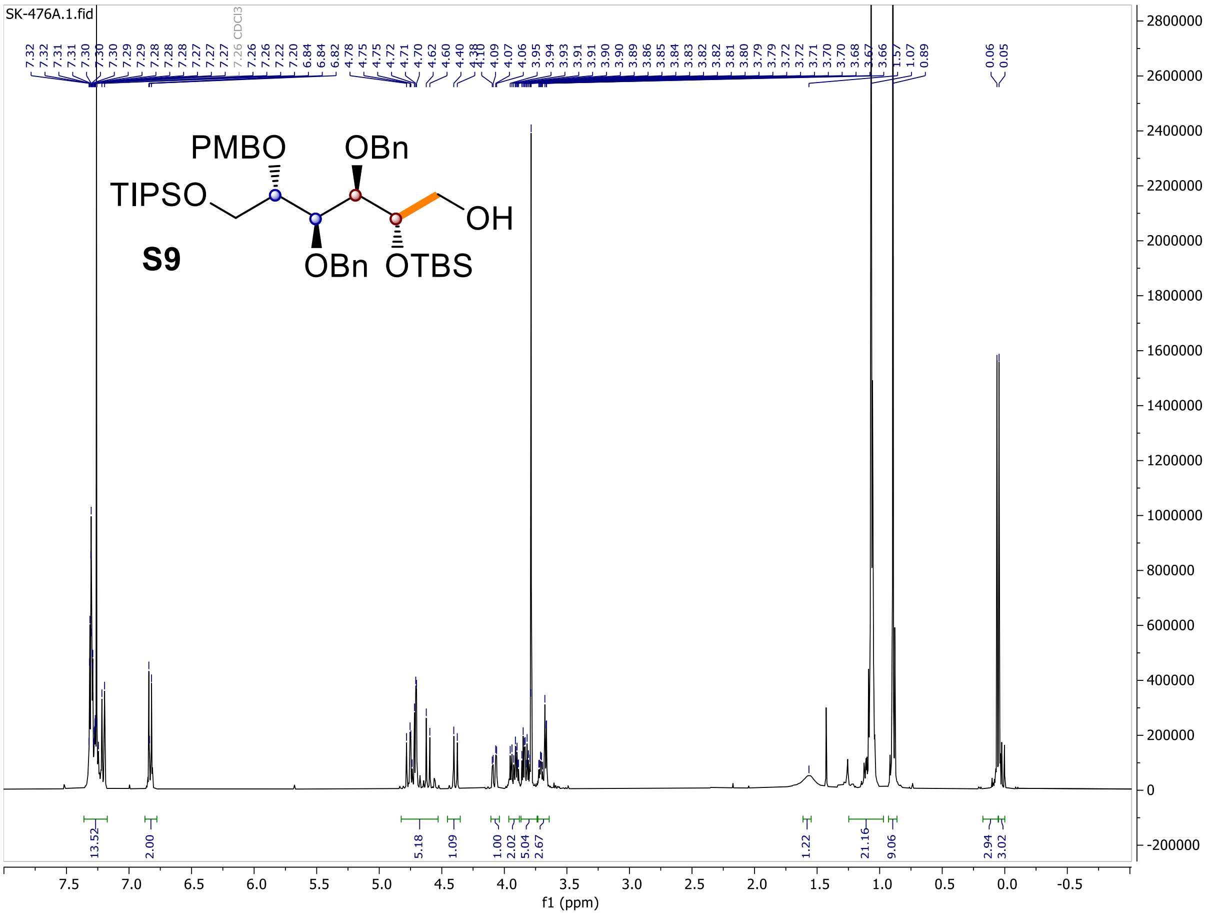
SK-476A.1.fid
PMBO
OBn
TIPSO
OH
S9
OBn
OTBS
f1 (ppm)

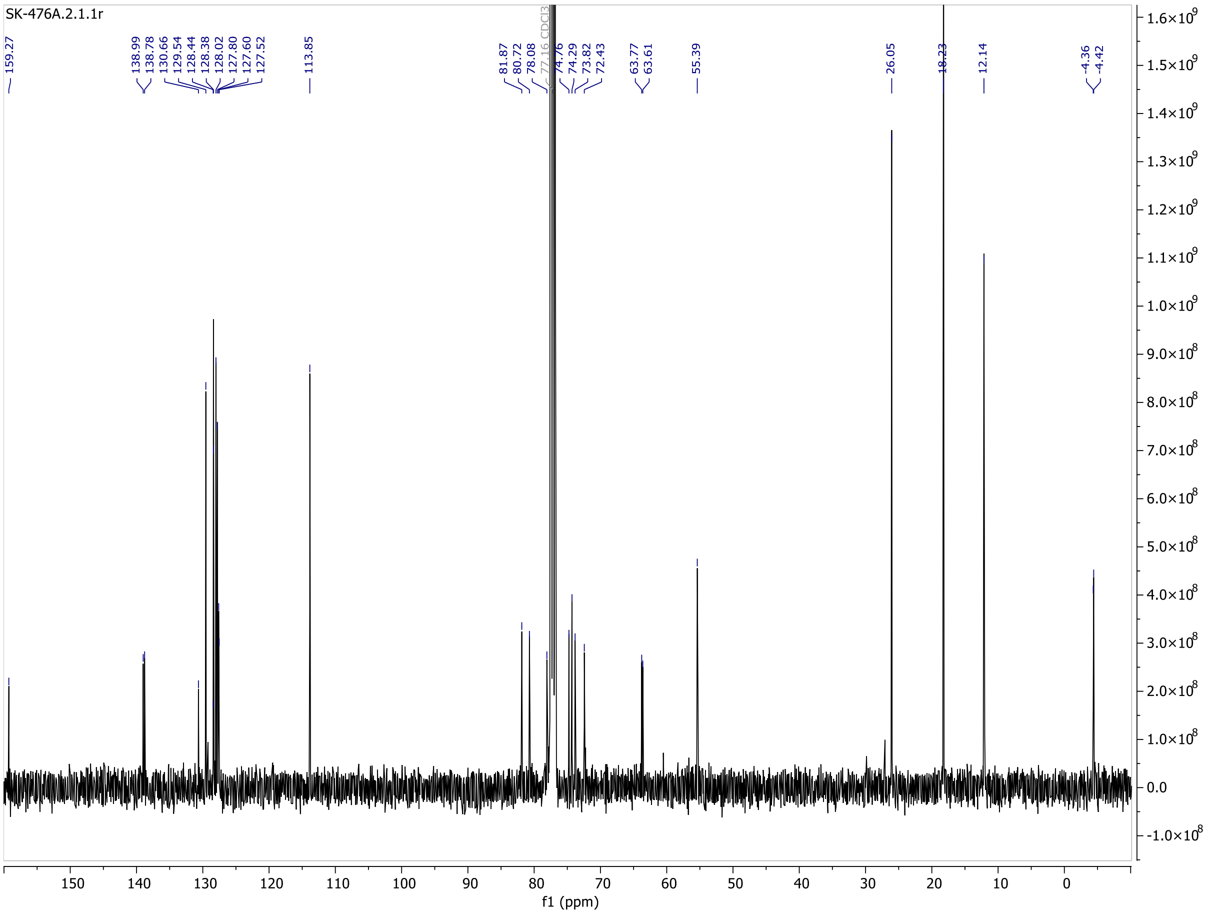
SK-476A.2.1.1r
f1 (ppm)

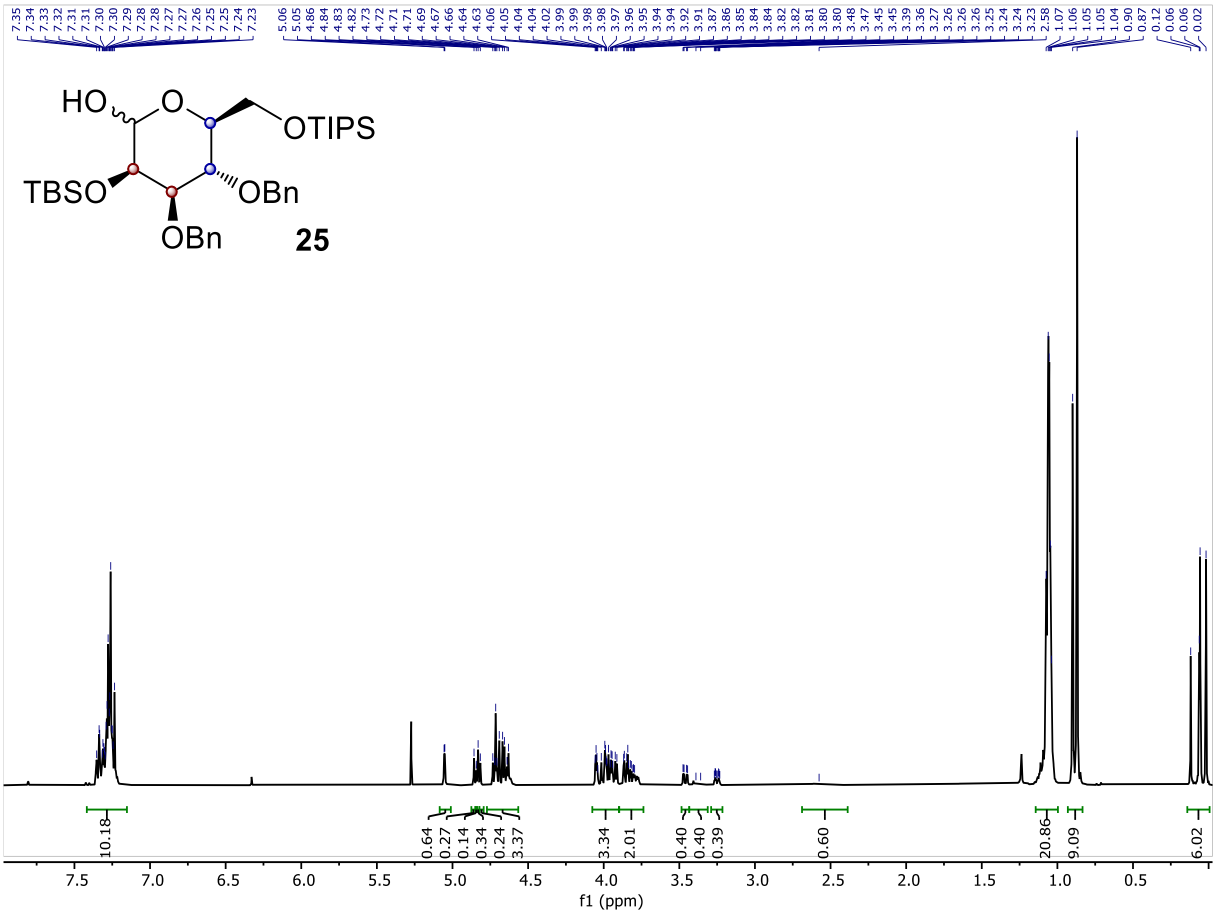
HO
O
OTIPS
TBSO
OBn
OBn
25
10.18
0.64
0.27
0.14
0.34
0.24
3.37
3.34
2.01
0.40
0.40
0.39
0.60
20.86
9.09
6.02
f1 (ppm)

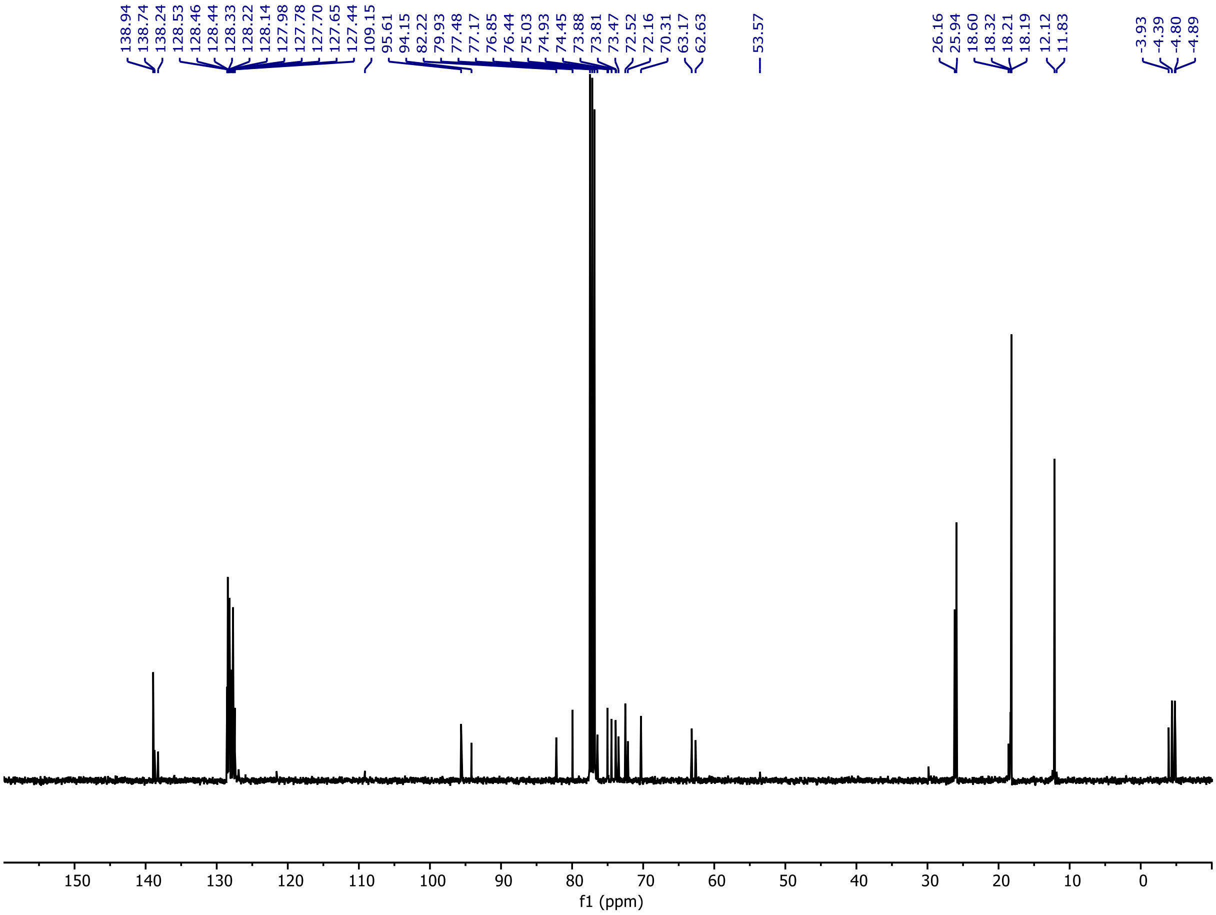
138.94
138.74
138.24
128.53
128.46
128.44
128.33
128.22
128.14
127.98
127.78
127.70
127.65
127.44
109.15
95.61
94.15
82.22
79.93
77.48
77.17
76.85
76.44
75.03
74.93
74.45
73.88
73.81
73.47
72.52
72.16
70.31
63.17
62.63
53.57
26.16
25.94
18.60
18.32
18.21
18.19
12.12
11.83
-3.93
-4.39
-4.80
-4.89
f1 (ppm)

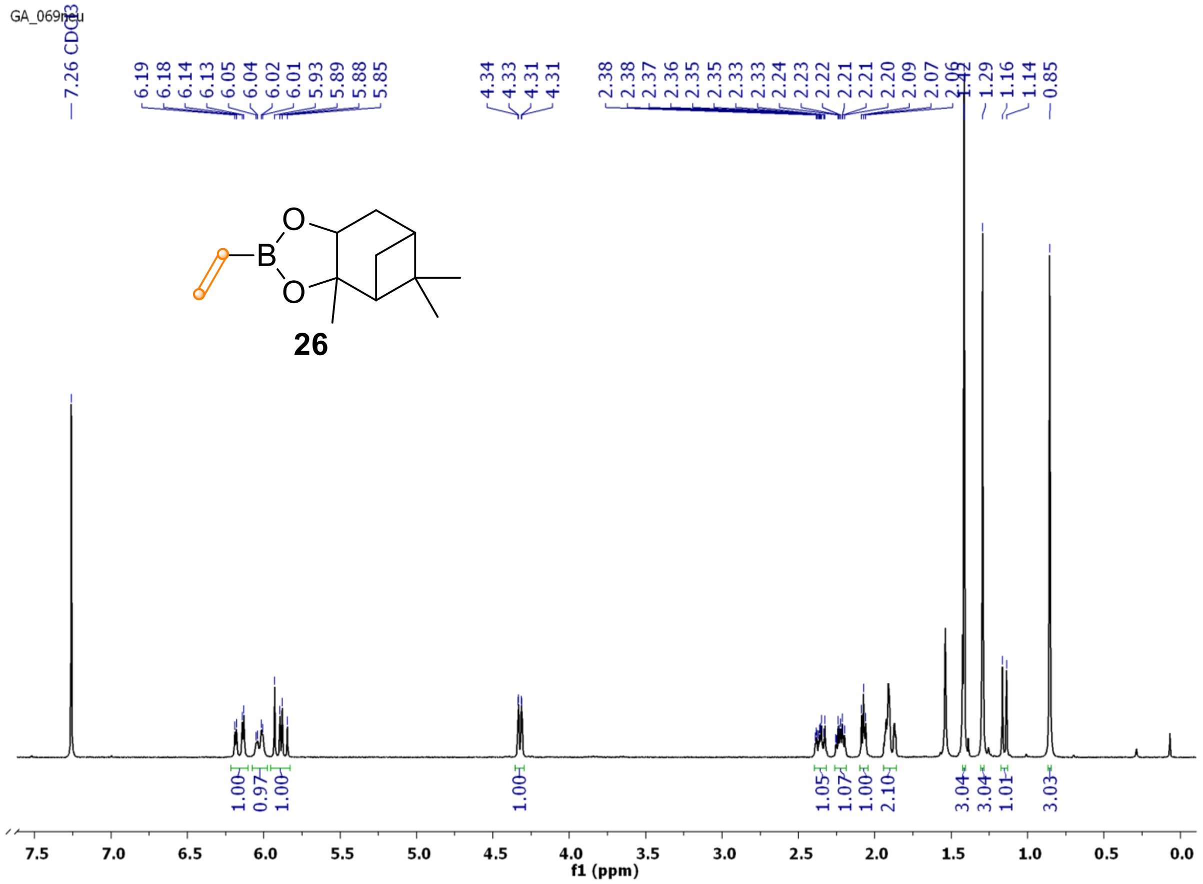
GA_069neu
7.26 CDCl3
6.19
6.18
6.14
6.13
6.05
6.04
6.02
6.01
5.93
5.89
5.88
5.85
4.34
4.33
4.31
4.31
2.38
2.38
2.37
2.36
2.35
2.35
2.33
2.33
2.24
2.23
2.22
2.22
2.21
2.21
2.20
2.09
2.07
2.06
1.42
1.29
1.16
1.14
0.85
O
B
O
26
1.00
0.97
1.00
1.00
1.05
1.07
1.00
2.10
3.04
3.04
1.01
3.03
f1 (ppm)

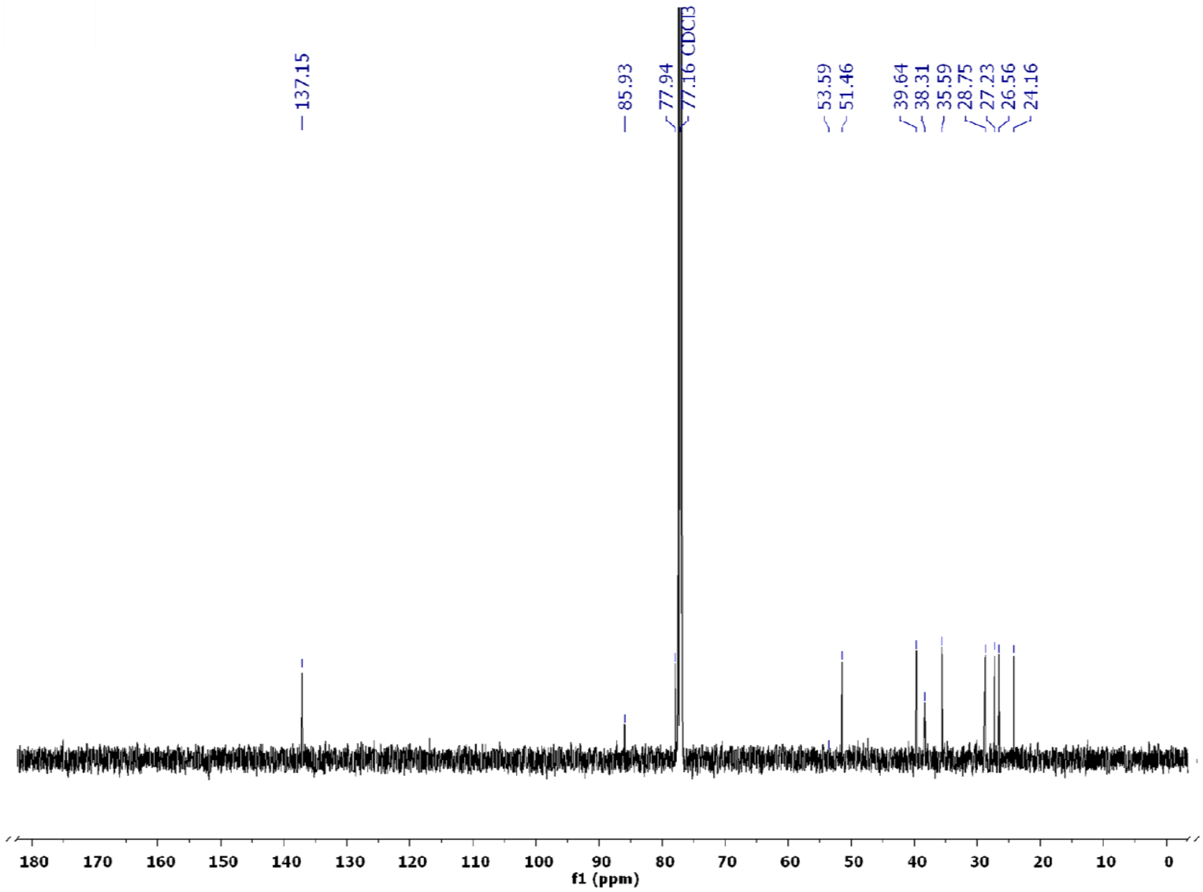
137.15
85.93
77.94
77.16 CDCl3
53.59
51.46
39.64
38.31
35.59
28.75
27.23
26.56
24.16
f1 (ppm)

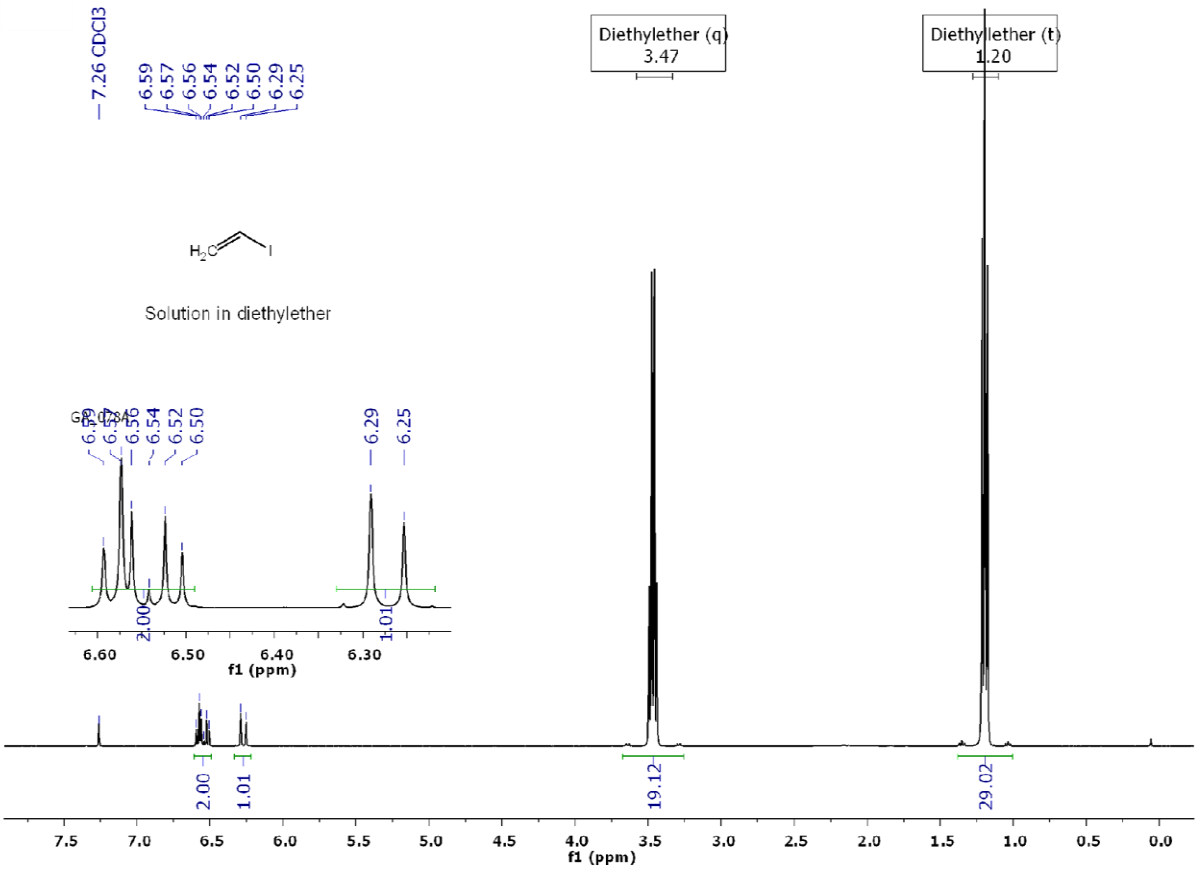
7.26 CDCl3
6.59
6.57
6.56
6.54
6.52
6.50
6.29
6.25
Diethylether (q)
3.47
Diethylether (t)
1.20
Solution in diethylether
2.00
1.01
19.12
29.02
f1 (ppm)

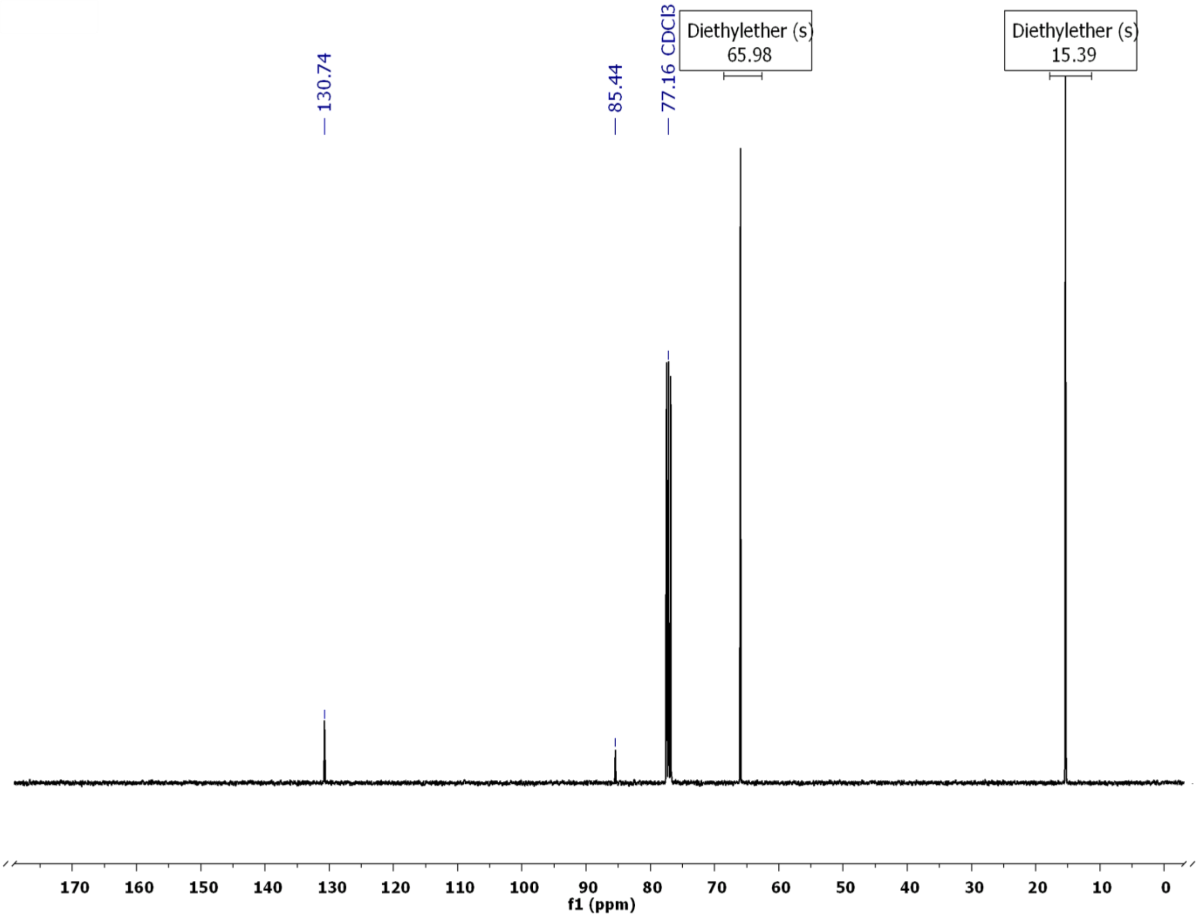
130.74
85.44
77.16 CDCl3
Diethylether (s)
65.98
Diethylether (s)
15.39
f1 (ppm)

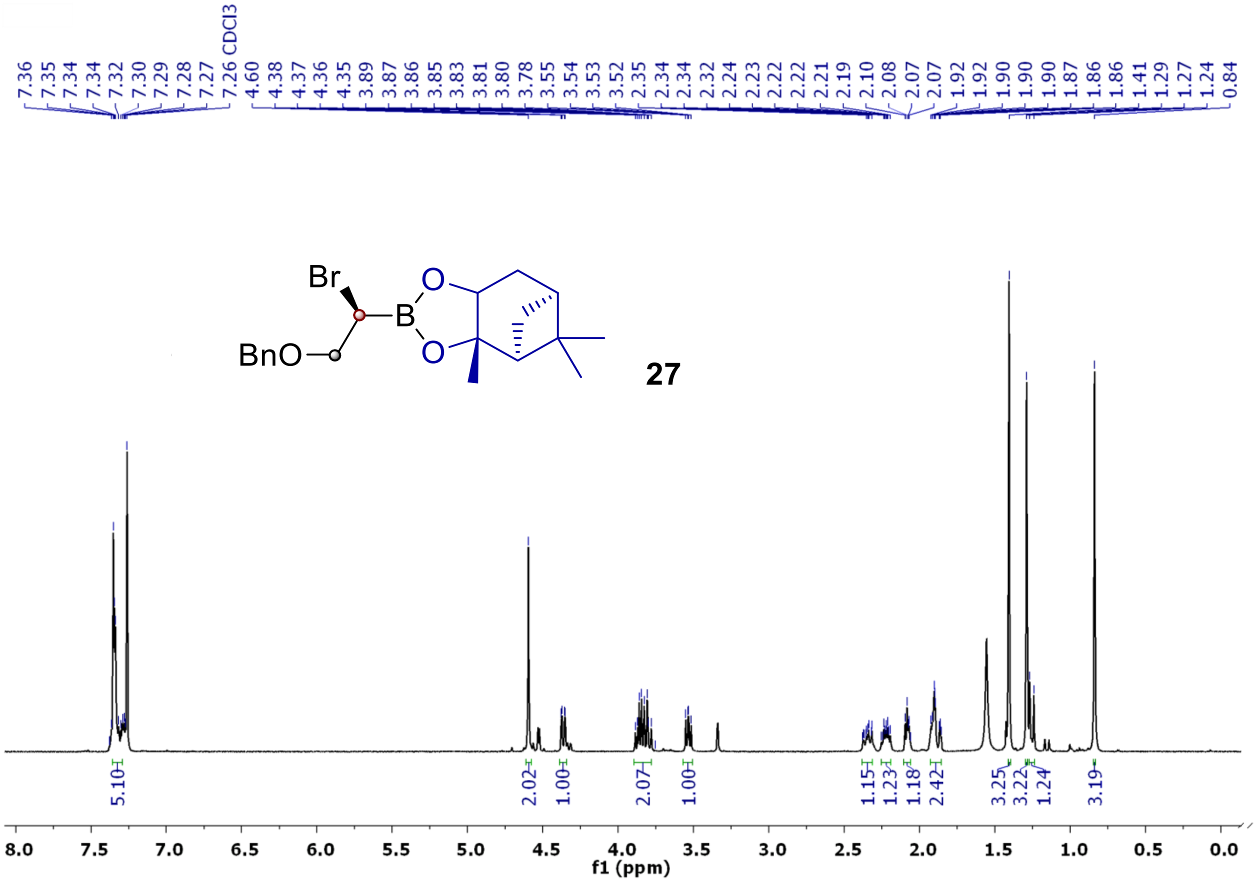

7.36 7.35 7.34 7.34 7.32 7.30 7.29 7.28 7.27 7.26 CDCl3 4.60 4.38 4.37 4.36 4.35 3.89 3.87 3.86 3.85 3.83 3.81 3.80 3.78 3.55 3.54 3.53 3.52 2.35 2.34 2.34 2.32 2.24 2.23 2.22 2.22 2.21 2.19 2.10 2.08 2.07 2.07 1.92 1.92 1.90 1.90 1.90 1.87 1.86 1.86 1.41 1.29 1.27 1.24 0.84
Br
B
O
O
BnO
27
5.10 2.02 1.00 2.07 1.00 1.15 1.23 1.18 2.42 3.25 3.22 1.24 3.19
8.0 7.5 7.0 6.5 6.0 5.5 5.0 4.5 4.0 3.5 3.0 2.5 2.0 1.5 1.0 0.5 0.0
f1 (ppm)

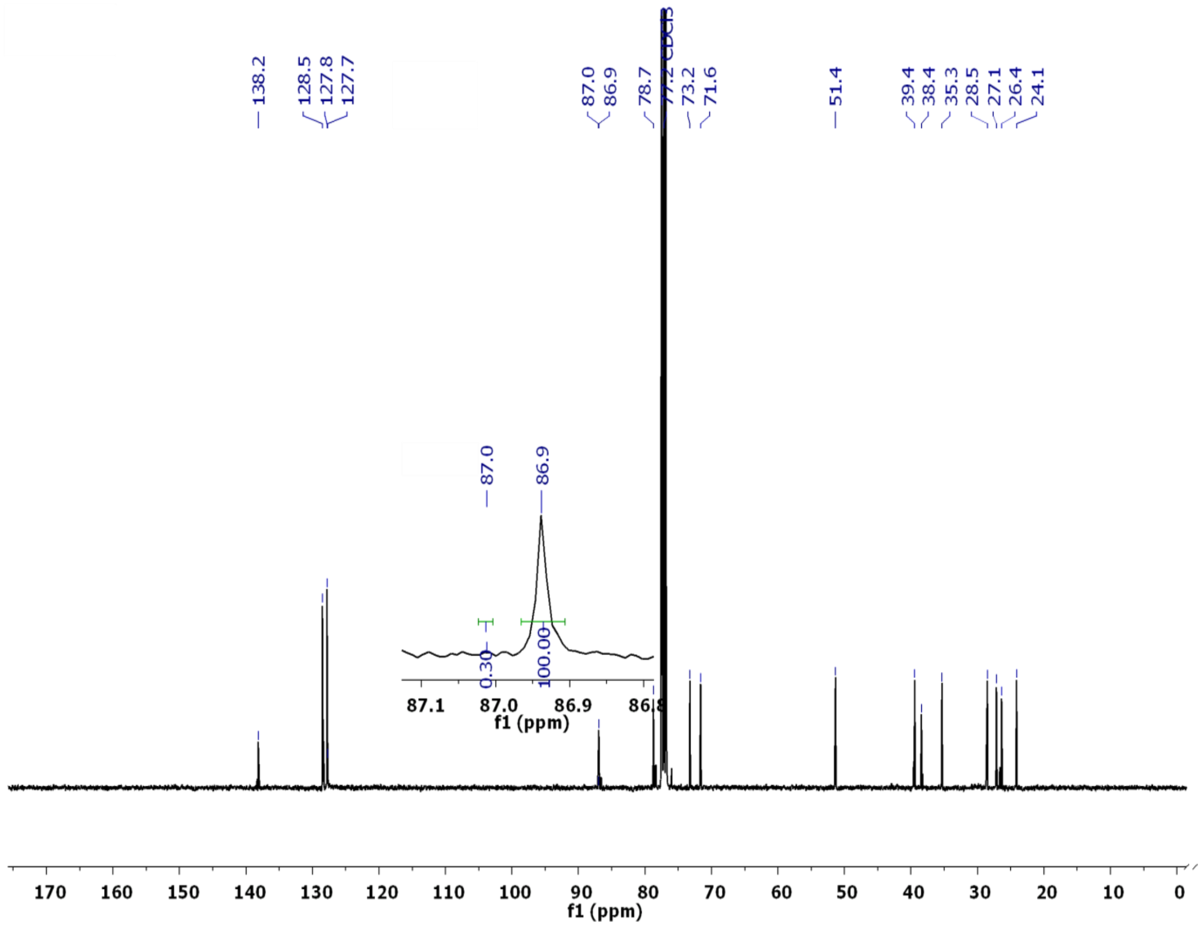

138.2 128.5 127.8 127.7 87.0 86.9 78.7 77.2 CDCl3 73.2 71.6 51.4 39.4 38.4 35.3 28.5 27.1 26.4 24.1
87.0 86.9
0.30 100.00
87.1 87.0 86.9 86.8
f1 (ppm)
170 160 150 140 130 120 110 100 90 80 70 60 50 40 30 20 10 0
f1 (ppm)

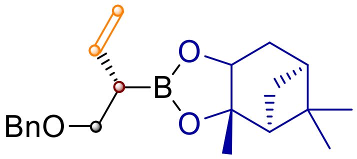

28

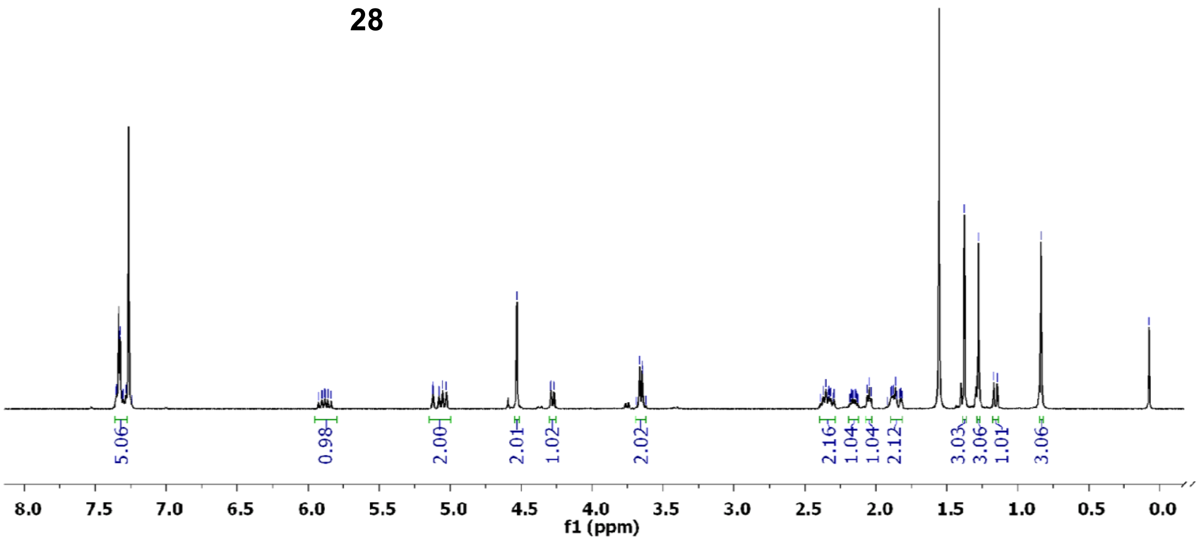

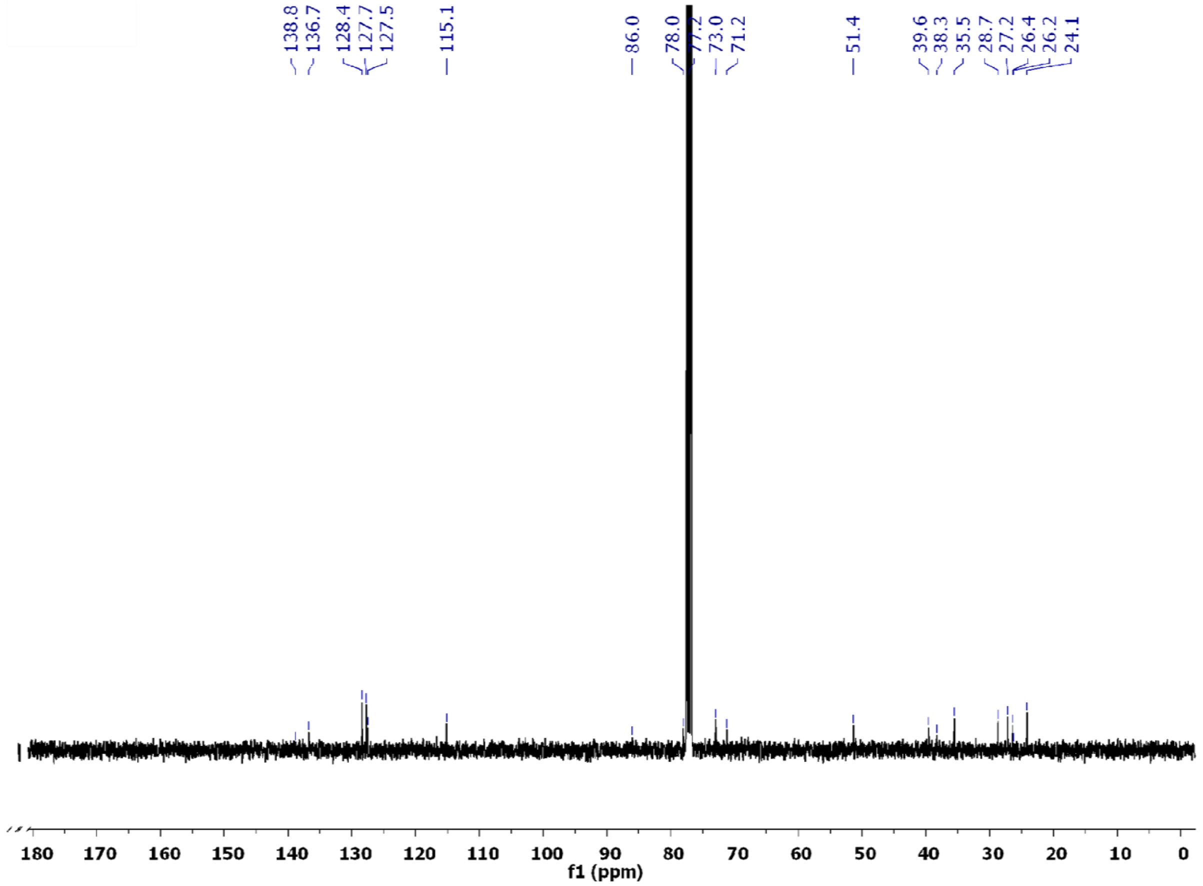

^{13}*C-resonances confirmed by comparison with original sample*

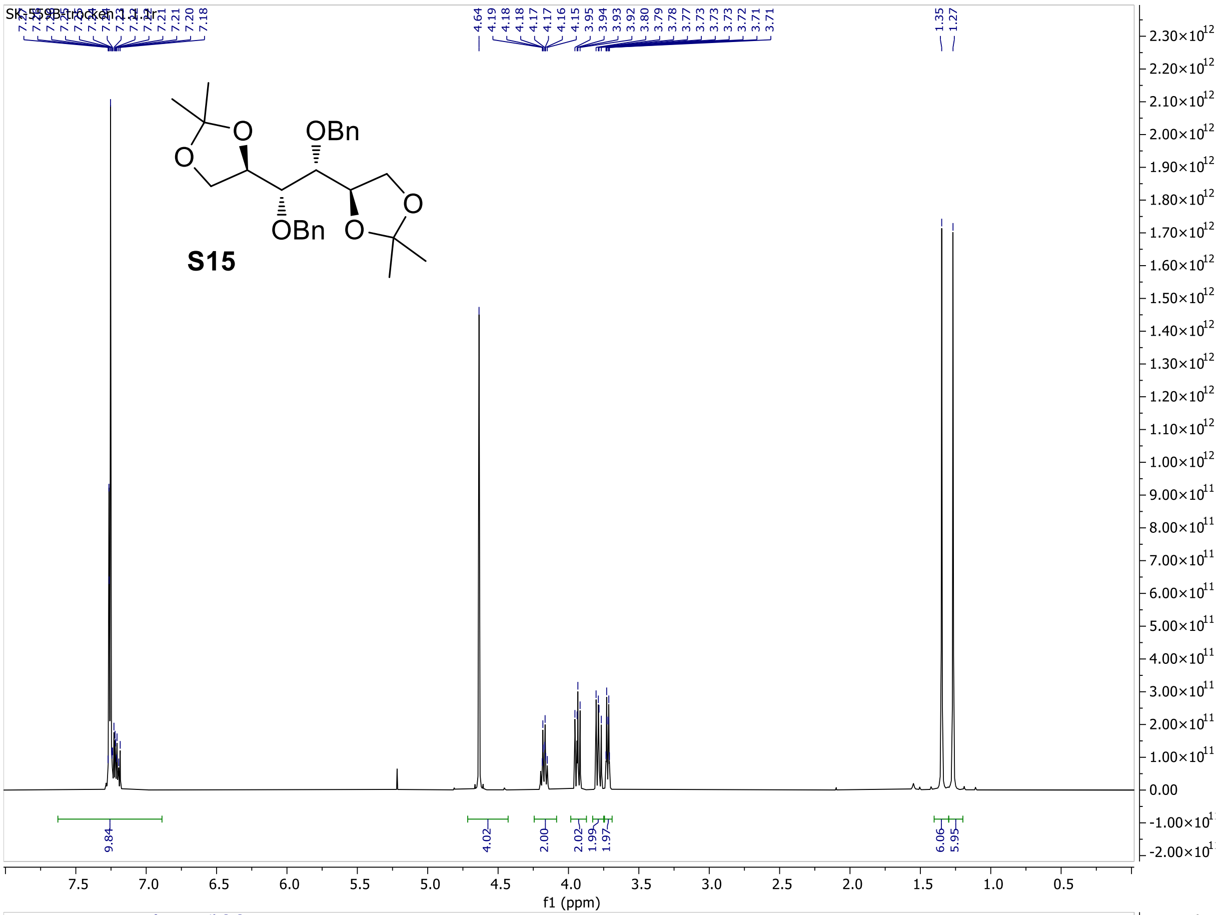
SK-559B-trocken.1.1.1r
7.21
7.20
7.18
4.64
4.19
4.18
4.18
4.17
4.17
4.16
4.15
3.95
3.94
3.93
3.92
3.80
3.79
3.78
3.77
3.73
3.73
3.73
3.72
3.71
3.71
1.35
1.27
O
O
OBn
OBn
O
O
S15
9.84
4.02
2.00
2.02
1.99
1.97
6.06
5.95
f1 (ppm)

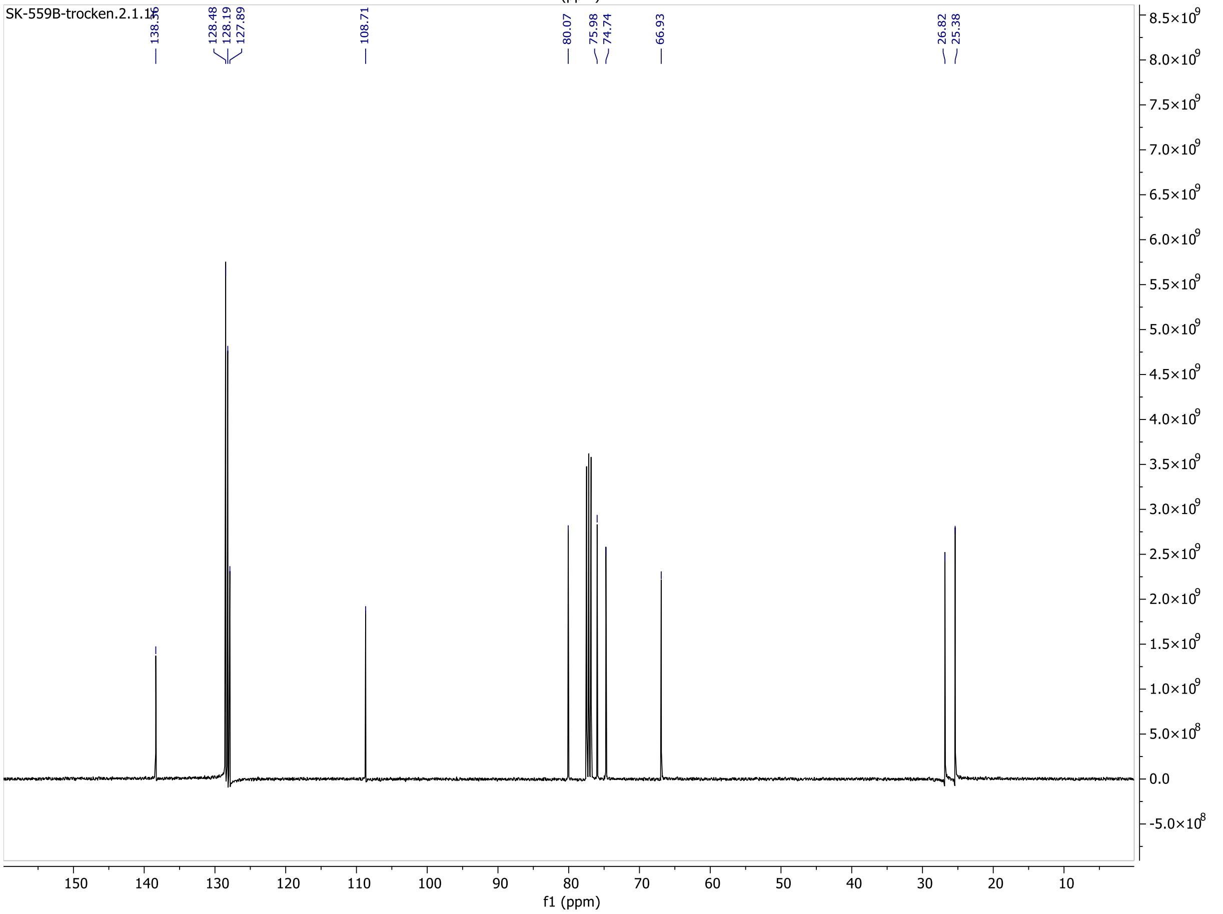
SK-559B-trocken.2.1.1r
138.56
128.48
128.19
127.89
108.71
80.07
75.98
74.74
66.93
26.82
25.38
f1 (ppm)

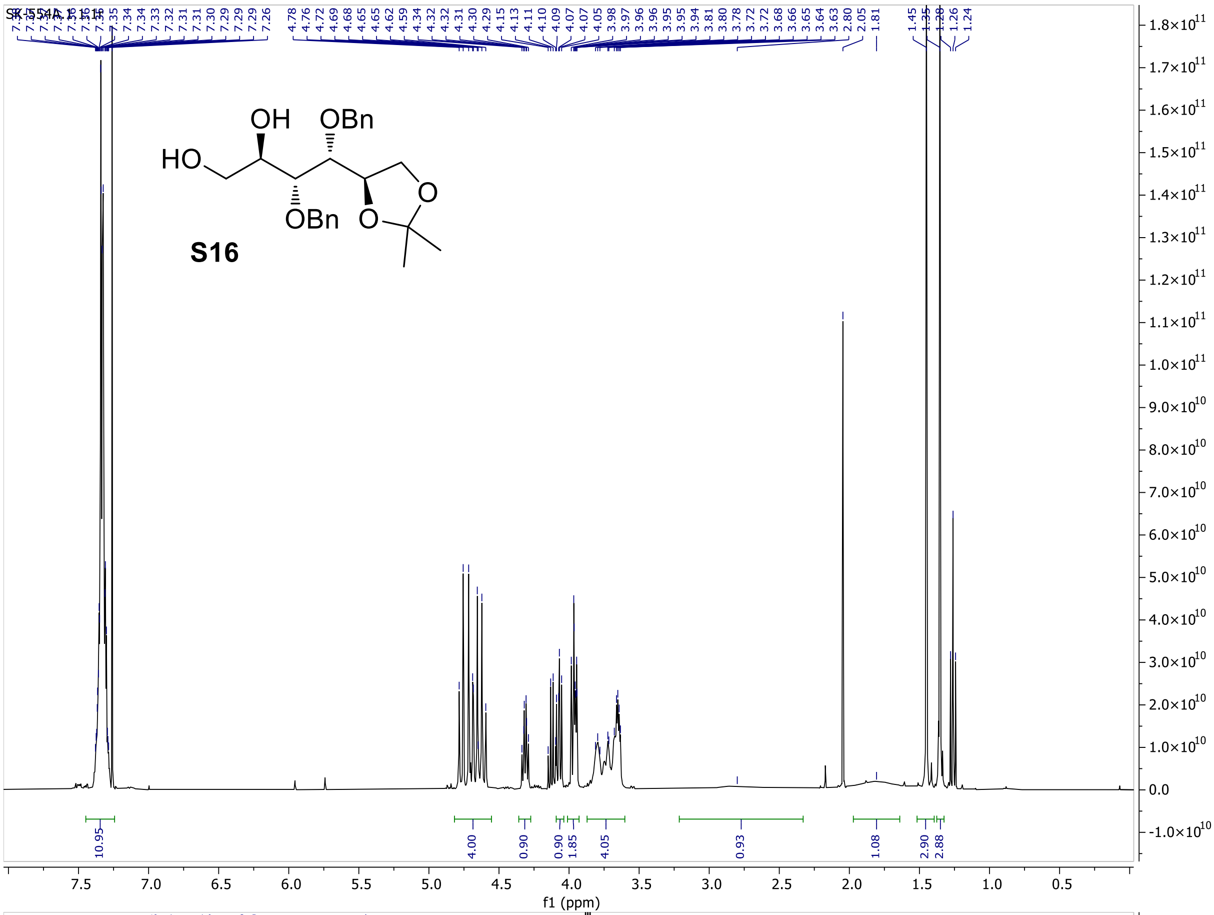
7.35 7.34 7.34 7.33 7.32 7.31 7.31 7.30 7.29 7.29 7.29 7.26
4.78 4.76 4.72 4.69 4.68 4.65 4.65 4.62 4.59 4.34 4.32 4.32 4.31 4.30 4.29 4.15 4.13 4.11 4.10 4.09 4.07 4.07 4.05 3.98 3.97 3.96 3.96 3.95 3.95 3.94 3.81 3.80 3.78 3.72 3.72 3.68 3.66 3.65 3.64 3.63 2.80 2.05 1.81
1.45 1.26 1.24
OH
OBn
HO
OBn
O
O
S16
10.95
4.00
0.90
0.90
1.85
4.05
0.93
1.08
2.90
2.88
f1 (ppm)

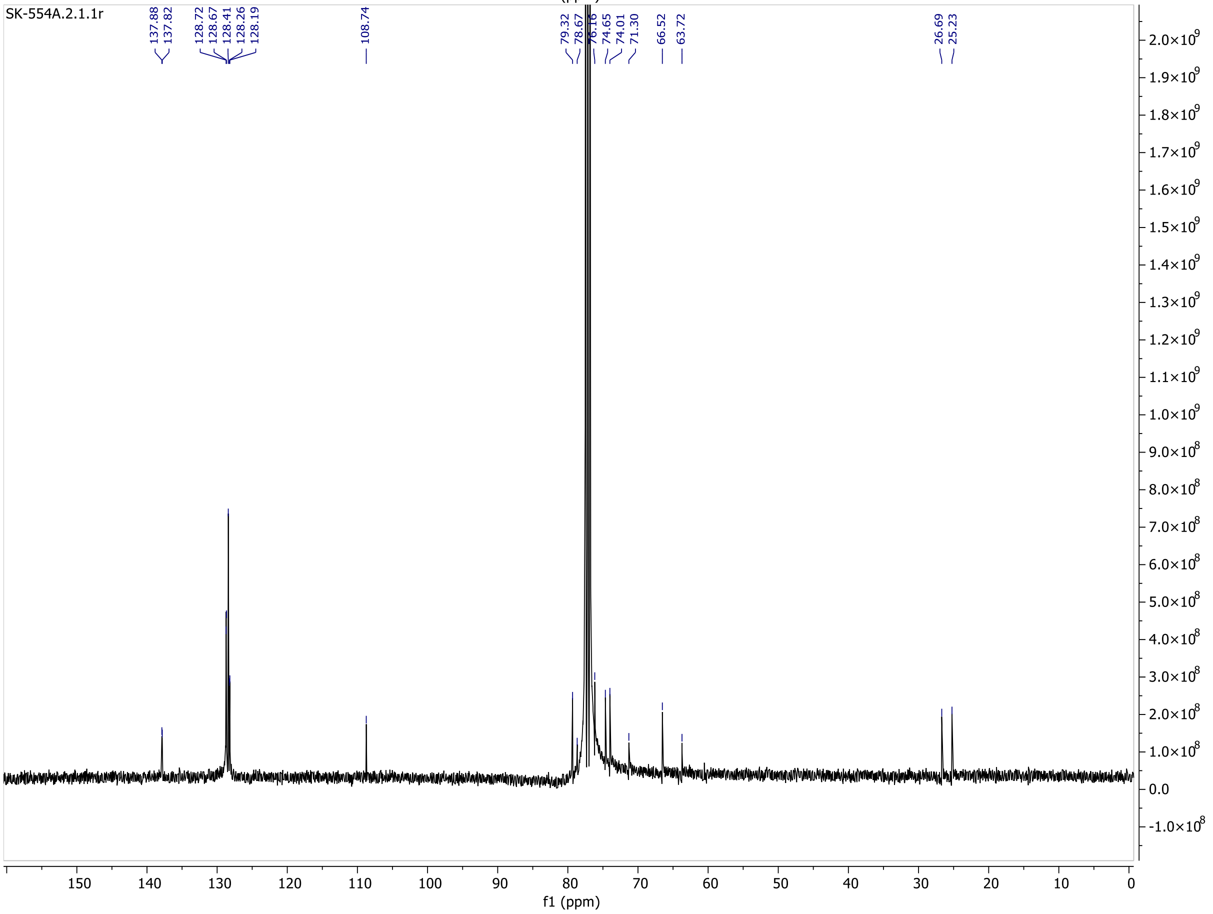
SK-554A.2.1.1r
137.88 137.82
128.72 128.67 128.41 128.26 128.19
108.74
79.32 78.67 74.65 74.01 71.30
66.52
63.72
26.69 25.23
f1 (ppm)

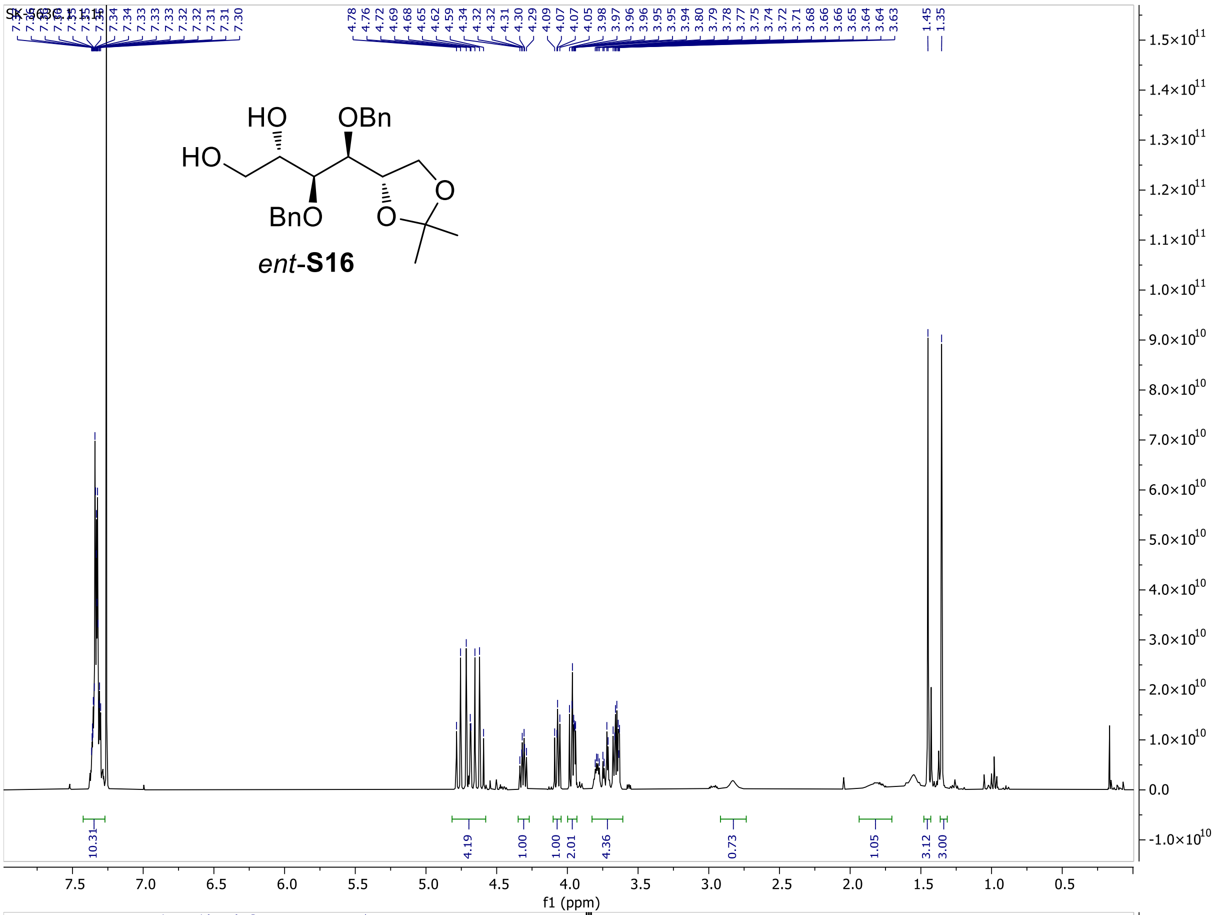

HO
OBn
HO
BnO
O
O
ent-**S16**
10.31
4.19
1.00
1.00
2.01
4.36
0.73
1.05
3.12
3.00
f1 (ppm)

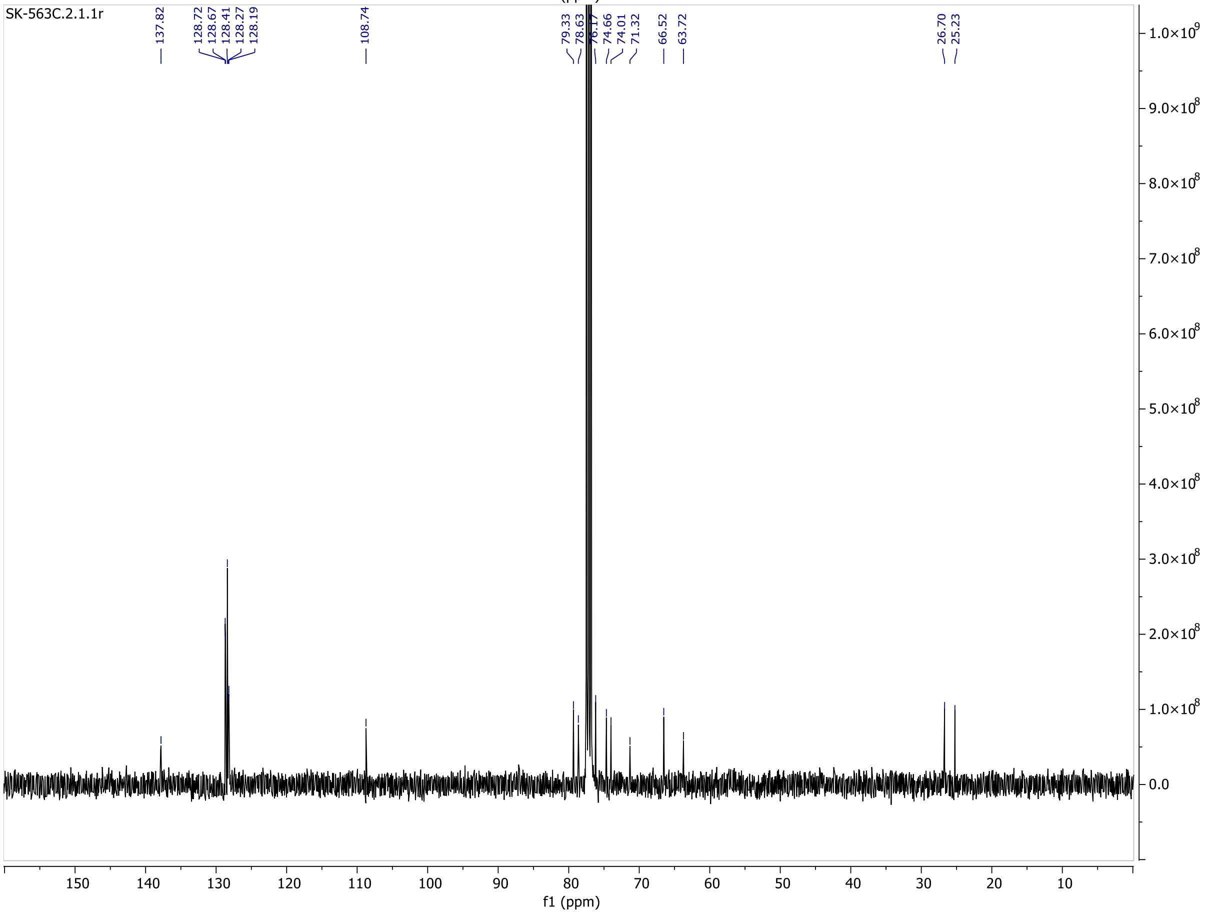

SK-563C.2.1.1r
137.82
128.72
128.67
128.41
128.27
128.19
108.74
79.33
78.63
74.66
74.01
71.32
66.52
63.72
26.70
25.23
f1 (ppm)
